# SNX9 Inhibits Cell Proliferation and Cyst Development in Autosomal Dominant Polycystic Kidney Disease via Activation of the Hippo-YAP Signaling Pathway

**DOI:** 10.3389/fcell.2020.00811

**Published:** 2020-08-21

**Authors:** Ai-Wen Shen, Li-Li Fu, Lu Lin, Bo Sun, Dong-Xu Song, Wu-Tao Wang, Yi-Hao Wang, Pei-Ran Yin, Sheng-Qiang Yu

**Affiliations:** ^1^Department of Nephrology, Changzheng Hospital, Naval Medical University, Shanghai, China; ^2^Department of Nephrology, The Second Affiliated Hospital of Soochow University, Suzhou, China; ^3^Division of Nephrology, Department of Medicine, The 5th Hospital of Sun Yat-sen University1, Zhuhai, China; ^4^Department of Nephrology, Second People’s Hospital of Fuyang City, Fuyang, China

**Keywords:** SNX9: sorting nexin 9, ADPKD: autosomal dominant polycystic kidney disease, signaling mechanism, the Hippo-YAP signaling pathway, Hippo-Yes associated pathway, therapy

## Abstract

Autosomal dominant polycystic kidney disease (ADPKD) is a complex process, involving the alteration of multiple genes and signaling pathways, and the pathogenesis of ADPKD remains largely unknown. Here, we demonstrated the suppressive role of sorting nexin 9 (SNX9) during ADPKD development. Sorting nexin 9 expression was detected in the kidney tissues of ADPKD patients, for the first time, and SNX9 expression was also detected in *Pkd1* knockout (*Pkd1*^–/–^) and control mice. Subsequently, a series of gain- and loss-of-function studies were performed, to explore the biological roles and underlying molecular mechanisms of SNX9 in ADPKD progression. The expression of SNX9 was significantly downregulated in ADPKD patients and *Pkd1*^–/–^ mice compared with control individuals and wild-type mice (Pkd1^+/+^), respectively. The ectopic expression of SNX9 significantly inhibited ADPKD cell proliferation, renal cyst formation and enlargement, whereas these effects were promoted by SNX9 silencing. Mechanistically, we found that SNX9 interacted directly with yes-associated protein (YAP) and increased the large tumor suppressor kinase 1-mediated phosphorylation of YAP, resulting in the cytoplasmic retention of YAP, the decreased transcriptional activity of the YAP/TEA domain transcription factor 4 complex, and, consequently, the inhibition of Hippo target gene expression and ADPKD development. Taken together, our findings provided novel insights into the role played by SNX9 during ADPKD pathogenesis and may reveal novel therapeutic approaches for ADPKD and related kidney diseases.

## Introduction

Autosomal dominant polycystic kidney disease (ADPKD) is a monogenic, inherited renal disease, with an incidence that ranges between 1/400 and 1/1000, worldwide (Chebib and Torres, 2016). Generally, ADPKD is caused by mutations in *PKD1* or *PKD2*, which encode polycystin (PC)1 or PC2, respectively (Padovano and Podrini, 2018). Autosomal dominant polycystic kidney disease is characterized by the formation and development of numerous renal cysts, which result in the massive enlargement of the kidney and may eventually result in end-stage renal disease (Wilson, 2004; Malekshahabi et al., 2019). Limited therapeutic options are currently available for ADPKD; thus, identifying the deregulated molecular targets and pathways that are associated with disease progression are of great importance for the development of mechanism-based therapeutic strategies (LaRiviere et al., 2015).

The Hippo-yes-associated protein (YAP) pathway plays vital roles in kidney homeostasis and ADPKD pathogenesis (Cai et al., 2018; Ma and Guan, 2018). This pathway is characterized by the mammalian sterile 20-like (MST)-large tumor suppressor (LATS) kinase cascade, which inactivates the downstream transcription coactivator YAP (Yu et al., 2015). Usually, after phosphorylation by the MST-LATS cascade, YAP is sequestrated in the cytoplasm, via 14-3-3 binding, and then subjected to ubiquitination and degradation. When the Hippo pathway is suppressed, YAP translocates into the nucleus and binds the TEA domain family (TEAD1–4) of transcription factors, to promote the expression of target genes involved in cell proliferation (Mo et al., 2014; Park et al., 2015). Although the core components of the Hippo-YAP pathway have been increasingly characterized, further in-depth exploration of the Hippo signaling pathway may extend our understanding of the pathophysiology underlying ADPKD and facilitate the identification of rational personalized therapies.

Sorting nexin 9 (SNX9) maps to human chromosome 6q25.3 and was first identified for its role in clathrin-mediated endocytosis (Bendris and Schmid, 2017). As a multifunctional protein, SNX9 plays vital roles in diverse endocytic vesicle sorting activities, the regulation of actin polymerization, cell migration and invasion, and cell division (Soulet et al., 2005; Shin et al., 2008; Yarar et al., 2008; Bendris et al., 2016a; Tanigawa and Maekawa, 2019). However, the expression pattern and potential biological functions of SNX9 during ADPKD remain unclear.

In this study, we demonstrated that SNX9 expression was decreased in ADPKD patients and *Pkd1*^–/–^ mice compared to control patients and mice, respectively. In addition, functional experiments revealed that SNX9 could inhibit cell proliferation, renal cyst formation, and growth. Further investigation into the molecular mechanisms suggested that SNX9 interacts directly with YAP and increases the large tumor suppressor kinase 1 (LATS1)-mediated phosphorylation of YAP, resulting in the cytoplasmic retention of YAP and the decreased transcriptional activity of YAP/TEA domain transcription factor 4 (TEAD4), which, consequently, inhibits the expression of Hippo target genes and prevents ADPKD development.

## Materials and Methods

### Tissue Specimens

Tissue samples from three ADPKD patients and three controls (normal subjects) were obtained from Shanghai Changzheng Hospital. This study was approved and supervised by the hospital Ethics Committee (Approval no. 2017SL039). Written informed consent was obtained from all participants.

### Animals

Mouse kidney tissues were collected from *Pkd1*^fl/fl^;*Cre*/*Esr1*^+^ (*Pkd1*^–/–^) ADPKD model mice and control *Pkd1*^fl/fl^;*Cre*/*Esr1*^–^ (wild-type, *Pkd1*^+/+^) littermates, which were generated as described previously by our facility (Yang et al., 2018).

### Cell Culture

Human immortalized ADPKD (WT9-12) cells and human renal cortical tubular epithelial cells (RCTECs) were kindly provided by Prof. Jing Zhou (Harvard Medical School, United States) (Loghman-Adham et al., 2003). Canine renal epithelial cells (MDCK) were gifts from Prof. Rudolf P. Wüthrich (University Hospital, Zürich, Switzerland) (Mangoo-Karim et al., 1989; Shi et al., 2018). All cells were cultured in Dulbecco’s modified Eagle medium (DMEM), containing 10% fetal bovine serum, and maintained at 37°C, in a humidified incubator containing 5% CO_2_. In addition, SNX9-depleted and control RCTEC cells were subjected to YAP/TEAD4 inhibitor verteporfin (1 μmol/L) or DMSO treatment for 24 h to determine the critical role of the Hippo-YAP signaling pathway in SNX9-induced inhibition of ADPKD development (Figure 5).

### Plasmid Construction and Transfection

The full-length SNX9 sequence was synthesized and then sub-cloned into the pLOV-EF1α-P2A plasmid (Obio, Shanghai, China), for use in SNX9 overexpression experiments. The small hairpin RNA (shRNA) constructs against SNX9 were obtained from Biolink (Shanghai, China), and the target sequences were as follows: sh-1: 5'-GCT GCT GAA CCT GGA AAT AAT-3'; sh-2: 5'-GGT TCC CAC AGA CTA CGT TGA-3'. All plasmid sequences were confirmed by DNA sequencing, before use. Transient transfection was performed using the Lipofectamine 2000 reagent (Invitrogen), according to the manufacturer's instructions. Lentiviruses were prepared in HEK-293T cells, by transfection with the indicated vectors and viral packaging constructs. Target cells were infected with lentivirus, in the presence of polybrene (6 mg/ml; Sigma-Aldrich), at a multiplicity of infection of 0.7. Two days after infection, the cells were selected with 2μg/ml puromycin for 2 weeks, to construct stable cell lines with SNX9 overexpression or knockdown.

### Quantitative Real–Time Polymerase Chain Reaction

Total RNA from tissues or cells was extracted with Trizol reagent (Invitrogen, Carlsbad, CA, United States). Reverse transcription was performed with 1 μg total RNA, using the PrimeScript RT reagent kit (TaKaRa, Dalian, China). Each quantitative real–time polymerase chain reaction (qRT–PCR) analysis was performed with 1 μl cDNA, using the ABI 7500 Sequence Detection System (Applied Biosystems, Foster, United States). The relative expression levels of mRNA were calculated using the 2^–Δ^ ^Ct^ method. Glyceraldehyde 3-phosphate dehydrogenase (GAPDH) was used as an internal control. The primer sequences were as follows. SNX9: forward: 5'-TGT AGG TGG AGG ATG GCT GGA AG-30, reverse: 50-CGA ACT GGC CTG AGC TGT GC- 30; GAPDH: forward: 50-GGC ATC CTG GGC TAC ACT GA-30, reverse: 50-GAG TGG GTG TCG CTG TTG AA-30; connective tissues growth factor (CTGF): forward: 50-AAA AGT GCA TCC GTA CTC CCA-30, reverse: 50-CCG TCG GTA CAT ACT CCA CAG-30; cysteine-rich angiogenic inducer 61 (CYR61): forward: 50-GGT CAA AGT TAC CGG GCA GT-30, reverse: 50-GGA GGC ATC GAA TCC CAG C-30; and caudal-type homeobox 2 (CDX2): forward: 50-GAC GTG AGC ATG TAC CCT AGC-30, reverse: 50-GCG TAG CCA TTC CAG TCC T-30.

### Western Blotting Analysis

Total protein was extracted from human tissues or cells, using RIPA lysis buffer, containing protease inhibitors (Biyuntian Biotech, China). The nuclear protein was prepared using a Nuclear and Cytoplasmic Protein Extraction Kit (Beyotime, Shanghai, China) according to the manufacturer’s instructions. The bicinchoninic acid (BCA) assay was used to determine the lysate protein concentrations. Equal amounts of protein lysates were resolved by sodium dodecyl sulfate–polyacrylamide gel electrophoresis (SDS–PAGE) and transferred, electrophoretically, onto a polyvinylidene fluoride membrane (Millipore, United States). The membrane was then incubated with primary antibodies (Supplementary Table S1), at 4°C overnight. After incubation with horseradish peroxidase (HRP)–conjugated secondary antibody, the signals were detected with enhanced chemiluminescent (ECL) detection reagents (Millipore, United States).

### Immunohistochemistry Analysis

Human and mouse tissue samples were stained, according to standard immunohistochemistry (IHC) protocols, as previously described (Jing et al., 2018). After deparaffinization and rehydration, the slides were subjected to antigen retrieval using citrate buffer (pH 6.0), at 100°C for 6 min, endogenous peroxidase was blocked with 3% H_2_O_2_, and the slides were subsequently incubated with primary antibody (Supplementary Table S1), at 4°C, overnight. Finally, the reaction was visualized with 3, 3'-diaminobenzidine (DAB), counterstained with hematoxylin, and observed with a microscope.

### Cell Counting Kit–8 Assay

Cell proliferation was analyzed using the cell counting kit–8 (CCK–8) assay. Briefly, the indicated cells were seeded in 96–well plates, at a density of 1 × 10^3^ cells/well, in 100 μl medium, and cell viability was measured continuously, from day 1 to 4. At each time-point, cells were incubated with 10 μl CCK-8 reagent, at 37°C for 2 h. The absorbance of each sample was then measured at 450 nm.

### Colony Formation Assay

Colony formation assay was performed to determine the effect of modified SNX9 expression on ADPKD cell proliferation. Approximately 0.5 × 10^3^ cells were individually plated in each well of a 6-well plate and incubated with complete DMEM medium, for 14 days. Cells were then fixed with 4% paraformaldehyde, for 15 min, and stained with Giemsa solution, for visualization and quantification.

### Ethynyldeoxyuridine (EdU) Incorporation Assay

Ethynyldeoxyuridine (EdU) incorporation assay was performed to explore the effect of modified SNX9 expression on ADPKD cell proliferation. The indicated cells were first cultured with the EdU labeling medium (RiboBio, Guangzhou, China), for 2 h. Then, the cells were fixed with 4% paraformaldehyde, for 30 min, incubated with 1% Triton X-100, for 30 min, and dyed with Apollo, for 30 min. Finally, Hoechst was used to dye DNA, and the cells were visualized with a confocal microscope.

### MDCK Cyst Model

To explore the effect of SNX9 on ADPKD development, a renal cyst formation model was developed using a three-dimensional culture of MDCK cells, for one week (Wang et al., 2015; Shi et al., 2018). Approximately 2 × 10^4^ MDCK cells were suspended in 2 ml collagen matrix (1,200 μl Collagon I, 800 μl DMEM, 40 μl 1M HEPES, and 24 μl 1M NaOH). Then, 400 μl cell suspensions were plated onto 24-well plates and cultured at 37°C, for 15 min. After the collagen matrix is solidified, 500 μl complete medium was plated to each well, and then subjected to culture for 7 days. The percentage of renal cyst formation was quantified based on at least 100 cysts per group. Only cysts larger than 50 μm were considered to be positive and counted for quantification.

### RNA Sequencing Analysis

The RNA-sequencing analysis was conducted, using SNX9-depleted RCTEC and control cells, to explore the molecular mechanism by which SNX9 inhibits ADPKD evolution. Total RNA was extracted with Trizol reagent. Strand-specific RNA-seq libraries were prepared, purified, subjected to quality control, and sequenced, using HiSeq 3000 (Illumina, United States). Data processing was performed using LifeScope v2.5.1, to align the reads to the genome, generate raw counts corresponding to known genes, and calculate the expression values.

### Immunofluorescence Assay

The immunofluorescence assay was conducted to examine the influence of SNX9 on subcellular localization of YAP protein. The indicated cells were seeded onto sterilized coverslips, incubated for 36 h, fixed in 4% paraformaldehyde for 20 min, treated with 0.5% Triton X-100 for 5 min, and then blocked with 1% bovine serum albumin (BSA), for 1 h. Cells were then incubated with anti-YAP antibody (14074, Cell Signaling Technology, United States), at 4°C overnight, followed by incubation with fluorescent secondary antibody, at room temperature for 1 h, and counterstaining with 4',6-diamidino-2-phenylindole (DAPI). Cells were imaged with a confocal microscope.

### Luciferase Reporter Assay

The formation of YAP/TEAD4 complex activates the transcription of Hippo signaling target genes, and CTGF (Connective tissue growth factor) is one of the most important transcriptional targets of YAP/TEAD4 complex (Zhao et al., 2010). We thus constructed the YAP/TEAD4 luciferase reporter by subcloning the promoter of CTGF into the pGM-CMV-Luc vector (Yeasen Biotech, Shanghai, China). Then indicated cells were co-transfected with vectors with modified SNX9 expression, and YAP/TEAD4 luciferase reporter using Lipofectamine 2000 reagent (Invitrogen). Each group was run in triplicate in 96-well plates. The luciferase activity was detected after 48 h of transfection using a Dual-Luciferase Reporter Assay System (Promega). Renilla luciferase activity was normalized against Firefly luciferase activity.

### Co-immunoprecipitation Assay

Indicated cells were lysed to obtain the total protein. Then, 500 μg protein was incubated with 2 μg primary antibody (or control IgG) (Supplementary Table S1) and 20 μl A/G PLUS-Agarose beads (Santa Cruz Biotechnology, CA, United States), at 4°C overnight. After incubation, the beads were separated from the lysis buffer, washed five times in cold phosphate-buffered saline, and then subjected to standard western blotting analysis.

### Statistical Analysis

To compare the difference of continuous variables, we used Shapiro–Wilk Normality Test method to test their distribution first. We used parametric methods (e.g., *T*-test between two groups and one-way ANOVA analysis among multiple groups) to compare the difference when variables had a normal distribution. Otherwise, we would use Mann–Whitney U and Kruskal–Wallis H as nonparametric test method to compare the difference between two groups or among multiple groups, respectively. For multiple groups’ comparison, further post-hoc multiple comparisons were achieved by Nemenyi test. Two-tailed *P* value <0.05 was considered statistically difference.

## Results

### SNX9 Is Significantly Downregulated in ADPKD Patients and *Pkd1*^–/–^ Mice

**FIGURE 1:**
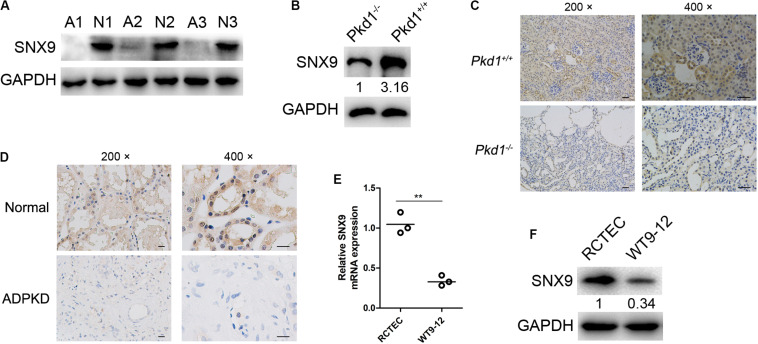
SNX9 is significantly downregulated in ADPKD patients and *Pkd1*^–/–^ mice. **(A)** The protein expression levels of SNX9 were examined by western blot analysis, using kidney tissues from three ADPKD patients (A1, A2, and A3) and three normal controls (N1, N2, and N3). **(B)** The protein level of SNX9 was examined by western blot analysis using kidney tissues from wild-type mice (*Pkd1*^+/+^) and *Pkd1* knockout mice (*Pkd1*^–/–^). **(C)** Immunohistochemistry (IHC) analysis of SNX9 expression was conducted, using kidney tissues from *Pkd1*^+/+^ and *Pkd1*^–/–^ mice. Representative IHC images showing the cellular distribution of SNX9. Scale bars, 200 μm. **(D)** IHC analysis of SNX9 expression was conducted using kidney tissues from normal controls and ADPKD patients. Representative IHC images showing the cellular distribution of SNX9. Scale bars, 200 μm. **(E)** The mRNA levels of SNX9 in RCTEC and WT9-12 cells were examined by qRT-PCR. Data are presented with scatterplots and mean values; *n* = 3. **indicates *P* < 0.01 by Student’s *t*-test. **(F)** The protein levels of SNX9 in RCTEC and WT9-12 cells were determined by Western blot analysis.

The expression of SNX9 was first investigated by western blot analysis, using kidney tissues from three ADPKD patients and three normal controls. As shown in Figure 1A, the SNX9 protein levels in ADPKD patients were significantly lower than those in normal controls. Then, the SNX9 expression levels were examined using kidney tissues from wild-type mice (*Pkd1*^+/+^), simulating normal people, and *Pkd1* knockout mice (*Pkd1*^–/–^), modeling ADPKD patients. The results also showed the decreased expression levels of SNX9 in ADPKD model mice (Figure 1B). In addition, IHC analysis of mouse and human kidney tissues demonstrated that SNX9 was distributed in both the nucleus and the cytoplasm, but predominantly in the cytoplasm (Figures 1C,D). Subsequently, SNX9 expression was detected in the human ADPKD cell line WT9-12 and normal RCTECs. Similarly, both SNX9 mRNA and protein levels were significantly downregulated in WT9-12 cells compared with those in RCTEC cells (Figures 1E,F). Taken together, our data clearly indicated the decreased expression of SNX9 in ADPKD patients and *Pkd1*^–/–^ mice compared with normal controls and wild-type mice.

### SNX9 Inhibits Cell Proliferation and Renal Cyst Formation and Enlargement

**FIGURE 2:**
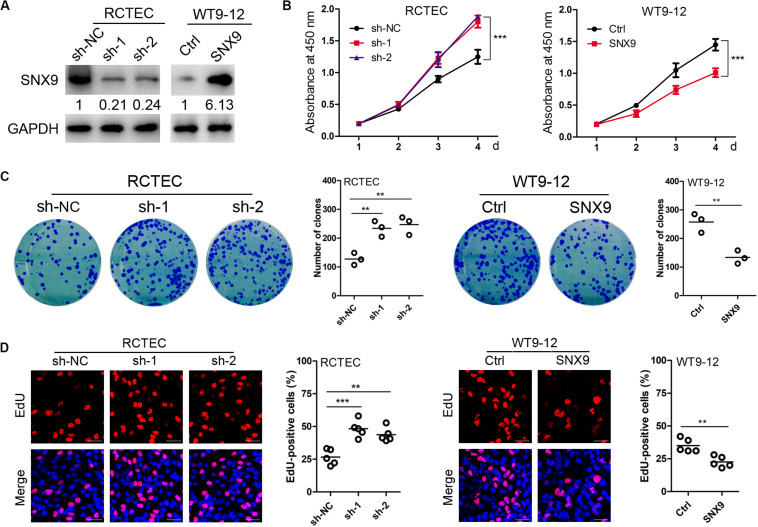
SNX9 inhibits ADPKD cell proliferation. **(A)** Effects of SNX9 overexpression and silencing were examined by Western blot analysis. **(B)** CCK-8 assay, using the indicated cells with modified SNX9 expression levels. ***Indicates *P* < 0.001 by Student’s *t*-test, *n* = 5. **(C)** Cell colony formation assay, using the indicated cells with modified SNX9 expression levels. Data are presented with scatterplots and mean values; *n* = 3. **Indicates *P* < 0.01 by Student’s *t*-test. **(D)** The indicated cells, with modified SNX9 expression levels, were subjected to EdU incorporation assay. Representative micrographs and the quantification of EdU labeling in the indicated cells are shown. Data are presented with scatterplots and mean values; *n* = 5. **Indicates *P* < 0.01; ***Indicates *P* < 0.001 by Student’s *t*-test.

To investigate the impacts of SNX9 on cell proliferation, gain- and loss-of-function studies were performed. We stably overexpressed SNX9 in WT9-12 cells, due to the relatively reduced level of SNX9 background expression, and constructed two stable knockdown RCTEC cell lines, which show relatively increased SNX9 expression (Figure 2A). As determined by the CCK-8 assay, the depletion of SNX9 significantly enhanced cell viability, whereas SNX9 overexpression compromised cell proliferation (Figure 2B). In addition, the colony formation assay revealed that SNX9 depletion resulted in significantly increased colony numbers, whereas SNX9 overexpression decreased colony numbers, compared with the numbers in control cells (Figure 2C). Consistently, the EdU incorporation assay demonstrated a significant increase in the percentage of EdU-positive cells in SNX9-depleted RCTEC cells, whereas a decrease was observed in SNX9-overexpressing WT9-12 cells, compared with that in control cells (Figure 2D). Collectively, these data suggested a suppressive role for SNX9 in ADPKD cell proliferation.

**FIGURE 3:**
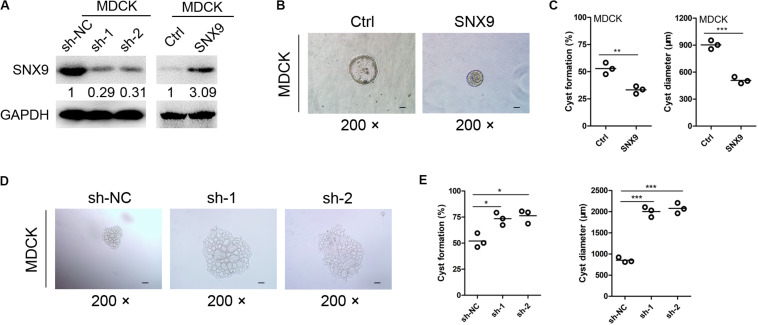
SNX9 inhibits renal cyst formation and enlargement. **(A)** The SNX9 overexpression and silencing effects were examined by Western blot analysis. **(B)** SNX9-overexpressing and control MDCK cells were subjected to three-dimensional cultures, to establish a renal cyst formation model. Representative cysts are shown. Scale bars, 200 μm. **(C)** The percentage of cyst formation and the cyst diameters of the indicated cells are shown. Data are presented with scatterplots and mean values; *n* = 3. **Indicates *P* < 0.01; ***indicates *P* < 0.001 by Student’s *t*-test. **(D)** SNX9-depleted and control MDCK cells were subjected to three-dimensional cultures, to establish a renal cyst formation model. Representative cysts are shown. Scale bars, 200 μm. **(E)** The percentage of cyst formation and cyst diameters of the indicated cells are shown. Data are presented with scatterplots and mean values; *n* = 3. *Indicates *P* < 0.05; ***indicates *P* < 0.001 by Student’s *t*-test.

To further investigate the effects of SNX9 on renal cyst development, an *in vitro* cyst formation model was developed, using a three-dimensional culture of MDCK cells with modified SNX9 expression (Figure 3A). As shown in Figure 3B, the cysts formed by control MDCK cells consisted of a central lumen and a surrounding monolayer of polarized cells. In contrast, SNX9-overexpressing MDCK cells formed cyst-like cell clusters, that showed no discernible lumens. The percentage of SNX9-overexpressing MDCK cells that formed renal cysts was 33.3 ± 3.4%, which was significantly lower than that observed for control MDCK cells (52.9 ± 5.3%) (Figure 3C). In addition, the cyst diameters in the SNX9-overexpressing MDCK group (505 ± 30 μm) was significantly lower than that in the control MDCK group (905 ± 55 μm) (Figure 3C). In contrast, SNX9 silencing in MDCK cells resulted in significant increases in both the percentage of renal cyst formation and cyst diameter compared with those in control cells (Figures 3D,E). Accordingly, we concluded that SNX9 inhibits renal cyst formation and enlargement in ADPKD cells.

### SNX9 Inhibits ADPKD Progression via the Activation of the Hippo Signaling Pathway

**FIGURE 4:**
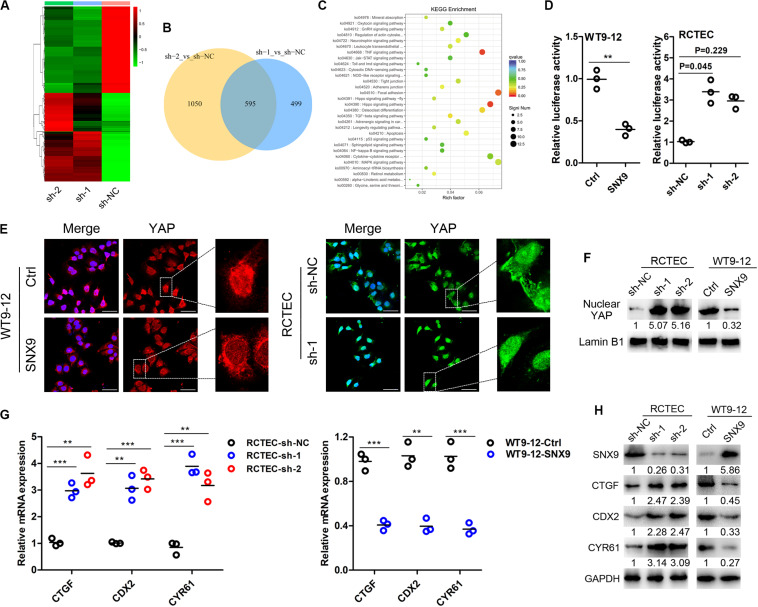
SNX9 activates the Hippo signaling pathway in ADPKD cells. **(A)** RNA sequencing analysis was conducted in SNX9-depleted RCTEC (sh-1 and sh-2) and control (sh-NC) cells. **(B)** A Venn diagram showing the differentially expressed genes (DEGs) identified in cells transfected with each of the two SNX9 shRNAs, as identified by RNA-sequencing analysis. **(C)** KEGG analysis of the RNA-seq data from SNX9-depleted RCTEC and control cells. **(D)** YAP/TEAD4 luciferase reporter assay was performed in WT9-12 and RCTEC cells with modified SNX9 expression levels. Data are presented with scatterplots and mean values, *n* = 3. For WT9-12 cells, **indicates *P* < 0.01 by Student’s *t*-test. For RCTEC cells, a boundary significant trend was observed among the sh-NC, sh-1 and sh-2 groups (*P* = 0.051) using Kruskal–Wallis H test. Compared with sh-NC group, the YAP/TEAD4 transcriptional activity was significantly promoted by transfection of sh-1 (*P* = 0.045), but not by sh-2 (*P* = 0.229) using Nemenyi test. **(E)** Representative immunofluorescence images of the indicated cells show the subcellular YAP localization. YAP was stained with red color in WT9-12 cells and green color in RCTEC cells. The nuclei were counterstained with DAPI (blue). Scale bars, 50 μm. **(F)** Nuclear fractions were prepared and then subjected to western blot analysis to detect nuclear YAP protein levels. Lamin B1 was used as a loading control. **(G)** qRT-PCR analysis was performed to examine the mRNA expression levels of the Hippo target genes. Data are presented with scatterplots and mean values, *n* = 3. **Indicates *P* < 0.01; ***indicates *P* < 0.001 by Student’s *t*-test. **(H)** Western blot analysis was conducted to explore the protein levels of the Hippo target genes.

To explore the molecular mechanisms through which SNX9 inhibits ADPKD progression, RNA sequencing analysis was conducted, using SNX9-depleted RCTEC and control cells. The fold change (FC) of gene expression was calculated relative to the control cells, and genes with log_2_| FC| > 1 were considered to be differentially expressed (Figure 4A). In total, the expression of 595 genes was disrupted by both shRNAs, with approximately 54.4 and 36.2% of differentially expressed genes (DEGs) overlapping between the two shRNA data sets (Figure 4B and Supplementary Tables S2–S4). The Kyoto Encyclopedia of Genes and Genomes (KEGG) enrichment analysis was then conducted, using genes affected by both shRNAs, which showed similar expression patterns, and the results suggested that the Hippo signaling pathway was enriched most significantly (Figure 4C and Supplementary Table S5). Subsequently, the luciferase reporter assay was performed, and the results suggested that YAP/TEAD4 transcriptional activity was markedly inhibited by SNX9 overexpression (Figure 4D). Meanwhile, we found a boundary significant trend towards increased luciferase activity from control sh-NC to the experimental sh-RNAs groups (*P* = 0.051). Compared with sh-NC group, the YAP/TEAD4 transcriptional activity was significantly promoted by transfection of sh-1 (*P* = 0.045), but not by sh-2 (*P* = 0.229), indicating that SNX9 might repress the YAP/TEAD4 transcriptional activity (Figure 4D). In addition, as determined by immunofluorescence and nuclear fraction assays, SNX9 depletion resulted in the substantial nuclear accumulation of YAP, whereas SNX9 overexpression led to the decreased expression of nuclear YAP (Figures 4E,F). Subsequently, the impacts of SNX9 on the target genes of the Hippo-YAP pathway, including CTGF, CDX2, and CYR61 (Jiao et al., 2014), were also detected. The results showed that SNX9 silencing profoundly increased and SNX9 overexpression reduced the mRNA and protein levels of these target genes (Figures 4G,H). Taken together, these findings suggested that SNX9 activates the Hippo signaling pathway by promoting the cytoplasmic retention of YAP, inhibiting YAP/TEAD4 transcriptional activity and target gene expression.

**FIGURE 5:**
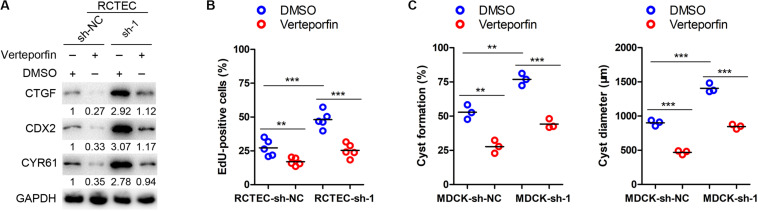
SNX9 inhibits ADPKD progression via the activation of the Hippo signaling pathway. **(A)** SNX9-depleted RCTEC and control cells were treated with the YAP/TEAD4 inhibitor verteporfin (1 μM/L) or DMSO for 24 h. Then, the indicated protein levels were assayed by western blot analysis. **(B)** SNX9-depleted and control RCTEC cells were subjected to verteporfin (1 μM/L) or DMSO treatment for 24 h. Then the indicated cells were subjected to EdU incorporation assay. Data are presented with scatterplots and mean values, *n* = 5. **Indicates *P* < 0.01; ***indicates *P* < 0.001 by Student’s *t*-test. **(C)** SNX9-depleted and control MDCK cells were subjected to verteporfin (1 μM/L) or DMSO treatment for 24 h. Then three-dimensional cultures using collagen matrix, with or without verteporfin (1 μM/L) were used to develop a renal cyst formation model. The percentage of cyst formation and the cyst diameters of the indicated cells were shown. Data are presented with scatterplots and mean values, *n* = 3. **Indicates *P* < 0.01; ***indicates *P* < 0.001 by Student’s *t*-test.

To determine whether the SNX9-induced inhibition of ADPKD development was mediated by the Hippo pathway, the YAP/TEAD4 inhibitor verteporfin (Weiler et al., 2017) was used to treat SNX9-depleted RCTEC and MDCK cells. As shown in Figure 5A, verteporfin treatment reversed the activation of YAP target genes that resulted from SNX9 silencing, further confirming the crucial role of SNX9 in the Hippo-YAP signaling pathway. Furthermore, the EdU incorporation assay suggested that the increased proliferative ability of RCTEC cells that was induced by SNX9 depletion was significantly blocked by verteporfin treatment (Figure 5B). Importantly, the increased renal cyst formation percentage and the diameter enlargement observed in MDCK cells following SNX9 depletion were markedly inhibited by verteporfin treatment (Figure 5C). Taken together, our findings suggested that SNX9 inhibits ADPKD progression via the activation of the Hippo signaling pathway.

### SNX9 Promotes LATS1 Binding to and Phosphorylating YAP to Activate the Hippo Signaling Pathway

**FIGURE 6:**
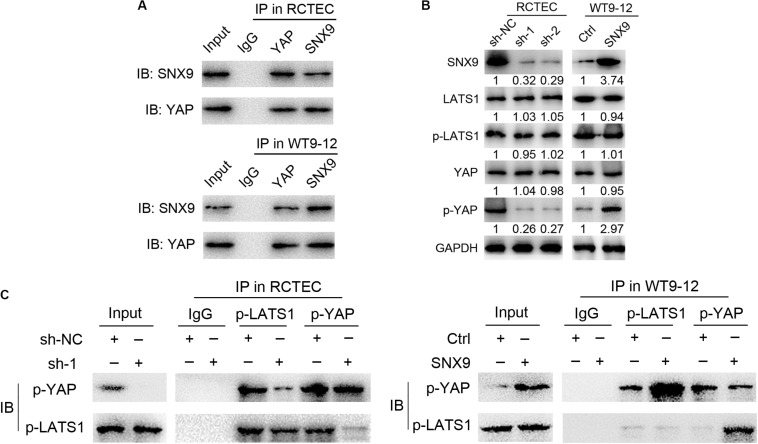
SNX9 promotes LATS1 binding and phosphorylation of YAP, to activate the Hippo signaling pathway. **(A)** Co-IP experiments examining the interaction between SNX9 with YAP in RCTEC and WT9-12 cells. **(B)** Western blot analysis was performed to examine the p-LATS1, p-YAP, total LATS1, and total YAP protein levels of the indicated cells. **(C)** Co-IP experiments examining the interaction between p-YAP and p-LATS1 in the indicated cells, with modified SNX9 expression levels.

To determine how SNX9 inhibits the YAP activity, we evaluated whether SNX9 could interact with YAP. As shown in Figure 6A, the co-immunoprecipitation (Co-IP) assay identified an endogenous interaction between SNX9 and YAP, in both RCTEC and WT9-12 cells. Because YAP activity is tightly regulated by the phosphorylation, mediated by the kinase LATS1 (Yu et al., 2015), we then examined the effect of SNX9 on LATS1, p-LATS1, and YAP expression. Interestingly, the modification of SNX9 expression resulted in no expression changes among these proteins (Figure 6B). In contrast, we found that the p-YAP protein level was markedly increased by SNX9 overexpression in WT9-12 cells and decreased by SNX9 depletion in RCTEC cells (Figure 6B). We then asked whether SNX9 is involved in the LATS1-mediated phosphorylation of YAP. As expected, the binding of p-LATS1 and p-YAP was markedly upregulated by SNX9 overexpression in WT9-12 cells and downregulated by SNX9 depletion in RCTEC cells (Figure 6C). Collectively, these results suggested that SNX9 activates the Hippo pathway by promoting the binding between LATS1 and YAP and the subsequent YAP phosphorylation, mediated by LATS1.

## Discussion

**FIGURE 7:**
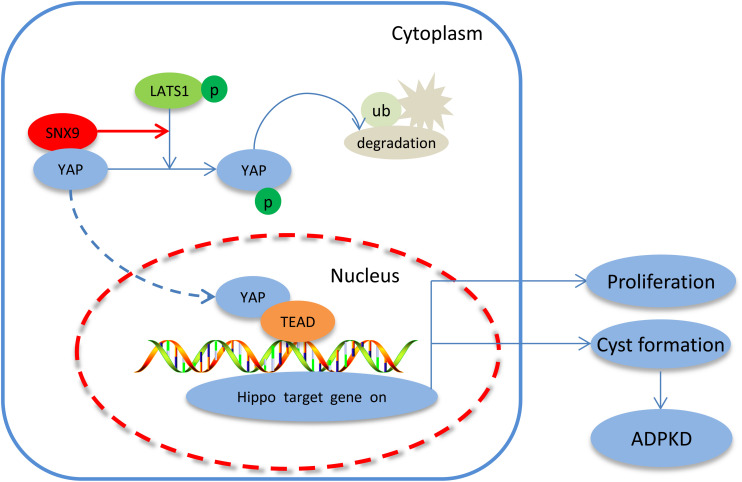
Proposed signaling pathways involved in the SNX9-induced inhibition of ADPKD development. SNX9 interacted directly with YAP, which increased the LATS1-mediated phosphorylation of YAP, leading to the cytoplasmic retention of YAP and the decreased transcriptional activity of the YAP/TEAD4 complex, and, consequently, inhibited the expression of Hippo target genes and ADPKD development.

This study highlighted the expression pattern and inhibitory role of SNX9 during ADPKD development. Furthermore, we demonstrated that SNX9 interacted directly with YAP and promoted LATS1-mediated YAP phosphorylation, resulting in the cytoplasmic retention of YAP and the transcriptional inactivation of the YAP/TEAD4 complex, which, consequently, retards renal cyst development. We have represented this mechanism through a scheme in Figure 7.

Emerging studies have identified SNX9 to be a multifunctional scaffold protein that coordinates membrane and actin dynamics to affect multiple cellular processes (Bendris and Schmid, 2017). Not surprisingly, the deregulation of SNX9 expression contributes to many human diseases, including Lowe syndrome (Nandez et al., 2014), Chlamydia-induced infection (Hansch et al., 2020), chronic inflammation (Ish-Shalom et al., 2016), and cancer (Bendris et al., 2016a, b). As a scaffold, changes in SNX9 expression could result in internal competition for binding partners, disrupting cellular activities, such as cell metastasis and angiogenesis (Bendris et al., 2016a, b; Bendris and Schmid, 2017; Tanigawa and Maekawa, 2019). Of note, recent studies have uncovered the vital role played by SNX9 in the pathogenesis of renal diseases. For example, SNX9 functions as a facilitator of podocin endocytosis in injured podocytes (Sasaki et al., 2017). The interaction between SNX9 and phosphatidylinositol 3,4-bisphosphate [PI(3,4)P2] is essential for lumen formation in MDCK cells (Román-Fernández et al., 2018). In this study, we confirmed that the downregulation of SNX9, in both ADPKD patients and a Pkd1^–/–^ mouse model relative to the levels of SNX9 in controls humans and mice. We also revealed the relatively lower SNX9 expression in WT9-12 cells compared with RCTEC cells. Polycystic kidney disease, a typical tumor-like disease, is characterized by the hyper-proliferation of cyst-lined epithelial cells and the formation of multiple fluid-filled kidney cysts (Ibrahim, 2007). Through a series of functional studies, we revealed the suppressive role of SNX9 in ADPKD cell proliferation, using CCK-8, colony formation, and EdU incorporation assays. Furthermore, we also demonstrated that SNX9 inhibits ADPKD development, by developing an *in vitro* MDCK cyst model. Collectively, our data confirmed the important functions of SNX9 during ADPKD evolution.

Autosomal dominant polycystic kidney disease is a complex disease, in which many genes and signaling pathways are altered, resulting in a heterogeneous molecular profile (Malekshahabi et al., 2019), and this heterogeneity contributes to the clinical management of ADPKD patients (Bergmann et al., 2018). A broad range of signaling pathways have been identified to be involved in ADPKD development, including the cAMP, mammalian target of rapamycin (mTOR), Wnt, and Hippo-YAP signaling pathways (Bergmann et al., 2018). Recently, the involvement of the Hippo pathway during ADPKD development has been increasingly recognized (Ma and Guan, 2018; Muller and Schermer, 2019). For example, the Hippo pathway is inactivated in *PKD1* knockout mouse models and in ADPKD patients, which is associated with the translocation of cytoplasmic YAP into the nucleus (Happe et al., 2011). The RhoA–YAP–c-Myc signaling axis has been demonstrated to promote the development of polycystic kidney disease (Cai et al., 2018). Although the core components of the Hippo-YAP pathway have been intensively studied (Yu et al., 2015), the upstream molecules are more complex and must be further clarified. In this study, we demonstrated that SNX9 promotes the cytoplasmic retention of YAP, retards YAP/TEAD4 transcriptional activity, and downregulates Hippo signaling target gene expression. We further characterized the Hippo pathway as a mediator, involved in the SNX9-induced inhibition of ADPKD development. Thus, our data identified SNX9 to be a novel regulator of the Hippo pathway. These results also underscored the significance of SNX9 in the regulatory network of the Hippo signaling pathway.

Despite the well-established association between SNX9 and ADPKD inhibition, which is mediated by Hippo-YAP signaling, the detailed molecular mechanisms remain poorly understood. The kinase LATS1, which is the most important negative regulator of YAP, inhibits YAP by direct phosphorylation at S127, leading to YAP ubiquitination and cytoplasmic sequestration (Hao et al., 2008). In agreement with those findings, our data showed that a significant increase in the p-YAP level following SNX9 overexpression. Importantly, we also demonstrated that SNX9 promotes the physical interaction between LATS1 and YAP, based on the Co-IP analysis. Thus, we propose that SNX9 may inactivate YAP by facilitating the binding and phosphorylation of YAP by LATS1.

In summary, this study examined the expression pattern, biological function, and molecular mechanisms of SNX9 during ADPKD evolution. We demonstrated that SNX9 activates the Hippo signaling pathway, which attenuated cell proliferation and renal cyst formation and enlargement. These findings provide novel insights into the pathogenesis and therapeutic development of ADPKD. Our study may also deepen the understanding of the molecular and functional network associated with the Hippo-YAP signaling pathway and contribute to mechanistic clues that can drive novel therapeutic approaches for ADPKD and other related kidney diseases.

## Data Availability Statement

All datasets presented in this study are included in the article/Supplementary Material.

## Ethics Statement

The animal study was reviewed and approved by Shanghai Changzheng Hospital Biomedical Research Ethics Committee.

## Author Contributions

S-QY and A-WS designed the study. A-WS and P-RY wrote the manuscript. A-WS, L-LF, and LL done the experiment. D-XS, W-TW, and Y-HW analyzed the data. All authors read and approved the final manuscript.

## Conflict of Interest

The authors declare that the research was conducted in the absence of any commercial or financial relationships that could be construed as a potential conflict of interest.

## Supplementary Material

TABLE S1 Antibodies used in this study.

TABLE S2 RNA sequencing analysis of the gene expression profile, following sh-1and sh-NC treatment for SNX9.

TABLE S3 RNA sequencing analysis of the gene expression profile, following sh-2 and sh-NC treatment for SNX9.

TABLE S4 RNA sequencing analysis of the gene expression profile, following both sh-1 and sh-2 treatment for SNX9.

TABLE S5 KEGG analysis of RNA sequencing data from SNX9-depleted RCTEC and control cells, using genes affected by both shRNAs (sh-1 and sh-2) that showed similar expression patterns.
