# Supplementary material for: SNX9 Inhibits Cell Proliferation and Cyst Development in Autosomal Dominant Polycystic Kidney Disease via Activation of the Hippo-YAP Signaling Pathway
Source: Front Cell Dev Biol. 2020 Aug 21;8:811. doi: 10.3389/fcell.2020.00811 (PMC7472854; doi:10.3389/fcell.2020.00811)
Supplement: TABLE S1 — Antibodies used in this study. [file Table_1.DOCX]

**Supplementary material**

**Sup.Tab.1** Antibodies used in this study.

| **Protein** | **Cat no.** | **Company** | **Source** | **KD** | **Dilution factors** |
| --- | --- | --- | --- | --- | --- |
| Lamin B1 | 12987-1-AP | Protein tech | Rabbit | 66 | WB: 1:2000 |
| GAPDH | 60004-1-Ig | Protein tech | Mouse | 36 | WB: 1:5000 |
| SNX9 | ab181856 | Abcam | Rabbit | 78 | WB: 1:1000; IHC: 1:200; IP: 1:50 |
| YAP | 14074 | CST | Rabbit | 65 | WB: 1:1000; IF: 1:100; IP: 1:50 |
| p-YAP(Ser127) | 13008 | CST | Rabbit | 65 | WB: 1:1000; IP: 1:200 |
| LATS1 | 3477 | CST | Rabbit | 140 | WB: 1:1000 |
| p-LATS1( Thr1079) | D151300 | Sangon | Rabbit | 140 | WB: 1:1000; IP: 1:50 |
| CTGF | 23936-1-AP | Protein tech | Rabbit | 38 | WB: 1:1000 |
| CDX2 | 60243-1-Ig | Protein tech | Mouse | 34 | WB: 1:1000 |
| CYR61 | 14479 | CST | Rabbit | 41 | WB: 1:1000 |

**Sup.Tab.2** RNA-seq analysis of the gene expression profile affected by sh-1and sh-NC treatment for SNX9.

| Gene id | MeanTPM (sh-1) | MeanTPM (sh-NC) | log2FoldChange | pValue | qValue |
| --- | --- | --- | --- | --- | --- |
| ENSG00000137693 | 54.06374 | 1288.154 | -4.5745 | 0.000000000000 | 0.000000000000 |
| ENSG00000210082 | 6094.444 | 2417.161 | 1.334181 | 0.000000000000 | 0.000000000000 |
| ENSG00000142871 | 56.62669 | 1064.849 | -4.23302 | 0.000000000000 | 0.000000000000 |
| ENSG00000021826 | 2258.516 | 1023.183 | 1.142311 | 0.000000000000 | 0.000000000000 |
| ENSG00000196924 | 585.1019 | 1601.531 | -1.45269 | 0.000000000000 | 0.000000000000 |
| ENSG00000075624 | 2713.167 | 6123.888 | -1.17447 | 0.000000000000 | 0.000000000000 |
| ENSG00000070404 | 345.0061 | 1121.674 | -1.70096 | 0.000000000000 | 0.000000000000 |
| ENSG00000100345 | 207.3561 | 496.3726 | -1.25931 | 0.000000000000 | 0.000000000000 |
| ENSG00000142627 | 71.29536 | 340.3448 | -2.25512 | 0.000000000000 | 0.000000000000 |
| ENSG00000161011 | 216.6722 | 753.3383 | -1.79778 | 0.000000000000 | 0.000000000000 |
| ENSG00000142949 | 151.7501 | 399.0557 | -1.39489 | 0.000000000000 | 0.000000000000 |
| ENSG00000198763 | 2682.055 | 1308.574 | 1.035343 | 0.000000000000 | 0.000000000000 |
| ENSG00000163430 | 35.56473 | 178.1041 | -2.3242 | 0.000000000000 | 0.000000000000 |
| ENSG00000184009 | 1168.945 | 2491.544 | -1.09183 | 0.000000000000 | 0.000000000000 |
| ENSG00000159176 | 86.09644 | 439.1967 | -2.35084 | 0.000000000000 | 0.000000000000 |
| ENSG00000111057 | 383.6769 | 987.8673 | -1.36443 | 0.000000000000 | 0.000000000000 |
| ENSG00000187608 | 772.2281 | 1951.132 | -1.33721 | 0.000000000000 | 0.000000000000 |
| ENSG00000271503 | 192.8952 | 650.8859 | -1.75459 | 0.000000000000 | 0.000000000000 |
| ENSG00000125148 | 243.3604 | 941.0363 | -1.95116 | 0.000000000000 | 0.000000000000 |
| ENSG00000120129 | 277.4541 | 669.8516 | -1.27159 | 0.000000000000 | 0.000000000000 |
| ENSG00000165389 | 183.9585 | 40.41221 | 2.186517 | 0.000000000000 | 0.000000000000 |
| ENSG00000198899 | 2056.984 | 1003.091 | 1.036078 | 0.000000000000 | 0.000000000000 |
| ENSG00000142910 | 35.23274 | 220.9791 | -2.64892 | 0.000000000000 | 0.000000000000 |
| ENSG00000148773 | 51.25858 | 15.69159 | 1.707802 | 0.000000000000 | 0.000000000000 |
| ENSG00000158104 | 750.2538 | 323.3627 | 1.214225 | 0.000000000000 | 0.000000000000 |
| ENSG00000114019 | 33.14326 | 186.7007 | -2.49394 | 0.000000000000 | 0.000000000000 |
| ENSG00000113739 | 19.48043 | 94.89493 | -2.28431 | 0.000000000000 | 0.000000000000 |
| ENSG00000006327 | 69.90039 | 342.2341 | -2.29161 | 0.000000000000 | 0.000000000000 |
| ENSG00000136244 | 14.81992 | 205.0733 | -3.79053 | 0.000000000000 | 0.000000000000 |
| ENSG00000116285 | 73.78388 | 229.5957 | -1.63772 | 0.000000000000 | 0.000000000000 |
| ENSG00000162734 | 38.94631 | 151.1154 | -1.95609 | 0.000000000000 | 0.000000000000 |
| ENSG00000145901 | 91.96287 | 258.675 | -1.49202 | 0.000000000000 | 0.000000000000 |
| ENSG00000109079 | 42.10978 | 135.3004 | -1.68394 | 0.000000000000 | 0.000000000000 |
| ENSG00000128272 | 283.9095 | 627.3217 | -1.14377 | 0.000000000000 | 0.000000000000 |
| ENSG00000137331 | 169.3504 | 469.2563 | -1.47037 | 0.000000000000 | 0.000000000000 |
| ENSG00000189060 | 176.161 | 394.5346 | -1.16326 | 0.000000000000 | 0.000000000000 |
| ENSG00000138166 | 14.39932 | 103.0572 | -2.83937 | 0.000000000000 | 0.000000000000 |
| ENSG00000167767 | 74.2631 | 183.8642 | -1.30792 | 0.000000000000 | 0.000000000000 |
| ENSG00000111859 | 8.912534 | 59.88854 | -2.74837 | 0.000000000000 | 0.000000000000 |
| ENSG00000169180 | 83.90013 | 185.5785 | -1.14528 | 0.000000000000 | 0.000000000000 |
| ENSG00000077150 | 121.6136 | 283.4767 | -1.22093 | 0.000000000000 | 0.000000000000 |
| ENSG00000139112 | 45.25466 | 178.1454 | -1.97692 | 0.000000000000 | 0.000000000000 |
| ENSG00000258017 | 472.8357 | 1019.874 | -1.10898 | 0.000000000000 | 0.000000000000 |
| ENSG00000143570 | 267.8422 | 93.35365 | 1.520605 | 0.000000000000 | 0.000000000000 |
| ENSG00000113083 | 132.9537 | 270.7804 | -1.0262 | 0.000000000000 | 0.000000000000 |
| ENSG00000161638 | 116.8113 | 237.4228 | -1.02328 | 0.000000000000 | 0.000000000000 |
| ENSG00000103187 | 130.9991 | 346.7943 | -1.40452 | 0.000000000000 | 0.000000000000 |
| ENSG00000072310 | 290.8403 | 121.9969 | 1.253382 | 0.000000000000 | 0.000000000000 |
| ENSG00000237412 | 379.3038 | 189.0722 | 1.004416 | 0.000000000000 | 0.000000000000 |
| ENSG00000105281 | 132.6085 | 397.7608 | -1.58473 | 0.000000000000 | 0.000000000000 |
| ENSG00000169604 | 63.17478 | 151.2794 | -1.2598 | 0.000000000000 | 0.000000000000 |
| ENSG00000163347 | 10.22227 | 64.4744 | -2.65701 | 0.000000000000 | 0.000000000000 |
| ENSG00000100092 | 6.60346 | 51.76777 | -2.97076 | 0.000000000000 | 0.000000000000 |
| ENSG00000087074 | 31.89844 | 132.249 | -2.0517 | 0.000000000000 | 0.000000000000 |
| ENSG00000137801 | 3.473638 | 29.25703 | -3.07426 | 0.000000000000 | 0.000000000000 |
| ENSG00000023445 | 21.69048 | 90.94766 | -2.06797 | 0.000000000000 | 0.000000000000 |
| ENSG00000115884 | 10.12663 | 74.41278 | -2.8774 | 0.000000000000 | 0.000000000000 |
| ENSG00000117525 | 24.49643 | 142.2828 | -2.53812 | 0.000000000000 | 0.000000000000 |
| ENSG00000106366 | 21.28414 | 89.70608 | -2.07543 | 0.000000000000 | 0.000000000000 |
| ENSG00000110092 | 11.94222 | 61.34182 | -2.3608 | 0.000000000000 | 0.000000000000 |
| ENSG00000101150 | 89.9199 | 246.3996 | -1.45429 | 0.000000000000 | 0.000000000000 |
| ENSG00000167772 | 71.97578 | 227.6768 | -1.6614 | 0.000000000000 | 0.000000000000 |
| ENSG00000174282 | 48.69279 | 13.68937 | 1.830653 | 0.000000000000 | 0.000000000000 |
| ENSG00000188153 | 93.55817 | 43.47642 | 1.105631 | 0.000000000000 | 0.000000000000 |
| ENSG00000124201 | 12.83116 | 39.89739 | -1.63664 | 0.000000000000 | 0.000000000000 |
| ENSG00000107262 | 267.7868 | 115.0594 | 1.218705 | 0.000000000000 | 0.000000000000 |
| ENSG00000138772 | 41.58538 | 155.0399 | -1.89849 | 0.000000000000 | 0.000000000000 |
| ENSG00000129473 | 8.47987 | 35.89 | -2.08147 | 0.000000000000 | 0.000000000000 |
| ENSG00000268621 | 115.659 | 524.5468 | -2.18119 | 0.000000000000 | 0.000000000000 |
| ENSG00000178252 | 86.47184 | 39.88927 | 1.11623 | 0.000000000000 | 0.000000000000 |
| ENSG00000114857 | 29.46929 | 12.89443 | 1.192464 | 0.000000000000 | 0.000000000000 |
| ENSG00000135919 | 23.30874 | 90.4394 | -1.95608 | 0.000000000000 | 0.000000000000 |
| ENSG00000125743 | 440.8645 | 180.5744 | 1.287742 | 0.000000000000 | 0.000000000000 |
| ENSG00000148841 | 32.61293 | 94.09417 | -1.52866 | 0.000000000000 | 0.000000000000 |
| ENSG00000225630 | 397.3091 | 175.0973 | 1.182105 | 0.000000000000 | 0.000000000000 |
| ENSG00000120875 | 2.525687 | 23.06345 | -3.19086 | 0.000000000000 | 0.000000000000 |
| ENSG00000214049 | 6.58123 | 59.95556 | -3.18746 | 0.000000000000 | 0.000000000000 |
| ENSG00000139289 | 1.813883 | 19.51024 | -3.42708 | 0.000000000000 | 0.000000000000 |
| ENSG00000132470 | 35.98082 | 99.32121 | -1.46487 | 0.000000000000 | 0.000000000000 |
| ENSG00000101654 | 9.070107 | 29.78619 | -1.71545 | 0.000000000000 | 0.000000000000 |
| ENSG00000241360 | 76.70679 | 15.41179 | 2.31532 | 0.000000000000 | 0.000000000000 |
| ENSG00000135245 | 57.36197 | 188.3824 | -1.7155 | 0.000000000000 | 0.000000000000 |
| ENSG00000101255 | 55.05401 | 142.0244 | -1.36722 | 0.000000000000 | 0.000000000000 |
| ENSG00000198959 | 16.49186 | 56.56125 | -1.77806 | 0.000000000000 | 0.000000000000 |
| ENSG00000171161 | 42.58164 | 10.58061 | 2.008809 | 0.000000000000 | 0.000000000000 |
| ENSG00000137936 | 23.89459 | 79.52197 | -1.73467 | 0.000000000000 | 0.000000000000 |
| ENSG00000120708 | 56.63454 | 137.9248 | -1.28413 | 0.000000000000 | 0.000000000000 |
| ENSG00000204291 | 53.88449 | 21.4538 | 1.328637 | 0.000000000000 | 0.000000000000 |
| ENSG00000172379 | 21.69067 | 6.222623 | 1.80148 | 0.000000000000 | 0.000000000000 |
| ENSG00000090339 | 18.5767 | 68.24806 | -1.87729 | 0.000000000000 | 0.000000000000 |
| ENSG00000275216 | 38.49088 | 91.54212 | -1.24992 | 0.000000000000 | 0.000000000000 |
| ENSG00000116574 | 42.28198 | 11.59871 | 1.866079 | 0.000000000000 | 0.000000000000 |
| ENSG00000173391 | 10.69802 | 62.59703 | -2.54875 | 0.000000000000 | 0.000000000000 |
| ENSG00000173559 | 54.74773 | 22.29068 | 1.296358 | 0.000000000000 | 0.000000000000 |
| ENSG00000210135 | 2968.349 | 944.7426 | 1.651668 | 0.000000000000 | 0.000000000000 |
| ENSG00000198492 | 63.5898 | 12.32914 | 2.366723 | 0.000000000000 | 0.000000000000 |
| ENSG00000057294 | 13.98466 | 45.95919 | -1.71651 | 0.000000000000 | 0.000000000000 |
| ENSG00000270136 | 46.53352 | 0.696356 | 6.062301 | 0.000000000000 | 0.000000000000 |
| ENSG00000130513 | 74.84061 | 217.8267 | -1.54129 | 0.000000000000 | 0.000000000000 |
| ENSG00000128510 | 3.110029 | 37.07925 | -3.57561 | 0.000000000000 | 0.000000000000 |
| ENSG00000100342 | 5.880852 | 37.09788 | -2.65724 | 0.000000000000 | 0.000000000000 |
| ENSG00000210127 | 1991.51 | 406.3652 | 2.293014 | 0.000000000000 | 0.000000000000 |
| ENSG00000131503 | 30.46945 | 66.98547 | -1.13648 | 0.000000000000 | 0.000000000000 |
| ENSG00000284292 | 110.1805 | 42.75087 | 1.365843 | 0.000000000000 | 0.000000000000 |
| ENSG00000170345 | 143.682 | 31.09114 | 2.208304 | 0.000000000000 | 0.000000000000 |
| ENSG00000115365 | 79.52888 | 39.27602 | 1.01783 | 0.000000000000 | 0.000000000000 |
| ENSG00000108846 | 85.23998 | 182.5772 | -1.0989 | 0.000000000000 | 0.000000000000 |
| ENSG00000079257 | 129.315 | 30.20948 | 2.097816 | 0.000000000000 | 0.000000000000 |
| ENSG00000106105 | 164.9189 | 358.6987 | -1.12102 | 0.000000000000 | 0.000000000000 |
| ENSG00000147689 | 98.72232 | 43.82692 | 1.171559 | 0.000000000000 | 0.000000000000 |
| ENSG00000179862 | 91.52481 | 226.4734 | -1.30711 | 0.000000000000 | 0.000000000000 |
| ENSG00000187634 | 104.9201 | 27.01196 | 1.957621 | 0.000000000000 | 0.000000000000 |
| ENSG00000158050 | 81.29019 | 16.3904 | 2.31023 | 0.000000000000 | 0.000000000000 |
| ENSG00000177885 | 29.99451 | 79.47469 | -1.4058 | 0.000000000000 | 0.000000000000 |
| ENSG00000173852 | 27.18322 | 8.630136 | 1.655261 | 0.000000000000 | 0.000000000000 |
| ENSG00000168994 | 21.3035 | 81.28843 | -1.93196 | 0.000000000000 | 0.000000000000 |
| ENSG00000131094 | 83.09348 | 209.3566 | -1.33316 | 0.000000000000 | 0.000000000000 |
| ENSG00000214510 | 187.2939 | 63.07437 | 1.570178 | 0.000000000000 | 0.000000000000 |
| ENSG00000187193 | 69.19692 | 299.5215 | -2.11388 | 0.000000000000 | 0.000000000000 |
| ENSG00000141232 | 193.7881 | 95.12643 | 1.026562 | 0.000000000000 | 0.000000000000 |
| ENSG00000063322 | 9.543899 | 44.09955 | -2.20811 | 0.000000000000 | 0.000000000000 |
| ENSG00000228253 | 1015.98 | 385.0959 | 1.399582 | 0.000000000000 | 0.000000000000 |
| ENSG00000042445 | 96.02091 | 45.50444 | 1.077341 | 0.000000000000 | 0.000000000000 |
| ENSG00000104140 | 155.923 | 68.72408 | 1.181946 | 0.000000000000 | 0.000000000000 |
| ENSG00000185567 | 13.59762 | 6.78913 | 1.002055 | 0.000000000000 | 0.000000000000 |
| ENSG00000198796 | 6.104886 | 21.46776 | -1.81414 | 0.000000000000 | 0.000000000000 |
| ENSG00000156467 | 92.2686 | 37.37625 | 1.303718 | 0.000000000000 | 0.000000000000 |
| ENSG00000135046 | 165.7178 | 407.2406 | -1.29715 | 0.000000000000 | 0.000000000000 |
| ENSG00000128283 | 75.47955 | 162.2724 | -1.10426 | 0.000000000000 | 0.000000000000 |
| ENSG00000162073 | 74.1468 | 21.26003 | 1.802241 | 0.000000000000 | 0.000000000000 |
| ENSG00000143322 | 17.52598 | 40.16933 | -1.1966 | 0.000000000000 | 0.000000000000 |
| ENSG00000108219 | 7.543307 | 28.7604 | -1.93081 | 0.000000000000 | 0.000000000000 |
| ENSG00000171552 | 32.63746 | 85.23518 | -1.38492 | 0.000000000000 | 0.000000000000 |
| ENSG00000163395 | 1.01826 | 9.658869 | -3.24575 | 0.000000000000 | 0.000000000000 |
| ENSG00000054793 | 38.67486 | 19.19312 | 1.010807 | 0.000000000000 | 0.000000000000 |
| ENSG00000197172 | 31.35389 | 0.198212 | 7.305456 | 0.000000000000 | 0.000000000000 |
| ENSG00000025800 | 30.44066 | 12.7371 | 1.256963 | 0.000000000000 | 0.000000000000 |
| ENSG00000224389 | 39.85053 | 13.85739 | 1.523944 | 0.000000000000 | 0.000000000000 |
| ENSG00000156510 | 14.63301 | 44.79305 | -1.61405 | 0.000000000000 | 0.000000000000 |
| ENSG00000089486 | 46.3597 | 142.3642 | -1.61864 | 0.000000000000 | 0.000000000000 |
| ENSG00000134531 | 11.70248 | 73.00797 | -2.64124 | 0.000000000000 | 0.000000000000 |
| ENSG00000143653 | 99.37812 | 45.93105 | 1.113459 | 0.000000000000 | 0.000000000000 |
| ENSG00000112773 | 44.92656 | 21.16555 | 1.08585 | 0.000000000000 | 0.000000000000 |
| ENSG00000148677 | 0.112719 | 28.95184 | -8.00478 | 0.000000000000 | 0.000000000000 |
| ENSG00000119922 | 23.17565 | 63.28692 | -1.4493 | 0.000000000000 | 0.000000000000 |
| ENSG00000187908 | 8.037423 | 23.17676 | -1.52787 | 0.000000000000 | 0.000000000000 |
| ENSG00000132906 | 171.8865 | 82.68601 | 1.055741 | 0.000000000000 | 0.000000000000 |
| ENSG00000075142 | 289.1088 | 137.1893 | 1.075445 | 0.000000000000 | 0.000000000000 |
| ENSG00000168461 | 51.34853 | 24.46822 | 1.069414 | 0.000000000000 | 0.000000000000 |
| ENSG00000168528 | 77.43636 | 168.911 | -1.12518 | 0.000000000000 | 0.000000000000 |
| ENSG00000159228 | 117.6141 | 241.036 | -1.03519 | 0.000000000000 | 0.000000000000 |
| ENSG00000101216 | 11.97374 | 36.03139 | -1.58938 | 0.000000000000 | 0.000000000000 |
| ENSG00000280987 | 1.322959 | 19.83568 | -3.90626 | 0.000000000000 | 0.000000000000 |
| ENSG00000118495 | 38.12812 | 119.2903 | -1.64555 | 0.000000000000 | 0.000000000000 |
| ENSG00000137710 | 64.25623 | 25.45235 | 1.336038 | 0.000000000000 | 0.000000000000 |
| ENSG00000128342 | 18.04887 | 47.33786 | -1.39109 | 0.000000000000 | 0.000000000000 |
| ENSG00000166986 | 73.54325 | 165.3721 | -1.16905 | 0.000000000000 | 0.000000000000 |
| ENSG00000177606 | 22.96075 | 56.57069 | -1.30089 | 0.000000000000 | 0.000000000000 |
| ENSG00000139182 | 93.24456 | 38.67713 | 1.269539 | 0.000000000000 | 0.000000000000 |
| ENSG00000163814 | 12.90573 | 34.01145 | -1.39801 | 0.000000000000 | 0.000000000000 |
| ENSG00000107738 | 9.312737 | 29.49046 | -1.66297 | 0.000000000000 | 0.000000000000 |
| ENSG00000167657 | 92.44242 | 185.4782 | -1.00462 | 0.000000000000 | 0.000000000000 |
| ENSG00000105854 | 173.7657 | 83.8883 | 1.050602 | 0.000000000000 | 0.000000000000 |
| ENSG00000272325 | 14.72755 | 5.468764 | 1.429231 | 0.000000000000 | 0.000000000000 |
| ENSG00000277957 | 37.20763 | 12.79078 | 1.540494 | 0.000000000000 | 0.000000000000 |
| ENSG00000132819 | 8.387429 | 33.54177 | -1.99966 | 0.000000000000 | 0.000000000000 |
| ENSG00000125257 | 35.37663 | 16.39387 | 1.10964 | 0.000000000000 | 0.000000000000 |
| ENSG00000109572 | 34.58646 | 11.97586 | 1.530078 | 0.000000000000 | 0.000000000000 |
| ENSG00000130766 | 8.644769 | 31.88035 | -1.88277 | 0.000000000000 | 0.000000000000 |
| ENSG00000145495 | 150.331 | 69.01823 | 1.123093 | 0.000000000000 | 0.000000000000 |
| ENSG00000213024 | 38.77651 | 80.7963 | -1.05911 | 0.000000000000 | 0.000000000000 |
| ENSG00000101544 | 18.17913 | 6.5267 | 1.477857 | 0.000000000000 | 0.000000000000 |
| ENSG00000278189 | 367.9951 | 43.87384 | 3.068254 | 0.000000000000 | 0.000000000000 |
| ENSG00000119471 | 62.39102 | 27.58761 | 1.177318 | 0.000000000000 | 0.000000000000 |
| ENSG00000034152 | 35.72795 | 126.3015 | -1.82175 | 0.000000000000 | 0.000000000000 |
| ENSG00000130202 | 70.60207 | 144.9592 | -1.03786 | 0.000000000000 | 0.000000000000 |
| ENSG00000074527 | 9.608791 | 35.01678 | -1.86562 | 0.000000000000 | 0.000000000000 |
| ENSG00000101335 | 39.46577 | 101.2765 | -1.35963 | 0.000000000000 | 0.000000000000 |
| ENSG00000143416 | 119.6304 | 58.898 | 1.022294 | 0.000000000000 | 0.000000000000 |
| ENSG00000167106 | 34.65952 | 14.1629 | 1.291135 | 0.000000000000 | 0.000000000000 |
| ENSG00000175832 | 8.858068 | 45.8654 | -2.37234 | 0.000000000000 | 0.000000000000 |
| ENSG00000284526 | 7.657531 | 0.032991 | 7.858663 | 0.000000000000 | 0.000000000000 |
| ENSG00000171401 | 11.01056 | 52.83612 | -2.26264 | 0.000000000000 | 0.000000000000 |
| ENSG00000223764 | 17.45554 | 3.308716 | 2.399342 | 0.000000000000 | 0.000000000000 |
| ENSG00000112146 | 60.96586 | 28.5725 | 1.093375 | 0.000000000000 | 0.000000000000 |
| ENSG00000048707 | 15.06659 | 6.22477 | 1.27526 | 0.000000000000 | 0.000000000000 |
| ENSG00000186480 | 92.94971 | 43.29433 | 1.102272 | 0.000000000000 | 0.000000000000 |
| ENSG00000169359 | 18.52364 | 4.443292 | 2.059667 | 0.000000000000 | 0.000000000000 |
| ENSG00000243207 | 4.659064 | 27.82485 | -2.57826 | 0.000000000000 | 0.000000000000 |
| ENSG00000149798 | 17.03354 | 55.73309 | -1.71016 | 0.000000000000 | 0.000000000000 |
| ENSG00000168398 | 18.94923 | 4.901495 | 1.950845 | 0.000000000000 | 0.000000000000 |
| ENSG00000114779 | 90.28227 | 38.85341 | 1.216402 | 0.000000000000 | 0.000000000000 |
| ENSG00000156273 | 18.04753 | 43.05792 | -1.25448 | 0.000000000000 | 0.000000000000 |
| ENSG00000244731 | 29.57844 | 12.86394 | 1.201214 | 0.000000000000 | 0.000000000000 |
| ENSG00000244405 | 8.926489 | 30.70835 | -1.78247 | 0.000000000000 | 0.000000000000 |
| ENSG00000078804 | 9.184402 | 0.504089 | 4.187435 | 0.000000000000 | 0.000000000000 |
| ENSG00000102034 | 28.64705 | 9.087803 | 1.656383 | 0.000000000000 | 0.000000000000 |
| ENSG00000116717 | 12.19781 | 59.26122 | -2.28047 | 0.000000000000 | 0.000000000000 |
| ENSG00000140612 | 124.843 | 51.27478 | 1.283794 | 0.000000000000 | 0.000000000000 |
| ENSG00000131263 | 20.58422 | 9.901253 | 1.055855 | 0.000000000000 | 0.000000000000 |
| ENSG00000128165 | 2.108003 | 14.29593 | -2.76166 | 0.000000000000 | 0.000000000000 |
| ENSG00000179195 | 42.16477 | 19.34788 | 1.123863 | 0.000000000000 | 0.000000000000 |
| ENSG00000107338 | 9.474549 | 34.03703 | -1.84498 | 0.000000000000 | 0.000000000000 |
| ENSG00000100234 | 25.24033 | 51.49959 | -1.02883 | 0.000000000000 | 0.000000000000 |
| ENSG00000133318 | 170.7026 | 76.98557 | 1.148825 | 0.000000000000 | 0.000000000000 |
| ENSG00000117395 | 118.1386 | 237.7915 | -1.00922 | 0.000000000000 | 0.000000000000 |
| ENSG00000123485 | 15.16406 | 40.13832 | -1.40432 | 0.000000000000 | 0.000000000000 |
| ENSG00000175040 | 42.41334 | 20.9754 | 1.01582 | 0.000000000000 | 0.000000000000 |
| ENSG00000280071 | 78.76458 | 24.44698 | 1.687891 | 0.000000000000 | 0.000000000000 |
| ENSG00000178922 | 43.6506 | 119.5984 | -1.45412 | 0.000000000000 | 0.000000000000 |
| ENSG00000008513 | 10.54592 | 28.29582 | -1.4239 | 0.000000000000 | 0.000000000000 |
| ENSG00000134215 | 50.64249 | 17.61734 | 1.523352 | 0.000000000000 | 0.000000000000 |
| ENSG00000115963 | 16.5572 | 85.41354 | -2.36701 | 0.000000000000 | 0.000000000000 |
| ENSG00000052344 | 38.99276 | 93.37869 | -1.25989 | 0.000000000000 | 0.000000000000 |
| ENSG00000148411 | 13.37043 | 4.267676 | 1.647523 | 0.000000000000 | 0.000000000000 |
| ENSG00000140950 | 37.24938 | 79.64086 | -1.09629 | 0.000000000000 | 0.000000000000 |
| ENSG00000135269 | 49.30535 | 105.8917 | -1.10277 | 0.000000000000 | 0.000000000000 |
| ENSG00000092295 | 4.819242 | 24.61626 | -2.35273 | 0.000000000000 | 0.000000000000 |
| ENSG00000001617 | 24.7808 | 54.6382 | -1.14069 | 0.000000000000 | 0.000000000000 |
| ENSG00000269968 | 340.8014 | 148.4596 | 1.19886 | 0.000000000000 | 0.000000000000 |
| ENSG00000133466 | 34.32223 | 74.9159 | -1.12613 | 0.000000000000 | 0.000000000000 |
| ENSG00000110619 | 27.12905 | 69.9801 | -1.36711 | 0.000000000000 | 0.000000000000 |
| ENSG00000115464 | 33.15097 | 11.22018 | 1.562955 | 0.000000000000 | 0.000000000000 |
| ENSG00000127184 | 99.32891 | 266.0641 | -1.42149 | 0.000000000000 | 0.000000000000 |
| ENSG00000162413 | 31.90739 | 14.34845 | 1.152996 | 0.000000000000 | 0.000000000000 |
| ENSG00000102100 | 36.69828 | 90.2485 | -1.29819 | 0.000000000000 | 0.000000000000 |
| ENSG00000105141 | 40.30068 | 16.54831 | 1.28412 | 0.000000000000 | 0.000000000000 |
| ENSG00000153162 | 35.38839 | 73.72942 | -1.05896 | 0.000000000000 | 0.000000000000 |
| ENSG00000115758 | 48.78614 | 107.1528 | -1.13513 | 0.000000000000 | 0.000000000000 |
| ENSG00000262160 | 9.80634 | 0.23642 | 5.374291 | 0.000000000000 | 0.000000000000 |
| ENSG00000135318 | 3.031011 | 17.59933 | -2.53765 | 0.000000000000 | 0.000000000000 |
| ENSG00000074590 | 2.595947 | 10.90112 | -2.07014 | 0.000000000000 | 0.000000000000 |
| ENSG00000123444 | 28.1285 | 9.425426 | 1.577403 | 0.000000000000 | 0.000000000000 |
| ENSG00000132613 | 56.47854 | 114.5606 | -1.02034 | 0.000000000000 | 0.000000000000 |
| ENSG00000278540 | 44.75671 | 20.05101 | 1.158429 | 0.000000000000 | 0.000000000000 |
| ENSG00000257341 | 187.6531 | 79.29115 | 1.242837 | 0.000000000000 | 0.000000000000 |
| ENSG00000097021 | 59.31126 | 127.3666 | -1.10261 | 0.000000000000 | 0.000000000000 |
| ENSG00000213859 | 48.46461 | 97.06745 | -1.00206 | 0.000000000000 | 0.000000000000 |
| ENSG00000173221 | 65.03477 | 150.186 | -1.20747 | 0.000000000000 | 0.000000000000 |
| ENSG00000179304 | 87.30881 | 38.43723 | 1.183623 | 0.000000000000 | 0.000000000000 |
| ENSG00000198406 | 103.7862 | 34.56565 | 1.586204 | 0.000000000000 | 0.000000000000 |
| ENSG00000181026 | 29.25505 | 58.80644 | -1.00729 | 0.000000000000 | 0.000000000000 |
| ENSG00000105974 | 37.84763 | 81.54184 | -1.10734 | 0.000000000000 | 0.000000000000 |
| ENSG00000060749 | 10.21609 | 3.780136 | 1.434333 | 0.000000000000 | 0.000000000000 |
| ENSG00000170298 | 5.633287 | 40.41373 | -2.8428 | 0.000000000000 | 0.000000000000 |
| ENSG00000179583 | 7.175155 | 1.005348 | 2.835315 | 0.000000000000 | 0.000000000000 |
| ENSG00000167774 | 4.206734 | 60.14008 | -3.83755 | 0.000000000000 | 0.000000000000 |
| ENSG00000181666 | 25.59292 | 61.09491 | -1.25531 | 0.000000000000 | 0.000000000000 |
| ENSG00000220842 | 144.6617 | 41.38263 | 1.805586 | 0.000000000000 | 0.000000000000 |
| ENSG00000165887 | 1.684479 | 23.67719 | -3.81312 | 0.000000000000 | 0.000000000000 |
| ENSG00000070087 | 37.0058 | 118.2781 | -1.67636 | 0.000000000000 | 0.000000000000 |
| ENSG00000258947 | 24.24763 | 6.109335 | 1.988756 | 0.000000000000 | 0.000000000000 |
| ENSG00000066279 | 9.942974 | 3.994333 | 1.315723 | 0.000000000000 | 0.000000000000 |
| ENSG00000162783 | 16.54786 | 36.70538 | -1.14935 | 0.000000000000 | 0.000000000000 |
| ENSG00000057252 | 18.99626 | 8.702838 | 1.126157 | 0.000000000000 | 0.000000000000 |
| ENSG00000122035 | 65.29326 | 17.13928 | 1.929628 | 0.000000000000 | 0.000000000000 |
| ENSG00000176046 | 28.18925 | 73.80618 | -1.3886 | 0.000000000000 | 0.000000000000 |
| ENSG00000251537 | 0.119331 | 5.05874 | -5.40574 | 0.000000000000 | 0.000000000000 |
| ENSG00000210144 | 2025.982 | 935.6816 | 1.114532 | 0.000000000000 | 0.000000000000 |
| ENSG00000113369 | 31.67639 | 15.02256 | 1.076277 | 0.000000000000 | 0.000000000000 |
| ENSG00000196923 | 67.12927 | 138.858 | -1.0486 | 0.000000000000 | 0.000000000000 |
| ENSG00000171867 | 31.63415 | 69.76747 | -1.14107 | 0.000000000000 | 0.000000000000 |
| ENSG00000149485 | 118.396 | 51.26579 | 1.207552 | 0.000000000000 | 0.000000000000 |
| ENSG00000148926 | 108.7323 | 220.381 | -1.01922 | 0.000000000000 | 0.000000000000 |
| ENSG00000184916 | 8.857919 | 24.26113 | -1.45361 | 0.000000000000 | 0.000000000000 |
| ENSG00000171421 | 85.13149 | 203.9693 | -1.26059 | 0.000000000000 | 0.000000000000 |
| ENSG00000272398 | 50.11204 | 23.89338 | 1.068546 | 0.000000000000 | 0.000000000000 |
| ENSG00000000971 | 17.56569 | 4.070459 | 2.109497 | 0.000000000000 | 0.000000000000 |
| ENSG00000177000 | 21.5137 | 9.562443 | 1.169805 | 0.000000000000 | 0.000000000000 |
| ENSG00000168234 | 55.9403 | 27.12397 | 1.04432 | 0.000000000000 | 0.000000000000 |
| ENSG00000170779 | 36.46066 | 12.84001 | 1.505694 | 0.000000000000 | 0.000000000000 |
| ENSG00000255339 | 58.6913 | 19.51516 | 1.588551 | 0.000000000000 | 0.000000000000 |
| ENSG00000105372 | 588.2961 | 1404.996 | -1.25595 | 0.000000000000 | 0.000000000000 |
| ENSG00000138685 | 7.628989 | 21.62127 | -1.50289 | 0.000000000000 | 0.000000000000 |
| ENSG00000047617 | 7.538409 | 26.37013 | -1.80657 | 0.000000000000 | 0.000000000000 |
| ENSG00000113558 | 252.2648 | 104.6955 | 1.268739 | 0.000000000000 | 0.000000000000 |
| ENSG00000182795 | 6.147115 | 16.52634 | -1.42679 | 0.000000000000 | 0.000000000000 |
| ENSG00000175197 | 38.03598 | 98.48229 | -1.3725 | 0.000000000000 | 0.000000000000 |
| ENSG00000033327 | 6.661526 | 17.65492 | -1.40615 | 0.000000000000 | 0.000000000000 |
| ENSG00000167447 | 17.75515 | 5.694531 | 1.640589 | 0.000000000000 | 0.000000000000 |
| ENSG00000151239 | 33.34925 | 13.22277 | 1.33463 | 0.000000000000 | 0.000000000000 |
| ENSG00000006611 | 36.07842 | 5.946612 | 2.600996 | 0.000000000000 | 0.000000000000 |
| ENSG00000180035 | 4.45434 | 16.54764 | -1.89334 | 0.000000000000 | 0.000000000000 |
| ENSG00000132003 | 18.87815 | 38.92604 | -1.04402 | 0.000000000000 | 0.000000000000 |
| ENSG00000006062 | 9.868215 | 24.04326 | -1.28477 | 0.000000000000 | 0.000000000000 |
| ENSG00000245910 | 218.0719 | 107.0308 | 1.026777 | 0.000000000000 | 0.000000000000 |
| ENSG00000117054 | 53.76198 | 14.06499 | 1.934478 | 0.000000000000 | 0.000000000000 |
| ENSG00000172780 | 19.37968 | 7.626609 | 1.345431 | 0.000000000000 | 0.000000000000 |
| ENSG00000170153 | 7.78548 | 2.603935 | 1.580093 | 0.000000000000 | 0.000000000000 |
| ENSG00000107099 | 19.89734 | 8.695357 | 1.194259 | 0.000000000000 | 0.000000000000 |
| ENSG00000133112 | 313.8133 | 139.8554 | 1.16597 | 0.000000000000 | 0.000000000000 |
| ENSG00000257390 | 1.316835 | 15.12403 | -3.5217 | 0.000000000000 | 0.000000000000 |
| ENSG00000183801 | 5.045249 | 20.74291 | -2.03962 | 0.000000000000 | 0.000000000000 |
| ENSG00000140688 | 51.81666 | 107.3603 | -1.05097 | 0.000000000000 | 0.000000000000 |
| ENSG00000276612 | 0.0001 | 20.71474 | -17.6603 | 0.000000000000 | 0.000000000000 |
| ENSG00000069535 | 42.02546 | 19.46027 | 1.110732 | 0.000000000000 | 0.000000000000 |
| ENSG00000183479 | 1.47458 | 12.53391 | -3.08746 | 0.000000000000 | 0.000000000000 |
| ENSG00000228502 | 61.60261 | 25.03862 | 1.298836 | 0.000000000000 | 0.000000000000 |
| ENSG00000141750 | 14.41151 | 3.689662 | 1.965661 | 0.000000000000 | 0.000000000000 |
| ENSG00000143367 | 17.12977 | 39.65257 | -1.21091 | 0.000000000000 | 0.000000000000 |
| ENSG00000183696 | 30.17527 | 65.20166 | -1.11154 | 0.000000000000 | 0.000000000000 |
| ENSG00000212719 | 4.379372 | 11.48042 | -1.39038 | 0.000000000000 | 0.000000000000 |
| ENSG00000172965 | 59.36145 | 149.9291 | -1.33668 | 0.000000000000 | 0.000000000000 |
| ENSG00000108825 | 6.462104 | 22.68096 | -1.81141 | 0.000000000000 | 0.000000000000 |
| ENSG00000171570 | 0.319772 | 8.550097 | -4.74083 | 0.000000000000 | 0.000000000000 |
| ENSG00000090539 | 29.96802 | 11.52904 | 1.378151 | 0.000000000000 | 0.000000000000 |
| ENSG00000144895 | 96.53768 | 45.34486 | 1.090153 | 0.000000000000 | 0.000000000000 |
| ENSG00000132823 | 58.34975 | 25.43351 | 1.197996 | 0.000000000000 | 0.000000000000 |
| ENSG00000138623 | 7.754066 | 29.16613 | -1.91127 | 0.000000000000 | 0.000000000000 |
| ENSG00000075651 | 23.96515 | 9.866661 | 1.280304 | 0.000000000000 | 0.000000000000 |
| ENSG00000143390 | 68.97915 | 28.97918 | 1.251144 | 0.000000000000 | 0.000000000000 |
| ENSG00000165169 | 29.78462 | 9.511756 | 1.646784 | 0.000000000000 | 0.000000000000 |
| ENSG00000070669 | 29.53699 | 70.20199 | -1.24899 | 0.000000000000 | 0.000000000000 |
| ENSG00000100292 | 47.91849 | 20.00753 | 1.260039 | 0.000000000000 | 0.000000000000 |
| ENSG00000129514 | 14.02201 | 3.289006 | 2.09197 | 0.000000000000 | 0.000000000000 |
| ENSG00000118193 | 7.85074 | 2.448234 | 1.681087 | 0.000000000000 | 0.000000000000 |
| ENSG00000163545 | 4.298685 | 15.50222 | -1.85051 | 0.000000000000 | 0.000000000000 |
| ENSG00000167797 | 73.34015 | 36.40022 | 1.010656 | 0.000000000000 | 0.000000000000 |
| ENSG00000128708 | 48.49409 | 22.43377 | 1.112137 | 0.000000000000 | 0.000000000000 |
| ENSG00000154127 | 6.813389 | 16.06333 | -1.23733 | 0.000000000000 | 0.000000000000 |
| ENSG00000115738 | 46.29166 | 14.11651 | 1.713369 | 0.000000000000 | 0.000000000000 |
| ENSG00000146072 | 5.69143 | 17.59484 | -1.62829 | 0.000000000000 | 0.000000000000 |
| ENSG00000138942 | 24.93481 | 9.444736 | 1.400579 | 0.000000000000 | 0.000000000000 |
| ENSG00000166689 | 7.859536 | 23.17032 | -1.55976 | 0.000000000000 | 0.000000000000 |
| ENSG00000140416 | 70.23589 | 146.5716 | -1.06133 | 0.000000000000 | 0.000000000000 |
| ENSG00000214389 | 138.9034 | 68.39671 | 1.022083 | 0.000000000000 | 0.000000000000 |
| ENSG00000114854 | 29.93264 | 90.19296 | -1.5913 | 0.000000000000 | 0.000000000000 |
| ENSG00000139531 | 22.21421 | 7.748486 | 1.519496 | 0.000000000000 | 0.000000000000 |
| ENSG00000185432 | 7.664958 | 0.93921 | 3.028758 | 0.000000000000 | 0.000000000000 |
| ENSG00000055955 | 14.26144 | 2.062194 | 2.789867 | 0.000000000000 | 0.000000000000 |
| ENSG00000166897 | 5.257161 | 12.95472 | -1.30112 | 0.000000000000 | 0.000000000000 |
| ENSG00000113407 | 97.26041 | 239.3131 | -1.29897 | 0.000000000000 | 0.000000000000 |
| ENSG00000139269 | 2.320173 | 13.89998 | -2.58278 | 0.000000000000 | 0.000000000000 |
| ENSG00000183291 | 53.87877 | 20.74058 | 1.377261 | 0.000000000000 | 0.000000000000 |
| ENSG00000047644 | 7.50699 | 16.8219 | -1.16403 | 0.000000000000 | 0.000000000000 |
| ENSG00000233369 | 50.24997 | 24.35354 | 1.044991 | 0.000000000000 | 0.000000000000 |
| ENSG00000167600 | 6.961202 | 22.22416 | -1.67472 | 0.000000000000 | 0.000000000000 |
| ENSG00000103260 | 45.99155 | 20.88909 | 1.138619 | 0.000000000000 | 0.000000000000 |
| ENSG00000164048 | 4.002345 | 13.90266 | -1.79644 | 0.000000000000 | 0.000000000000 |
| ENSG00000103353 | 16.6854 | 34.48526 | -1.04739 | 0.000000000000 | 0.000000000000 |
| ENSG00000268879 | 21.31878 | 101.0577 | -2.24498 | 0.000000000000 | 0.000000000000 |
| ENSG00000269955 | 15.31743 | 0.0001 | 17.22481 | 0.000000000000 | 0.000000000000 |
| ENSG00000172667 | 14.76769 | 7.112667 | 1.053981 | 0.000000000000 | 0.000000000000 |
| ENSG00000188112 | 3.415668 | 11.79637 | -1.7881 | 0.000000000000 | 0.000000000000 |
| ENSG00000164236 | 1.792662 | 5.770862 | -1.68668 | 0.000000000000 | 0.000000000000 |
| ENSG00000167635 | 14.53876 | 29.16063 | -1.00412 | 0.000000000000 | 0.000000000000 |
| ENSG00000172354 | 243.4419 | 505.7793 | -1.05493 | 0.000000000000 | 0.000000000000 |
| ENSG00000271447 | 43.85548 | 19.03827 | 1.203855 | 0.000000000000 | 0.000000000000 |
| ENSG00000147536 | 8.786242 | 18.07118 | -1.04037 | 0.000000000000 | 0.000000000000 |
| ENSG00000175592 | 13.16257 | 42.37821 | -1.68688 | 0.000000000000 | 0.000000000000 |
| ENSG00000154734 | 8.00856 | 19.09107 | -1.25328 | 0.000000000000 | 0.000000000000 |
| ENSG00000117983 | 8.766535 | 1.84788 | 2.246136 | 0.000000000000 | 0.000000000000 |
| ENSG00000128463 | 80.65986 | 32.45008 | 1.313629 | 0.000000000000 | 0.000000000000 |
| ENSG00000234797 | 56.5445 | 17.25008 | 1.712783 | 0.000000000000 | 0.000000000000 |
| ENSG00000125966 | 8.176608 | 19.18489 | -1.2304 | 0.000000000000 | 0.000000000000 |
| ENSG00000172183 | 12.29373 | 34.79675 | -1.50103 | 0.000000000000 | 0.000000000000 |
| ENSG00000205403 | 11.69289 | 1.149027 | 3.347147 | 0.000000000000 | 0.000000000000 |
| ENSG00000149311 | 21.54603 | 8.092015 | 1.412851 | 0.000000000000 | 0.000000000000 |
| ENSG00000023228 | 87.04068 | 42.0486 | 1.049632 | 0.000000000000 | 0.000000000000 |
| ENSG00000148426 | 25.00202 | 59.82249 | -1.25864 | 0.000000000000 | 0.000000000000 |
| ENSG00000197971 | 5.361972 | 12.86357 | -1.26246 | 0.000000000000 | 0.000000000000 |
| ENSG00000198668 | 52.7314 | 21.7092 | 1.280356 | 0.000000000000 | 0.000000000000 |
| ENSG00000180354 | 11.4292 | 4.452253 | 1.360117 | 0.000000000000 | 0.000000000000 |
| ENSG00000108064 | 5.698326 | 1.186444 | 2.263894 | 0.000000000000 | 0.000000000000 |
| ENSG00000128294 | 9.542148 | 3.193487 | 1.579181 | 0.000000000000 | 0.000000000000 |
| ENSG00000243137 | 1.968911 | 20.30757 | -3.36655 | 0.000000000000 | 0.000000000000 |
| ENSG00000168685 | 1.183603 | 7.602631 | -2.68331 | 0.000000000000 | 0.000000000000 |
| ENSG00000105879 | 4.730409 | 13.52643 | -1.51574 | 0.000000000000 | 0.000000000000 |
| ENSG00000082497 | 3.182686 | 16.71874 | -2.39315 | 0.000000000000 | 0.000000000000 |
| ENSG00000180573 | 39.94202 | 15.81472 | 1.336639 | 0.000000000000 | 0.000000000000 |
| ENSG00000272414 | 6.204491 | 0.680415 | 3.188826 | 0.000000000000 | 0.000000000000 |
| ENSG00000176907 | 3.961195 | 18.08891 | -2.1911 | 0.000000000000 | 0.000000000000 |
| ENSG00000107731 | 11.68872 | 5.433326 | 1.10521 | 0.000000000000 | 0.000000000000 |
| ENSG00000204941 | 0.366014 | 11.20667 | -4.93632 | 0.000000000000 | 0.000000000000 |
| ENSG00000072786 | 7.609546 | 16.25917 | -1.09537 | 0.000000000000 | 0.000000000000 |
| ENSG00000205765 | 10.85925 | 4.150264 | 1.38765 | 0.000000000000 | 0.000000000000 |
| ENSG00000173320 | 5.320085 | 1.487811 | 1.838258 | 0.000000000000 | 0.000000000000 |
| ENSG00000118496 | 6.146449 | 2.387528 | 1.364236 | 0.000000000000 | 0.000000000000 |
| ENSG00000196182 | 11.22324 | 27.85671 | -1.31154 | 0.000000000000 | 0.000000000000 |
| ENSG00000150782 | 19.68157 | 49.33057 | -1.32564 | 0.000000000000 | 0.000000000000 |
| ENSG00000214485 | 93.34318 | 41.80284 | 1.158944 | 0.000000000000 | 0.000000000000 |
| ENSG00000162645 | 9.023298 | 1.955655 | 2.206003 | 0.000000000000 | 0.000000000000 |
| ENSG00000127920 | 7.545259 | 19.91579 | -1.40027 | 0.000000000000 | 0.000000000000 |
| ENSG00000167191 | 1.018464 | 5.625278 | -2.46553 | 0.000000000000 | 0.000000000000 |
| ENSG00000268460 | 1.365369 | 13.01766 | -3.25311 | 0.000000000000 | 0.000000000000 |
| ENSG00000172650 | 11.20333 | 2.0352 | 2.460685 | 0.000000000000 | 0.000000000000 |
| ENSG00000100033 | 7.644488 | 23.39253 | -1.61356 | 0.000000000000 | 0.000000000000 |
| ENSG00000147789 | 15.48649 | 33.09272 | -1.0955 | 0.000000000000 | 0.000000000000 |
| ENSG00000275342 | 3.289503 | 10.18741 | -1.63085 | 0.000000000000 | 0.000000000000 |
| ENSG00000106789 | 6.922274 | 15.30681 | -1.14486 | 0.000000000000 | 0.000000000000 |
| ENSG00000188910 | 7.341332 | 22.19155 | -1.5959 | 0.000000000000 | 0.000000000000 |
| ENSG00000155287 | 54.04807 | 24.07488 | 1.166714 | 0.000000000000 | 0.000000000000 |
| ENSG00000152377 | 9.557899 | 21.691 | -1.18233 | 0.000000000000 | 0.000000000000 |
| ENSG00000255860 | 1.649386 | 24.16694 | -3.87303 | 0.000000000000 | 0.000000000000 |
| ENSG00000116711 | 21.21326 | 9.008233 | 1.235651 | 0.000000000000 | 0.000000000000 |
| ENSG00000108176 | 48.66774 | 21.26234 | 1.194665 | 0.000000000000 | 0.000000000000 |
| ENSG00000103066 | 12.16975 | 27.22361 | -1.16156 | 0.000000000000 | 0.000000000000 |
| ENSG00000020577 | 4.143133 | 11.62556 | -1.48851 | 0.000000000000 | 0.000000000000 |
| ENSG00000101246 | 19.36991 | 44.58841 | -1.20285 | 0.000000000000 | 0.000000000000 |
| ENSG00000213977 | 32.22167 | 66.82596 | -1.05238 | 0.000000000000 | 0.000000000000 |
| ENSG00000273088 | 25.56524 | 0.0001 | 17.96382 | 0.000000000000 | 0.000000000000 |
| ENSG00000160200 | 13.37004 | 30.50477 | -1.19003 | 0.000000000000 | 0.000000000000 |
| ENSG00000138018 | 5.05156 | 1.42874 | 1.821986 | 0.000000000000 | 0.000000000000 |
| ENSG00000151229 | 10.19556 | 2.93948 | 1.794308 | 0.000000000000 | 0.000000000000 |
| ENSG00000127329 | 7.455741 | 1.815496 | 2.037988 | 0.000000000000 | 0.000000000000 |
| ENSG00000215492 | 75.43398 | 36.09269 | 1.063508 | 0.000000000000 | 0.000000000000 |
| ENSG00000118523 | 0.50747 | 7.105779 | -3.8076 | 0.000000000000 | 0.000000000000 |
| ENSG00000082258 | 7.449181 | 2.833947 | 1.394269 | 0.000000000000 | 0.000000000000 |
| ENSG00000163898 | 4.014787 | 12.30018 | -1.61528 | 0.000000000000 | 0.000000000000 |
| ENSG00000273000 | 3.378614 | 9.849027 | -1.54355 | 0.000000000000 | 0.000000000000 |
| ENSG00000047457 | 11.45414 | 1.898502 | 2.592936 | 0.000000000000 | 0.000000000000 |
| ENSG00000075420 | 31.80343 | 66.00932 | -1.05349 | 0.000000000000 | 0.000000000000 |
| ENSG00000069020 | 8.488106 | 4.104088 | 1.048381 | 0.000000000000 | 0.000000000000 |
| ENSG00000126368 | 8.532321 | 21.36758 | -1.32441 | 0.000000000000 | 0.000000000000 |
| ENSG00000196214 | 2.109308 | 5.998098 | -1.50774 | 0.000000000000 | 0.000000000000 |
| ENSG00000267519 | 21.28296 | 45.52696 | -1.09702 | 0.000000000000 | 0.000000000000 |
| ENSG00000115252 | 25.96231 | 7.610585 | 1.77034 | 0.000000000000 | 0.000000000000 |
| ENSG00000104825 | 16.99473 | 40.46269 | -1.2515 | 0.000000000000 | 0.000000000000 |
| ENSG00000072163 | 8.757208 | 30.2634 | -1.78903 | 0.000000000000 | 0.000000000000 |
| ENSG00000161249 | 49.28587 | 107.6484 | -1.12708 | 0.000000000000 | 0.000000000000 |
| ENSG00000116514 | 18.22044 | 36.93905 | -1.01959 | 0.000000000000 | 0.000000000000 |
| ENSG00000248592 | 9.477839 | 0.129654 | 6.19182 | 0.000000000000 | 0.000000000000 |
| ENSG00000140961 | 6.677604 | 25.03589 | -1.9066 | 0.000000000000 | 0.000000000000 |
| ENSG00000100814 | 18.22299 | 40.24072 | -1.1429 | 0.000000000000 | 0.000000000000 |
| ENSG00000164294 | 10.34681 | 23.9829 | -1.21282 | 0.000000000000 | 0.000000000000 |
| ENSG00000139793 | 25.66692 | 52.67363 | -1.03717 | 0.000000000000 | 0.000000000000 |
| ENSG00000149591 | 8.706709 | 33.9072 | -1.96139 | 0.000000000000 | 0.000000000000 |
| ENSG00000198146 | 14.52334 | 7.193478 | 1.013611 | 0.000000000000 | 0.000000000000 |
| ENSG00000069424 | 27.48286 | 58.10721 | -1.08018 | 0.000000000000 | 0.000000000000 |
| ENSG00000260260 | 168.479 | 75.08788 | 1.165917 | 0.000000000000 | 0.000000000000 |
| ENSG00000198862 | 9.334195 | 4.071027 | 1.197133 | 0.000000000000 | 0.000000000000 |
| ENSG00000152256 | 57.76468 | 17.14849 | 1.752106 | 0.000000000000 | 0.000000000000 |
| ENSG00000142327 | 19.53641 | 58.56077 | -1.58377 | 0.000000000000 | 0.000000000000 |
| ENSG00000176170 | 21.77656 | 44.2212 | -1.02196 | 0.000000000000 | 0.000000000000 |
| ENSG00000141682 | 7.695209 | 21.85617 | -1.50601 | 0.000000000000 | 0.000000000000 |
| ENSG00000078401 | 4.702872 | 16.50404 | -1.81121 | 0.000000000000 | 0.000000000000 |
| ENSG00000116161 | 67.98969 | 33.25993 | 1.031531 | 0.000000000000 | 0.000000000000 |
| ENSG00000137414 | 13.1132 | 6.190029 | 1.083001 | 0.000000000000 | 0.000000000000 |
| ENSG00000198369 | 9.186329 | 20.19675 | -1.13656 | 0.000000000000 | 0.000000000000 |
| ENSG00000099994 | 3.558857 | 11.04396 | -1.63377 | 0.000000000000 | 0.000000000000 |
| ENSG00000051108 | 62.56398 | 171.0305 | -1.45085 | 0.000000000000 | 0.000000000000 |
| ENSG00000115255 | 32.43572 | 14.39663 | 1.171852 | 0.000000000000 | 0.000000000000 |
| ENSG00000145632 | 239.6736 | 115.8055 | 1.049368 | 0.000000000000 | 0.000000000000 |
| ENSG00000129315 | 16.82823 | 7.439802 | 1.177547 | 0.000000000000 | 0.000000000000 |
| ENSG00000166949 | 52.02271 | 107.3908 | -1.04566 | 0.000000000000 | 0.000000000000 |
| ENSG00000248167 | 8.813608 | 0.0001 | 16.42745 | 0.000000000000 | 0.000000000000 |
| ENSG00000059804 | 0.46031 | 7.904806 | -4.10205 | 0.000000000000 | 0.000000000000 |
| ENSG00000162688 | 7.256157 | 3.303396 | 1.135256 | 0.000000000000 | 0.000000000000 |
| ENSG00000182871 | 3.84564 | 10.48701 | -1.44731 | 0.000000000000 | 0.000000000000 |
| ENSG00000126653 | 8.412362 | 27.62474 | -1.71538 | 0.000000000000 | 0.000000000000 |
| ENSG00000203706 | 8.164653 | 36.39032 | -2.15609 | 0.000000000000 | 0.000000000000 |
| ENSG00000011465 | 10.02476 | 1.323328 | 2.921325 | 0.000000000000 | 0.000000000000 |
| ENSG00000274276 | 8.418365 | 21.19039 | -1.3318 | 0.000000000000 | 0.000000000000 |
| ENSG00000087266 | 81.92847 | 38.49459 | 1.089709 | 0.000000000000 | 0.000000000000 |
| ENSG00000171916 | 6.649242 | 26.77542 | -2.00965 | 0.000000000000 | 0.000000000000 |
| ENSG00000071794 | 19.75002 | 6.829925 | 1.531913 | 0.000000000000 | 0.000000000000 |
| ENSG00000128965 | 2.00728 | 12.50558 | -2.63926 | 0.000000000000 | 0.000000000000 |
| ENSG00000132952 | 7.493647 | 3.523852 | 1.088515 | 0.000000000000 | 0.000000000000 |
| ENSG00000278299 | 0.0001 | 6.699725 | -16.0318 | 0.000000000000 | 0.000000000000 |
| ENSG00000157510 | 12.64947 | 25.77798 | -1.02706 | 0.000000000000 | 0.000000000000 |
| ENSG00000107201 | 5.63781 | 12.62329 | -1.16288 | 0.000000000000 | 0.000000000000 |
| ENSG00000174327 | 3.459972 | 14.60335 | -2.07747 | 0.000000000000 | 0.000000000000 |
| ENSG00000210194 | 352.3842 | 90.62433 | 1.959179 | 0.000000000000 | 0.000000000000 |
| ENSG00000204267 | 35.03219 | 97.62315 | -1.47854 | 0.000000000000 | 0.000000000000 |
| ENSG00000027869 | 22.07924 | 49.5309 | -1.16564 | 0.000000000000 | 0.000000000000 |
| ENSG00000166004 | 10.5609 | 4.970973 | 1.087133 | 0.000000000000 | 0.000000000000 |
| ENSG00000101596 | 13.25571 | 6.042057 | 1.133502 | 0.000000000000 | 0.000000000000 |
| ENSG00000198585 | 10.12229 | 4.979214 | 1.023546 | 0.000000000000 | 0.000000000000 |
| ENSG00000205413 | 5.244119 | 1.934027 | 1.439092 | 0.000000000000 | 0.000000000000 |
| ENSG00000258289 | 20.38861 | 7.053098 | 1.531434 | 0.000000000000 | 0.000000000000 |
| ENSG00000283154 | 17.71559 | 36.72746 | -1.05184 | 0.000000000000 | 0.000000000000 |
| ENSG00000071189 | 21.39494 | 8.104114 | 1.400543 | 0.000000000000 | 0.000000000000 |
| ENSG00000214944 | 2.24694 | 7.129524 | -1.66584 | 0.000000000000 | 0.000000000000 |
| ENSG00000284431 | 8.171803 | 16.41863 | -1.00661 | 0.000000000000 | 0.000000000000 |
| ENSG00000183826 | 7.122818 | 3.422076 | 1.057576 | 0.000000000000 | 0.000000000000 |
| ENSG00000156136 | 5.84138 | 1.013593 | 2.526831 | 0.000000000000 | 0.000000000000 |
| ENSG00000198894 | 8.119675 | 3.373883 | 1.267012 | 0.000000000000 | 0.000000000000 |
| ENSG00000124102 | 4.780984 | 30.39834 | -2.66861 | 0.000000000000 | 0.000000000000 |
| ENSG00000110025 | 8.627399 | 17.97974 | -1.05937 | 0.000000000000 | 0.000000000000 |
| ENSG00000253270 | 1.884361 | 27.07085 | -3.84459 | 0.000000000000 | 0.000000000000 |
| ENSG00000209082 | 316.9012 | 83.60369 | 1.922394 | 0.000000000000 | 0.000000000000 |
| ENSG00000085117 | 32.04839 | 9.647872 | 1.731969 | 0.000000000000 | 0.000000000000 |
| ENSG00000170525 | 13.61358 | 32.07465 | -1.23639 | 0.000000000000 | 0.000000000000 |
| ENSG00000198223 | 7.528271 | 20.14739 | -1.4202 | 0.000000000000 | 0.000000000000 |
| ENSG00000171604 | 61.70652 | 29.47308 | 1.066025 | 0.000000000000 | 0.000000000000 |
| ENSG00000125954 | 0.0001 | 8.950398 | -16.4497 | 0.000000000000 | 0.000000000000 |
| ENSG00000116704 | 8.240123 | 3.839228 | 1.10185 | 0.000000000000 | 0.000000000000 |
| ENSG00000133710 | 10.00046 | 3.487135 | 1.519953 | 0.000000000000 | 0.000000000000 |
| ENSG00000270504 | 8.636332 | 19.53524 | -1.17759 | 0.000000000000 | 0.000000000000 |
| ENSG00000115009 | 1.45277 | 15.88397 | -3.45069 | 0.000000000000 | 0.000000000000 |
| ENSG00000171155 | 24.4075 | 10.00044 | 1.28726 | 0.000000000000 | 0.000000000000 |
| ENSG00000257671 | 12.41437 | 29.14229 | -1.2311 | 0.000000000000 | 0.000000000000 |
| ENSG00000249992 | 2.509922 | 11.49298 | -2.19504 | 0.000000000000 | 0.000000000000 |
| ENSG00000204758 | 2.612242 | 15.16679 | -2.53756 | 0.000000000000 | 0.000000000000 |
| ENSG00000162636 | 7.303399 | 2.910681 | 1.327211 | 0.000000000000 | 0.000000000000 |
| ENSG00000254979 | 5.664546 | 14.67962 | -1.37378 | 0.000000000000 | 0.000000000000 |
| ENSG00000163083 | 1.280648 | 6.149646 | -2.26363 | 0.000000000000 | 0.000000000000 |
| ENSG00000151014 | 10.49137 | 25.44338 | -1.27809 | 0.000000000000 | 0.000000000000 |
| ENSG00000104613 | 88.19218 | 41.22834 | 1.097015 | 0.000000000000 | 0.000000000000 |
| ENSG00000174473 | 5.007505 | 1.176522 | 2.089564 | 0.000000000000 | 0.000000000000 |
| ENSG00000166473 | 0.798092 | 6.027668 | -2.91697 | 0.000000000000 | 0.000000000000 |
| ENSG00000165929 | 5.929895 | 1.185057 | 2.32305 | 0.000000000000 | 0.000000000000 |
| ENSG00000130844 | 20.45229 | 10.176 | 1.007092 | 0.000000000000 | 0.000000000000 |
| ENSG00000085719 | 8.144391 | 17.68343 | -1.11852 | 0.000000000000 | 0.000000000000 |
| ENSG00000041988 | 20.15984 | 44.37545 | -1.13828 | 0.000000000000 | 0.000000000000 |
| ENSG00000121774 | 93.1381 | 221.4473 | -1.24952 | 0.000000000000 | 0.000000000000 |
| ENSG00000196196 | 13.73221 | 37.57282 | -1.45212 | 0.000000000000 | 0.000000000000 |
| ENSG00000234912 | 2.366711 | 9.335606 | -1.97986 | 0.000000000000 | 0.000000000000 |
| ENSG00000077713 | 5.446009 | 12.69908 | -1.22145 | 0.000000000000 | 0.000000000000 |
| ENSG00000167779 | 26.98938 | 54.6252 | -1.01717 | 0.000000000000 | 0.000000000000 |
| ENSG00000243566 | 5.24191 | 20.93468 | -1.99773 | 0.000000000000 | 0.000000000000 |
| ENSG00000185561 | 5.530388 | 2.132923 | 1.374549 | 0.000000000000 | 0.000000000000 |
| ENSG00000052802 | 9.020772 | 2.588246 | 1.801276 | 0.000000000000 | 0.000000000000 |
| ENSG00000183929 | 26.16608 | 9.420673 | 1.473796 | 0.000000000000 | 0.000000000000 |
| ENSG00000104611 | 22.93129 | 47.81968 | -1.06029 | 0.000000000000 | 0.000000000000 |
| ENSG00000166575 | 8.415404 | 2.129299 | 1.982654 | 0.000000000000 | 0.000000000000 |
| ENSG00000092969 | 3.199069 | 8.052628 | -1.33181 | 0.000000000000 | 0.000000000000 |
| ENSG00000118849 | 19.59371 | 6.480159 | 1.596289 | 0.000000000000 | 0.000000000000 |
| ENSG00000272808 | 5.005835 | 10.74666 | -1.10221 | 0.000000000000 | 0.000000000000 |
| ENSG00000162006 | 20.85847 | 5.120699 | 2.02622 | 0.000000000000 | 0.000000000000 |
| ENSG00000158882 | 7.029928 | 17.49189 | -1.3151 | 0.000000000000 | 0.000000000000 |
| ENSG00000228223 | 12.12265 | 5.165976 | 1.230592 | 0.000000000000 | 0.000000000000 |
| ENSG00000277701 | 5.126956 | 1.019497 | 2.330245 | 0.000000000000 | 0.000000000000 |
| ENSG00000238266 | 6.769897 | 15.40596 | -1.18628 | 0.000000000000 | 0.000000000000 |
| ENSG00000221995 | 5.72629 | 11.55238 | -1.01252 | 0.000000000000 | 0.000000000000 |
| ENSG00000214900 | 3.401938 | 12.96652 | -1.93036 | 0.000000000000 | 0.000000000000 |
| ENSG00000183508 | 7.352125 | 3.370196 | 1.125329 | 0.000000000000 | 0.000000000000 |
| ENSG00000111530 | 47.7528 | 23.43276 | 1.027059 | 0.000000000000 | 0.000000000000 |
| ENSG00000187017 | 9.379729 | 3.391159 | 1.467768 | 0.000000000000 | 0.000000000000 |
| ENSG00000277971 | 1.406256 | 17.03695 | -3.59874 | 0.000000000000 | 0.000000000000 |
| ENSG00000125898 | 5.791785 | 16.10064 | -1.47504 | 0.000000000000 | 0.000000000000 |
| ENSG00000198860 | 16.99982 | 7.673226 | 1.147614 | 0.000000000000 | 0.000000000000 |
| ENSG00000133328 | 37.19742 | 76.54984 | -1.0412 | 0.000000000000 | 0.000000000000 |
| ENSG00000198182 | 5.363544 | 1.827426 | 1.553374 | 0.000000000000 | 0.000000000000 |
| ENSG00000155545 | 5.995603 | 2.248186 | 1.415143 | 0.000000000000 | 0.000000000000 |
| ENSG00000072422 | 12.61824 | 6.295423 | 1.003135 | 0.000000000000 | 0.000000000000 |
| ENSG00000110218 | 8.803641 | 18.72831 | -1.08905 | 0.000000000000 | 0.000000000000 |
| ENSG00000181350 | 2.888177 | 10.88092 | -1.91357 | 0.000000000000 | 0.000000000000 |
| ENSG00000263740 | 72.37756 | 160.4339 | -1.14836 | 0.000000000000 | 0.000000000000 |
| ENSG00000168765 | 22.15633 | 45.43968 | -1.03623 | 0.000000000000 | 0.000000000000 |
| ENSG00000276850 | 12.65417 | 28.84908 | -1.18891 | 0.000000000000 | 0.000000000000 |
| ENSG00000164674 | 2.954086 | 8.574103 | -1.53727 | 0.000000000000 | 0.000000000000 |
| ENSG00000168952 | 2.86823 | 8.114644 | -1.50037 | 0.000000000000 | 0.000000000000 |
| ENSG00000174804 | 5.502957 | 2.544693 | 1.112715 | 0.000000000000 | 0.000000000000 |
| ENSG00000147168 | 5.840919 | 20.32346 | -1.79888 | 0.000000000000 | 0.000000000000 |
| ENSG00000248751 | 0.0001 | 5.057969 | -15.6263 | 0.000000000000 | 0.000000000000 |
| ENSG00000143320 | 32.26756 | 78.36319 | -1.28009 | 0.000000000000 | 0.000000000000 |
| ENSG00000210191 | 311.8642 | 96.94064 | 1.685744 | 0.000000000000 | 0.000000000000 |
| ENSG00000144460 | 4.43725 | 9.349642 | -1.07525 | 0.000000000000 | 0.000000000000 |
| ENSG00000281490 | 13.86541 | 6.252997 | 1.148871 | 0.000000000000 | 0.000000000000 |
| ENSG00000095739 | 12.71399 | 26.84948 | -1.07848 | 0.000000000000 | 0.000000000000 |
| ENSG00000165685 | 1.097797 | 10.35095 | -3.23708 | 0.000000000000 | 0.000000000000 |
| ENSG00000067191 | 10.65727 | 3.483206 | 1.61335 | 0.000000000000 | 0.000000000000 |
| ENSG00000142583 | 5.578845 | 13.64367 | -1.29019 | 0.000000000000 | 0.000000000000 |
| ENSG00000164070 | 6.633367 | 13.85724 | -1.06283 | 0.000000000000 | 0.000000000000 |
| ENSG00000114209 | 20.9603 | 45.22402 | -1.10943 | 0.000000000000 | 0.000000000000 |
| ENSG00000275215 | 130.9144 | 38.84138 | 1.752957 | 0.000000000000 | 0.000000000000 |
| ENSG00000278334 | 0.030694 | 44.19896 | -10.4918 | 0.000000000000 | 0.000000000000 |
| ENSG00000168502 | 15.81268 | 32.08633 | -1.02088 | 0.000000000000 | 0.000000000000 |
| ENSG00000196872 | 8.520631 | 20.85468 | -1.29134 | 0.000000000000 | 0.000000000000 |
| ENSG00000119900 | 3.517731 | 7.284561 | -1.0502 | 0.000000000000 | 0.000000000000 |
| ENSG00000084731 | 2.222118 | 6.003907 | -1.43397 | 0.000000000000 | 0.000000000000 |
| ENSG00000197969 | 5.705082 | 2.627396 | 1.118614 | 0.000000000000 | 0.000000000000 |
| ENSG00000130940 | 4.388596 | 9.377694 | -1.09547 | 0.000000000000 | 0.000000000000 |
| ENSG00000139163 | 9.393798 | 3.220998 | 1.544201 | 0.000000000000 | 0.000000000000 |
| ENSG00000210184 | 402.4044 | 142.6896 | 1.495766 | 0.000000000000 | 0.000000000000 |
| ENSG00000244122 | 15.9299 | 7.47476 | 1.091638 | 0.000000000000 | 0.000000000000 |
| ENSG00000176697 | 5.406145 | 11.5529 | -1.09558 | 0.000000000000 | 0.000000000000 |
| ENSG00000123838 | 5.509568 | 0.717635 | 2.940617 | 0.000000000000 | 0.000000000000 |
| ENSG00000118804 | 4.064575 | 9.910404 | -1.28584 | 0.000000000000 | 0.000000000000 |
| ENSG00000196428 | 7.928784 | 17.4708 | -1.13977 | 0.000000000000 | 0.000000000000 |
| ENSG00000228315 | 12.68395 | 5.637286 | 1.169932 | 0.000000000000 | 0.000000000000 |
| ENSG00000137285 | 6.380656 | 15.9355 | -1.32047 | 0.000000000000 | 0.000000000000 |
| ENSG00000267530 | 15.32524 | 39.68639 | -1.37273 | 0.000000000000 | 0.000000000000 |
| ENSG00000198898 | 21.92592 | 8.312141 | 1.399345 | 0.000000000000 | 0.000000000000 |
| ENSG00000205084 | 4.816947 | 18.10611 | -1.91029 | 0.000000000000 | 0.000000000000 |
| ENSG00000115902 | 4.665025 | 10.45629 | -1.16441 | 0.000000000000 | 0.000000000000 |
| ENSG00000163235 | 2.405673 | 7.40124 | -1.62133 | 0.000000000000 | 0.000000000000 |
| ENSG00000155729 | 9.483393 | 3.900583 | 1.281714 | 0.000000000000 | 0.000000000000 |
| ENSG00000280433 | 2.175245 | 6.308181 | -1.53605 | 0.000000000000 | 0.000000000000 |
| ENSG00000232472 | 16.00592 | 2.493145 | 2.682567 | 0.000000000000 | 0.000000000000 |
| ENSG00000136122 | 7.309694 | 2.501409 | 1.54707 | 0.000000000000 | 0.000000000000 |
| ENSG00000204257 | 29.09909 | 12.19137 | 1.255114 | 0.000000000000 | 0.000000000000 |
| ENSG00000151876 | 20.78063 | 8.616144 | 1.270125 | 0.000000000000 | 0.000000000000 |
| ENSG00000115594 | 8.721858 | 3.955136 | 1.140908 | 0.000000000000 | 0.000000000000 |
| ENSG00000186866 | 38.01929 | 79.40298 | -1.06246 | 0.000000000000 | 0.000000000000 |
| ENSG00000167550 | 8.494582 | 26.23334 | -1.62679 | 0.000000000000 | 0.000000000000 |
| ENSG00000257732 | 2.391289 | 16.02687 | -2.74463 | 0.000000000000 | 0.000000000000 |
| ENSG00000125347 | 16.7981 | 34.93863 | -1.05653 | 0.000000000000 | 0.000000000000 |
| ENSG00000270757 | 12.05216 | 1.947285 | 2.629756 | 0.000000000000 | 0.000000000000 |
| ENSG00000225972 | 44.02619 | 12.42344 | 1.825297 | 0.000000000000 | 0.000000000000 |
| ENSG00000029153 | 2.415703 | 7.158309 | -1.56718 | 0.000000000000 | 0.000000000000 |
| ENSG00000197006 | 36.54185 | 12.86291 | 1.506333 | 0.000000000000 | 0.000000000000 |
| ENSG00000166073 | 6.283751 | 13.11103 | -1.06108 | 0.000000000000 | 0.000000000000 |
| ENSG00000137393 | 2.698996 | 6.452221 | -1.25737 | 0.000000000000 | 0.000000000000 |
| ENSG00000204396 | 19.06735 | 7.004081 | 1.444837 | 0.000000000000 | 0.000000000000 |
| ENSG00000152270 | 5.991162 | 1.751267 | 1.774437 | 0.000000000000 | 0.000000000000 |
| ENSG00000127993 | 11.61225 | 2.521735 | 2.203159 | 0.000000000000 | 0.000000000000 |
| ENSG00000277209 | 32.54404 | 5.633257 | 2.530352 | 0.000000000000 | 0.000000000000 |
| ENSG00000188647 | 14.57584 | 7.2114 | 1.015227 | 0.000000000000 | 0.000000000000 |
| ENSG00000003137 | 9.983809 | 22.79785 | -1.19124 | 0.000000000000 | 0.000000000000 |
| ENSG00000130159 | 30.75902 | 14.7471 | 1.060579 | 0.000000000000 | 0.000000000000 |
| ENSG00000183722 | 5.058035 | 13.00624 | -1.36256 | 0.000000000000 | 0.000000000000 |
| ENSG00000162241 | 4.837348 | 16.04423 | -1.72977 | 0.000000000000 | 0.000000000000 |
| ENSG00000139263 | 5.408374 | 11.63299 | -1.10496 | 0.000000000000 | 0.000000000000 |
| ENSG00000230699 | 5.402083 | 1.590714 | 1.763841 | 0.000000000000 | 0.000000000000 |
| ENSG00000277739 | 116.2276 | 36.36565 | 1.676305 | 0.000000000000 | 0.000000000000 |
| ENSG00000145911 | 3.174284 | 6.686694 | -1.07486 | 0.000000000000 | 0.000000000000 |
| ENSG00000113070 | 3.332738 | 9.560733 | -1.52041 | 0.000000000000 | 0.000000000000 |
| ENSG00000137944 | 11.58225 | 4.243639 | 1.448542 | 0.000000000000 | 0.000000000000 |
| ENSG00000217716 | 51.75352 | 21.30141 | 1.280708 | 0.000000000000 | 0.000000000000 |
| ENSG00000105971 | 19.48209 | 41.44701 | -1.08912 | 0.000000000000 | 0.000000000000 |
| ENSG00000226145 | 4.10813 | 18.43274 | -2.16572 | 0.000000000000 | 0.000000000000 |
| ENSG00000165338 | 6.776588 | 1.608446 | 2.074892 | 0.000000000000 | 0.000000000000 |
| ENSG00000156453 | 7.251198 | 19.08578 | -1.39621 | 0.000000000000 | 0.000000000000 |
| ENSG00000179598 | 5.836413 | 13.24537 | -1.18233 | 0.000000000000 | 0.000000000000 |
| ENSG00000214922 | 6.044002 | 19.59513 | -1.69692 | 0.000000000000 | 0.000000000000 |
| ENSG00000123213 | 4.814337 | 13.20238 | -1.45539 | 0.000000000000 | 0.000000000000 |
| ENSG00000162813 | 44.03944 | 21.21788 | 1.053515 | 0.000000000000 | 0.000000000000 |
| ENSG00000223784 | 9.394662 | 25.86388 | -1.46103 | 0.000000000000 | 0.000000000000 |
| ENSG00000250182 | 10.79489 | 3.026227 | 1.834757 | 0.000000000000 | 0.000000000000 |
| ENSG00000283515 | 4.311295 | 9.956838 | -1.20757 | 0.000000000000 | 0.000000000000 |
| ENSG00000164951 | 6.682982 | 19.38303 | -1.53623 | 0.000000000000 | 0.000000000000 |
| ENSG00000154265 | 6.67147 | 3.224083 | 1.049116 | 0.000000000000 | 0.000000000000 |
| ENSG00000104953 | 13.6627 | 28.55182 | -1.06334 | 0.000000000000 | 0.000000000000 |
| ENSG00000166200 | 19.94892 | 2.271461 | 3.134618 | 0.000000000000 | 0.000000000000 |
| ENSG00000278233 | 111.1666 | 36.0295 | 1.625473 | 0.000000000000 | 0.000000000000 |
| ENSG00000197565 | 5.844309 | 1.859187 | 1.652361 | 0.000000000000 | 0.000000000000 |
| ENSG00000064270 | 4.683844 | 10.42383 | -1.15412 | 0.000000000000 | 0.000000000000 |
| ENSG00000260836 | 6.69362 | 0.16052 | 5.381962 | 0.000000000000 | 0.000000000000 |
| ENSG00000236824 | 112.7117 | 44.87355 | 1.3287 | 0.000000000000 | 0.000000000000 |
| ENSG00000273173 | 7.415818 | 29.15132 | -1.97488 | 0.000000000000 | 0.000000000000 |
| ENSG00000106714 | 7.067959 | 3.171883 | 1.155954 | 0.000000000000 | 0.000000000000 |
| ENSG00000108510 | 24.65158 | 9.964604 | 1.306795 | 0.000000000000 | 0.000000000000 |
| ENSG00000166407 | 11.83616 | 3.222923 | 1.876759 | 0.000000000000 | 0.000000000000 |
| ENSG00000171462 | 5.590249 | 14.30094 | -1.35513 | 0.000000000000 | 0.000000000000 |
| ENSG00000225031 | 13.33343 | 5.615624 | 1.24753 | 0.000000000000 | 0.000000000000 |
| ENSG00000256206 | 1.102352 | 7.654489 | -2.79572 | 0.000000000000 | 0.000000000000 |
| ENSG00000235750 | 5.184176 | 10.92835 | -1.07589 | 0.000000000000 | 0.000000000000 |
| ENSG00000167315 | 39.7917 | 80.61488 | -1.01858 | 0.000000000000 | 0.000000000000 |
| ENSG00000135317 | 17.41166 | 5.039798 | 1.788616 | 0.000000000000 | 0.000000000000 |
| ENSG00000074047 | 1.474928 | 5.827523 | -1.98224 | 0.000000000000 | 0.000000000000 |
| ENSG00000145819 | 3.226203 | 8.338859 | -1.37001 | 0.000000000000 | 0.000000000000 |
| ENSG00000083857 | 11.00339 | 22.72935 | -1.04661 | 0.000000000000 | 0.000000000000 |
| ENSG00000196757 | 5.535344 | 1.846463 | 1.583909 | 0.000000000000 | 0.000000000000 |
| ENSG00000094841 | 11.05879 | 4.924454 | 1.167158 | 0.000000000000 | 0.000000000000 |
| ENSG00000105929 | 1.321568 | 5.485311 | -2.05332 | 0.000000000000 | 0.000000000000 |
| ENSG00000234498 | 7.608696 | 25.91884 | -1.76828 | 0.000000000000 | 0.000000000000 |
| ENSG00000284461 | 3.403725 | 7.424412 | -1.12516 | 0.000000000000 | 0.000000000000 |
| ENSG00000105427 | 55.74442 | 26.81832 | 1.055608 | 0.000000000000 | 0.000000000000 |
| ENSG00000163633 | 4.254445 | 15.71704 | -1.88529 | 0.000000000000 | 0.000000000000 |
| ENSG00000182400 | 5.100638 | 1.820256 | 1.486536 | 0.000000000000 | 0.000000000000 |
| ENSG00000133138 | 6.685957 | 2.748084 | 1.282708 | 0.000000000000 | 0.000000000000 |
| ENSG00000168477 | 4.558661 | 9.451612 | -1.05195 | 0.000000000000 | 0.000000000000 |
| ENSG00000250208 | 14.31124 | 6.171992 | 1.213341 | 0.000000000000 | 0.000000000000 |
| ENSG00000168490 | 15.95343 | 4.421131 | 1.851379 | 0.000000000000 | 0.000000000000 |
| ENSG00000187098 | 16.8473 | 45.21529 | -1.42429 | 0.000000000000 | 0.000000000000 |
| ENSG00000202538 | 42.25275 | 0.0001 | 18.68869 | 0.000000000000 | 0.000000000000 |
| ENSG00000235655 | 67.1379 | 31.7098 | 1.082199 | 0.000000000000 | 0.000000000000 |
| ENSG00000171621 | 5.990388 | 12.52979 | -1.06464 | 0.000000000000 | 0.000000000000 |
| ENSG00000122958 | 11.70154 | 5.28142 | 1.147701 | 0.000000000000 | 0.000000000000 |
| ENSG00000148908 | 14.74256 | 33.45702 | -1.18232 | 0.000000000000 | 0.000000000000 |
| ENSG00000170190 | 6.726677 | 15.19856 | -1.17597 | 0.000000000000 | 0.000000000000 |
| ENSG00000158158 | 6.570539 | 15.80486 | -1.26628 | 0.000000000000 | 0.000000000000 |
| ENSG00000246273 | 5.04835 | 16.84321 | -1.73828 | 0.000000000000 | 0.000000000000 |
| ENSG00000130935 | 41.38559 | 19.81117 | 1.062814 | 0.000000000000 | 0.000000000000 |
| ENSG00000138592 | 26.04163 | 12.58499 | 1.049116 | 0.000000000000 | 0.000000000000 |
| ENSG00000271335 | 7.677238 | 18.15576 | -1.24177 | 0.000000000000 | 0.000000000000 |
| ENSG00000173432 | 2.663166 | 15.16456 | -2.50949 | 0.000000000000 | 0.000000000000 |
| ENSG00000273541 | 14.97232 | 0.116997 | 6.999682 | 0.000000000000 | 0.000000000000 |
| ENSG00000100802 | 17.87061 | 7.406551 | 1.270715 | 0.000000000000 | 0.000000000000 |
| ENSG00000131171 | 9.801673 | 3.269634 | 1.583899 | 0.000000000000 | 0.000000000000 |
| ENSG00000182287 | 5.601254 | 1.539209 | 1.863561 | 0.000000000000 | 0.000000000000 |
| ENSG00000235174 | 105.9506 | 38.58744 | 1.457188 | 0.000000000000 | 0.000000000000 |
| ENSG00000269242 | 11.81838 | 2.636641 | 2.164259 | 0.000000000000 | 0.000000000000 |
| ENSG00000104164 | 11.68952 | 5.436001 | 1.104597 | 0.000000000000 | 0.000000000000 |
| ENSG00000172869 | 16.20083 | 6.527703 | 1.31142 | 0.000000000000 | 0.000000000000 |
| ENSG00000149380 | 9.684098 | 4.044387 | 1.259697 | 0.000000000000 | 0.000000000000 |
| ENSG00000183607 | 1.88114 | 10.92316 | -2.53771 | 0.000000000000 | 0.000000000000 |
| ENSG00000100867 | 2.931396 | 8.322936 | -1.5055 | 0.000000000000 | 0.000000000000 |
| ENSG00000138182 | 11.93328 | 4.669056 | 1.353788 | 0.000000000000 | 0.000000000000 |
| ENSG00000057663 | 9.324044 | 4.349873 | 1.099983 | 0.000000000000 | 0.000000000000 |
| ENSG00000105246 | 6.903797 | 17.02231 | -1.30196 | 0.000000000000 | 0.000000000000 |
| ENSG00000186847 | 3.130322 | 8.898859 | -1.50731 | 0.000000000000 | 0.000000000000 |
| ENSG00000260772 | 6.898139 | 1.849463 | 1.899101 | 0.000000000000 | 0.000000000000 |
| ENSG00000279821 | 2.548664 | 6.564902 | -1.36503 | 0.000000000000 | 0.000000000000 |
| ENSG00000280407 | 6.695593 | 2.744271 | 1.286789 | 0.000000000000 | 0.000000000000 |
| ENSG00000138399 | 11.66756 | 5.609192 | 1.056638 | 0.000000000000 | 0.000000000000 |
| ENSG00000134070 | 3.330148 | 7.420543 | -1.15594 | 0.000000000000 | 0.000000000000 |
| ENSG00000162894 | 0.547955 | 8.205155 | -3.9044 | 0.000000000000 | 0.000000000000 |
| ENSG00000220793 | 46.68312 | 21.3555 | 1.128294 | 0.000000000000 | 0.000000000000 |
| ENSG00000251259 | 9.065315 | 0.0001 | 16.46807 | 0.000000000000 | 0.000000000000 |
| ENSG00000210176 | 306.9093 | 136.4086 | 1.169877 | 0.000000000000 | 0.000000000000 |
| ENSG00000266711 | 3.210518 | 16.48018 | -2.35985 | 0.000000000000 | 0.000000000000 |
| ENSG00000168078 | 25.90718 | 11.41051 | 1.182989 | 0.000000000000 | 0.000000000000 |
| ENSG00000268592 | 20.36112 | 9.853696 | 1.04708 | 0.000000000000 | 0.000000000000 |
| ENSG00000158234 | 22.43945 | 8.934488 | 1.32858 | 0.000000000000 | 0.000000000000 |
| ENSG00000101928 | 13.52042 | 6.289828 | 1.104047 | 0.000000000000 | 0.000000000000 |
| ENSG00000178700 | 2.856678 | 5.764727 | -1.01291 | 0.000000000000 | 0.000000000000 |
| ENSG00000215196 | 7.585446 | 3.540517 | 1.099274 | 0.000000000000 | 0.000000000000 |
| ENSG00000115514 | 15.10033 | 6.199316 | 1.284399 | 0.000000000000 | 0.000000000000 |
| ENSG00000267922 | 4.801218 | 25.4126 | -2.40407 | 0.000000000000 | 0.000000000000 |
| ENSG00000145725 | 6.45032 | 2.114451 | 1.609088 | 0.000000000000 | 0.000000000000 |
| ENSG00000213780 | 39.16436 | 85.57123 | -1.12758 | 0.000000000000 | 0.000000000000 |
| ENSG00000188293 | 0.882219 | 7.930596 | -3.16822 | 0.000000000000 | 0.000000000000 |
| ENSG00000189143 | 2.125629 | 6.120054 | -1.52565 | 0.000000000000 | 0.000000000000 |
| ENSG00000233493 | 17.83644 | 7.550856 | 1.240116 | 0.000000000000 | 0.000000000000 |
| ENSG00000228172 | 5.04032 | 2.008988 | 1.327046 | 0.000000000000 | 0.000000000000 |
| ENSG00000173875 | 3.144983 | 6.884838 | -1.13037 | 0.000000000000 | 0.000000000000 |
| ENSG00000273221 | 7.013629 | 0.0001 | 16.09787 | 0.000000000000 | 0.000000000000 |
| ENSG00000041357 | 105.6023 | 48.90033 | 1.110725 | 0.000000000000 | 0.000000000000 |
| ENSG00000276980 | 4.451735 | 12.05003 | -1.4366 | 0.000000000000 | 0.000000000000 |
| ENSG00000255198 | 66.07108 | 28.56519 | 1.209761 | 0.000000000000 | 0.000000000000 |
| ENSG00000005469 | 5.56431 | 2.249084 | 1.306865 | 0.000000000000 | 0.000000000000 |
| ENSG00000198160 | 14.21801 | 6.313188 | 1.171279 | 0.000000000000 | 0.000000000000 |
| ENSG00000227039 | 2.454772 | 6.795229 | -1.46893 | 0.000000000000 | 0.000000000000 |
| ENSG00000258315 | 16.71951 | 4.643372 | 1.848287 | 0.000000000000 | 0.000000000000 |
| ENSG00000067955 | 41.89469 | 13.57401 | 1.625921 | 0.000000000000 | 0.000000000000 |
| ENSG00000239470 | 12.50266 | 1.79949 | 2.796575 | 0.000000000000 | 0.000000000000 |
| ENSG00000244313 | 43.10175 | 20.64646 | 1.061852 | 0.000000000000 | 0.000000000000 |
| ENSG00000157426 | 9.756977 | 4.56564 | 1.095617 | 0.000000000000 | 0.000000000000 |
| ENSG00000129422 | 28.99848 | 14.14674 | 1.035507 | 0.000000000000 | 0.000000000000 |
| ENSG00000079337 | 3.66061 | 9.708027 | -1.40709 | 0.000000000000 | 0.000000000000 |
| ENSG00000133816 | 4.361292 | 11.83 | -1.43962 | 0.000000000000 | 0.000000000000 |
| ENSG00000148019 | 12.07522 | 5.787356 | 1.061073 | 0.000000000000 | 0.000000000000 |
| ENSG00000152056 | 6.657026 | 1.943735 | 1.776046 | 0.000000000000 | 0.000000000000 |
| ENSG00000135083 | 2.473018 | 7.641281 | -1.62754 | 0.000000000000 | 0.000000000000 |
| ENSG00000087903 | 11.06734 | 24.35487 | -1.1379 | 0.000000000000 | 0.000000000000 |
| ENSG00000156162 | 9.254557 | 3.831795 | 1.272144 | 0.000000000000 | 0.000000000000 |
| ENSG00000225345 | 5.866694 | 0.647332 | 3.17997 | 0.000000000000 | 0.000000000000 |
| ENSG00000139926 | 3.077285 | 9.535909 | -1.63171 | 0.000000000000 | 0.000000000000 |
| ENSG00000140044 | 2.421064 | 13.32521 | -2.46044 | 0.000000000000 | 0.000000000000 |
| ENSG00000087299 | 10.9572 | 3.915025 | 1.484786 | 0.000000000000 | 0.000000000000 |
| ENSG00000185664 | 6.886831 | 1.557591 | 2.144524 | 0.000000000000 | 0.000000000000 |
| ENSG00000238103 | 26.18961 | 10.82774 | 1.274263 | 0.000000000000 | 0.000000000000 |
| ENSG00000169203 | 12.0617 | 25.30586 | -1.06904 | 0.000000000000 | 0.000000000000 |
| ENSG00000137269 | 5.744483 | 15.97227 | -1.47532 | 0.000000000000 | 0.000000000000 |
| ENSG00000198835 | 3.800741 | 8.719253 | -1.19792 | 0.000000000000 | 0.000000000000 |
| ENSG00000213178 | 27.34181 | 9.251516 | 1.563347 | 0.000000000000 | 0.000000000000 |
| ENSG00000164975 | 17.82846 | 7.096884 | 1.328925 | 0.000000000000 | 0.000000000000 |
| ENSG00000258643 | 11.14514 | 3.622798 | 1.621238 | 0.000000000000 | 0.000000000000 |
| ENSG00000104892 | 5.829432 | 13.78461 | -1.24163 | 0.000000000000 | 0.000000000000 |
| ENSG00000173744 | 30.27902 | 76.87247 | -1.34415 | 0.000000000000 | 0.000000000000 |
| ENSG00000162913 | 8.895361 | 4.090527 | 1.120766 | 0.000000000000 | 0.000000000000 |
| ENSG00000173846 | 5.229921 | 11.99293 | -1.19732 | 0.000000000000 | 0.000000000000 |
| ENSG00000249007 | 12.01834 | 4.281701 | 1.488982 | 0.000000000000 | 0.000000000000 |
| ENSG00000072849 | 52.98711 | 26.38384 | 1.005987 | 0.000000000000 | 0.000000000000 |
| ENSG00000253738 | 5.695763 | 2.365417 | 1.267795 | 0.000000000000 | 0.000000000000 |
| ENSG00000144810 | 8.47 | 18.99717 | -1.16535 | 0.000000000000 | 0.000000000000 |
| ENSG00000116701 | 7.402899 | 14.93615 | -1.01265 | 0.000000000000 | 0.000000000000 |
| ENSG00000230606 | 8.039764 | 3.238047 | 1.312029 | 0.000000000000 | 0.000000000000 |
| ENSG00000155324 | 10.16409 | 21.2971 | -1.06718 | 0.000000000000 | 0.000000000000 |
| ENSG00000113851 | 38.41774 | 14.02758 | 1.453507 | 0.000000000000 | 0.000000000000 |
| ENSG00000143398 | 55.70304 | 126.7732 | -1.18642 | 0.000000000000 | 0.000000000000 |
| ENSG00000002834 | 83.16226 | 197.6291 | -1.24879 | 0.000000000000 | 0.000000000000 |
| ENSG00000155066 | 6.284703 | 2.102012 | 1.580074 | 0.000000000000 | 0.000000000000 |
| ENSG00000189050 | 20.71028 | 6.409154 | 1.692141 | 0.000000000000 | 0.000000000000 |
| ENSG00000121753 | 2.608271 | 6.948603 | -1.41363 | 0.000000000000 | 0.000000000000 |
| ENSG00000163006 | 8.061496 | 3.627654 | 1.152011 | 0.000000000000 | 0.000000000000 |
| ENSG00000133121 | 2.676569 | 6.904871 | -1.36723 | 0.000000000000 | 0.000000000000 |
| ENSG00000114107 | 12.9624 | 5.990731 | 1.113529 | 0.000000000000 | 0.000000000000 |
| ENSG00000167701 | 9.286328 | 3.677182 | 1.336508 | 0.000000000000 | 0.000000000000 |
| ENSG00000198162 | 11.74001 | 3.604065 | 1.703737 | 0.000000000000 | 0.000000000000 |
| ENSG00000137692 | 26.35132 | 12.19496 | 1.11159 | 0.000000000000 | 0.000000000000 |
| ENSG00000182841 | 18.78173 | 50.12208 | -1.41612 | 0.000000000000 | 0.000000000000 |
| ENSG00000175105 | 5.84533 | 2.453033 | 1.252718 | 0.000000000000 | 0.000000000000 |
| ENSG00000138801 | 75.5626 | 29.19992 | 1.371708 | 0.000000000000 | 0.000000000000 |
| ENSG00000231305 | 11.87351 | 4.11621 | 1.528358 | 0.000000000000 | 0.000000000000 |
| ENSG00000200795 | 25.02162 | 0.0001 | 17.93282 | 0.000000000000 | 0.000000000000 |
| ENSG00000270087 | 6.979473 | 0.0001 | 16.09083 | 0.000000000000 | 0.000000000000 |
| ENSG00000213860 | 12.3534 | 2.375226 | 2.378772 | 0.000000000000 | 0.000000000000 |
| ENSG00000257184 | 6.041887 | 0.724216 | 3.060507 | 0.000000000000 | 0.000000000000 |
| ENSG00000272601 | 8.429969 | 0.587227 | 3.843537 | 0.000000000000 | 0.000000000000 |
| ENSG00000239523 | 5.602302 | 16.3062 | -1.54133 | 0.000000000000 | 0.000000000000 |
| ENSG00000247240 | 5.775959 | 2.650153 | 1.123985 | 0.000000000000 | 0.000000000000 |
| ENSG00000125740 | 5.579677 | 2.723072 | 1.034946 | 0.000000000000 | 0.000000000000 |
| ENSG00000244021 | 32.01985 | 14.51081 | 1.141838 | 0.000000000000 | 0.000000000000 |
| ENSG00000279806 | 10.54803 | 5.206684 | 1.018537 | 0.000000000000 | 0.000000000000 |
| ENSG00000197258 | 9.707894 | 4.747367 | 1.032031 | 0.000000000000 | 0.000000000000 |
| ENSG00000083123 | 11.58708 | 5.786042 | 1.001868 | 0.000000000000 | 0.000000000000 |
| ENSG00000266401 | 1.515603 | 5.69095 | -1.90878 | 0.000000000000 | 0.000000000000 |
| ENSG00000123607 | 16.60119 | 7.40406 | 1.164898 | 0.000000000000 | 0.000000000000 |
| ENSG00000110203 | 1.749204 | 9.071077 | -2.37458 | 0.000000000000 | 0.000000000000 |
| ENSG00000205309 | 8.231311 | 3.345147 | 1.299053 | 0.000000000000 | 0.000000000000 |
| ENSG00000145439 | 5.546796 | 2.090754 | 1.407631 | 0.000000000000 | 0.000000000000 |
| ENSG00000188010 | 12.71031 | 4.353026 | 1.545908 | 0.000000000000 | 0.000000000000 |
| ENSG00000189223 | 6.356928 | 15.11404 | -1.24949 | 0.000000000000 | 0.000000000000 |
| ENSG00000156804 | 4.712052 | 13.48114 | -1.51651 | 0.000000000000 | 0.000000000000 |
| ENSG00000138650 | 6.289527 | 2.59962 | 1.274651 | 0.000000000000 | 0.000000000000 |
| ENSG00000069667 | 2.757787 | 6.04735 | -1.13279 | 0.000000000000 | 0.000000000000 |
| ENSG00000128298 | 3.692481 | 8.126961 | -1.13813 | 0.000000000000 | 0.000000000000 |
| ENSG00000124208 | 3.841499 | 7.731574 | -1.00909 | 0.000000000000 | 0.000000000000 |
| ENSG00000272335 | 5.092083 | 2.170429 | 1.230276 | 0.000000000000 | 0.000000000000 |
| ENSG00000198839 | 10.56569 | 3.587011 | 1.558533 | 0.000000000000 | 0.000000000000 |
| ENSG00000265752 | 7.689348 | 2.41921 | 1.668325 | 0.000000000000 | 0.000000000000 |
| ENSG00000234684 | 4.132288 | 9.213378 | -1.15679 | 0.000000000000 | 0.000000000000 |
| ENSG00000128710 | 6.048402 | 2.724718 | 1.150447 | 0.000000000000 | 0.000000000000 |
| ENSG00000223749 | 4.216588 | 15.0557 | -1.83616 | 0.000000000000 | 0.000000000000 |
| ENSG00000200463 | 22.02967 | 0.413822 | 5.734294 | 0.000000000000 | 0.000000000000 |
| ENSG00000114744 | 22.48841 | 10.19179 | 1.141774 | 0.000000000000 | 0.000000000000 |
| ENSG00000279117 | 5.094016 | 2.141529 | 1.250162 | 0.000000000000 | 0.000000000000 |
| ENSG00000005102 | 13.41238 | 5.861109 | 1.194319 | 0.000000000000 | 0.000000000000 |
| ENSG00000141338 | 6.424245 | 2.269588 | 1.501096 | 0.000000000000 | 0.000000000000 |
| ENSG00000231721 | 4.40085 | 11.77128 | -1.41942 | 0.000000000000 | 0.000000000000 |
| ENSG00000004766 | 30.3349 | 10.46178 | 1.535849 | 0.000000000000 | 0.000000000000 |
| ENSG00000267261 | 8.548162 | 2.916716 | 1.551269 | 0.000000000000 | 0.000000000000 |
| ENSG00000147996 | 12.61628 | 26.03231 | -1.04502 | 0.000000000000 | 0.000000000000 |
| ENSG00000274602 | 3.081714 | 7.648781 | -1.3115 | 0.000000000000 | 0.000000000000 |
| ENSG00000086200 | 31.06835 | 12.54952 | 1.307814 | 0.000000000000 | 0.000000000000 |
| ENSG00000163701 | 5.312628 | 2.225413 | 1.255353 | 0.000000000000 | 0.000000000000 |
| ENSG00000136147 | 11.32931 | 5.126069 | 1.144135 | 0.000000000000 | 0.000000000000 |
| ENSG00000254501 | 7.099144 | 0.832276 | 3.092511 | 0.000000000000 | 0.000000000000 |
| ENSG00000182771 | 3.662437 | 7.55764 | -1.04513 | 0.000000000000 | 0.000000000000 |
| ENSG00000142677 | 3.032571 | 6.307595 | -1.05655 | 0.000000000000 | 0.000000000000 |
| ENSG00000146592 | 2.131747 | 6.429279 | -1.59262 | 0.000000000000 | 0.000000000001 |
| ENSG00000113597 | 9.612766 | 4.100664 | 1.229094 | 0.000000000000 | 0.000000000001 |
| ENSG00000207513 | 38.70692 | 10.76639 | 1.846057 | 0.000000000000 | 0.000000000001 |
| ENSG00000171084 | 3.585779 | 7.743074 | -1.11062 | 0.000000000000 | 0.000000000002 |
| ENSG00000134533 | 2.984942 | 7.295405 | -1.28929 | 0.000000000000 | 0.000000000002 |
| ENSG00000207005 | 38.46573 | 10.73495 | 1.841258 | 0.000000000000 | 0.000000000002 |
| ENSG00000171658 | 3.322799 | 10.49249 | -1.65889 | 0.000000000000 | 0.000000000002 |
| ENSG00000214856 | 5.371001 | 11.86527 | -1.14348 | 0.000000000000 | 0.000000000002 |
| ENSG00000231006 | 11.00437 | 3.931548 | 1.484907 | 0.000000000001 | 0.000000000003 |
| ENSG00000237424 | 2.161845 | 5.057785 | -1.22624 | 0.000000000001 | 0.000000000005 |
| ENSG00000250474 | 1.71604 | 6.391468 | -1.89706 | 0.000000000001 | 0.000000000006 |
| ENSG00000244041 | 3.357777 | 8.0849 | -1.26772 | 0.000000000001 | 0.000000000006 |
| ENSG00000228873 | 19.92994 | 3.091345 | 2.68863 | 0.000000000001 | 0.000000000006 |
| ENSG00000223478 | 23.10361 | 10.88667 | 1.085555 | 0.000000000001 | 0.000000000006 |
| ENSG00000230408 | 10.22304 | 3.935106 | 1.37735 | 0.000000000001 | 0.000000000007 |
| ENSG00000278774 | 22.51253 | 4.189622 | 2.425836 | 0.000000000001 | 0.000000000008 |
| ENSG00000226332 | 11.68959 | 23.95408 | -1.03505 | 0.000000000002 | 0.000000000010 |
| ENSG00000283378 | 5.233351 | 2.104565 | 1.314213 | 0.000000000003 | 0.000000000016 |
| ENSG00000066583 | 33.20302 | 13.73801 | 1.273142 | 0.000000000003 | 0.000000000017 |
| ENSG00000266992 | 6.037678 | 2.315274 | 1.382811 | 0.000000000004 | 0.000000000021 |
| ENSG00000258056 | 5.475363 | 11.10449 | -1.02012 | 0.000000000004 | 0.000000000023 |
| ENSG00000125246 | 5.388276 | 1.71028 | 1.655591 | 0.000000000005 | 0.000000000024 |
| ENSG00000185010 | 6.217755 | 2.845805 | 1.127557 | 0.000000000006 | 0.000000000031 |
| ENSG00000147687 | 11.58603 | 5.191864 | 1.158062 | 0.000000000006 | 0.000000000031 |
| ENSG00000210049 | 129.6104 | 52.64635 | 1.299776 | 0.000000000006 | 0.000000000031 |
| ENSG00000231686 | 5.285429 | 12.06623 | -1.19088 | 0.000000000009 | 0.000000000049 |
| ENSG00000265096 | 7.725533 | 16.75563 | -1.11694 | 0.000000000013 | 0.000000000068 |
| ENSG00000006606 | 10.27692 | 4.403841 | 1.222573 | 0.000000000013 | 0.000000000069 |
| ENSG00000101624 | 16.72581 | 7.73885 | 1.111885 | 0.000000000014 | 0.000000000071 |
| ENSG00000140682 | 2.189657 | 5.624534 | -1.36103 | 0.000000000014 | 0.000000000072 |
| ENSG00000183153 | 7.487411 | 2.52278 | 1.569453 | 0.000000000014 | 0.000000000073 |
| ENSG00000254416 | 5.972736 | 1.158468 | 2.366174 | 0.000000000015 | 0.000000000077 |
| ENSG00000134470 | 3.364795 | 8.67068 | -1.36563 | 0.000000000016 | 0.000000000081 |
| ENSG00000205871 | 6.756793 | 1.896505 | 1.832995 | 0.000000000025 | 0.000000000130 |
| ENSG00000254685 | 6.523109 | 2.933147 | 1.15311 | 0.000000000029 | 0.000000000147 |
| ENSG00000272288 | 18.0703 | 6.420415 | 1.492882 | 0.000000000032 | 0.000000000162 |
| ENSG00000230629 | 23.72417 | 9.31902 | 1.348107 | 0.000000000042 | 0.000000000215 |
| ENSG00000178429 | 8.429705 | 3.069077 | 1.457677 | 0.000000000043 | 0.000000000217 |
| ENSG00000070961 | 42.93575 | 21.39021 | 1.005229 | 0.000000000047 | 0.000000000238 |
| ENSG00000092020 | 6.335446 | 2.168191 | 1.546954 | 0.000000000071 | 0.000000000354 |
| ENSG00000198417 | 3.039379 | 9.482778 | -1.64153 | 0.000000000078 | 0.000000000388 |
| ENSG00000139974 | 5.462656 | 2.135543 | 1.355 | 0.000000000083 | 0.000000000412 |
| ENSG00000159556 | 5.345253 | 11.34273 | -1.08544 | 0.000000000088 | 0.000000000436 |
| ENSG00000085788 | 26.04264 | 11.02146 | 1.240561 | 0.000000000092 | 0.000000000454 |
| ENSG00000212664 | 17.09427 | 7.657707 | 1.158528 | 0.000000000095 | 0.000000000471 |
| ENSG00000241288 | 1.474141 | 6.470905 | -2.13409 | 0.000000000101 | 0.000000000501 |
| ENSG00000185437 | 7.287851 | 2.158583 | 1.755409 | 0.000000000114 | 0.000000000562 |
| ENSG00000071967 | 4.912742 | 16.02399 | -1.70563 | 0.000000000115 | 0.000000000567 |
| ENSG00000282907 | 5.701432 | 13.19027 | -1.21008 | 0.000000000147 | 0.000000000717 |
| ENSG00000240682 | 21.62542 | 49.97912 | -1.2086 | 0.000000000195 | 0.000000000947 |
| ENSG00000134287 | 78.09964 | 220.5344 | -1.49762 | 0.000000000219 | 0.000000001059 |
| ENSG00000119314 | 29.54495 | 14.26201 | 1.050735 | 0.000000000237 | 0.000000001142 |
| ENSG00000101974 | 6.405258 | 1.642737 | 1.963155 | 0.000000000316 | 0.000000001507 |
| ENSG00000257261 | 1.306551 | 7.389573 | -2.49973 | 0.000000000343 | 0.000000001630 |
| ENSG00000277459 | 8.471076 | 18.19439 | -1.10288 | 0.000000000350 | 0.000000001663 |
| ENSG00000227403 | 10.91428 | 4.642375 | 1.233282 | 0.000000000432 | 0.000000002044 |
| ENSG00000218227 | 5.401725 | 13.77531 | -1.35059 | 0.000000000631 | 0.000000002956 |
| ENSG00000072954 | 2.63928 | 5.355286 | -1.02082 | 0.000000000693 | 0.000000003240 |
| ENSG00000281991 | 2.155802 | 6.416695 | -1.57361 | 0.000000000721 | 0.000000003369 |
| ENSG00000100558 | 5.139154 | 10.68423 | -1.05588 | 0.000000000743 | 0.000000003466 |
| ENSG00000047579 | 27.54139 | 59.90912 | -1.12117 | 0.000000000832 | 0.000000003865 |
| ENSG00000206585 | 25.33263 | 6.664264 | 1.926479 | 0.000000000863 | 0.000000004002 |
| ENSG00000168228 | 3.858263 | 8.181206 | -1.08436 | 0.000000000870 | 0.000000004034 |
| ENSG00000230795 | 1.776237 | 5.614767 | -1.6604 | 0.000000001009 | 0.000000004662 |
| ENSG00000224546 | 5.173454 | 2.491296 | 1.054231 | 0.000000001073 | 0.000000004946 |
| ENSG00000108771 | 5.099013 | 10.30652 | -1.01527 | 0.000000001266 | 0.000000005811 |
| ENSG00000155111 | 7.156885 | 2.999806 | 1.254463 | 0.000000001301 | 0.000000005963 |
| ENSG00000109911 | 13.33901 | 4.447105 | 1.584714 | 0.000000001338 | 0.000000006130 |
| ENSG00000143554 | 8.575445 | 3.974656 | 1.109382 | 0.000000001351 | 0.000000006187 |
| ENSG00000261089 | 1.684747 | 5.427768 | -1.68783 | 0.000000001418 | 0.000000006468 |
| ENSG00000105717 | 4.166813 | 8.509734 | -1.03017 | 0.000000001422 | 0.000000006487 |
| ENSG00000263776 | 25.38893 | 5.672505 | 2.162142 | 0.000000001549 | 0.000000007052 |
| ENSG00000138376 | 7.690441 | 3.806322 | 1.014669 | 0.000000002110 | 0.000000009550 |
| ENSG00000081019 | 9.276406 | 3.243674 | 1.515937 | 0.000000002202 | 0.000000009959 |
| ENSG00000276496 | 17.47441 | 0.337197 | 5.695508 | 0.000000002611 | 0.000000011728 |
| ENSG00000155622 | 4.21902 | 11.20168 | -1.40874 | 0.000000002637 | 0.000000011840 |
| ENSG00000116815 | 8.027303 | 3.752207 | 1.097176 | 0.000000003078 | 0.000000013763 |
| ENSG00000259884 | 8.38905 | 2.533298 | 1.727491 | 0.000000003438 | 0.000000015325 |
| ENSG00000168298 | 3.881181 | 9.788504 | -1.33459 | 0.000000004088 | 0.000000018081 |
| ENSG00000148346 | 3.608727 | 9.854642 | -1.44931 | 0.000000004886 | 0.000000021495 |
| ENSG00000251141 | 12.22977 | 2.680649 | 2.189743 | 0.000000005709 | 0.000000024994 |
| ENSG00000185480 | 8.291643 | 4.045255 | 1.035427 | 0.000000005750 | 0.000000025163 |
| ENSG00000269888 | 23.35609 | 8.70497 | 1.423888 | 0.000000006233 | 0.000000027199 |
| ENSG00000138613 | 6.431819 | 2.848522 | 1.175013 | 0.000000006591 | 0.000000028682 |
| ENSG00000277998 | 5.978623 | 0.457121 | 3.709165 | 0.000000007481 | 0.000000032421 |
| ENSG00000278921 | 5.614638 | 2.069931 | 1.43961 | 0.000000008425 | 0.000000036398 |
| ENSG00000204778 | 6.390436 | 1.853659 | 1.785538 | 0.000000009394 | 0.000000040395 |
| ENSG00000273768 | 25.32631 | 7.345079 | 1.785787 | 0.000000009394 | 0.000000040395 |
| ENSG00000261824 | 3.988253 | 10.50855 | -1.39773 | 0.000000009430 | 0.000000040532 |
| ENSG00000185838 | 4.781179 | 15.26697 | -1.67498 | 0.000000010485 | 0.000000044890 |
| ENSG00000148655 | 9.07079 | 3.965035 | 1.193895 | 0.000000011191 | 0.000000047778 |
| ENSG00000206737 | 24.62433 | 7.031068 | 1.808269 | 0.000000011937 | 0.000000050866 |
| ENSG00000268995 | 5.060506 | 0.331936 | 3.930305 | 0.000000014521 | 0.000000061461 |
| ENSG00000155542 | 9.105632 | 3.830039 | 1.2494 | 0.000000015491 | 0.000000065482 |
| ENSG00000171100 | 9.761496 | 3.866167 | 1.336198 | 0.000000017404 | 0.000000073241 |
| ENSG00000187185 | 3.164171 | 7.670444 | -1.27748 | 0.000000018282 | 0.000000076823 |
| ENSG00000263934 | 33.61927 | 15.27275 | 1.138329 | 0.000000018892 | 0.000000079272 |
| ENSG00000243885 | 1.982617 | 5.641873 | -1.50877 | 0.000000019207 | 0.000000080549 |
| ENSG00000162994 | 8.16992 | 3.249904 | 1.329925 | 0.000000019517 | 0.000000081758 |
| ENSG00000228638 | 7.043998 | 2.215255 | 1.668922 | 0.000000019674 | 0.000000082386 |
| ENSG00000059915 | 6.232264 | 2.699056 | 1.207301 | 0.000000019722 | 0.000000082572 |
| ENSG00000128891 | 16.00006 | 6.961604 | 1.200585 | 0.000000021441 | 0.000000089511 |
| ENSG00000236534 | 17.5471 | 7.812251 | 1.167422 | 0.000000021533 | 0.000000089844 |
| ENSG00000179387 | 6.708862 | 3.274977 | 1.034583 | 0.000000022352 | 0.000000093209 |
| ENSG00000269834 | 2.395724 | 5.153096 | -1.10498 | 0.000000023798 | 0.000000099041 |
| ENSG00000266933 | 10.30342 | 5.129048 | 1.006361 | 0.000000024316 | 0.000000101141 |
| ENSG00000154646 | 5.070404 | 2.360194 | 1.103195 | 0.000000028726 | 0.000000119077 |
| ENSG00000221381 | 0.268058 | 17.27634 | -6.01011 | 0.000000030678 | 0.000000126892 |
| ENSG00000197385 | 3.654538 | 7.605809 | -1.05741 | 0.000000043245 | 0.000000176643 |
| ENSG00000088451 | 6.075656 | 2.498567 | 1.281939 | 0.000000045451 | 0.000000185525 |
| ENSG00000271810 | 1.411612 | 5.061922 | -1.84234 | 0.000000046012 | 0.000000187648 |
| ENSG00000272831 | 5.468837 | 1.278741 | 2.09651 | 0.000000050159 | 0.000000203799 |
| ENSG00000261159 | 8.775291 | 4.089236 | 1.101616 | 0.000000059696 | 0.000000241160 |
| ENSG00000163738 | 7.438244 | 3.659946 | 1.02314 | 0.000000065809 | 0.000000264992 |
| ENSG00000249641 | 2.602528 | 5.748573 | -1.14329 | 0.000000074010 | 0.000000296663 |
| ENSG00000229931 | 2.99646 | 8.739803 | -1.54434 | 0.000000084144 | 0.000000335524 |
| ENSG00000228206 | 1.00463 | 7.450386 | -2.89065 | 0.000000090303 | 0.000000358836 |
| ENSG00000103811 | 6.327306 | 3.078784 | 1.039231 | 0.000000095533 | 0.000000379093 |
| ENSG00000234353 | 2.732877 | 6.844221 | -1.32447 | 0.000000108286 | 0.000000427921 |
| ENSG00000257337 | 13.92993 | 4.694623 | 1.569107 | 0.000000108631 | 0.000000429210 |
| ENSG00000165949 | 2.575152 | 7.014045 | -1.44559 | 0.000000114995 | 0.000000453341 |
| ENSG00000261353 | 5.127094 | 0.483521 | 3.406491 | 0.000000125542 | 0.000000492973 |
| ENSG00000227329 | 8.111075 | 0.275277 | 4.880937 | 0.000000128602 | 0.000000504037 |
| ENSG00000206652 | 19.6412 | 5.42847 | 1.855265 | 0.000000132603 | 0.000000519278 |
| ENSG00000237595 | 2.121899 | 6.187731 | -1.54405 | 0.000000181740 | 0.000000702468 |
| ENSG00000156475 | 3.441122 | 8.521544 | -1.30824 | 0.000000224276 | 0.000000861652 |
| ENSG00000198353 | 5.168902 | 2.350395 | 1.136955 | 0.000000234566 | 0.000000899528 |
| ENSG00000253368 | 3.753495 | 8.647322 | -1.20402 | 0.000000244823 | 0.000000937765 |
| ENSG00000264346 | 10.54075 | 0.119224 | 6.466159 | 0.000000248530 | 0.000000951728 |
| ENSG00000207145 | 9.784752 | 0.26651 | 5.198274 | 0.000000248530 | 0.000000951728 |
| ENSG00000253187 | 3.814288 | 8.251082 | -1.11317 | 0.000000320710 | 0.000001218677 |
| ENSG00000232456 | 1.576715 | 5.346038 | -1.76155 | 0.000000344347 | 0.000001306117 |
| ENSG00000272473 | 2.892751 | 7.031042 | -1.2813 | 0.000000390294 | 0.000001474666 |
| ENSG00000273443 | 4.319702 | 9.278742 | -1.103 | 0.000000443878 | 0.000001668742 |
| ENSG00000200418 | 13.22041 | 1.51783 | 3.122685 | 0.000000459374 | 0.000001725867 |
| ENSG00000207389 | 18.2761 | 5.092112 | 1.843623 | 0.000000467168 | 0.000001753428 |
| ENSG00000200624 | 16.78407 | 2.566618 | 2.709152 | 0.000000475689 | 0.000001784242 |
| ENSG00000276170 | 1.769629 | 6.85889 | -1.95453 | 0.000000511196 | 0.000001914605 |
| ENSG00000210156 | 38.87694 | 9.454102 | 2.039902 | 0.000000529822 | 0.000001982427 |
| ENSG00000182327 | 5.346652 | 2.363617 | 1.177639 | 0.000000700021 | 0.000002591365 |
| ENSG00000259316 | 19.64799 | 40.15578 | -1.03123 | 0.000000728715 | 0.000002689773 |
| ENSG00000121769 | 1.210592 | 5.088786 | -2.07161 | 0.000000933388 | 0.000003419121 |
| ENSG00000122435 | 9.413114 | 2.779017 | 1.760097 | 0.000001159599 | 0.000004207122 |
| ENSG00000251580 | 7.701867 | 2.484292 | 1.632373 | 0.000001285687 | 0.000004646944 |
| ENSG00000262831 | 6.494785 | 2.251375 | 1.528475 | 0.000001499331 | 0.000005380147 |
| ENSG00000204538 | 2.338245 | 5.299008 | -1.1803 | 0.000001502193 | 0.000005389152 |
| ENSG00000237864 | 1.648954 | 5.096415 | -1.62793 | 0.000001646014 | 0.000005886702 |
| ENSG00000214401 | 2.103165 | 6.832972 | -1.69995 | 0.000001646014 | 0.000005886702 |
| ENSG00000242028 | 34.45461 | 12.73907 | 1.435438 | 0.000001823618 | 0.000006490009 |
| ENSG00000212304 | 12.36983 | 0.122066 | 6.663021 | 0.000001824499 | 0.000006492137 |
| ENSG00000210077 | 80.80602 | 37.75095 | 1.09795 | 0.000001966358 | 0.000006959143 |
| ENSG00000215375 | 20.73937 | 9.298038 | 1.157373 | 0.000002285825 | 0.000008058692 |
| ENSG00000267769 | 7.453463 | 2.767907 | 1.429115 | 0.000002298678 | 0.000008100271 |
| ENSG00000053770 | 6.950406 | 3.339878 | 1.057302 | 0.000002304849 | 0.000008119523 |
| ENSG00000111348 | 1.878029 | 5.592893 | -1.57438 | 0.000002399910 | 0.000008433686 |
| ENSG00000255513 | 14.36506 | 6.430089 | 1.159653 | 0.000002412013 | 0.000008473623 |
| ENSG00000256591 | 5.672279 | 12.57109 | -1.14811 | 0.000002544127 | 0.000008924088 |
| ENSG00000101076 | 6.966496 | 2.313282 | 1.590492 | 0.000002752879 | 0.000009622496 |
| ENSG00000117569 | 7.534044 | 2.736989 | 1.460835 | 0.000002788320 | 0.000009741925 |
| ENSG00000232995 | 11.56702 | 4.199763 | 1.461638 | 0.000002991745 | 0.000010417758 |
| ENSG00000135931 | 8.402138 | 26.42094 | -1.65285 | 0.000003189991 | 0.000011079495 |
| ENSG00000186001 | 13.3457 | 30.83314 | -1.20811 | 0.000003257086 | 0.000011300558 |
| ENSG00000223975 | 15.80767 | 6.674026 | 1.243995 | 0.000003350145 | 0.000011604124 |
| ENSG00000259485 | 5.320228 | 1.414917 | 1.910771 | 0.000003476151 | 0.000012026055 |
| ENSG00000186395 | 61.54594 | 19.78256 | 1.637435 | 0.000003767353 | 0.000012990404 |
| ENSG00000262888 | 3.064101 | 7.123475 | -1.21712 | 0.000003900230 | 0.000013432435 |
| ENSG00000156463 | 4.711085 | 10.67283 | -1.17981 | 0.000004118472 | 0.000014158558 |
| ENSG00000266402 | 24.31622 | 7.383738 | 1.719496 | 0.000004525195 | 0.000015504527 |
| ENSG00000267697 | 0.151407 | 6.378196 | -5.39664 | 0.000004527894 | 0.000015509142 |
| ENSG00000201129 | 0.056684 | 8.343835 | -7.20163 | 0.000004527894 | 0.000015509142 |
| ENSG00000234160 | 10.46021 | 22.21716 | -1.08676 | 0.000005386604 | 0.000018347720 |
| ENSG00000174500 | 1.068634 | 5.622062 | -2.39533 | 0.000005628342 | 0.000019131359 |
| ENSG00000166669 | 5.709823 | 2.16557 | 1.398699 | 0.000005804007 | 0.000019675996 |
| ENSG00000160678 | 6.468075 | 3.124248 | 1.049827 | 0.000005845006 | 0.000019809131 |
| ENSG00000228626 | 1.899094 | 8.09058 | -2.09093 | 0.000006688097 | 0.000022526673 |
| ENSG00000273143 | 2.090835 | 8.717273 | -2.0598 | 0.000006771889 | 0.000022802205 |
| ENSG00000248774 | 7.265583 | 2.87882 | 1.335601 | 0.000007243585 | 0.000024311997 |
| ENSG00000206596 | 16.82542 | 5.314462 | 1.662647 | 0.000007367764 | 0.000024705295 |
| ENSG00000206588 | 16.78253 | 5.356251 | 1.647665 | 0.000007367764 | 0.000024705295 |
| ENSG00000083312 | 41.97363 | 93.04951 | -1.14852 | 0.000009114119 | 0.000030266949 |
| ENSG00000176383 | 5.100317 | 10.97362 | -1.10538 | 0.000009227546 | 0.000030621466 |
| ENSG00000124140 | 6.783002 | 2.92399 | 1.213986 | 0.000010535767 | 0.000034721724 |
| ENSG00000283537 | 4.345136 | 9.225619 | -1.08624 | 0.000011666526 | 0.000038212245 |
| ENSG00000157224 | 16.30028 | 33.13702 | -1.02355 | 0.000011800408 | 0.000038634213 |
| ENSG00000261801 | 1.638091 | 5.141551 | -1.65019 | 0.000011982190 | 0.000039190215 |
| ENSG00000070367 | 3.134456 | 8.351699 | -1.41385 | 0.000013333804 | 0.000043308436 |
| ENSG00000234219 | 3.068153 | 7.112116 | -1.21291 | 0.000013766722 | 0.000044626120 |
| ENSG00000259248 | 1.653079 | 6.513769 | -1.97834 | 0.000013861995 | 0.000044909575 |
| ENSG00000226281 | 2.904361 | 7.232504 | -1.31627 | 0.000016369949 | 0.000052685105 |
| ENSG00000166965 | 43.6322 | 95.18443 | -1.12533 | 0.000019712017 | 0.000062754617 |
| ENSG00000244184 | 6.114612 | 2.129413 | 1.521805 | 0.000021477317 | 0.000068109959 |
| ENSG00000131351 | 9.61509 | 19.88983 | -1.04866 | 0.000021690639 | 0.000068748449 |
| ENSG00000250920 | 3.408477 | 7.014958 | -1.04131 | 0.000022869873 | 0.000072326172 |
| ENSG00000145348 | 6.252496 | 2.967116 | 1.075371 | 0.000030282225 | 0.000094414372 |
| ENSG00000229344 | 6.280814 | 2.974192 | 1.078454 | 0.000031050648 | 0.000096718173 |
| ENSG00000133134 | 2.870388 | 5.833993 | -1.02324 | 0.000032105398 | 0.000099840913 |
| ENSG00000233483 | 10.08907 | 4.48961 | 1.168131 | 0.000035849467 | 0.000110793396 |
| ENSG00000224126 | 4.17573 | 9.274365 | -1.15122 | 0.000036761727 | 0.000113437035 |
| ENSG00000254706 | 4.845965 | 10.11318 | -1.06138 | 0.000038956634 | 0.000119815149 |
| ENSG00000127720 | 5.851532 | 2.421038 | 1.273189 | 0.000039381493 | 0.000121040720 |
| ENSG00000178107 | 5.361255 | 1.470725 | 1.866043 | 0.000041893213 | 0.000128347960 |
| ENSG00000260418 | 12.07161 | 5.915083 | 1.029148 | 0.000042381635 | 0.000129775020 |
| ENSG00000167578 | 12.72656 | 5.758515 | 1.144074 | 0.000043260910 | 0.000132308492 |
| ENSG00000251992 | 0.13179 | 6.507768 | -5.62585 | 0.000043389744 | 0.000132649473 |
| ENSG00000278202 | 5.217617 | 2.423172 | 1.106494 | 0.000055025999 | 0.000166098740 |
| ENSG00000270038 | 3.006403 | 6.497783 | -1.11191 | 0.000056957926 | 0.000171749592 |
| ENSG00000235241 | 8.078395 | 3.886057 | 1.055762 | 0.000059670747 | 0.000179505128 |
| ENSG00000243988 | 8.549305 | 3.800638 | 1.169566 | 0.000068353775 | 0.000204526474 |
| ENSG00000232987 | 3.071009 | 6.610671 | -1.10608 | 0.000070125011 | 0.000209607640 |
| ENSG00000275560 | 6.136312 | 2.676549 | 1.196998 | 0.000074420828 | 0.000221927669 |
| ENSG00000173867 | 7.261626 | 3.581302 | 1.019808 | 0.000074684794 | 0.000222685888 |
| ENSG00000228705 | 2.359114 | 6.16747 | -1.38643 | 0.000083805186 | 0.000248106604 |
| ENSG00000248607 | 5.837608 | 12.48523 | -1.09677 | 0.000093256278 | 0.000274404879 |
| ENSG00000270820 | 3.970258 | 9.106625 | -1.19768 | 0.000093256278 | 0.000274404879 |
| ENSG00000244128 | 3.39453 | 7.536037 | -1.15059 | 0.000104666253 | 0.000306035747 |
| ENSG00000238597 | 10.64172 | 0.172254 | 5.949051 | 0.000106484762 | 0.000311016025 |
| ENSG00000203684 | 2.745143 | 6.286121 | -1.19529 | 0.000123241607 | 0.000357840475 |
| ENSG00000150054 | 3.890296 | 8.154612 | -1.06774 | 0.000151185900 | 0.000433709589 |
| ENSG00000153094 | 6.847607 | 3.103173 | 1.141856 | 0.000173610462 | 0.000493901853 |
| ENSG00000238795 | 8.888731 | 3.385566 | 1.39258 | 0.000226807456 | 0.000635548395 |
| ENSG00000234630 | 2.398721 | 5.849597 | -1.28607 | 0.000240350746 | 0.000671203448 |
| ENSG00000267265 | 6.222302 | 2.61572 | 1.25024 | 0.000308572881 | 0.000846674979 |
| ENSG00000239246 | 6.318804 | 2.69388 | 1.229966 | 0.000319210597 | 0.000873773318 |
| ENSG00000226278 | 5.94444 | 1.609653 | 1.884791 | 0.000325023601 | 0.000888730963 |
| ENSG00000282386 | 3.116425 | 6.24618 | -1.00308 | 0.000371208983 | 0.001006620837 |
| ENSG00000241975 | 7.69304 | 3.181439 | 1.273874 | 0.000456468971 | 0.001222008525 |
| ENSG00000260727 | 2.318432 | 5.540525 | -1.25687 | 0.000461591239 | 0.001234496956 |
| ENSG00000226564 | 3.067953 | 6.682032 | -1.12301 | 0.000466986773 | 0.001248199502 |
| ENSG00000255142 | 2.042575 | 6.716921 | -1.71741 | 0.000480365399 | 0.001280602302 |
| ENSG00000220804 | 6.064767 | 2.174572 | 1.479721 | 0.000549687343 | 0.001452329157 |
| ENSG00000239797 | 5.944034 | 2.71273 | 1.131697 | 0.000574087032 | 0.001512267838 |
| ENSG00000154380 | 24.82115 | 52.16511 | -1.07152 | 0.000600820545 | 0.001576715841 |
| ENSG00000128908 | 14.18014 | 35.71541 | -1.33267 | 0.000628305386 | 0.001645080312 |
| ENSG00000178075 | 5.484102 | 2.288896 | 1.260603 | 0.000649940354 | 0.001699012359 |
| ENSG00000278876 | 2.456052 | 6.718362 | -1.45177 | 0.000684006610 | 0.001783391781 |
| ENSG00000015153 | 5.566939 | 11.85952 | -1.09109 | 0.000756181815 | 0.001954690802 |
| ENSG00000265749 | 5.549641 | 2.343099 | 1.243977 | 0.000770945470 | 0.001990051512 |
| ENSG00000270412 | 5.733322 | 2.50552 | 1.194261 | 0.000855111118 | 0.002195821991 |
| ENSG00000135437 | 3.274583 | 7.102071 | -1.11693 | 0.000884698230 | 0.002268751111 |
| ENSG00000202503 | 25.8909 | 9.826547 | 1.397689 | 0.000964203045 | 0.002450858546 |
| ENSG00000279528 | 6.875736 | 3.345413 | 1.03933 | 0.000981630968 | 0.002491982843 |
| ENSG00000276965 | 4.198428 | 10.35382 | -1.30224 | 0.001139313800 | 0.002869105018 |
| ENSG00000126070 | 2.458848 | 7.180855 | -1.54617 | 0.001240818513 | 0.003109364346 |
| ENSG00000284258 | 0.375786 | 9.956633 | -4.72767 | 0.001339943831 | 0.003344066053 |
| ENSG00000206878 | 8.182586 | 1.902349 | 2.104775 | 0.001362025327 | 0.003393637304 |
| ENSG00000253616 | 2.791175 | 6.265612 | -1.16658 | 0.001380000795 | 0.003436372720 |
| ENSG00000253683 | 8.09879 | 2.808606 | 1.527852 | 0.001431000526 | 0.003557384047 |
| ENSG00000254741 | 6.838895 | 3.383188 | 1.01538 | 0.001506930515 | 0.003724150552 |
| ENSG00000202521 | 7.555683 | 1.331807 | 2.504177 | 0.001619466152 | 0.003980612929 |
| ENSG00000281420 | 0.0001 | 5.441983 | -15.7318 | 0.001628988144 | 0.003998021497 |
| ENSG00000269814 | 5.117262 | 2.312912 | 1.145662 | 0.001700985265 | 0.004156715937 |
| ENSG00000265660 | 26.14595 | 10.01967 | 1.383753 | 0.001753691346 | 0.004277996305 |
| ENSG00000277887 | 16.10616 | 7.005337 | 1.201086 | 0.001877154314 | 0.004561473055 |
| ENSG00000278662 | 5.498786 | 2.002964 | 1.456977 | 0.001890556362 | 0.004590150812 |
| ENSG00000204603 | 2.673984 | 5.423295 | -1.02018 | 0.001906680884 | 0.004624406575 |
| ENSG00000161057 | 70.28458 | 180.0995 | -1.35751 | 0.001925633929 | 0.004663965467 |
| ENSG00000212452 | 14.28067 | 3.492603 | 2.031689 | 0.001933984980 | 0.004681967964 |
| ENSG00000254485 | 3.532033 | 7.733324 | -1.13059 | 0.002072148948 | 0.004994577735 |
| ENSG00000268357 | 6.005813 | 2.79252 | 1.104792 | 0.002288871115 | 0.005476106242 |
| ENSG00000252743 | 16.34552 | 6.611354 | 1.305878 | 0.003649234750 | 0.008384122776 |
| ENSG00000277942 | 10.22455 | 24.78643 | -1.27751 | 0.005163534638 | 0.011529574107 |
| ENSG00000231887 | 4.474112 | 8.989506 | -1.00664 | 0.005171197009 | 0.011543874700 |
| ENSG00000272030 | 2.896429 | 6.126413 | -1.08077 | 0.006471969335 | 0.014126033525 |
| ENSG00000273709 | 7.545579 | 3.125391 | 1.271595 | 0.006685194469 | 0.014569945562 |
| ENSG00000276753 | 23.50352 | 10.82465 | 1.118557 | 0.006926359043 | 0.015029164164 |
| ENSG00000210154 | 15.09233 | 4.608305 | 1.711508 | 0.007280251023 | 0.015705710072 |
| ENSG00000258788 | 5.184825 | 1.892041 | 1.454352 | 0.007844683674 | 0.016833146804 |
| ENSG00000265933 | 3.427305 | 7.968639 | -1.21726 | 0.007862881937 | 0.016868252751 |
| ENSG00000185837 | 5.231569 | 2.080845 | 1.330074 | 0.010206951952 | 0.021333482631 |
| ENSG00000201998 | 6.10494 | 2.237525 | 1.448073 | 0.010627518167 | 0.022143920157 |
| ENSG00000202252 | 7.244886 | 1.691379 | 2.098763 | 0.011625296444 | 0.024022227370 |
| ENSG00000208772 | 8.528011 | 3.362806 | 1.342544 | 0.012339416113 | 0.025368778074 |
| ENSG00000210151 | 17.05645 | 6.619739 | 1.365471 | 0.012339416113 | 0.025368778074 |
| ENSG00000273061 | 5.057127 | 2.431535 | 1.056451 | 0.012764781038 | 0.025923889013 |
| ENSG00000284520 | 0.303376 | 5.362282 | -4.14367 | 0.013373894811 | 0.027069881570 |
| ENSG00000182022 | 5.821472 | 11.9392 | -1.03625 | 0.015385559205 | 0.030771118409 |
| ENSG00000257921 | 2.261379 | 6.441549 | -1.5102 | 0.015973973350 | 0.031866003960 |
| ENSG00000210174 | 16.25554 | 6.451745 | 1.33317 | 0.016823109871 | 0.033378539681 |
| ENSG00000172748 | 1.024341 | 5.967116 | -2.54234 | 0.021838412344 | 0.042203456123 |
| ENSG00000041353 | 2.278814 | 5.18227 | -1.1853 | 0.024548035482 | 0.046974989665 |

**Sup.Tab.3** RNA-seq analysis of the gene expression profile affected by sh-2 and sh-NC treatment for SNX9.

| Gene id | MeanTPM (sh-2) | MeanTPM (sh-NC) | log2FoldChange | pValue | qValue |
| --- | --- | --- | --- | --- | --- |
| ENSG00000167996 | 1522.617 | 8115.109 | -2.41406 | 0.000000000000 | 0.000000000000 |
| ENSG00000137693 | 104.4371 | 1288.154 | -3.6246 | 0.000000000000 | 0.000000000000 |
| ENSG00000142871 | 74.87948 | 1064.849 | -3.82993 | 0.000000000000 | 0.000000000000 |
| ENSG00000128422 | 1726.865 | 4080.206 | -1.24049 | 0.000000000000 | 0.000000000000 |
| ENSG00000124942 | 62.38624 | 29.97977 | 1.057238 | 0.000000000000 | 0.000000000000 |
| ENSG00000013588 | 357.9435 | 735.5185 | -1.03903 | 0.000000000000 | 0.000000000000 |
| ENSG00000196924 | 716.3938 | 1601.531 | -1.16063 | 0.000000000000 | 0.000000000000 |
| ENSG00000255508 | 220.7141 | 33.99407 | 2.698824 | 0.000000000000 | 0.000000000000 |
| ENSG00000125730 | 1368.147 | 452.748 | 1.595444 | 0.000000000000 | 0.000000000000 |
| ENSG00000161011 | 239.3861 | 753.3383 | -1.65396 | 0.000000000000 | 0.000000000000 |
| ENSG00000166598 | 233.6386 | 594.2241 | -1.34673 | 0.000000000000 | 0.000000000000 |
| ENSG00000237973 | 403.3029 | 1082.635 | -1.42461 | 0.000000000000 | 0.000000000000 |
| ENSG00000146674 | 542.9229 | 1112.46 | -1.03493 | 0.000000000000 | 0.000000000000 |
| ENSG00000196352 | 603.1342 | 1236.996 | -1.03629 | 0.000000000000 | 0.000000000000 |
| ENSG00000044574 | 264.1476 | 577.0569 | -1.12737 | 0.000000000000 | 0.000000000000 |
| ENSG00000274012 | 2604.823 | 529.6602 | 2.298046 | 0.000000000000 | 0.000000000000 |
| ENSG00000198918 | 265.4282 | 1511.186 | -2.50929 | 0.000000000000 | 0.000000000000 |
| ENSG00000276168 | 2578.46 | 551.9261 | 2.223963 | 0.000000000000 | 0.000000000000 |
| ENSG00000116285 | 32.1517 | 229.5957 | -2.83613 | 0.000000000000 | 0.000000000000 |
| ENSG00000142627 | 128.2564 | 340.3448 | -1.40797 | 0.000000000000 | 0.000000000000 |
| ENSG00000167325 | 182.9301 | 544.7104 | -1.5742 | 0.000000000000 | 0.000000000000 |
| ENSG00000122026 | 55.22574 | 640.7736 | -3.5364 | 0.000000000000 | 0.000000000000 |
| ENSG00000169710 | 251.2269 | 99.92799 | 1.33003 | 0.000000000000 | 0.000000000000 |
| ENSG00000237412 | 497.8948 | 189.0722 | 1.396903 | 0.000000000000 | 0.000000000000 |
| ENSG00000145425 | 122.6667 | 639.514 | -2.38223 | 0.000000000000 | 0.000000000000 |
| ENSG00000111716 | 291.5533 | 794.3792 | -1.44607 | 0.000000000000 | 0.000000000000 |
| ENSG00000120129 | 286.8113 | 669.8516 | -1.22374 | 0.000000000000 | 0.000000000000 |
| ENSG00000125148 | 264.7469 | 941.0363 | -1.82964 | 0.000000000000 | 0.000000000000 |
| ENSG00000102024 | 41.33023 | 185.0779 | -2.16286 | 0.000000000000 | 0.000000000000 |
| ENSG00000165389 | 192.2692 | 40.41221 | 2.250265 | 0.000000000000 | 0.000000000000 |
| ENSG00000248527 | 1619.557 | 647.957 | 1.321629 | 0.000000000000 | 0.000000000000 |
| ENSG00000072310 | 383.6657 | 121.9969 | 1.653005 | 0.000000000000 | 0.000000000000 |
| ENSG00000204291 | 96.0925 | 21.4538 | 2.16319 | 0.000000000000 | 0.000000000000 |
| ENSG00000080824 | 127.3112 | 269.261 | -1.08065 | 0.000000000000 | 0.000000000000 |
| ENSG00000079308 | 131.067 | 49.99197 | 1.390537 | 0.000000000000 | 0.000000000000 |
| ENSG00000258017 | 356.3826 | 1019.874 | -1.51689 | 0.000000000000 | 0.000000000000 |
| ENSG00000281181 | 629.685 | 166.2361 | 1.921397 | 0.000000000000 | 0.000000000000 |
| ENSG00000277957 | 98.84206 | 12.79078 | 2.950021 | 0.000000000000 | 0.000000000000 |
| ENSG00000147604 | 222.0876 | 678.7966 | -1.61185 | 0.000000000000 | 0.000000000000 |
| ENSG00000198431 | 226.7145 | 575.1669 | -1.3431 | 0.000000000000 | 0.000000000000 |
| ENSG00000136244 | 8.685369 | 205.0733 | -4.56141 | 0.000000000000 | 0.000000000000 |
| ENSG00000058262 | 169.5166 | 353.9067 | -1.06194 | 0.000000000000 | 0.000000000000 |
| ENSG00000144381 | 154.3817 | 397.227 | -1.36346 | 0.000000000000 | 0.000000000000 |
| ENSG00000268173 | 44.28087 | 1.448561 | 4.933991 | 0.000000000000 | 0.000000000000 |
| ENSG00000023445 | 22.57702 | 90.94766 | -2.01018 | 0.000000000000 | 0.000000000000 |
| ENSG00000106366 | 9.743946 | 89.70608 | -3.20263 | 0.000000000000 | 0.000000000000 |
| ENSG00000143570 | 282.3334 | 93.35365 | 1.596621 | 0.000000000000 | 0.000000000000 |
| ENSG00000107957 | 41.30654 | 10.6687 | 1.952987 | 0.000000000000 | 0.000000000000 |
| ENSG00000115919 | 15.84491 | 134.4001 | -3.08444 | 0.000000000000 | 0.000000000000 |
| ENSG00000162734 | 39.31604 | 151.1154 | -1.94246 | 0.000000000000 | 0.000000000000 |
| ENSG00000072274 | 103.8131 | 218.3134 | -1.07241 | 0.000000000000 | 0.000000000000 |
| ENSG00000204389 | 430.2092 | 210.8047 | 1.029132 | 0.000000000000 | 0.000000000000 |
| ENSG00000101654 | 1.40915 | 29.78619 | -4.40175 | 0.000000000000 | 0.000000000000 |
| ENSG00000117525 | 20.09074 | 142.2828 | -2.82416 | 0.000000000000 | 0.000000000000 |
| ENSG00000135046 | 108.2783 | 407.2406 | -1.91114 | 0.000000000000 | 0.000000000000 |
| ENSG00000152952 | 48.9578 | 150.2023 | -1.6173 | 0.000000000000 | 0.000000000000 |
| ENSG00000095303 | 136.4634 | 58.88503 | 1.212541 | 0.000000000000 | 0.000000000000 |
| ENSG00000163435 | 393.4181 | 168.4787 | 1.223497 | 0.000000000000 | 0.000000000000 |
| ENSG00000103187 | 131.3345 | 346.7943 | -1.40083 | 0.000000000000 | 0.000000000000 |
| ENSG00000169180 | 89.22257 | 185.5785 | -1.05655 | 0.000000000000 | 0.000000000000 |
| ENSG00000169604 | 60.67312 | 151.2794 | -1.31809 | 0.000000000000 | 0.000000000000 |
| ENSG00000104408 | 24.02008 | 144.0393 | -2.58415 | 0.000000000000 | 0.000000000000 |
| ENSG00000064687 | 183.0426 | 76.65553 | 1.255717 | 0.000000000000 | 0.000000000000 |
| ENSG00000135114 | 336.5996 | 160.1052 | 1.072013 | 0.000000000000 | 0.000000000000 |
| ENSG00000113739 | 33.88232 | 94.89493 | -1.4858 | 0.000000000000 | 0.000000000000 |
| ENSG00000259040 | 144.2813 | 292.8124 | -1.02109 | 0.000000000000 | 0.000000000000 |
| ENSG00000138772 | 25.04069 | 155.0399 | -2.63029 | 0.000000000000 | 0.000000000000 |
| ENSG00000283293 | 780.8082 | 158.6336 | 2.29927 | 0.000000000000 | 0.000000000000 |
| ENSG00000122884 | 20.21203 | 96.89838 | -2.26126 | 0.000000000000 | 0.000000000000 |
| ENSG00000159176 | 205.2008 | 439.1967 | -1.09783 | 0.000000000000 | 0.000000000000 |
| ENSG00000114019 | 55.33068 | 186.7007 | -1.75458 | 0.000000000000 | 0.000000000000 |
| ENSG00000006327 | 151.9216 | 342.2341 | -1.17166 | 0.000000000000 | 0.000000000000 |
| ENSG00000164924 | 43.53165 | 181.3329 | -2.0585 | 0.000000000000 | 0.000000000000 |
| ENSG00000137331 | 205.9567 | 469.2563 | -1.18803 | 0.000000000000 | 0.000000000000 |
| ENSG00000144824 | 42.14714 | 134.6444 | -1.67565 | 0.000000000000 | 0.000000000000 |
| ENSG00000167775 | 534.3856 | 245.6723 | 1.121146 | 0.000000000000 | 0.000000000000 |
| ENSG00000138031 | 213.0629 | 78.55787 | 1.439451 | 0.000000000000 | 0.000000000000 |
| ENSG00000109046 | 63.92016 | 284.4629 | -2.1539 | 0.000000000000 | 0.000000000000 |
| ENSG00000105976 | 44.77576 | 99.88092 | -1.15749 | 0.000000000000 | 0.000000000000 |
| ENSG00000280614 | 374.7471 | 121.2577 | 1.627841 | 0.000000000000 | 0.000000000000 |
| ENSG00000204054 | 219.3598 | 107.9744 | 1.022609 | 0.000000000000 | 0.000000000000 |
| ENSG00000087074 | 38.53396 | 132.249 | -1.77905 | 0.000000000000 | 0.000000000000 |
| ENSG00000111859 | 14.81113 | 59.88854 | -2.0156 | 0.000000000000 | 0.000000000000 |
| ENSG00000138166 | 26.07173 | 103.0572 | -1.98289 | 0.000000000000 | 0.000000000000 |
| ENSG00000142910 | 92.09924 | 220.9791 | -1.26265 | 0.000000000000 | 0.000000000000 |
| ENSG00000100092 | 6.45774 | 51.76777 | -3.00295 | 0.000000000000 | 0.000000000000 |
| ENSG00000255717 | 88.43142 | 323.6706 | -1.8719 | 0.000000000000 | 0.000000000000 |
| ENSG00000092201 | 36.70837 | 89.39188 | -1.28403 | 0.000000000000 | 0.000000000000 |
| ENSG00000237550 | 57.22855 | 319.4269 | -2.48068 | 0.000000000000 | 0.000000000000 |
| ENSG00000243716 | 74.09818 | 35.47695 | 1.062556 | 0.000000000000 | 0.000000000000 |
| ENSG00000173391 | 7.334275 | 62.59703 | -3.09337 | 0.000000000000 | 0.000000000000 |
| ENSG00000108821 | 144.3863 | 69.30804 | 1.058839 | 0.000000000000 | 0.000000000000 |
| ENSG00000160211 | 189.9689 | 83.80079 | 1.180728 | 0.000000000000 | 0.000000000000 |
| ENSG00000086598 | 28.1971 | 94.25686 | -1.74105 | 0.000000000000 | 0.000000000000 |
| ENSG00000233276 | 263.4947 | 680.3718 | -1.36855 | 0.000000000000 | 0.000000000000 |
| ENSG00000129083 | 30.66159 | 86.37285 | -1.49415 | 0.000000000000 | 0.000000000000 |
| ENSG00000163347 | 16.42535 | 64.4744 | -1.9728 | 0.000000000000 | 0.000000000000 |
| ENSG00000130707 | 676.9054 | 318.6505 | 1.08698 | 0.000000000000 | 0.000000000000 |
| ENSG00000143322 | 15.21222 | 40.16933 | -1.40086 | 0.000000000000 | 0.000000000000 |
| ENSG00000137801 | 6.175326 | 29.25703 | -2.2442 | 0.000000000000 | 0.000000000000 |
| ENSG00000106351 | 56.76666 | 16.79785 | 1.756767 | 0.000000000000 | 0.000000000000 |
| ENSG00000178252 | 99.23796 | 39.88927 | 1.314891 | 0.000000000000 | 0.000000000000 |
| ENSG00000111206 | 45.10838 | 104.4755 | -1.2117 | 0.000000000000 | 0.000000000000 |
| ENSG00000102172 | 140.3373 | 340.1434 | -1.27724 | 0.000000000000 | 0.000000000000 |
| ENSG00000120708 | 49.8614 | 137.9248 | -1.46789 | 0.000000000000 | 0.000000000000 |
| ENSG00000147689 | 100.2947 | 43.82692 | 1.194356 | 0.000000000000 | 0.000000000000 |
| ENSG00000162073 | 98.14322 | 21.26003 | 2.206745 | 0.000000000000 | 0.000000000000 |
| ENSG00000167772 | 86.99195 | 227.6768 | -1.38803 | 0.000000000000 | 0.000000000000 |
| ENSG00000115484 | 57.07644 | 150.0715 | -1.39468 | 0.000000000000 | 0.000000000000 |
| ENSG00000079785 | 18.88473 | 75.76071 | -2.00423 | 0.000000000000 | 0.000000000000 |
| ENSG00000156273 | 11.17333 | 43.05792 | -1.94622 | 0.000000000000 | 0.000000000000 |
| ENSG00000188153 | 90.19756 | 43.47642 | 1.052855 | 0.000000000000 | 0.000000000000 |
| ENSG00000148358 | 17.16169 | 46.81048 | -1.44764 | 0.000000000000 | 0.000000000000 |
| ENSG00000128050 | 32.82182 | 82.56615 | -1.3309 | 0.000000000000 | 0.000000000000 |
| ENSG00000241360 | 78.51775 | 15.41179 | 2.348984 | 0.000000000000 | 0.000000000000 |
| ENSG00000135919 | 26.72032 | 90.4394 | -1.75901 | 0.000000000000 | 0.000000000000 |
| ENSG00000087586 | 43.72521 | 102.221 | -1.22515 | 0.000000000000 | 0.000000000000 |
| ENSG00000173227 | 168.5766 | 82.15214 | 1.037034 | 0.000000000000 | 0.000000000000 |
| ENSG00000187634 | 119.8085 | 27.01196 | 2.14906 | 0.000000000000 | 0.000000000000 |
| ENSG00000263740 | 631.6392 | 160.4339 | 1.977122 | 0.000000000000 | 0.000000000000 |
| ENSG00000177000 | 36.55113 | 9.562443 | 1.934465 | 0.000000000000 | 0.000000000000 |
| ENSG00000221869 | 119.3577 | 40.94373 | 1.543577 | 0.000000000000 | 0.000000000000 |
| ENSG00000270136 | 50.38129 | 0.696356 | 6.176919 | 0.000000000000 | 0.000000000000 |
| ENSG00000171161 | 42.60628 | 10.58061 | 2.009644 | 0.000000000000 | 0.000000000000 |
| ENSG00000126351 | 48.7214 | 12.75864 | 1.933081 | 0.000000000000 | 0.000000000000 |
| ENSG00000106799 | 7.358852 | 42.8855 | -2.54294 | 0.000000000000 | 0.000000000000 |
| ENSG00000090539 | 57.39818 | 11.52904 | 2.315732 | 0.000000000000 | 0.000000000000 |
| ENSG00000124207 | 13.7348 | 52.34059 | -1.93009 | 0.000000000000 | 0.000000000000 |
| ENSG00000101255 | 59.71193 | 142.0244 | -1.25005 | 0.000000000000 | 0.000000000000 |
| ENSG00000079257 | 139.904 | 30.20948 | 2.211365 | 0.000000000000 | 0.000000000000 |
| ENSG00000158615 | 42.91567 | 89.34271 | -1.05785 | 0.000000000000 | 0.000000000000 |
| ENSG00000101236 | 63.1866 | 28.99811 | 1.12366 | 0.000000000000 | 0.000000000000 |
| ENSG00000143947 | 308.2809 | 715.4911 | -1.21469 | 0.000000000000 | 0.000000000000 |
| ENSG00000115365 | 103.8396 | 39.27602 | 1.402636 | 0.000000000000 | 0.000000000000 |
| ENSG00000148411 | 21.15726 | 4.267676 | 2.30963 | 0.000000000000 | 0.000000000000 |
| ENSG00000163938 | 28.04535 | 110.1416 | -1.97353 | 0.000000000000 | 0.000000000000 |
| ENSG00000113732 | 58.57647 | 172.4077 | -1.55743 | 0.000000000000 | 0.000000000000 |
| ENSG00000253352 | 23.9711 | 54.21298 | -1.17734 | 0.000000000000 | 0.000000000000 |
| ENSG00000058668 | 56.72121 | 11.96907 | 2.244578 | 0.000000000000 | 0.000000000000 |
| ENSG00000139289 | 2.623854 | 19.51024 | -2.89447 | 0.000000000000 | 0.000000000000 |
| ENSG00000187908 | 5.306388 | 23.17676 | -2.12688 | 0.000000000000 | 0.000000000000 |
| ENSG00000271303 | 30.49628 | 90.21783 | -1.56478 | 0.000000000000 | 0.000000000000 |
| ENSG00000253729 | 164.302 | 64.04231 | 1.359253 | 0.000000000000 | 0.000000000000 |
| ENSG00000163659 | 10.80907 | 54.84593 | -2.34314 | 0.000000000000 | 0.000000000000 |
| ENSG00000126777 | 4.092661 | 26.9227 | -2.71771 | 0.000000000000 | 0.000000000000 |
| ENSG00000104140 | 172.7404 | 68.72408 | 1.329718 | 0.000000000000 | 0.000000000000 |
| ENSG00000187193 | 58.15234 | 299.5215 | -2.36475 | 0.000000000000 | 0.000000000000 |
| ENSG00000275216 | 41.41526 | 91.54212 | -1.14427 | 0.000000000000 | 0.000000000000 |
| ENSG00000113916 | 113.1423 | 50.43504 | 1.165641 | 0.000000000000 | 0.000000000000 |
| ENSG00000224389 | 43.82162 | 13.85739 | 1.660988 | 0.000000000000 | 0.000000000000 |
| ENSG00000139182 | 103.2245 | 38.67713 | 1.416232 | 0.000000000000 | 0.000000000000 |
| ENSG00000120875 | 4.149472 | 23.06345 | -2.47461 | 0.000000000000 | 0.000000000000 |
| ENSG00000197930 | 16.61391 | 70.72547 | -2.08984 | 0.000000000000 | 0.000000000000 |
| ENSG00000156467 | 4.327854 | 37.37625 | -3.1104 | 0.000000000000 | 0.000000000000 |
| ENSG00000101986 | 99.69649 | 44.93098 | 1.149832 | 0.000000000000 | 0.000000000000 |
| ENSG00000073921 | 79.95942 | 233.5868 | -1.54662 | 0.000000000000 | 0.000000000000 |
| ENSG00000188707 | 53.38894 | 13.44818 | 1.98913 | 0.000000000000 | 0.000000000000 |
| ENSG00000143416 | 150.655 | 58.898 | 1.354958 | 0.000000000000 | 0.000000000000 |
| ENSG00000280800 | 279.254 | 106.4976 | 1.390757 | 0.000000000000 | 0.000000000000 |
| ENSG00000117143 | 52.87046 | 136.1647 | -1.36482 | 0.000000000000 | 0.000000000000 |
| ENSG00000096746 | 98.77938 | 235.992 | -1.25646 | 0.000000000000 | 0.000000000000 |
| ENSG00000131747 | 19.09609 | 49.59816 | -1.37701 | 0.000000000000 | 0.000000000000 |
| ENSG00000124145 | 61.91302 | 137.2154 | -1.14813 | 0.000000000000 | 0.000000000000 |
| ENSG00000122035 | 101.9646 | 17.13928 | 2.57269 | 0.000000000000 | 0.000000000000 |
| ENSG00000069011 | 256.3136 | 81.75179 | 1.648588 | 0.000000000000 | 0.000000000000 |
| ENSG00000116741 | 15.78201 | 82.20533 | -2.38095 | 0.000000000000 | 0.000000000000 |
| ENSG00000234975 | 24.24012 | 221.0249 | -3.18874 | 0.000000000000 | 0.000000000000 |
| ENSG00000198492 | 44.08571 | 12.32914 | 1.838239 | 0.000000000000 | 0.000000000000 |
| ENSG00000101782 | 15.25483 | 51.36385 | -1.75149 | 0.000000000000 | 0.000000000000 |
| ENSG00000130066 | 47.57964 | 162.2097 | -1.76944 | 0.000000000000 | 0.000000000000 |
| ENSG00000041357 | 6.218572 | 48.90033 | -2.97519 | 0.000000000000 | 0.000000000000 |
| ENSG00000101126 | 14.60405 | 34.78281 | -1.25201 | 0.000000000000 | 0.000000000000 |
| ENSG00000198722 | 49.61533 | 19.10373 | 1.376931 | 0.000000000000 | 0.000000000000 |
| ENSG00000214736 | 153.6386 | 0.005323 | 14.81694 | 0.000000000000 | 0.000000000000 |
| ENSG00000084652 | 65.83199 | 29.4507 | 1.160487 | 0.000000000000 | 0.000000000000 |
| ENSG00000110092 | 23.41967 | 61.34182 | -1.38915 | 0.000000000000 | 0.000000000000 |
| ENSG00000113643 | 9.00792 | 51.3633 | -2.51147 | 0.000000000000 | 0.000000000000 |
| ENSG00000090339 | 24.96147 | 68.24806 | -1.45109 | 0.000000000000 | 0.000000000000 |
| ENSG00000115758 | 35.34729 | 107.1528 | -1.6 | 0.000000000000 | 0.000000000000 |
| ENSG00000140525 | 38.16702 | 94.6056 | -1.3096 | 0.000000000000 | 0.000000000000 |
| ENSG00000100342 | 6.816349 | 37.09788 | -2.44427 | 0.000000000000 | 0.000000000000 |
| ENSG00000123562 | 101.4034 | 223.231 | -1.13843 | 0.000000000000 | 0.000000000000 |
| ENSG00000011052 | 154.5233 | 318.2119 | -1.04216 | 0.000000000000 | 0.000000000000 |
| ENSG00000176087 | 94.29533 | 36.91602 | 1.352939 | 0.000000000000 | 0.000000000000 |
| ENSG00000183779 | 55.87743 | 18.8129 | 1.570544 | 0.000000000000 | 0.000000000000 |
| ENSG00000065325 | 39.40004 | 79.11723 | -1.00579 | 0.000000000000 | 0.000000000000 |
| ENSG00000173812 | 37.90886 | 125.0713 | -1.72214 | 0.000000000000 | 0.000000000000 |
| ENSG00000244731 | 44.2492 | 12.86394 | 1.782319 | 0.000000000000 | 0.000000000000 |
| ENSG00000115524 | 47.95897 | 98.68902 | -1.04109 | 0.000000000000 | 0.000000000000 |
| ENSG00000172780 | 31.04679 | 7.626609 | 2.025331 | 0.000000000000 | 0.000000000000 |
| ENSG00000100664 | 12.19791 | 36.52042 | -1.58207 | 0.000000000000 | 0.000000000000 |
| ENSG00000173237 | 266.1401 | 125.1348 | 1.088703 | 0.000000000000 | 0.000000000000 |
| ENSG00000277027 | 298.1484 | 34.74024 | 3.101351 | 0.000000000000 | 0.000000000000 |
| ENSG00000164543 | 36.10839 | 88.60084 | -1.29499 | 0.000000000000 | 0.000000000000 |
| ENSG00000181885 | 258.5431 | 104.3781 | 1.308585 | 0.000000000000 | 0.000000000000 |
| ENSG00000177606 | 20.35871 | 56.57069 | -1.47441 | 0.000000000000 | 0.000000000000 |
| ENSG00000108819 | 85.28319 | 39.48912 | 1.110806 | 0.000000000000 | 0.000000000000 |
| ENSG00000148175 | 196.2872 | 96.90864 | 1.018269 | 0.000000000000 | 0.000000000000 |
| ENSG00000101608 | 78.87919 | 193.0465 | -1.29123 | 0.000000000000 | 0.000000000000 |
| ENSG00000184575 | 14.60561 | 37.63737 | -1.36564 | 0.000000000000 | 0.000000000000 |
| ENSG00000088256 | 75.46352 | 36.5347 | 1.046512 | 0.000000000000 | 0.000000000000 |
| ENSG00000070915 | 66.91099 | 27.63952 | 1.27551 | 0.000000000000 | 0.000000000000 |
| ENSG00000172379 | 17.69871 | 6.222623 | 1.508049 | 0.000000000000 | 0.000000000000 |
| ENSG00000255339 | 88.68805 | 19.51516 | 2.184144 | 0.000000000000 | 0.000000000000 |
| ENSG00000175040 | 50.08347 | 20.9754 | 1.255636 | 0.000000000000 | 0.000000000000 |
| ENSG00000128510 | 6.025421 | 37.07925 | -2.62148 | 0.000000000000 | 0.000000000000 |
| ENSG00000116574 | 35.56544 | 11.59871 | 1.616512 | 0.000000000000 | 0.000000000000 |
| ENSG00000153162 | 28.45546 | 73.72942 | -1.37353 | 0.000000000000 | 0.000000000000 |
| ENSG00000127804 | 7.364857 | 18.55121 | -1.33278 | 0.000000000000 | 0.000000000000 |
| ENSG00000281383 | 184.3966 | 65.00731 | 1.504138 | 0.000000000000 | 0.000000000000 |
| ENSG00000088325 | 47.96928 | 96.37257 | -1.00651 | 0.000000000000 | 0.000000000000 |
| ENSG00000028203 | 15.28656 | 54.796 | -1.84181 | 0.000000000000 | 0.000000000000 |
| ENSG00000101544 | 23.623 | 6.5267 | 1.855767 | 0.000000000000 | 0.000000000000 |
| ENSG00000244255 | 13.36842 | 0.126421 | 6.724449 | 0.000000000000 | 0.000000000000 |
| ENSG00000143933 | 71.18908 | 175.9486 | -1.30543 | 0.000000000000 | 0.000000000000 |
| ENSG00000210140 | 3131.188 | 1267.158 | 1.305114 | 0.000000000000 | 0.000000000000 |
| ENSG00000119922 | 24.16797 | 63.28692 | -1.38881 | 0.000000000000 | 0.000000000000 |
| ENSG00000105289 | 45.96011 | 13.65765 | 1.750673 | 0.000000000000 | 0.000000000000 |
| ENSG00000113387 | 15.55849 | 52.45106 | -1.75327 | 0.000000000000 | 0.000000000000 |
| ENSG00000054793 | 39.02939 | 19.19312 | 1.023972 | 0.000000000000 | 0.000000000000 |
| ENSG00000115963 | 21.33429 | 85.41354 | -2.00129 | 0.000000000000 | 0.000000000000 |
| ENSG00000112078 | 22.42105 | 50.243 | -1.16407 | 0.000000000000 | 0.000000000000 |
| ENSG00000228526 | 22.98278 | 0.0001 | 17.81019 | 0.000000000000 | 0.000000000000 |
| ENSG00000006652 | 17.58641 | 79.47433 | -2.17603 | 0.000000000000 | 0.000000000000 |
| ENSG00000049239 | 52.41455 | 25.39376 | 1.045493 | 0.000000000000 | 0.000000000000 |
| ENSG00000171631 | 53.95867 | 10.07015 | 2.421769 | 0.000000000000 | 0.000000000000 |
| ENSG00000270276 | 17.15283 | 67.29474 | -1.97205 | 0.000000000000 | 0.000000000000 |
| ENSG00000166025 | 22.28991 | 9.213736 | 1.274533 | 0.000000000000 | 0.000000000000 |
| ENSG00000117395 | 110.1743 | 237.7915 | -1.10991 | 0.000000000000 | 0.000000000000 |
| ENSG00000065534 | 42.70369 | 19.00286 | 1.168144 | 0.000000000000 | 0.000000000000 |
| ENSG00000185022 | 66.23257 | 23.92292 | 1.469147 | 0.000000000000 | 0.000000000000 |
| ENSG00000181458 | 34.43382 | 93.98034 | -1.44853 | 0.000000000000 | 0.000000000000 |
| ENSG00000134531 | 13.26266 | 73.00797 | -2.46068 | 0.000000000000 | 0.000000000000 |
| ENSG00000132967 | 35.94811 | 153.8557 | -2.09759 | 0.000000000000 | 0.000000000000 |
| ENSG00000167315 | 24.02562 | 80.61488 | -1.74647 | 0.000000000000 | 0.000000000000 |
| ENSG00000070756 | 369.8303 | 743.7407 | -1.00794 | 0.000000000000 | 0.000000000000 |
| ENSG00000151414 | 4.005568 | 22.41707 | -2.48452 | 0.000000000000 | 0.000000000000 |
| ENSG00000133706 | 19.15289 | 53.07212 | -1.47039 | 0.000000000000 | 0.000000000000 |
| ENSG00000117983 | 6.263389 | 1.84788 | 1.761072 | 0.000000000000 | 0.000000000000 |
| ENSG00000171552 | 39.66205 | 85.23518 | -1.10369 | 0.000000000000 | 0.000000000000 |
| ENSG00000145506 | 54.73568 | 122.0475 | -1.15689 | 0.000000000000 | 0.000000000000 |
| ENSG00000137628 | 5.062605 | 18.58692 | -1.87634 | 0.000000000000 | 0.000000000000 |
| ENSG00000154518 | 371.6099 | 117.4947 | 1.661193 | 0.000000000000 | 0.000000000000 |
| ENSG00000156510 | 15.99625 | 44.79305 | -1.48554 | 0.000000000000 | 0.000000000000 |
| ENSG00000137936 | 37.50143 | 79.52197 | -1.08441 | 0.000000000000 | 0.000000000000 |
| ENSG00000178913 | 27.12178 | 70.36431 | -1.37539 | 0.000000000000 | 0.000000000000 |
| ENSG00000146112 | 32.96496 | 72.46555 | -1.13636 | 0.000000000000 | 0.000000000000 |
| ENSG00000115380 | 50.01743 | 113.3175 | -1.17987 | 0.000000000000 | 0.000000000000 |
| ENSG00000125977 | 50.20271 | 101.2572 | -1.01219 | 0.000000000000 | 0.000000000000 |
| ENSG00000175197 | 24.06219 | 98.48229 | -2.0331 | 0.000000000000 | 0.000000000000 |
| ENSG00000129128 | 122.2889 | 9.630301 | 3.666568 | 0.000000000000 | 0.000000000000 |
| ENSG00000164930 | 5.246425 | 28.5229 | -2.44271 | 0.000000000000 | 0.000000000000 |
| ENSG00000011426 | 56.42843 | 116.5234 | -1.04613 | 0.000000000000 | 0.000000000000 |
| ENSG00000101972 | 8.188043 | 21.56483 | -1.39709 | 0.000000000000 | 0.000000000000 |
| ENSG00000102034 | 30.26334 | 9.087803 | 1.735568 | 0.000000000000 | 0.000000000000 |
| ENSG00000214389 | 6.889951 | 68.39671 | -3.31136 | 0.000000000000 | 0.000000000000 |
| ENSG00000151366 | 327.1816 | 146.2882 | 1.161278 | 0.000000000000 | 0.000000000000 |
| ENSG00000228253 | 79.90579 | 385.0959 | -2.26885 | 0.000000000000 | 0.000000000000 |
| ENSG00000172939 | 14.34063 | 36.35912 | -1.34221 | 0.000000000000 | 0.000000000000 |
| ENSG00000168398 | 20.87707 | 4.901495 | 2.090626 | 0.000000000000 | 0.000000000000 |
| ENSG00000175029 | 44.10923 | 138.8921 | -1.65481 | 0.000000000000 | 0.000000000000 |
| ENSG00000012963 | 4.351363 | 20.3653 | -2.22657 | 0.000000000000 | 0.000000000000 |
| ENSG00000140853 | 46.66041 | 21.39636 | 1.124833 | 0.000000000000 | 0.000000000000 |
| ENSG00000157985 | 24.98773 | 11.21264 | 1.156094 | 0.000000000000 | 0.000000000000 |
| ENSG00000072736 | 6.100401 | 17.37924 | -1.51039 | 0.000000000000 | 0.000000000000 |
| ENSG00000204569 | 86.95265 | 35.66205 | 1.28584 | 0.000000000000 | 0.000000000000 |
| ENSG00000114346 | 20.23665 | 47.52197 | -1.23162 | 0.000000000000 | 0.000000000000 |
| ENSG00000013441 | 30.8974 | 66.074 | -1.0966 | 0.000000000000 | 0.000000000000 |
| ENSG00000135052 | 66.99246 | 32.31599 | 1.05175 | 0.000000000000 | 0.000000000000 |
| ENSG00000147676 | 32.82332 | 70.9511 | -1.1121 | 0.000000000000 | 0.000000000000 |
| ENSG00000137942 | 3.4225 | 16.66306 | -2.28353 | 0.000000000000 | 0.000000000000 |
| ENSG00000082898 | 14.16649 | 49.95567 | -1.81817 | 0.000000000000 | 0.000000000000 |
| ENSG00000139921 | 1.517721 | 13.99436 | -3.20487 | 0.000000000000 | 0.000000000000 |
| ENSG00000169764 | 7.04753 | 42.18529 | -2.58155 | 0.000000000000 | 0.000000000000 |
| ENSG00000132463 | 15.47955 | 39.71169 | -1.3592 | 0.000000000000 | 0.000000000000 |
| ENSG00000277209 | 121.8875 | 5.633257 | 4.435437 | 0.000000000000 | 0.000000000000 |
| ENSG00000147224 | 97.15568 | 222.6951 | -1.1967 | 0.000000000000 | 0.000000000000 |
| ENSG00000128342 | 19.922 | 47.33786 | -1.24863 | 0.000000000000 | 0.000000000000 |
| ENSG00000101557 | 11.42324 | 33.28128 | -1.54274 | 0.000000000000 | 0.000000000000 |
| ENSG00000060491 | 189.9421 | 86.36617 | 1.137022 | 0.000000000000 | 0.000000000000 |
| ENSG00000107731 | 16.57563 | 5.433326 | 1.609156 | 0.000000000000 | 0.000000000000 |
| ENSG00000114779 | 89.39919 | 38.85341 | 1.202221 | 0.000000000000 | 0.000000000000 |
| ENSG00000078804 | 9.9652 | 0.504089 | 4.305148 | 0.000000000000 | 0.000000000000 |
| ENSG00000099326 | 67.97749 | 31.1929 | 1.123839 | 0.000000000000 | 0.000000000000 |
| ENSG00000145555 | 58.2137 | 28.19681 | 1.045827 | 0.000000000000 | 0.000000000000 |
| ENSG00000143126 | 32.24656 | 15.25895 | 1.079489 | 0.000000000000 | 0.000000000000 |
| ENSG00000148677 | 0.020697 | 28.95184 | -10.45 | 0.000000000000 | 0.000000000000 |
| ENSG00000086065 | 38.73307 | 99.95085 | -1.36765 | 0.000000000000 | 0.000000000000 |
| ENSG00000171298 | 79.86464 | 36.88767 | 1.114418 | 0.000000000000 | 0.000000000000 |
| ENSG00000150347 | 45.72142 | 16.72863 | 1.450551 | 0.000000000000 | 0.000000000000 |
| ENSG00000163395 | 2.458775 | 9.658869 | -1.97391 | 0.000000000000 | 0.000000000000 |
| ENSG00000122545 | 30.83407 | 71.59309 | -1.21529 | 0.000000000000 | 0.000000000000 |
| ENSG00000100292 | 61.76481 | 20.00753 | 1.626242 | 0.000000000000 | 0.000000000000 |
| ENSG00000055044 | 9.780236 | 41.44667 | -2.08331 | 0.000000000000 | 0.000000000000 |
| ENSG00000149100 | 14.14508 | 62.62353 | -2.1464 | 0.000000000000 | 0.000000000000 |
| ENSG00000137970 | 1.495529 | 49.17199 | -5.03911 | 0.000000000000 | 0.000000000000 |
| ENSG00000197965 | 34.94259 | 81.88437 | -1.2286 | 0.000000000000 | 0.000000000000 |
| ENSG00000280407 | 19.12475 | 2.744271 | 2.800946 | 0.000000000000 | 0.000000000000 |
| ENSG00000063322 | 16.0202 | 44.09955 | -1.46087 | 0.000000000000 | 0.000000000000 |
| ENSG00000155304 | 4.162543 | 18.37292 | -2.14204 | 0.000000000000 | 0.000000000000 |
| ENSG00000141750 | 18.90302 | 3.689662 | 2.357056 | 0.000000000000 | 0.000000000000 |
| ENSG00000087263 | 13.58585 | 33.30821 | -1.29377 | 0.000000000000 | 0.000000000000 |
| ENSG00000170606 | 18.62194 | 42.85202 | -1.20236 | 0.000000000000 | 0.000000000000 |
| ENSG00000265808 | 15.6096 | 31.53557 | -1.01455 | 0.000000000000 | 0.000000000000 |
| ENSG00000074855 | 42.6733 | 17.43886 | 1.291028 | 0.000000000000 | 0.000000000000 |
| ENSG00000106991 | 70.40495 | 35.015 | 1.007704 | 0.000000000000 | 0.000000000000 |
| ENSG00000197172 | 24.31321 | 0.198212 | 6.938552 | 0.000000000000 | 0.000000000000 |
| ENSG00000167635 | 9.865224 | 29.16063 | -1.5636 | 0.000000000000 | 0.000000000000 |
| ENSG00000183579 | 20.73971 | 8.792933 | 1.237979 | 0.000000000000 | 0.000000000000 |
| ENSG00000179304 | 78.49545 | 38.43723 | 1.030105 | 0.000000000000 | 0.000000000000 |
| ENSG00000070087 | 43.8109 | 118.2781 | -1.43282 | 0.000000000000 | 0.000000000000 |
| ENSG00000168079 | 28.90919 | 6.058816 | 2.25442 | 0.000000000000 | 0.000000000000 |
| ENSG00000196954 | 35.75182 | 119.2014 | -1.73731 | 0.000000000000 | 0.000000000000 |
| ENSG00000110013 | 37.65014 | 13.2683 | 1.504672 | 0.000000000000 | 0.000000000000 |
| ENSG00000116489 | 14.87205 | 56.54644 | -1.92683 | 0.000000000000 | 0.000000000000 |
| ENSG00000067167 | 27.14842 | 58.27485 | -1.10201 | 0.000000000000 | 0.000000000000 |
| ENSG00000108175 | 18.48546 | 7.678784 | 1.267441 | 0.000000000000 | 0.000000000000 |
| ENSG00000001617 | 23.71743 | 54.6382 | -1.20396 | 0.000000000000 | 0.000000000000 |
| ENSG00000008952 | 4.529009 | 33.92996 | -2.90529 | 0.000000000000 | 0.000000000000 |
| ENSG00000214049 | 21.6377 | 59.95556 | -1.47035 | 0.000000000000 | 0.000000000000 |
| ENSG00000046604 | 23.61399 | 49.86422 | -1.07836 | 0.000000000000 | 0.000000000000 |
| ENSG00000204392 | 80.94096 | 181.0248 | -1.16125 | 0.000000000000 | 0.000000000000 |
| ENSG00000173653 | 116.3799 | 45.33638 | 1.3601 | 0.000000000000 | 0.000000000000 |
| ENSG00000112699 | 85.71909 | 36.98707 | 1.212595 | 0.000000000000 | 0.000000000000 |
| ENSG00000149196 | 5.362936 | 60.50854 | -3.49604 | 0.000000000000 | 0.000000000000 |
| ENSG00000280987 | 6.755331 | 19.83568 | -1.554 | 0.000000000000 | 0.000000000000 |
| ENSG00000090975 | 28.85267 | 13.53675 | 1.091823 | 0.000000000000 | 0.000000000000 |
| ENSG00000179583 | 5.621894 | 1.005348 | 2.483361 | 0.000000000000 | 0.000000000000 |
| ENSG00000171241 | 23.18189 | 52.84729 | -1.18883 | 0.000000000000 | 0.000000000000 |
| ENSG00000237506 | 39.14556 | 115.9641 | -1.56676 | 0.000000000000 | 0.000000000000 |
| ENSG00000261115 | 6.75215 | 1.834959 | 1.879599 | 0.000000000000 | 0.000000000000 |
| ENSG00000124839 | 117.6286 | 56.67548 | 1.053442 | 0.000000000000 | 0.000000000000 |
| ENSG00000185591 | 114.6617 | 44.64097 | 1.360943 | 0.000000000000 | 0.000000000000 |
| ENSG00000120437 | 22.73236 | 51.15488 | -1.17012 | 0.000000000000 | 0.000000000000 |
| ENSG00000070961 | 9.230935 | 21.39021 | -1.2124 | 0.000000000000 | 0.000000000000 |
| ENSG00000134057 | 67.06211 | 144.7412 | -1.10991 | 0.000000000000 | 0.000000000000 |
| ENSG00000166710 | 53.36969 | 117.3224 | -1.13639 | 0.000000000000 | 0.000000000000 |
| ENSG00000223573 | 36.73408 | 15.89812 | 1.208263 | 0.000000000000 | 0.000000000000 |
| ENSG00000176020 | 0.302227 | 10.65485 | -5.13973 | 0.000000000000 | 0.000000000000 |
| ENSG00000162702 | 1.963827 | 11.87742 | -2.59648 | 0.000000000000 | 0.000000000000 |
| ENSG00000168874 | 61.0489 | 28.11296 | 1.11873 | 0.000000000000 | 0.000000000000 |
| ENSG00000205323 | 17.93865 | 71.41816 | -1.99322 | 0.000000000000 | 0.000000000000 |
| ENSG00000114120 | 22.23836 | 72.31651 | -1.70127 | 0.000000000000 | 0.000000000000 |
| ENSG00000116717 | 16.10521 | 59.26122 | -1.87956 | 0.000000000000 | 0.000000000000 |
| ENSG00000119326 | 19.06171 | 48.08033 | -1.33477 | 0.000000000000 | 0.000000000000 |
| ENSG00000119917 | 23.88318 | 55.91425 | -1.22722 | 0.000000000000 | 0.000000000000 |
| ENSG00000197971 | 5.58958 | 12.86357 | -1.20248 | 0.000000000000 | 0.000000000000 |
| ENSG00000070669 | 24.73806 | 70.20199 | -1.50478 | 0.000000000000 | 0.000000000000 |
| ENSG00000173064 | 48.99681 | 20.48497 | 1.258122 | 0.000000000000 | 0.000000000000 |
| ENSG00000258790 | 0.815267 | 9.414044 | -3.52947 | 0.000000000000 | 0.000000000000 |
| ENSG00000135392 | 50.43919 | 17.0773 | 1.562465 | 0.000000000000 | 0.000000000000 |
| ENSG00000111684 | 61.16312 | 26.94649 | 1.182565 | 0.000000000000 | 0.000000000000 |
| ENSG00000113558 | 14.06721 | 104.6955 | -2.89579 | 0.000000000000 | 0.000000000000 |
| ENSG00000137285 | 0.066018 | 15.9355 | -7.91517 | 0.000000000000 | 0.000000000000 |
| ENSG00000135318 | 4.062657 | 17.59933 | -2.11503 | 0.000000000000 | 0.000000000000 |
| ENSG00000167106 | 32.10283 | 14.1629 | 1.180584 | 0.000000000000 | 0.000000000000 |
| ENSG00000077097 | 4.162055 | 13.33589 | -1.67995 | 0.000000000000 | 0.000000000000 |
| ENSG00000126804 | 8.311237 | 17.73613 | -1.09356 | 0.000000000000 | 0.000000000000 |
| ENSG00000280071 | 76.9287 | 24.44698 | 1.653865 | 0.000000000000 | 0.000000000000 |
| ENSG00000189339 | 52.14873 | 25.47208 | 1.033716 | 0.000000000000 | 0.000000000000 |
| ENSG00000057294 | 22.0056 | 45.95919 | -1.06248 | 0.000000000000 | 0.000000000000 |
| ENSG00000168958 | 61.48852 | 128.2482 | -1.06055 | 0.000000000000 | 0.000000000000 |
| ENSG00000163655 | 10.84268 | 41.42372 | -1.93374 | 0.000000000000 | 0.000000000000 |
| ENSG00000210144 | 2100.01 | 935.6816 | 1.166306 | 0.000000000000 | 0.000000000000 |
| ENSG00000166454 | 10.09104 | 25.41732 | -1.33274 | 0.000000000000 | 0.000000000000 |
| ENSG00000108055 | 9.873922 | 26.14446 | -1.40481 | 0.000000000000 | 0.000000000000 |
| ENSG00000169504 | 11.78788 | 24.44108 | -1.052 | 0.000000000000 | 0.000000000000 |
| ENSG00000158050 | 54.31991 | 16.3904 | 1.72863 | 0.000000000000 | 0.000000000000 |
| ENSG00000116062 | 12.00066 | 29.88991 | -1.31654 | 0.000000000000 | 0.000000000000 |
| ENSG00000144867 | 24.83172 | 54.9056 | -1.14477 | 0.000000000000 | 0.000000000000 |
| ENSG00000138398 | 9.050264 | 20.81992 | -1.20193 | 0.000000000000 | 0.000000000000 |
| ENSG00000152104 | 39.62968 | 19.41744 | 1.029228 | 0.000000000000 | 0.000000000000 |
| ENSG00000074527 | 11.95656 | 35.01678 | -1.55024 | 0.000000000000 | 0.000000000000 |
| ENSG00000178974 | 3.369972 | 15.35804 | -2.18819 | 0.000000000000 | 0.000000000000 |
| ENSG00000168813 | 2.391608 | 8.0395 | -1.74912 | 0.000000000000 | 0.000000000000 |
| ENSG00000284526 | 5.096903 | 0.032991 | 7.271405 | 0.000000000000 | 0.000000000000 |
| ENSG00000278189 | 282.9572 | 43.87384 | 2.689151 | 0.000000000000 | 0.000000000000 |
| ENSG00000088766 | 1.076035 | 10.01949 | -3.21901 | 0.000000000000 | 0.000000000000 |
| ENSG00000123933 | 52.0146 | 23.07427 | 1.172632 | 0.000000000000 | 0.000000000000 |
| ENSG00000185838 | 37.55559 | 15.26697 | 1.298614 | 0.000000000000 | 0.000000000000 |
| ENSG00000170779 | 37.05384 | 12.84001 | 1.528976 | 0.000000000000 | 0.000000000000 |
| ENSG00000126698 | 38.09597 | 82.25044 | -1.11039 | 0.000000000000 | 0.000000000000 |
| ENSG00000082212 | 1.383584 | 6.786643 | -2.29429 | 0.000000000000 | 0.000000000000 |
| ENSG00000121892 | 5.608718 | 16.02717 | -1.51478 | 0.000000000000 | 0.000000000000 |
| ENSG00000223764 | 13.85379 | 3.308716 | 2.065937 | 0.000000000000 | 0.000000000000 |
| ENSG00000138071 | 19.88149 | 49.29741 | -1.31009 | 0.000000000000 | 0.000000000000 |
| ENSG00000235552 | 11.44408 | 57.50403 | -2.32906 | 0.000000000000 | 0.000000000000 |
| ENSG00000145386 | 12.63474 | 34.36887 | -1.44371 | 0.000000000000 | 0.000000000000 |
| ENSG00000138180 | 6.169868 | 23.95056 | -1.95675 | 0.000000000000 | 0.000000000000 |
| ENSG00000198856 | 11.0513 | 45.93932 | -2.05551 | 0.000000000000 | 0.000000000000 |
| ENSG00000151247 | 1.593147 | 21.36752 | -3.74547 | 0.000000000000 | 0.000000000000 |
| ENSG00000143771 | 15.233 | 33.82756 | -1.151 | 0.000000000000 | 0.000000000000 |
| ENSG00000170677 | 0.455192 | 5.74177 | -3.65695 | 0.000000000000 | 0.000000000000 |
| ENSG00000166260 | 37.05967 | 5.20421 | 2.832099 | 0.000000000000 | 0.000000000000 |
| ENSG00000101773 | 4.991886 | 20.09654 | -2.00929 | 0.000000000000 | 0.000000000000 |
| ENSG00000111845 | 11.81155 | 39.5434 | -1.74324 | 0.000000000000 | 0.000000000000 |
| ENSG00000143401 | 4.712695 | 26.29921 | -2.4804 | 0.000000000000 | 0.000000000000 |
| ENSG00000241258 | 30.12511 | 6.472274 | 2.218622 | 0.000000000000 | 0.000000000000 |
| ENSG00000103260 | 47.45161 | 20.88909 | 1.183707 | 0.000000000000 | 0.000000000000 |
| ENSG00000276612 | 2.036373 | 20.71474 | -3.34658 | 0.000000000000 | 0.000000000000 |
| ENSG00000183696 | 23.99577 | 65.20166 | -1.44213 | 0.000000000000 | 0.000000000000 |
| ENSG00000146072 | 4.787286 | 17.59484 | -1.87787 | 0.000000000000 | 0.000000000000 |
| ENSG00000180537 | 21.99578 | 44.6562 | -1.02163 | 0.000000000000 | 0.000000000000 |
| ENSG00000107201 | 2.968483 | 12.62329 | -2.08829 | 0.000000000000 | 0.000000000000 |
| ENSG00000136758 | 11.68005 | 30.83058 | -1.40031 | 0.000000000000 | 0.000000000000 |
| ENSG00000173166 | 9.755888 | 3.470331 | 1.4912 | 0.000000000000 | 0.000000000000 |
| ENSG00000003436 | 31.36945 | 65.58568 | -1.06402 | 0.000000000000 | 0.000000000000 |
| ENSG00000163001 | 2.01512 | 25.17724 | -3.64318 | 0.000000000000 | 0.000000000000 |
| ENSG00000148426 | 19.22532 | 59.82249 | -1.63768 | 0.000000000000 | 0.000000000000 |
| ENSG00000114209 | 7.14404 | 45.22402 | -2.66228 | 0.000000000000 | 0.000000000000 |
| ENSG00000167740 | 72.00986 | 29.27899 | 1.298329 | 0.000000000000 | 0.000000000000 |
| ENSG00000129219 | 59.13397 | 25.65051 | 1.205 | 0.000000000000 | 0.000000000000 |
| ENSG00000213949 | 8.95881 | 18.01652 | -1.00794 | 0.000000000000 | 0.000000000000 |
| ENSG00000172965 | 56.16071 | 149.9291 | -1.41665 | 0.000000000000 | 0.000000000000 |
| ENSG00000270181 | 3.011807 | 11.56017 | -1.94046 | 0.000000000000 | 0.000000000000 |
| ENSG00000164171 | 4.177587 | 12.95225 | -1.63246 | 0.000000000000 | 0.000000000000 |
| ENSG00000137509 | 19.60016 | 65.96331 | -1.7508 | 0.000000000000 | 0.000000000000 |
| ENSG00000176907 | 1.99517 | 18.08891 | -3.18052 | 0.000000000000 | 0.000000000000 |
| ENSG00000170852 | 10.0723 | 31.41823 | -1.64121 | 0.000000000000 | 0.000000000000 |
| ENSG00000083168 | 3.395436 | 9.203712 | -1.43862 | 0.000000000000 | 0.000000000000 |
| ENSG00000106028 | 51.93184 | 138.6004 | -1.41624 | 0.000000000000 | 0.000000000000 |
| ENSG00000210135 | 1956.37 | 944.7426 | 1.050186 | 0.000000000000 | 0.000000000000 |
| ENSG00000132432 | 24.02777 | 96.18842 | -2.00116 | 0.000000000000 | 0.000000000000 |
| ENSG00000169607 | 3.726229 | 13.83987 | -1.89304 | 0.000000000000 | 0.000000000000 |
| ENSG00000262160 | 7.743401 | 0.23642 | 5.033543 | 0.000000000000 | 0.000000000000 |
| ENSG00000118777 | 11.17109 | 34.80579 | -1.63956 | 0.000000000000 | 0.000000000000 |
| ENSG00000065548 | 7.017502 | 26.63665 | -1.92438 | 0.000000000000 | 0.000000000000 |
| ENSG00000262304 | 0.220003 | 5.319112 | -4.59559 | 0.000000000000 | 0.000000000000 |
| ENSG00000141198 | 0.0001 | 26.91113 | -18.0378 | 0.000000000000 | 0.000000000000 |
| ENSG00000159063 | 44.64194 | 103.2046 | -1.20904 | 0.000000000000 | 0.000000000000 |
| ENSG00000283515 | 0.95951 | 9.956838 | -3.37532 | 0.000000000000 | 0.000000000000 |
| ENSG00000181061 | 22.03553 | 71.76537 | -1.70346 | 0.000000000000 | 0.000000000000 |
| ENSG00000134001 | 26.95764 | 62.14719 | -1.205 | 0.000000000000 | 0.000000000000 |
| ENSG00000074590 | 3.644 | 10.90112 | -1.58088 | 0.000000000000 | 0.000000000000 |
| ENSG00000138685 | 8.839744 | 21.62127 | -1.29037 | 0.000000000000 | 0.000000000000 |
| ENSG00000171219 | 14.79876 | 5.733007 | 1.368112 | 0.000000000000 | 0.000000000000 |
| ENSG00000131778 | 34.4454 | 71.17001 | -1.04696 | 0.000000000000 | 0.000000000000 |
| ENSG00000127399 | 203.0242 | 96.09034 | 1.079188 | 0.000000000000 | 0.000000000000 |
| ENSG00000257390 | 1.152595 | 15.12403 | -3.71388 | 0.000000000000 | 0.000000000000 |
| ENSG00000167088 | 5.899196 | 21.00029 | -1.83182 | 0.000000000000 | 0.000000000000 |
| ENSG00000196214 | 0.994328 | 5.998098 | -2.59271 | 0.000000000000 | 0.000000000000 |
| ENSG00000261915 | 12.08262 | 0.001705 | 12.79087 | 0.000000000000 | 0.000000000000 |
| ENSG00000123444 | 24.87584 | 9.425426 | 1.400115 | 0.000000000000 | 0.000000000000 |
| ENSG00000154229 | 18.0924 | 8.994262 | 1.008307 | 0.000000000000 | 0.000000000000 |
| ENSG00000138134 | 11.72162 | 38.77121 | -1.72581 | 0.000000000000 | 0.000000000000 |
| ENSG00000172845 | 8.346025 | 39.00116 | -2.22436 | 0.000000000000 | 0.000000000000 |
| ENSG00000156502 | 17.59865 | 42.84012 | -1.2835 | 0.000000000000 | 0.000000000000 |
| ENSG00000271447 | 45.32612 | 19.03827 | 1.251441 | 0.000000000000 | 0.000000000000 |
| ENSG00000187017 | 23.32501 | 3.391159 | 2.782027 | 0.000000000000 | 0.000000000000 |
| ENSG00000092295 | 8.525472 | 24.61626 | -1.52976 | 0.000000000000 | 0.000000000000 |
| ENSG00000198406 | 3.124154 | 34.56565 | -3.4678 | 0.000000000000 | 0.000000000000 |
| ENSG00000136048 | 22.02974 | 45.93352 | -1.06009 | 0.000000000000 | 0.000000000000 |
| ENSG00000164086 | 10.58961 | 27.01135 | -1.35092 | 0.000000000000 | 0.000000000000 |
| ENSG00000121578 | 16.26015 | 57.26269 | -1.81625 | 0.000000000000 | 0.000000000000 |
| ENSG00000040275 | 8.311633 | 25.40388 | -1.61184 | 0.000000000000 | 0.000000000000 |
| ENSG00000275215 | 238.6765 | 38.84138 | 2.61939 | 0.000000000000 | 0.000000000000 |
| ENSG00000126878 | 24.51045 | 9.876076 | 1.311387 | 0.000000000000 | 0.000000000000 |
| ENSG00000033327 | 7.453468 | 17.65492 | -1.24409 | 0.000000000000 | 0.000000000000 |
| ENSG00000157216 | 158.1214 | 68.02232 | 1.216952 | 0.000000000000 | 0.000000000000 |
| ENSG00000116473 | 1.8681 | 19.0263 | -3.34835 | 0.000000000000 | 0.000000000000 |
| ENSG00000108786 | 9.343993 | 3.399164 | 1.458859 | 0.000000000000 | 0.000000000000 |
| ENSG00000136045 | 23.36597 | 58.79974 | -1.3314 | 0.000000000000 | 0.000000000000 |
| ENSG00000244716 | 61.82515 | 157.5494 | -1.34954 | 0.000000000000 | 0.000000000000 |
| ENSG00000139624 | 17.32913 | 47.55471 | -1.45639 | 0.000000000000 | 0.000000000000 |
| ENSG00000122705 | 48.87634 | 106.0141 | -1.11705 | 0.000000000000 | 0.000000000000 |
| ENSG00000160124 | 10.51292 | 61.65715 | -2.5521 | 0.000000000000 | 0.000000000000 |
| ENSG00000137414 | 16.5268 | 6.190029 | 1.41679 | 0.000000000000 | 0.000000000000 |
| ENSG00000162772 | 6.764602 | 29.26742 | -2.11322 | 0.000000000000 | 0.000000000000 |
| ENSG00000281490 | 20.95515 | 6.252997 | 1.744685 | 0.000000000000 | 0.000000000000 |
| ENSG00000085274 | 0.67029 | 8.071925 | -3.59006 | 0.000000000000 | 0.000000000000 |
| ENSG00000082497 | 2.773505 | 16.71874 | -2.59168 | 0.000000000000 | 0.000000000000 |
| ENSG00000136824 | 4.683483 | 14.55523 | -1.63588 | 0.000000000000 | 0.000000000000 |
| ENSG00000120699 | 8.495719 | 38.63969 | -2.18528 | 0.000000000000 | 0.000000000000 |
| ENSG00000165304 | 17.88444 | 43.54407 | -1.28377 | 0.000000000000 | 0.000000000000 |
| ENSG00000118217 | 9.429653 | 18.87216 | -1.00098 | 0.000000000000 | 0.000000000000 |
| ENSG00000180229 | 42.8536 | 18.93975 | 1.177999 | 0.000000000000 | 0.000000000000 |
| ENSG00000171055 | 12.42986 | 39.68581 | -1.67481 | 0.000000000000 | 0.000000000000 |
| ENSG00000136051 | 2.051977 | 13.06481 | -2.6706 | 0.000000000000 | 0.000000000000 |
| ENSG00000112062 | 8.074983 | 20.13069 | -1.31787 | 0.000000000000 | 0.000000000000 |
| ENSG00000100764 | 22.89308 | 50.0425 | -1.12824 | 0.000000000000 | 0.000000000000 |
| ENSG00000111237 | 34.43421 | 84.73276 | -1.29908 | 0.000000000000 | 0.000000000000 |
| ENSG00000121621 | 0.974706 | 7.085891 | -2.86191 | 0.000000000000 | 0.000000000000 |
| ENSG00000050130 | 3.536298 | 18.03078 | -2.35015 | 0.000000000000 | 0.000000000000 |
| ENSG00000225339 | 14.12705 | 3.187549 | 2.147941 | 0.000000000000 | 0.000000000000 |
| ENSG00000108825 | 6.40715 | 22.68096 | -1.82373 | 0.000000000000 | 0.000000000000 |
| ENSG00000157540 | 32.37679 | 12.82028 | 1.336532 | 0.000000000000 | 0.000000000000 |
| ENSG00000165804 | 50.26088 | 19.52876 | 1.363835 | 0.000000000000 | 0.000000000000 |
| ENSG00000151012 | 2.467477 | 7.134935 | -1.53186 | 0.000000000000 | 0.000000000000 |
| ENSG00000250644 | 31.15145 | 94.48025 | -1.60071 | 0.000000000000 | 0.000000000000 |
| ENSG00000104899 | 65.92786 | 30.34304 | 1.119523 | 0.000000000000 | 0.000000000000 |
| ENSG00000125629 | 5.288361 | 23.46912 | -2.14987 | 0.000000000000 | 0.000000000000 |
| ENSG00000065183 | 14.21094 | 31.05176 | -1.12767 | 0.000000000000 | 0.000000000000 |
| ENSG00000128585 | 5.343977 | 12.63489 | -1.24143 | 0.000000000000 | 0.000000000000 |
| ENSG00000121741 | 1.279213 | 9.198344 | -2.84612 | 0.000000000000 | 0.000000000000 |
| ENSG00000155287 | 69.27577 | 24.07488 | 1.524822 | 0.000000000000 | 0.000000000000 |
| ENSG00000170242 | 3.42858 | 12.44038 | -1.85935 | 0.000000000000 | 0.000000000000 |
| ENSG00000136522 | 12.0239 | 39.7272 | -1.72422 | 0.000000000000 | 0.000000000000 |
| ENSG00000005893 | 21.40525 | 48.35239 | -1.17562 | 0.000000000000 | 0.000000000000 |
| ENSG00000171792 | 25.21364 | 70.34204 | -1.48018 | 0.000000000000 | 0.000000000000 |
| ENSG00000186432 | 5.036266 | 11.57245 | -1.20027 | 0.000000000000 | 0.000000000000 |
| ENSG00000055208 | 7.233902 | 16.75978 | -1.21216 | 0.000000000000 | 0.000000000000 |
| ENSG00000075826 | 30.25985 | 13.64451 | 1.149085 | 0.000000000000 | 0.000000000000 |
| ENSG00000125676 | 6.746335 | 22.67491 | -1.74892 | 0.000000000000 | 0.000000000000 |
| ENSG00000147996 | 4.499243 | 26.03231 | -2.53255 | 0.000000000000 | 0.000000000000 |
| ENSG00000048707 | 16.78815 | 6.22477 | 1.431351 | 0.000000000000 | 0.000000000000 |
| ENSG00000156802 | 12.07916 | 26.0428 | -1.10836 | 0.000000000000 | 0.000000000000 |
| ENSG00000128165 | 4.529263 | 14.29593 | -1.65826 | 0.000000000000 | 0.000000000000 |
| ENSG00000185298 | 16.23594 | 33.78523 | -1.0572 | 0.000000000000 | 0.000000000000 |
| ENSG00000213281 | 5.28434 | 15.00372 | -1.50552 | 0.000000000000 | 0.000000000000 |
| ENSG00000198954 | 5.451078 | 21.16773 | -1.95725 | 0.000000000000 | 0.000000000000 |
| ENSG00000166037 | 4.993805 | 25.44695 | -2.34928 | 0.000000000000 | 0.000000000000 |
| ENSG00000168685 | 0.345398 | 7.602631 | -4.46017 | 0.000000000000 | 0.000000000000 |
| ENSG00000135049 | 1.059162 | 8.420228 | -2.99094 | 0.000000000000 | 0.000000000000 |
| ENSG00000123130 | 28.23431 | 61.19857 | -1.11605 | 0.000000000000 | 0.000000000000 |
| ENSG00000180263 | 3.84037 | 10.22629 | -1.41297 | 0.000000000000 | 0.000000000000 |
| ENSG00000109445 | 21.55747 | 59.56202 | -1.4662 | 0.000000000000 | 0.000000000000 |
| ENSG00000089685 | 153.6963 | 61.0618 | 1.33174 | 0.000000000000 | 0.000000000000 |
| ENSG00000008282 | 7.160927 | 24.21856 | -1.75789 | 0.000000000000 | 0.000000000000 |
| ENSG00000185009 | 12.52363 | 33.15265 | -1.40447 | 0.000000000000 | 0.000000000000 |
| ENSG00000064102 | 15.29567 | 33.02337 | -1.11036 | 0.000000000000 | 0.000000000000 |
| ENSG00000168476 | 24.60094 | 55.54562 | -1.17496 | 0.000000000000 | 0.000000000000 |
| ENSG00000166233 | 6.03018 | 18.19651 | -1.59339 | 0.000000000000 | 0.000000000000 |
| ENSG00000127870 | 6.183969 | 14.96416 | -1.27491 | 0.000000000000 | 0.000000000000 |
| ENSG00000120694 | 7.570829 | 24.10471 | -1.67079 | 0.000000000000 | 0.000000000000 |
| ENSG00000259781 | 14.96413 | 63.12978 | -2.07681 | 0.000000000000 | 0.000000000000 |
| ENSG00000108651 | 22.31029 | 54.35366 | -1.28467 | 0.000000000000 | 0.000000000000 |
| ENSG00000081692 | 6.84615 | 22.83295 | -1.73775 | 0.000000000000 | 0.000000000000 |
| ENSG00000265735 | 207.7871 | 77.49844 | 1.422867 | 0.000000000000 | 0.000000000000 |
| ENSG00000075188 | 7.171565 | 33.9463 | -2.24289 | 0.000000000000 | 0.000000000000 |
| ENSG00000152683 | 0.43689 | 7.115268 | -4.02558 | 0.000000000000 | 0.000000000000 |
| ENSG00000176046 | 34.59758 | 73.80618 | -1.09307 | 0.000000000000 | 0.000000000000 |
| ENSG00000120254 | 32.13907 | 71.09076 | -1.14533 | 0.000000000000 | 0.000000000000 |
| ENSG00000100519 | 6.091486 | 23.06489 | -1.92083 | 0.000000000000 | 0.000000000000 |
| ENSG00000147601 | 2.724468 | 9.76771 | -1.84205 | 0.000000000000 | 0.000000000000 |
| ENSG00000115255 | 38.1147 | 14.39663 | 1.404616 | 0.000000000000 | 0.000000000000 |
| ENSG00000154710 | 13.84879 | 28.23841 | -1.0279 | 0.000000000000 | 0.000000000000 |
| ENSG00000143390 | 68.16561 | 28.97918 | 1.234028 | 0.000000000000 | 0.000000000000 |
| ENSG00000219507 | 7.032102 | 52.45718 | -2.89911 | 0.000000000000 | 0.000000000000 |
| ENSG00000272068 | 27.33939 | 12.16899 | 1.167771 | 0.000000000000 | 0.000000000000 |
| ENSG00000210196 | 7.070919 | 274.6844 | -5.27973 | 0.000000000000 | 0.000000000000 |
| ENSG00000133398 | 38.14672 | 82.85012 | -1.11894 | 0.000000000000 | 0.000000000000 |
| ENSG00000079134 | 8.542314 | 21.98523 | -1.36384 | 0.000000000000 | 0.000000000000 |
| ENSG00000128708 | 4.4923 | 22.43377 | -2.32015 | 0.000000000000 | 0.000000000000 |
| ENSG00000101558 | 37.07375 | 86.60703 | -1.22409 | 0.000000000000 | 0.000000000000 |
| ENSG00000187555 | 20.75972 | 42.50345 | -1.03379 | 0.000000000000 | 0.000000000000 |
| ENSG00000159873 | 1.886813 | 8.455196 | -2.16389 | 0.000000000000 | 0.000000000000 |
| ENSG00000164048 | 4.990307 | 13.90266 | -1.47816 | 0.000000000000 | 0.000000000000 |
| ENSG00000188766 | 12.74393 | 5.077943 | 1.327494 | 0.000000000000 | 0.000000000000 |
| ENSG00000136213 | 25.53712 | 11.42971 | 1.159807 | 0.000000000000 | 0.000000000000 |
| ENSG00000188529 | 24.35719 | 53.6275 | -1.13863 | 0.000000000000 | 0.000000000000 |
| ENSG00000257341 | 25.33243 | 79.29115 | -1.64617 | 0.000000000000 | 0.000000000000 |
| ENSG00000130779 | 72.61836 | 34.10865 | 1.090197 | 0.000000000000 | 0.000000000000 |
| ENSG00000061676 | 4.518252 | 11.97942 | -1.40672 | 0.000000000000 | 0.000000000000 |
| ENSG00000020256 | 24.3872 | 9.743739 | 1.323577 | 0.000000000000 | 0.000000000000 |
| ENSG00000100883 | 12.26516 | 30.96591 | -1.33611 | 0.000000000000 | 0.000000000000 |
| ENSG00000119414 | 8.063974 | 18.61988 | -1.20728 | 0.000000000000 | 0.000000000000 |
| ENSG00000085721 | 11.83587 | 35.07187 | -1.56715 | 0.000000000000 | 0.000000000000 |
| ENSG00000170296 | 97.26527 | 38.70905 | 1.329254 | 0.000000000000 | 0.000000000000 |
| ENSG00000205302 | 12.19077 | 54.5764 | -2.16249 | 0.000000000000 | 0.000000000000 |
| ENSG00000198056 | 12.42433 | 37.86036 | -1.60752 | 0.000000000000 | 0.000000000000 |
| ENSG00000175745 | 55.44936 | 13.86415 | 1.999812 | 0.000000000000 | 0.000000000000 |
| ENSG00000079387 | 4.673079 | 11.99589 | -1.36009 | 0.000000000000 | 0.000000000000 |
| ENSG00000164163 | 6.200929 | 20.11069 | -1.69741 | 0.000000000000 | 0.000000000000 |
| ENSG00000243137 | 1.128092 | 20.30757 | -4.17006 | 0.000000000000 | 0.000000000000 |
| ENSG00000132823 | 55.82565 | 25.43351 | 1.134197 | 0.000000000000 | 0.000000000000 |
| ENSG00000092108 | 4.496368 | 25.14624 | -2.48351 | 0.000000000000 | 0.000000000000 |
| ENSG00000172667 | 18.72418 | 7.112667 | 1.39644 | 0.000000000000 | 0.000000000000 |
| ENSG00000198924 | 0.784738 | 5.694065 | -2.85918 | 0.000000000000 | 0.000000000000 |
| ENSG00000100814 | 14.07977 | 40.24072 | -1.51503 | 0.000000000000 | 0.000000000000 |
| ENSG00000180035 | 6.148777 | 16.54764 | -1.42825 | 0.000000000000 | 0.000000000000 |
| ENSG00000243477 | 38.30981 | 15.53697 | 1.302009 | 0.000000000000 | 0.000000000000 |
| ENSG00000047617 | 7.618532 | 26.37013 | -1.79132 | 0.000000000000 | 0.000000000000 |
| ENSG00000196670 | 9.758717 | 19.78252 | -1.01946 | 0.000000000000 | 0.000000000000 |
| ENSG00000146278 | 56.84452 | 27.03681 | 1.072097 | 0.000000000000 | 0.000000000000 |
| ENSG00000165030 | 20.16993 | 43.82395 | -1.11951 | 0.000000000000 | 0.000000000000 |
| ENSG00000051341 | 4.675596 | 11.65425 | -1.31763 | 0.000000000000 | 0.000000000000 |
| ENSG00000107338 | 15.98795 | 34.03703 | -1.09012 | 0.000000000000 | 0.000000000000 |
| ENSG00000087502 | 1.472659 | 7.660475 | -2.37901 | 0.000000000000 | 0.000000000000 |
| ENSG00000248592 | 11.35233 | 0.129654 | 6.452178 | 0.000000000000 | 0.000000000000 |
| ENSG00000152377 | 9.655465 | 21.691 | -1.16768 | 0.000000000000 | 0.000000000000 |
| ENSG00000125841 | 27.50622 | 12.28583 | 1.162763 | 0.000000000000 | 0.000000000000 |
| ENSG00000167964 | 56.66194 | 24.50827 | 1.209112 | 0.000000000000 | 0.000000000000 |
| ENSG00000165887 | 5.292754 | 23.67719 | -2.16141 | 0.000000000000 | 0.000000000000 |
| ENSG00000213977 | 31.16969 | 66.82596 | -1.10026 | 0.000000000000 | 0.000000000000 |
| ENSG00000019995 | 2.575822 | 8.387729 | -1.70325 | 0.000000000000 | 0.000000000000 |
| ENSG00000175592 | 15.01385 | 42.37821 | -1.49703 | 0.000000000000 | 0.000000000000 |
| ENSG00000139263 | 3.63353 | 11.63299 | -1.67878 | 0.000000000000 | 0.000000000000 |
| ENSG00000244165 | 35.68121 | 15.7345 | 1.181233 | 0.000000000000 | 0.000000000000 |
| ENSG00000137965 | 7.715768 | 32.61687 | -2.07974 | 0.000000000000 | 0.000000000000 |
| ENSG00000090006 | 36.64506 | 17.71954 | 1.048278 | 0.000000000000 | 0.000000000000 |
| ENSG00000106479 | 26.96705 | 9.216502 | 1.548906 | 0.000000000000 | 0.000000000000 |
| ENSG00000112742 | 0.145629 | 5.925416 | -5.34655 | 0.000000000000 | 0.000000000000 |
| ENSG00000123472 | 5.372679 | 27.06625 | -2.33278 | 0.000000000000 | 0.000000000000 |
| ENSG00000180901 | 7.984428 | 18.97349 | -1.24872 | 0.000000000000 | 0.000000000000 |
| ENSG00000175832 | 18.5025 | 45.8654 | -1.30969 | 0.000000000000 | 0.000000000000 |
| ENSG00000062194 | 5.388926 | 18.7066 | -1.79548 | 0.000000000000 | 0.000000000000 |
| ENSG00000165195 | 3.794178 | 14.43361 | -1.92757 | 0.000000000000 | 0.000000000000 |
| ENSG00000078401 | 3.679716 | 16.50404 | -2.16515 | 0.000000000000 | 0.000000000000 |
| ENSG00000108511 | 30.51768 | 10.68962 | 1.513435 | 0.000000000000 | 0.000000000000 |
| ENSG00000108559 | 52.1991 | 127.298 | -1.28611 | 0.000000000000 | 0.000000000000 |
| ENSG00000138614 | 15.78803 | 40.56355 | -1.36135 | 0.000000000000 | 0.000000000000 |
| ENSG00000128185 | 80.67717 | 39.07622 | 1.04587 | 0.000000000000 | 0.000000000000 |
| ENSG00000144895 | 21.09326 | 45.34486 | -1.10416 | 0.000000000000 | 0.000000000000 |
| ENSG00000076043 | 48.20557 | 103.6274 | -1.10413 | 0.000000000000 | 0.000000000000 |
| ENSG00000185104 | 17.04797 | 38.62826 | -1.18006 | 0.000000000000 | 0.000000000000 |
| ENSG00000284292 | 20.19897 | 42.75087 | -1.08167 | 0.000000000000 | 0.000000000000 |
| ENSG00000080822 | 13.46002 | 49.40996 | -1.87612 | 0.000000000000 | 0.000000000000 |
| ENSG00000167528 | 7.646211 | 24.4597 | -1.67759 | 0.000000000000 | 0.000000000000 |
| ENSG00000164024 | 11.97519 | 28.3057 | -1.24104 | 0.000000000000 | 0.000000000000 |
| ENSG00000106714 | 12.96859 | 3.171883 | 2.03161 | 0.000000000000 | 0.000000000000 |
| ENSG00000251022 | 11.82183 | 28.51692 | -1.27036 | 0.000000000000 | 0.000000000000 |
| ENSG00000115738 | 4.118507 | 14.11651 | -1.77719 | 0.000000000000 | 0.000000000000 |
| ENSG00000104549 | 6.903007 | 19.57982 | -1.50407 | 0.000000000000 | 0.000000000000 |
| ENSG00000256967 | 23.39873 | 1.370288 | 4.093879 | 0.000000000000 | 0.000000000000 |
| ENSG00000143952 | 1.063448 | 9.992591 | -3.23211 | 0.000000000000 | 0.000000000000 |
| ENSG00000183801 | 8.66392 | 20.74291 | -1.25953 | 0.000000000000 | 0.000000000000 |
| ENSG00000081041 | 10.90273 | 38.30201 | -1.81273 | 0.000000000000 | 0.000000000000 |
| ENSG00000164080 | 6.403748 | 2.837808 | 1.17414 | 0.000000000000 | 0.000000000000 |
| ENSG00000147654 | 1.459255 | 10.08214 | -2.7885 | 0.000000000000 | 0.000000000000 |
| ENSG00000136143 | 7.112345 | 21.92411 | -1.62412 | 0.000000000000 | 0.000000000000 |
| ENSG00000099308 | 11.9197 | 5.518788 | 1.110925 | 0.000000000000 | 0.000000000000 |
| ENSG00000122970 | 1.637821 | 15.79927 | -3.27001 | 0.000000000000 | 0.000000000000 |
| ENSG00000133103 | 1.76886 | 8.633661 | -2.28715 | 0.000000000000 | 0.000000000000 |
| ENSG00000173320 | 6.259068 | 1.487811 | 2.072757 | 0.000000000000 | 0.000000000000 |
| ENSG00000057657 | 21.32145 | 9.547245 | 1.159149 | 0.000000000000 | 0.000000000000 |
| ENSG00000166477 | 10.56071 | 28.97751 | -1.45623 | 0.000000000000 | 0.000000000000 |
| ENSG00000204175 | 37.70655 | 16.74132 | 1.171402 | 0.000000000000 | 0.000000000000 |
| ENSG00000164253 | 6.401053 | 19.75689 | -1.62597 | 0.000000000000 | 0.000000000000 |
| ENSG00000166847 | 14.85154 | 43.48543 | -1.54992 | 0.000000000000 | 0.000000000000 |
| ENSG00000262580 | 62.6818 | 27.86691 | 1.169494 | 0.000000000000 | 0.000000000000 |
| ENSG00000113312 | 20.02682 | 48.40981 | -1.27337 | 0.000000000000 | 0.000000000000 |
| ENSG00000133104 | 9.58629 | 38.67801 | -2.01247 | 0.000000000000 | 0.000000000000 |
| ENSG00000148339 | 9.90022 | 22.36202 | -1.17552 | 0.000000000000 | 0.000000000000 |
| ENSG00000221995 | 4.562587 | 11.55238 | -1.34027 | 0.000000000000 | 0.000000000000 |
| ENSG00000115541 | 64.64272 | 143.486 | -1.15035 | 0.000000000000 | 0.000000000000 |
| ENSG00000129514 | 15.43679 | 3.289006 | 2.230649 | 0.000000000000 | 0.000000000000 |
| ENSG00000238266 | 5.122982 | 15.40596 | -1.58843 | 0.000000000000 | 0.000000000000 |
| ENSG00000100033 | 8.758092 | 23.39253 | -1.41736 | 0.000000000000 | 0.000000000000 |
| ENSG00000164327 | 2.981684 | 6.854448 | -1.20091 | 0.000000000000 | 0.000000000000 |
| ENSG00000150782 | 21.28783 | 49.33057 | -1.21245 | 0.000000000000 | 0.000000000000 |
| ENSG00000176386 | 33.12399 | 78.50171 | -1.24485 | 0.000000000000 | 0.000000000000 |
| ENSG00000171497 | 15.74535 | 38.13723 | -1.27627 | 0.000000000000 | 0.000000000000 |
| ENSG00000204941 | 0.563218 | 11.20667 | -4.31452 | 0.000000000000 | 0.000000000000 |
| ENSG00000004700 | 8.316978 | 19.31003 | -1.21522 | 0.000000000000 | 0.000000000000 |
| ENSG00000132963 | 4.15584 | 20.65692 | -2.31341 | 0.000000000000 | 0.000000000000 |
| ENSG00000198369 | 8.820159 | 20.19675 | -1.19525 | 0.000000000000 | 0.000000000000 |
| ENSG00000189057 | 2.353703 | 6.550898 | -1.47676 | 0.000000000000 | 0.000000000000 |
| ENSG00000153187 | 140.0247 | 297.3356 | -1.08641 | 0.000000000000 | 0.000000000000 |
| ENSG00000171848 | 51.79705 | 113.9768 | -1.1378 | 0.000000000000 | 0.000000000000 |
| ENSG00000184182 | 14.86035 | 39.65369 | -1.41599 | 0.000000000000 | 0.000000000000 |
| ENSG00000171109 | 6.784055 | 15.39043 | -1.18181 | 0.000000000000 | 0.000000000000 |
| ENSG00000186352 | 31.93353 | 74.19623 | -1.21627 | 0.000000000000 | 0.000000000000 |
| ENSG00000088205 | 14.8798 | 33.57543 | -1.17405 | 0.000000000000 | 0.000000000000 |
| ENSG00000239521 | 32.26295 | 13.62049 | 1.2441 | 0.000000000000 | 0.000000000000 |
| ENSG00000055955 | 10.12821 | 2.062194 | 2.296127 | 0.000000000000 | 0.000000000000 |
| ENSG00000243207 | 11.83278 | 27.82485 | -1.23358 | 0.000000000000 | 0.000000000000 |
| ENSG00000113552 | 17.4611 | 35.07475 | -1.00629 | 0.000000000000 | 0.000000000000 |
| ENSG00000214110 | 42.61151 | 87.09992 | -1.03143 | 0.000000000000 | 0.000000000000 |
| ENSG00000103995 | 5.783984 | 15.16535 | -1.39064 | 0.000000000000 | 0.000000000000 |
| ENSG00000231419 | 12.56834 | 4.926568 | 1.351139 | 0.000000000000 | 0.000000000000 |
| ENSG00000144909 | 3.859491 | 11.10224 | -1.52437 | 0.000000000000 | 0.000000000000 |
| ENSG00000226221 | 2.541242 | 36.84816 | -3.85799 | 0.000000000000 | 0.000000000000 |
| ENSG00000106538 | 21.07558 | 6.935234 | 1.603556 | 0.000000000000 | 0.000000000000 |
| ENSG00000138081 | 4.047565 | 14.67516 | -1.85825 | 0.000000000000 | 0.000000000000 |
| ENSG00000234797 | 0.314968 | 17.25008 | -5.77525 | 0.000000000000 | 0.000000000000 |
| ENSG00000139734 | 5.724959 | 13.68018 | -1.25675 | 0.000000000000 | 0.000000000000 |
| ENSG00000118523 | 0.574535 | 7.105779 | -3.62853 | 0.000000000000 | 0.000000000000 |
| ENSG00000137692 | 34.16252 | 12.19496 | 1.486129 | 0.000000000000 | 0.000000000000 |
| ENSG00000258674 | 74.44039 | 36.22708 | 1.039017 | 0.000000000000 | 0.000000000000 |
| ENSG00000082213 | 5.500267 | 17.33864 | -1.65642 | 0.000000000000 | 0.000000000000 |
| ENSG00000132938 | 5.61164 | 0.627374 | 3.161025 | 0.000000000000 | 0.000000000000 |
| ENSG00000158480 | 11.27256 | 4.012678 | 1.490178 | 0.000000000000 | 0.000000000000 |
| ENSG00000158106 | 27.93976 | 13.59898 | 1.038821 | 0.000000000000 | 0.000000000000 |
| ENSG00000164022 | 2.902126 | 11.63171 | -2.00288 | 0.000000000000 | 0.000000000000 |
| ENSG00000257671 | 10.74558 | 29.14229 | -1.43937 | 0.000000000000 | 0.000000000000 |
| ENSG00000204314 | 61.17595 | 23.99876 | 1.350005 | 0.000000000000 | 0.000000000000 |
| ENSG00000274049 | 10.68948 | 0.0001 | 16.70583 | 0.000000000000 | 0.000000000000 |
| ENSG00000255860 | 2.322212 | 24.16694 | -3.37946 | 0.000000000000 | 0.000000000000 |
| ENSG00000168564 | 3.957133 | 13.12895 | -1.73022 | 0.000000000000 | 0.000000000000 |
| ENSG00000105821 | 6.564835 | 22.38393 | -1.76963 | 0.000000000000 | 0.000000000000 |
| ENSG00000111181 | 29.37145 | 11.69203 | 1.32889 | 0.000000000000 | 0.000000000000 |
| ENSG00000072571 | 2.443554 | 9.577507 | -1.97067 | 0.000000000000 | 0.000000000000 |
| ENSG00000198554 | 3.579987 | 9.255486 | -1.37035 | 0.000000000000 | 0.000000000000 |
| ENSG00000184481 | 38.76868 | 15.66187 | 1.307635 | 0.000000000000 | 0.000000000000 |
| ENSG00000137955 | 7.112357 | 28.13963 | -1.9842 | 0.000000000000 | 0.000000000000 |
| ENSG00000170298 | 16.8997 | 40.41373 | -1.25785 | 0.000000000000 | 0.000000000000 |
| ENSG00000125945 | 0.987631 | 5.833557 | -2.56233 | 0.000000000000 | 0.000000000000 |
| ENSG00000130024 | 12.11784 | 33.02399 | -1.44638 | 0.000000000000 | 0.000000000000 |
| ENSG00000128965 | 2.069566 | 12.50558 | -2.59517 | 0.000000000000 | 0.000000000000 |
| ENSG00000249992 | 1.7741 | 11.49298 | -2.69559 | 0.000000000000 | 0.000000000000 |
| ENSG00000005102 | 23.2206 | 5.861109 | 1.98616 | 0.000000000000 | 0.000000000000 |
| ENSG00000153879 | 8.588999 | 18.22092 | -1.08503 | 0.000000000000 | 0.000000000000 |
| ENSG00000173193 | 4.197525 | 8.943623 | -1.09132 | 0.000000000000 | 0.000000000000 |
| ENSG00000119125 | 17.66823 | 65.28413 | -1.88558 | 0.000000000000 | 0.000000000000 |
| ENSG00000137492 | 1.906721 | 5.877508 | -1.62411 | 0.000000000000 | 0.000000000000 |
| ENSG00000220842 | 6.02263 | 41.38263 | -2.78056 | 0.000000000000 | 0.000000000000 |
| ENSG00000166261 | 4.196916 | 12.27365 | -1.54816 | 0.000000000000 | 0.000000000000 |
| ENSG00000107262 | 232.8743 | 115.0594 | 1.017172 | 0.000000000000 | 0.000000000000 |
| ENSG00000171017 | 10.1346 | 4.058006 | 1.320446 | 0.000000000000 | 0.000000000000 |
| ENSG00000125691 | 423.0746 | 977.2738 | -1.20785 | 0.000000000000 | 0.000000000000 |
| ENSG00000127526 | 86.75655 | 40.58691 | 1.095958 | 0.000000000000 | 0.000000000000 |
| ENSG00000022567 | 23.02913 | 10.72799 | 1.10208 | 0.000000000000 | 0.000000000000 |
| ENSG00000149418 | 11.60943 | 3.780111 | 1.618797 | 0.000000000000 | 0.000000000000 |
| ENSG00000085117 | 28.28526 | 9.647872 | 1.551768 | 0.000000000000 | 0.000000000000 |
| ENSG00000092758 | 19.961 | 6.821835 | 1.548952 | 0.000000000000 | 0.000000000000 |
| ENSG00000091317 | 2.223709 | 8.276223 | -1.896 | 0.000000000000 | 0.000000000000 |
| ENSG00000170633 | 12.35479 | 30.52447 | -1.3049 | 0.000000000000 | 0.000000000000 |
| ENSG00000158623 | 6.920475 | 17.11802 | -1.30657 | 0.000000000000 | 0.000000000000 |
| ENSG00000108784 | 36.03021 | 17.6943 | 1.025922 | 0.000000000000 | 0.000000000000 |
| ENSG00000163510 | 0.506806 | 5.096946 | -3.33013 | 0.000000000000 | 0.000000000000 |
| ENSG00000266094 | 36.62881 | 16.36662 | 1.162222 | 0.000000000000 | 0.000000000000 |
| ENSG00000165644 | 87.48534 | 42.5698 | 1.039211 | 0.000000000000 | 0.000000000000 |
| ENSG00000123473 | 7.092582 | 15.33279 | -1.11224 | 0.000000000000 | 0.000000000000 |
| ENSG00000234771 | 8.272979 | 3.464859 | 1.25561 | 0.000000000000 | 0.000000000000 |
| ENSG00000262526 | 41.20837 | 88.34247 | -1.10017 | 0.000000000000 | 0.000000000000 |
| ENSG00000078674 | 5.966969 | 13.86916 | -1.21681 | 0.000000000000 | 0.000000000000 |
| ENSG00000093100 | 4.769367 | 11.85023 | -1.31305 | 0.000000000000 | 0.000000000000 |
| ENSG00000065809 | 21.24319 | 44.0514 | -1.05219 | 0.000000000000 | 0.000000000000 |
| ENSG00000214900 | 2.933975 | 12.96652 | -2.14386 | 0.000000000000 | 0.000000000000 |
| ENSG00000023041 | 18.45278 | 41.69236 | -1.17595 | 0.000000000000 | 0.000000000000 |
| ENSG00000180964 | 31.56234 | 11.15106 | 1.501023 | 0.000000000000 | 0.000000000000 |
| ENSG00000177380 | 30.35926 | 14.74445 | 1.041964 | 0.000000000000 | 0.000000000000 |
| ENSG00000100567 | 26.54625 | 62.58267 | -1.23726 | 0.000000000000 | 0.000000000000 |
| ENSG00000269955 | 9.433688 | 0.0001 | 16.52553 | 0.000000000000 | 0.000000000000 |
| ENSG00000110768 | 13.37476 | 31.87037 | -1.2527 | 0.000000000000 | 0.000000000000 |
| ENSG00000269378 | 2.519516 | 25.46743 | -3.33743 | 0.000000000000 | 0.000000000000 |
| ENSG00000104081 | 5.894927 | 0.641228 | 3.200565 | 0.000000000000 | 0.000000000000 |
| ENSG00000218336 | 22.85672 | 9.603154 | 1.251038 | 0.000000000000 | 0.000000000000 |
| ENSG00000277739 | 160.0355 | 36.36565 | 2.137744 | 0.000000000000 | 0.000000000000 |
| ENSG00000270882 | 24.38104 | 54.74941 | -1.16708 | 0.000000000000 | 0.000000000000 |
| ENSG00000162645 | 7.99136 | 1.955655 | 2.030789 | 0.000000000000 | 0.000000000000 |
| ENSG00000087206 | 18.23843 | 39.70494 | -1.12234 | 0.000000000000 | 0.000000000000 |
| ENSG00000066923 | 13.56327 | 5.285855 | 1.359497 | 0.000000000000 | 0.000000000000 |
| ENSG00000260342 | 66.1982 | 27.77493 | 1.253009 | 0.000000000000 | 0.000000000000 |
| ENSG00000176092 | 5.926249 | 12.10757 | -1.03072 | 0.000000000000 | 0.000000000000 |
| ENSG00000170860 | 7.759703 | 16.71089 | -1.10671 | 0.000000000000 | 0.000000000000 |
| ENSG00000169184 | 19.42922 | 9.686614 | 1.004163 | 0.000000000000 | 0.000000000000 |
| ENSG00000171928 | 12.12613 | 25.80375 | -1.08946 | 0.000000000000 | 0.000000000000 |
| ENSG00000145287 | 30.48994 | 67.18823 | -1.13988 | 0.000000000000 | 0.000000000000 |
| ENSG00000029363 | 5.217458 | 18.4821 | -1.82471 | 0.000000000000 | 0.000000000000 |
| ENSG00000147168 | 4.466211 | 20.32346 | -2.18602 | 0.000000000000 | 0.000000000000 |
| ENSG00000273173 | 5.126308 | 29.15132 | -2.50757 | 0.000000000000 | 0.000000000000 |
| ENSG00000196220 | 16.49641 | 5.938285 | 1.474034 | 0.000000000000 | 0.000000000000 |
| ENSG00000147400 | 19.60984 | 42.41179 | -1.11289 | 0.000000000000 | 0.000000000000 |
| ENSG00000121211 | 2.502975 | 18.51496 | -2.88698 | 0.000000000000 | 0.000000000000 |
| ENSG00000128654 | 10.12731 | 31.1739 | -1.62209 | 0.000000000000 | 0.000000000000 |
| ENSG00000163545 | 6.647307 | 15.50222 | -1.22163 | 0.000000000000 | 0.000000000000 |
| ENSG00000152778 | 1.303129 | 5.821064 | -2.1593 | 0.000000000000 | 0.000000000000 |
| ENSG00000198898 | 3.086262 | 8.312141 | -1.42936 | 0.000000000000 | 0.000000000000 |
| ENSG00000163528 | 13.86214 | 33.96244 | -1.29279 | 0.000000000000 | 0.000000000000 |
| ENSG00000186567 | 20.95635 | 7.201012 | 1.541116 | 0.000000000000 | 0.000000000000 |
| ENSG00000121749 | 5.109151 | 25.69896 | -2.33055 | 0.000000000000 | 0.000000000000 |
| ENSG00000147854 | 14.98852 | 34.55174 | -1.2049 | 0.000000000000 | 0.000000000000 |
| ENSG00000063180 | 22.50702 | 8.78807 | 1.356756 | 0.000000000000 | 0.000000000000 |
| ENSG00000029993 | 9.768414 | 22.60415 | -1.21039 | 0.000000000000 | 0.000000000000 |
| ENSG00000253270 | 1.650878 | 27.07085 | -4.03543 | 0.000000000000 | 0.000000000000 |
| ENSG00000262877 | 36.21137 | 16.29085 | 1.152381 | 0.000000000000 | 0.000000000000 |
| ENSG00000120690 | 10.26561 | 23.02841 | -1.16559 | 0.000000000000 | 0.000000000000 |
| ENSG00000136536 | 5.139144 | 12.42306 | -1.27342 | 0.000000000000 | 0.000000000000 |
| ENSG00000135801 | 10.77353 | 21.92451 | -1.02505 | 0.000000000000 | 0.000000000000 |
| ENSG00000168490 | 21.24267 | 4.421131 | 2.264478 | 0.000000000000 | 0.000000000000 |
| ENSG00000080608 | 13.43779 | 28.27301 | -1.07313 | 0.000000000000 | 0.000000000000 |
| ENSG00000179111 | 64.74409 | 20.65402 | 1.648326 | 0.000000000000 | 0.000000000000 |
| ENSG00000100603 | 24.5608 | 49.15335 | -1.00093 | 0.000000000000 | 0.000000000000 |
| ENSG00000273841 | 47.83115 | 96.47559 | -1.01221 | 0.000000000000 | 0.000000000000 |
| ENSG00000150776 | 4.40143 | 11.83793 | -1.42737 | 0.000000000000 | 0.000000000000 |
| ENSG00000242612 | 54.21325 | 25.11939 | 1.109844 | 0.000000000000 | 0.000000000000 |
| ENSG00000243725 | 15.57739 | 32.42956 | -1.05786 | 0.000000000000 | 0.000000000000 |
| ENSG00000167774 | 20.19368 | 60.14008 | -1.57442 | 0.000000000000 | 0.000000000000 |
| ENSG00000092969 | 2.985986 | 8.052628 | -1.43125 | 0.000000000000 | 0.000000000000 |
| ENSG00000262633 | 7.164916 | 0.230904 | 4.955585 | 0.000000000000 | 0.000000000000 |
| ENSG00000129680 | 3.720614 | 9.35158 | -1.32967 | 0.000000000000 | 0.000000000000 |
| ENSG00000115816 | 7.800345 | 18.63036 | -1.25605 | 0.000000000000 | 0.000000000000 |
| ENSG00000144674 | 25.67945 | 10.32513 | 1.314455 | 0.000000000000 | 0.000000000000 |
| ENSG00000171208 | 6.364241 | 25.98146 | -2.02942 | 0.000000000000 | 0.000000000000 |
| ENSG00000115009 | 1.229318 | 15.88397 | -3.69164 | 0.000000000000 | 0.000000000000 |
| ENSG00000174442 | 2.27482 | 10.43958 | -2.19824 | 0.000000000000 | 0.000000000000 |
| ENSG00000182796 | 47.62857 | 23.61028 | 1.012413 | 0.000000000000 | 0.000000000000 |
| ENSG00000171604 | 69.99442 | 29.47308 | 1.247842 | 0.000000000000 | 0.000000000000 |
| ENSG00000119203 | 12.23876 | 25.9342 | -1.0834 | 0.000000000000 | 0.000000000000 |
| ENSG00000107099 | 17.70273 | 8.695357 | 1.025655 | 0.000000000000 | 0.000000000000 |
| ENSG00000272949 | 7.617586 | 2.205757 | 1.78806 | 0.000000000000 | 0.000000000000 |
| ENSG00000065613 | 5.974983 | 12.98379 | -1.1197 | 0.000000000000 | 0.000000000000 |
| ENSG00000183735 | 2.171354 | 8.03649 | -1.88797 | 0.000000000000 | 0.000000000000 |
| ENSG00000168016 | 9.390743 | 3.877795 | 1.276003 | 0.000000000000 | 0.000000000000 |
| ENSG00000099219 | 17.19972 | 36.87339 | -1.10019 | 0.000000000000 | 0.000000000000 |
| ENSG00000173915 | 81.20072 | 165.0761 | -1.02357 | 0.000000000000 | 0.000000000000 |
| ENSG00000123505 | 6.40339 | 14.833 | -1.2119 | 0.000000000000 | 0.000000000000 |
| ENSG00000220793 | 0.280033 | 21.3555 | -6.25287 | 0.000000000000 | 0.000000000000 |
| ENSG00000214194 | 9.597019 | 29.43483 | -1.61687 | 0.000000000000 | 0.000000000000 |
| ENSG00000119900 | 3.06483 | 7.284561 | -1.24903 | 0.000000000000 | 0.000000000000 |
| ENSG00000171236 | 19.7607 | 9.854831 | 1.003731 | 0.000000000000 | 0.000000000000 |
| ENSG00000258818 | 16.72133 | 7.031056 | 1.249876 | 0.000000000000 | 0.000000000000 |
| ENSG00000118197 | 9.003452 | 21.6702 | -1.26716 | 0.000000000000 | 0.000000000000 |
| ENSG00000249353 | 7.323149 | 28.39016 | -1.95486 | 0.000000000000 | 0.000000000000 |
| ENSG00000134049 | 88.81535 | 21.21869 | 2.065474 | 0.000000000000 | 0.000000000000 |
| ENSG00000174695 | 2.826932 | 8.450583 | -1.57981 | 0.000000000000 | 0.000000000000 |
| ENSG00000268592 | 27.59653 | 9.853696 | 1.48575 | 0.000000000000 | 0.000000000000 |
| ENSG00000133835 | 41.26652 | 99.8374 | -1.27461 | 0.000000000000 | 0.000000000000 |
| ENSG00000133247 | 32.12665 | 13.94872 | 1.203638 | 0.000000000000 | 0.000000000000 |
| ENSG00000123737 | 7.936546 | 29.07566 | -1.87323 | 0.000000000000 | 0.000000000000 |
| ENSG00000102384 | 6.393107 | 13.52485 | -1.08102 | 0.000000000000 | 0.000000000000 |
| ENSG00000104756 | 6.291984 | 16.9264 | -1.42769 | 0.000000000000 | 0.000000000000 |
| ENSG00000113318 | 1.883806 | 6.459572 | -1.77779 | 0.000000000000 | 0.000000000000 |
| ENSG00000140830 | 7.032604 | 16.96953 | -1.27082 | 0.000000000000 | 0.000000000000 |
| ENSG00000119616 | 2.729063 | 10.42818 | -1.93401 | 0.000000000000 | 0.000000000000 |
| ENSG00000108384 | 12.42521 | 30.06097 | -1.27462 | 0.000000000000 | 0.000000000000 |
| ENSG00000278677 | 27.20083 | 1.496862 | 4.183638 | 0.000000000000 | 0.000000000000 |
| ENSG00000138138 | 6.099463 | 13.52127 | -1.14848 | 0.000000000000 | 0.000000000000 |
| ENSG00000152404 | 1.188331 | 5.689628 | -2.2594 | 0.000000000000 | 0.000000000000 |
| ENSG00000179477 | 9.723913 | 0.474548 | 4.356911 | 0.000000000000 | 0.000000000000 |
| ENSG00000156110 | 20.7373 | 49.61348 | -1.2585 | 0.000000000000 | 0.000000000000 |
| ENSG00000227039 | 1.668236 | 6.795229 | -2.0262 | 0.000000000000 | 0.000000000000 |
| ENSG00000139269 | 5.091661 | 13.89998 | -1.44887 | 0.000000000000 | 0.000000000000 |
| ENSG00000227345 | 7.434054 | 16.46559 | -1.14723 | 0.000000000000 | 0.000000000000 |
| ENSG00000197978 | 7.341646 | 3.196612 | 1.19956 | 0.000000000000 | 0.000000000000 |
| ENSG00000196505 | 1.023353 | 5.303171 | -2.37355 | 0.000000000000 | 0.000000000000 |
| ENSG00000263934 | 82.07217 | 15.27275 | 2.425933 | 0.000000000000 | 0.000000000000 |
| ENSG00000129055 | 13.813 | 31.25576 | -1.1781 | 0.000000000000 | 0.000000000000 |
| ENSG00000104412 | 1.7543 | 12.29683 | -2.80932 | 0.000000000000 | 0.000000000000 |
| ENSG00000248167 | 6.947999 | 0.0001 | 16.08431 | 0.000000000000 | 0.000000000000 |
| ENSG00000116750 | 14.08394 | 31.82717 | -1.17621 | 0.000000000000 | 0.000000000000 |
| ENSG00000187325 | 2.874491 | 9.813622 | -1.77148 | 0.000000000000 | 0.000000000000 |
| ENSG00000126858 | 6.910953 | 16.9936 | -1.29804 | 0.000000000000 | 0.000000000000 |
| ENSG00000115808 | 2.863749 | 6.60554 | -1.20577 | 0.000000000000 | 0.000000000000 |
| ENSG00000135722 | 57.81149 | 24.16073 | 1.258692 | 0.000000000000 | 0.000000000000 |
| ENSG00000036672 | 13.86949 | 6.881318 | 1.011158 | 0.000000000000 | 0.000000000000 |
| ENSG00000127423 | 6.255959 | 16.39684 | -1.39012 | 0.000000000000 | 0.000000000000 |
| ENSG00000162607 | 4.744767 | 11.11816 | -1.22851 | 0.000000000000 | 0.000000000000 |
| ENSG00000225830 | 12.40955 | 34.4971 | -1.47502 | 0.000000000000 | 0.000000000000 |
| ENSG00000154102 | 14.75151 | 36.48005 | -1.30625 | 0.000000000000 | 0.000000000000 |
| ENSG00000258727 | 8.310446 | 2.669941 | 1.638118 | 0.000000000000 | 0.000000000000 |
| ENSG00000122482 | 7.965333 | 17.57538 | -1.14175 | 0.000000000000 | 0.000000000000 |
| ENSG00000246273 | 3.501199 | 16.84321 | -2.26625 | 0.000000000000 | 0.000000000000 |
| ENSG00000147669 | 6.148167 | 19.60842 | -1.67325 | 0.000000000000 | 0.000000000000 |
| ENSG00000145220 | 9.895004 | 24.57644 | -1.3125 | 0.000000000000 | 0.000000000000 |
| ENSG00000064652 | 6.226844 | 20.87577 | -1.74526 | 0.000000000000 | 0.000000000000 |
| ENSG00000230699 | 6.510525 | 1.590714 | 2.033099 | 0.000000000000 | 0.000000000000 |
| ENSG00000111247 | 4.654161 | 17.36257 | -1.89939 | 0.000000000000 | 0.000000000000 |
| ENSG00000132688 | 7.872803 | 3.500197 | 1.169441 | 0.000000000000 | 0.000000000000 |
| ENSG00000112851 | 4.29154 | 9.991176 | -1.21916 | 0.000000000000 | 0.000000000000 |
| ENSG00000166900 | 9.800822 | 25.8045 | -1.39665 | 0.000000000000 | 0.000000000000 |
| ENSG00000018699 | 7.699986 | 15.94728 | -1.05038 | 0.000000000000 | 0.000000000000 |
| ENSG00000139350 | 3.113428 | 10.861 | -1.80258 | 0.000000000000 | 0.000000000000 |
| ENSG00000163029 | 3.254839 | 7.673879 | -1.23737 | 0.000000000000 | 0.000000000000 |
| ENSG00000210176 | 1.074053 | 136.4086 | -6.98873 | 0.000000000000 | 0.000000000000 |
| ENSG00000198836 | 3.864022 | 11.15942 | -1.53009 | 0.000000000000 | 0.000000000000 |
| ENSG00000118804 | 3.733283 | 9.910404 | -1.4085 | 0.000000000000 | 0.000000000000 |
| ENSG00000234498 | 4.687637 | 25.91884 | -2.46707 | 0.000000000000 | 0.000000000000 |
| ENSG00000256206 | 0.261518 | 7.654489 | -4.87132 | 0.000000000000 | 0.000000000000 |
| ENSG00000176731 | 3.646912 | 22.95162 | -2.65385 | 0.000000000000 | 0.000000000000 |
| ENSG00000084710 | 6.112044 | 2.166104 | 1.496552 | 0.000000000000 | 0.000000000000 |
| ENSG00000144596 | 8.669034 | 2.754684 | 1.653984 | 0.000000000000 | 0.000000000000 |
| ENSG00000135541 | 2.022439 | 8.679283 | -2.10148 | 0.000000000000 | 0.000000000000 |
| ENSG00000220785 | 16.35963 | 6.75672 | 1.275745 | 0.000000000000 | 0.000000000000 |
| ENSG00000183607 | 0.289213 | 10.92316 | -5.23911 | 0.000000000000 | 0.000000000000 |
| ENSG00000204758 | 4.10727 | 15.16679 | -1.88466 | 0.000000000000 | 0.000000000000 |
| ENSG00000168807 | 18.74848 | 6.823296 | 1.458233 | 0.000000000000 | 0.000000000000 |
| ENSG00000114529 | 7.821509 | 19.16108 | -1.29266 | 0.000000000000 | 0.000000000000 |
| ENSG00000166024 | 5.391914 | 12.5039 | -1.21351 | 0.000000000000 | 0.000000000000 |
| ENSG00000185561 | 5.53398 | 2.132923 | 1.375486 | 0.000000000000 | 0.000000000000 |
| ENSG00000275888 | 27.57326 | 5.991292 | 2.202331 | 0.000000000000 | 0.000000000000 |
| ENSG00000104231 | 14.68511 | 36.90327 | -1.32939 | 0.000000000000 | 0.000000000000 |
| ENSG00000152443 | 1.997091 | 7.202701 | -1.85064 | 0.000000000000 | 0.000000000000 |
| ENSG00000152990 | 5.54882 | 11.19167 | -1.01217 | 0.000000000000 | 0.000000000000 |
| ENSG00000167191 | 2.144515 | 5.625278 | -1.39127 | 0.000000000000 | 0.000000000000 |
| ENSG00000203668 | 3.901819 | 13.13444 | -1.75114 | 0.000000000000 | 0.000000000000 |
| ENSG00000234072 | 27.07441 | 10.18053 | 1.411117 | 0.000000000000 | 0.000000000000 |
| ENSG00000068784 | 1.798228 | 6.658308 | -1.88858 | 0.000000000000 | 0.000000000000 |
| ENSG00000151849 | 4.305387 | 12.2576 | -1.50946 | 0.000000000000 | 0.000000000000 |
| ENSG00000186063 | 2.820974 | 10.12866 | -1.84418 | 0.000000000000 | 0.000000000000 |
| ENSG00000102710 | 14.9937 | 30.78344 | -1.0378 | 0.000000000000 | 0.000000000000 |
| ENSG00000197579 | 2.315067 | 6.765999 | -1.54725 | 0.000000000000 | 0.000000000000 |
| ENSG00000135241 | 3.728187 | 10.30027 | -1.46614 | 0.000000000000 | 0.000000000000 |
| ENSG00000101901 | 14.34109 | 30.7303 | -1.09951 | 0.000000000000 | 0.000000000000 |
| ENSG00000102048 | 20.64953 | 9.436257 | 1.129822 | 0.000000000000 | 0.000000000000 |
| ENSG00000133119 | 10.1644 | 23.58743 | -1.21449 | 0.000000000000 | 0.000000000000 |
| ENSG00000197417 | 10.80849 | 4.86445 | 1.151816 | 0.000000000000 | 0.000000000000 |
| ENSG00000279117 | 8.241366 | 2.141529 | 1.944242 | 0.000000000000 | 0.000000000000 |
| ENSG00000104671 | 14.00055 | 41.09513 | -1.55348 | 0.000000000000 | 0.000000000000 |
| ENSG00000257732 | 1.732361 | 16.02687 | -3.20968 | 0.000000000000 | 0.000000000000 |
| ENSG00000164941 | 5.704374 | 14.64811 | -1.36057 | 0.000000000000 | 0.000000000000 |
| ENSG00000139684 | 18.97278 | 42.23041 | -1.15435 | 0.000000000000 | 0.000000000000 |
| ENSG00000155096 | 12.89568 | 31.16908 | -1.27323 | 0.000000000000 | 0.000000000000 |
| ENSG00000165169 | 27.06703 | 9.511756 | 1.508753 | 0.000000000000 | 0.000000000000 |
| ENSG00000127947 | 8.489148 | 19.32694 | -1.18692 | 0.000000000000 | 0.000000000000 |
| ENSG00000151422 | 2.072444 | 6.234844 | -1.58902 | 0.000000000000 | 0.000000000000 |
| ENSG00000128513 | 0.822188 | 8.388089 | -3.3508 | 0.000000000000 | 0.000000000000 |
| ENSG00000164172 | 7.252707 | 19.94965 | -1.45977 | 0.000000000000 | 0.000000000000 |
| ENSG00000207513 | 75.94651 | 10.76639 | 2.818449 | 0.000000000000 | 0.000000000000 |
| ENSG00000189403 | 43.41573 | 98.44044 | -1.18103 | 0.000000000000 | 0.000000000000 |
| ENSG00000006611 | 20.10034 | 5.946612 | 1.75708 | 0.000000000000 | 0.000000000000 |
| ENSG00000254692 | 9.530747 | 3.286574 | 1.536005 | 0.000000000000 | 0.000000000000 |
| ENSG00000168952 | 1.985762 | 8.114644 | -2.03084 | 0.000000000000 | 0.000000000000 |
| ENSG00000137962 | 2.420955 | 5.927928 | -1.29195 | 0.000000000000 | 0.000000000000 |
| ENSG00000092208 | 6.922591 | 24.07293 | -1.79803 | 0.000000000000 | 0.000000000000 |
| ENSG00000067191 | 10.12623 | 3.483206 | 1.539609 | 0.000000000000 | 0.000000000000 |
| ENSG00000215790 | 30.50542 | 14.47382 | 1.07562 | 0.000000000000 | 0.000000000000 |
| ENSG00000168944 | 2.346114 | 5.719285 | -1.28556 | 0.000000000000 | 0.000000000000 |
| ENSG00000259605 | 24.45963 | 10.21597 | 1.259577 | 0.000000000000 | 0.000000000000 |
| ENSG00000078142 | 5.561271 | 11.97712 | -1.10679 | 0.000000000000 | 0.000000000000 |
| ENSG00000234880 | 18.54415 | 8.357085 | 1.149893 | 0.000000000000 | 0.000000000000 |
| ENSG00000117475 | 3.148961 | 12.87154 | -2.03124 | 0.000000000000 | 0.000000000000 |
| ENSG00000274020 | 8.562779 | 21.92235 | -1.35625 | 0.000000000000 | 0.000000000000 |
| ENSG00000141219 | 5.683009 | 12.68358 | -1.15823 | 0.000000000000 | 0.000000000000 |
| ENSG00000205268 | 3.518042 | 8.669608 | -1.30119 | 0.000000000000 | 0.000000000000 |
| ENSG00000210184 | 0.97748 | 142.6896 | -7.1896 | 0.000000000000 | 0.000000000000 |
| ENSG00000134058 | 12.52042 | 28.90145 | -1.20686 | 0.000000000000 | 0.000000000000 |
| ENSG00000144810 | 9.024229 | 18.99717 | -1.07391 | 0.000000000000 | 0.000000000000 |
| ENSG00000170153 | 6.1459 | 2.603935 | 1.238931 | 0.000000000000 | 0.000000000000 |
| ENSG00000197603 | 1.59891 | 7.200524 | -2.17101 | 0.000000000000 | 0.000000000000 |
| ENSG00000124102 | 7.399406 | 30.39834 | -2.03851 | 0.000000000000 | 0.000000000000 |
| ENSG00000103199 | 14.43686 | 5.615687 | 1.362222 | 0.000000000000 | 0.000000000000 |
| ENSG00000196415 | 30.41734 | 12.19576 | 1.318514 | 0.000000000000 | 0.000000000000 |
| ENSG00000258890 | 9.374164 | 19.53503 | -1.0593 | 0.000000000000 | 0.000000000000 |
| ENSG00000181029 | 19.84664 | 9.369813 | 1.082803 | 0.000000000000 | 0.000000000000 |
| ENSG00000127314 | 13.13539 | 26.98292 | -1.03859 | 0.000000000000 | 0.000000000000 |
| ENSG00000213430 | 0.872448 | 6.627072 | -2.92523 | 0.000000000000 | 0.000000000000 |
| ENSG00000169139 | 7.942635 | 26.87808 | -1.75874 | 0.000000000000 | 0.000000000000 |
| ENSG00000198912 | 20.86852 | 42.82444 | -1.03711 | 0.000000000000 | 0.000000000000 |
| ENSG00000178927 | 98.61215 | 41.73293 | 1.240579 | 0.000000000000 | 0.000000000000 |
| ENSG00000204396 | 17.27702 | 7.004081 | 1.302587 | 0.000000000000 | 0.000000000000 |
| ENSG00000214485 | 16.42058 | 41.80284 | -1.3481 | 0.000000000000 | 0.000000000000 |
| ENSG00000140961 | 8.353488 | 25.03589 | -1.58355 | 0.000000000000 | 0.000000000000 |
| ENSG00000089820 | 67.3608 | 28.49586 | 1.241157 | 0.000000000000 | 0.000000000000 |
| ENSG00000205403 | 7.836173 | 1.149027 | 2.769737 | 0.000000000000 | 0.000000000000 |
| ENSG00000141682 | 10.34951 | 21.85617 | -1.07848 | 0.000000000000 | 0.000000000000 |
| ENSG00000165685 | 1.350984 | 10.35095 | -2.93768 | 0.000000000000 | 0.000000000000 |
| ENSG00000207005 | 72.51889 | 10.73495 | 2.756041 | 0.000000000000 | 0.000000000000 |
| ENSG00000247199 | 18.6046 | 8.348612 | 1.156051 | 0.000000000000 | 0.000000000000 |
| ENSG00000175054 | 3.487764 | 7.650382 | -1.13323 | 0.000000000000 | 0.000000000000 |
| ENSG00000196776 | 3.176247 | 11.00539 | -1.79282 | 0.000000000000 | 0.000000000000 |
| ENSG00000120948 | 35.26748 | 74.10542 | -1.07124 | 0.000000000000 | 0.000000000000 |
| ENSG00000141933 | 34.66798 | 16.36369 | 1.083106 | 0.000000000000 | 0.000000000000 |
| ENSG00000082258 | 7.803811 | 2.833947 | 1.461366 | 0.000000000000 | 0.000000000000 |
| ENSG00000213326 | 3.710167 | 21.94297 | -2.5642 | 0.000000000000 | 0.000000000000 |
| ENSG00000163808 | 3.644355 | 8.319949 | -1.19091 | 0.000000000000 | 0.000000000000 |
| ENSG00000186198 | 8.111494 | 0.208288 | 5.283316 | 0.000000000000 | 0.000000000000 |
| ENSG00000153094 | 7.13221 | 3.103173 | 1.200605 | 0.000000000000 | 0.000000000000 |
| ENSG00000155313 | 4.977868 | 12.86255 | -1.36958 | 0.000000000000 | 0.000000000000 |
| ENSG00000100479 | 6.266528 | 16.15922 | -1.36662 | 0.000000000000 | 0.000000000000 |
| ENSG00000144476 | 30.04853 | 97.23203 | -1.69414 | 0.000000000000 | 0.000000000000 |
| ENSG00000068796 | 7.074852 | 18.64116 | -1.39772 | 0.000000000000 | 0.000000000000 |
| ENSG00000154582 | 33.57758 | 76.98831 | -1.19714 | 0.000000000000 | 0.000000000000 |
| ENSG00000225067 | 1.961979 | 20.56075 | -3.38951 | 0.000000000000 | 0.000000000000 |
| ENSG00000125744 | 41.40539 | 15.53971 | 1.413859 | 0.000000000000 | 0.000000000000 |
| ENSG00000062725 | 3.055912 | 6.817906 | -1.15773 | 0.000000000000 | 0.000000000000 |
| ENSG00000172172 | 17.25274 | 37.33677 | -1.11377 | 0.000000000000 | 0.000000000000 |
| ENSG00000105372 | 541.9981 | 1404.996 | -1.37421 | 0.000000000000 | 0.000000000000 |
| ENSG00000240541 | 6.501511 | 24.41752 | -1.90907 | 0.000000000000 | 0.000000000000 |
| ENSG00000175354 | 12.3278 | 25.57584 | -1.05287 | 0.000000000000 | 0.000000000000 |
| ENSG00000196937 | 5.800407 | 13.9576 | -1.26682 | 0.000000000000 | 0.000000000000 |
| ENSG00000064270 | 3.240217 | 10.42383 | -1.68572 | 0.000000000000 | 0.000000000000 |
| ENSG00000266036 | 41.16619 | 14.04112 | 1.551802 | 0.000000000000 | 0.000000000000 |
| ENSG00000115596 | 6.108326 | 0.759918 | 3.006861 | 0.000000000000 | 0.000000000000 |
| ENSG00000169826 | 3.112189 | 7.916318 | -1.3469 | 0.000000000000 | 0.000000000000 |
| ENSG00000116641 | 8.930954 | 20.58931 | -1.20501 | 0.000000000000 | 0.000000000000 |
| ENSG00000119844 | 5.939943 | 16.08326 | -1.43704 | 0.000000000000 | 0.000000000000 |
| ENSG00000196754 | 11.08987 | 32.03248 | -1.53029 | 0.000000000000 | 0.000000000000 |
| ENSG00000188277 | 8.737947 | 2.770752 | 1.657017 | 0.000000000000 | 0.000000000000 |
| ENSG00000146826 | 33.4408 | 16.45459 | 1.023119 | 0.000000000000 | 0.000000000000 |
| ENSG00000171084 | 2.169781 | 7.743074 | -1.83536 | 0.000000000000 | 0.000000000000 |
| ENSG00000188428 | 4.720411 | 10.83872 | -1.19921 | 0.000000000000 | 0.000000000000 |
| ENSG00000077713 | 6.253309 | 12.69908 | -1.02203 | 0.000000000000 | 0.000000000000 |
| ENSG00000178685 | 49.32882 | 19.51571 | 1.337795 | 0.000000000000 | 0.000000000000 |
| ENSG00000086827 | 9.552886 | 20.12369 | -1.07489 | 0.000000000000 | 0.000000000000 |
| ENSG00000188092 | 7.079235 | 14.58195 | -1.04252 | 0.000000000000 | 0.000000000000 |
| ENSG00000152359 | 2.421971 | 9.648936 | -1.99419 | 0.000000000000 | 0.000000000000 |
| ENSG00000067066 | 19.95661 | 42.18361 | -1.07982 | 0.000000000000 | 0.000000000000 |
| ENSG00000234636 | 21.29023 | 6.439468 | 1.725178 | 0.000000000000 | 0.000000000000 |
| ENSG00000266642 | 10.819 | 1.844647 | 2.552151 | 0.000000000000 | 0.000000000000 |
| ENSG00000138709 | 2.039894 | 9.433967 | -2.20937 | 0.000000000000 | 0.000000000000 |
| ENSG00000159882 | 3.641648 | 7.573098 | -1.05629 | 0.000000000000 | 0.000000000000 |
| ENSG00000197601 | 1.842892 | 7.492251 | -2.02343 | 0.000000000000 | 0.000000000000 |
| ENSG00000128463 | 68.75674 | 32.45008 | 1.083279 | 0.000000000000 | 0.000000000000 |
| ENSG00000215196 | 9.084361 | 3.540517 | 1.359425 | 0.000000000000 | 0.000000000000 |
| ENSG00000149591 | 14.42315 | 33.9072 | -1.23321 | 0.000000000000 | 0.000000000000 |
| ENSG00000176182 | 21.00002 | 10.12483 | 1.052493 | 0.000000000000 | 0.000000000000 |
| ENSG00000174720 | 2.785964 | 13.70117 | -2.29805 | 0.000000000000 | 0.000000000000 |
| ENSG00000277363 | 16.91063 | 7.710387 | 1.133055 | 0.000000000000 | 0.000000000000 |
| ENSG00000123838 | 5.825329 | 0.717635 | 3.021017 | 0.000000000000 | 0.000000000000 |
| ENSG00000278334 | 0.011743 | 44.19896 | -11.878 | 0.000000000000 | 0.000000000000 |
| ENSG00000214944 | 3.399332 | 7.129524 | -1.06855 | 0.000000000000 | 0.000000000000 |
| ENSG00000006025 | 17.43276 | 8.305284 | 1.0697 | 0.000000000000 | 0.000000000000 |
| ENSG00000244021 | 0.088211 | 14.51081 | -7.36195 | 0.000000000000 | 0.000000000000 |
| ENSG00000146282 | 8.17753 | 19.48056 | -1.2523 | 0.000000000000 | 0.000000000000 |
| ENSG00000167971 | 5.783915 | 2.378988 | 1.281698 | 0.000000000000 | 0.000000000000 |
| ENSG00000169752 | 23.21079 | 47.41032 | -1.03041 | 0.000000000000 | 0.000000000000 |
| ENSG00000160408 | 46.72954 | 15.99711 | 1.546523 | 0.000000000000 | 0.000000000000 |
| ENSG00000065717 | 24.98598 | 8.511068 | 1.553707 | 0.000000000000 | 0.000000000000 |
| ENSG00000148671 | 46.23179 | 14.90133 | 1.633444 | 0.000000000000 | 0.000000000000 |
| ENSG00000234678 | 28.51006 | 10.2113 | 1.481304 | 0.000000000000 | 0.000000000000 |
| ENSG00000273513 | 5.576088 | 1.017464 | 2.454276 | 0.000000000000 | 0.000000000000 |
| ENSG00000166676 | 4.548413 | 9.612487 | -1.07955 | 0.000000000000 | 0.000000000000 |
| ENSG00000100526 | 8.406236 | 25.15608 | -1.58137 | 0.000000000000 | 0.000000000000 |
| ENSG00000162600 | 2.806931 | 9.042738 | -1.68777 | 0.000000000000 | 0.000000000000 |
| ENSG00000249007 | 16.01242 | 4.281701 | 1.902936 | 0.000000000000 | 0.000000000000 |
| ENSG00000214999 | 5.767781 | 0.887686 | 2.699895 | 0.000000000000 | 0.000000000000 |
| ENSG00000268439 | 15.1004 | 3.684476 | 2.035055 | 0.000000000000 | 0.000000000000 |
| ENSG00000135521 | 3.454704 | 10.77329 | -1.64082 | 0.000000000000 | 0.000000000000 |
| ENSG00000206262 | 14.69168 | 6.727762 | 1.1268 | 0.000000000000 | 0.000000000000 |
| ENSG00000085231 | 4.349079 | 14.42051 | -1.72934 | 0.000000000000 | 0.000000000000 |
| ENSG00000145780 | 3.281151 | 6.793633 | -1.04998 | 0.000000000000 | 0.000000000000 |
| ENSG00000155636 | 0.656915 | 5.68706 | -3.1139 | 0.000000000000 | 0.000000000000 |
| ENSG00000077254 | 6.79558 | 15.39137 | -1.17945 | 0.000000000000 | 0.000000000000 |
| ENSG00000275011 | 6.337707 | 0.581681 | 3.445661 | 0.000000000000 | 0.000000000000 |
| ENSG00000048544 | 8.806656 | 18.33332 | -1.0578 | 0.000000000000 | 0.000000000000 |
| ENSG00000137269 | 4.310088 | 15.97227 | -1.88978 | 0.000000000000 | 0.000000000000 |
| ENSG00000284116 | 6.775287 | 0.377213 | 4.166831 | 0.000000000000 | 0.000000000000 |
| ENSG00000214784 | 0.116911 | 7.938394 | -6.08536 | 0.000000000000 | 0.000000000000 |
| ENSG00000127914 | 4.576767 | 14.75245 | -1.68855 | 0.000000000000 | 0.000000000000 |
| ENSG00000171150 | 2.229472 | 6.00315 | -1.42902 | 0.000000000000 | 0.000000000000 |
| ENSG00000112029 | 4.027052 | 11.12456 | -1.46595 | 0.000000000000 | 0.000000000000 |
| ENSG00000102098 | 4.01387 | 9.407386 | -1.2288 | 0.000000000000 | 0.000000000000 |
| ENSG00000163945 | 9.565193 | 3.502204 | 1.449531 | 0.000000000000 | 0.000000000000 |
| ENSG00000117461 | 7.63078 | 3.557913 | 1.100799 | 0.000000000000 | 0.000000000000 |
| ENSG00000168301 | 3.052825 | 10.69523 | -1.80875 | 0.000000000000 | 0.000000000000 |
| ENSG00000210191 | 0.416748 | 96.94064 | -7.86178 | 0.000000000000 | 0.000000000000 |
| ENSG00000125863 | 56.72066 | 121.8353 | -1.10299 | 0.000000000000 | 0.000000000000 |
| ENSG00000085365 | 1.915348 | 5.202842 | -1.44169 | 0.000000000000 | 0.000000000000 |
| ENSG00000159184 | 8.048973 | 3.419369 | 1.235075 | 0.000000000000 | 0.000000000000 |
| ENSG00000198015 | 2.527237 | 10.97083 | -2.11804 | 0.000000000000 | 0.000000000000 |
| ENSG00000260285 | 7.520095 | 3.354355 | 1.164716 | 0.000000000000 | 0.000000000000 |
| ENSG00000102081 | 7.504014 | 15.53104 | -1.04942 | 0.000000000000 | 0.000000000000 |
| ENSG00000176396 | 23.46219 | 11.64959 | 1.010058 | 0.000000000000 | 0.000000000000 |
| ENSG00000159082 | 13.93894 | 4.321572 | 1.689493 | 0.000000000000 | 0.000000000000 |
| ENSG00000245849 | 23.22373 | 10.84692 | 1.098314 | 0.000000000000 | 0.000000000000 |
| ENSG00000138867 | 82.85433 | 21.35998 | 1.955667 | 0.000000000000 | 0.000000000000 |
| ENSG00000205744 | 33.93079 | 15.74271 | 1.107912 | 0.000000000000 | 0.000000000000 |
| ENSG00000111261 | 1.350258 | 6.920836 | -2.35771 | 0.000000000000 | 0.000000000000 |
| ENSG00000109929 | 8.791228 | 17.59693 | -1.00119 | 0.000000000000 | 0.000000000000 |
| ENSG00000203706 | 14.44241 | 36.39032 | -1.33324 | 0.000000000000 | 0.000000000000 |
| ENSG00000175110 | 15.59991 | 33.20457 | -1.08984 | 0.000000000000 | 0.000000000000 |
| ENSG00000197056 | 1.809341 | 5.967937 | -1.72177 | 0.000000000000 | 0.000000000000 |
| ENSG00000269937 | 5.247059 | 1.768864 | 1.568686 | 0.000000000000 | 0.000000000000 |
| ENSG00000134352 | 4.839273 | 9.891405 | -1.03139 | 0.000000000000 | 0.000000000000 |
| ENSG00000116711 | 3.633361 | 9.008233 | -1.30994 | 0.000000000000 | 0.000000000000 |
| ENSG00000137843 | 16.31942 | 6.429943 | 1.343712 | 0.000000000000 | 0.000000000000 |
| ENSG00000197062 | 3.358856 | 8.203787 | -1.28832 | 0.000000000000 | 0.000000000000 |
| ENSG00000164252 | 32.84668 | 7.249534 | 2.179787 | 0.000000000000 | 0.000000000000 |
| ENSG00000164815 | 4.585285 | 12.63214 | -1.46202 | 0.000000000000 | 0.000000000000 |
| ENSG00000103540 | 2.401345 | 6.711486 | -1.48279 | 0.000000000000 | 0.000000000000 |
| ENSG00000240344 | 13.64906 | 35.75898 | -1.3895 | 0.000000000000 | 0.000000000000 |
| ENSG00000139117 | 4.265884 | 8.85471 | -1.0536 | 0.000000000000 | 0.000000000000 |
| ENSG00000103528 | 37.20659 | 14.98753 | 1.311796 | 0.000000000000 | 0.000000000000 |
| ENSG00000111319 | 24.04354 | 8.124099 | 1.56537 | 0.000000000000 | 0.000000000000 |
| ENSG00000172482 | 6.425187 | 0.820572 | 2.969037 | 0.000000000000 | 0.000000000000 |
| ENSG00000029153 | 2.33186 | 7.158309 | -1.61814 | 0.000000000000 | 0.000000000000 |
| ENSG00000123178 | 4.698897 | 11.29412 | -1.26518 | 0.000000000000 | 0.000000000000 |
| ENSG00000206585 | 51.40012 | 6.664264 | 2.947254 | 0.000000000000 | 0.000000000000 |
| ENSG00000197275 | 1.080965 | 6.221104 | -2.52485 | 0.000000000000 | 0.000000000000 |
| ENSG00000211455 | 2.160335 | 5.331511 | -1.30329 | 0.000000000000 | 0.000000000000 |
| ENSG00000143028 | 6.266566 | 2.382413 | 1.395252 | 0.000000000000 | 0.000000000000 |
| ENSG00000063169 | 21.76421 | 9.589482 | 1.182433 | 0.000000000000 | 0.000000000000 |
| ENSG00000105419 | 16.39497 | 7.984154 | 1.038041 | 0.000000000000 | 0.000000000000 |
| ENSG00000162006 | 20.38545 | 5.120699 | 1.993127 | 0.000000000000 | 0.000000000000 |
| ENSG00000197498 | 2.903563 | 8.200432 | -1.49788 | 0.000000000000 | 0.000000000000 |
| ENSG00000151461 | 3.56391 | 7.46619 | -1.06691 | 0.000000000000 | 0.000000000000 |
| ENSG00000115942 | 3.09628 | 7.595844 | -1.29467 | 0.000000000000 | 0.000000000000 |
| ENSG00000100445 | 37.74462 | 18.06342 | 1.0632 | 0.000000000000 | 0.000000000000 |
| ENSG00000261305 | 15.38586 | 3.87114 | 1.990774 | 0.000000000000 | 0.000000000000 |
| ENSG00000163235 | 3.67285 | 7.40124 | -1.01087 | 0.000000000000 | 0.000000000000 |
| ENSG00000085832 | 7.489893 | 15.34997 | -1.03522 | 0.000000000000 | 0.000000000000 |
| ENSG00000173145 | 3.36983 | 7.84109 | -1.21838 | 0.000000000000 | 0.000000000000 |
| ENSG00000183340 | 1.882747 | 6.208485 | -1.7214 | 0.000000000000 | 0.000000000000 |
| ENSG00000210194 | 4.56423 | 90.62433 | -4.31146 | 0.000000000000 | 0.000000000000 |
| ENSG00000182518 | 5.554256 | 15.16695 | -1.44926 | 0.000000000000 | 0.000000000000 |
| ENSG00000115556 | 6.717972 | 1.514499 | 2.149185 | 0.000000000000 | 0.000000000000 |
| ENSG00000004766 | 4.402487 | 10.46178 | -1.24874 | 0.000000000000 | 0.000000000000 |
| ENSG00000143228 | 1.498653 | 9.043166 | -2.59316 | 0.000000000000 | 0.000000000000 |
| ENSG00000100749 | 5.309856 | 13.6873 | -1.36609 | 0.000000000000 | 0.000000000000 |
| ENSG00000280433 | 2.535475 | 6.308181 | -1.31497 | 0.000000000000 | 0.000000000000 |
| ENSG00000111432 | 10.30146 | 5.081747 | 1.019452 | 0.000000000000 | 0.000000000000 |
| ENSG00000122008 | 0.908529 | 7.027861 | -2.95148 | 0.000000000000 | 0.000000000000 |
| ENSG00000067955 | 49.03649 | 13.57401 | 1.853009 | 0.000000000000 | 0.000000000000 |
| ENSG00000173432 | 2.554448 | 15.16456 | -2.56962 | 0.000000000000 | 0.000000000000 |
| ENSG00000235174 | 1.844049 | 38.58744 | -4.38718 | 0.000000000000 | 0.000000000000 |
| ENSG00000096654 | 2.139476 | 6.110245 | -1.51397 | 0.000000000000 | 0.000000000000 |
| ENSG00000232187 | 0.628215 | 11.1599 | -4.15092 | 0.000000000000 | 0.000000000000 |
| ENSG00000176261 | 17.50249 | 38.88133 | -1.15152 | 0.000000000000 | 0.000000000000 |
| ENSG00000228502 | 12.30886 | 25.03862 | -1.02446 | 0.000000000000 | 0.000000000000 |
| ENSG00000136897 | 3.174534 | 7.546003 | -1.24917 | 0.000000000000 | 0.000000000000 |
| ENSG00000171916 | 11.4253 | 26.77542 | -1.22868 | 0.000000000000 | 0.000000000000 |
| ENSG00000055609 | 22.45438 | 11.20279 | 1.003139 | 0.000000000000 | 0.000000000000 |
| ENSG00000088836 | 8.443525 | 3.379026 | 1.321238 | 0.000000000000 | 0.000000000000 |
| ENSG00000174953 | 2.480075 | 18.95186 | -2.93388 | 0.000000000000 | 0.000000000000 |
| ENSG00000273088 | 8.11589 | 0.0001 | 16.30846 | 0.000000000000 | 0.000000000000 |
| ENSG00000261832 | 1.442521 | 5.454239 | -1.91879 | 0.000000000000 | 0.000000000000 |
| ENSG00000206737 | 50.05214 | 7.031068 | 2.831616 | 0.000000000000 | 0.000000000000 |
| ENSG00000260007 | 6.171731 | 2.21505 | 1.478336 | 0.000000000000 | 0.000000000000 |
| ENSG00000106976 | 6.671158 | 2.969991 | 1.167479 | 0.000000000000 | 0.000000000000 |
| ENSG00000156469 | 3.263799 | 9.304878 | -1.51144 | 0.000000000000 | 0.000000000000 |
| ENSG00000142609 | 8.232289 | 2.56732 | 1.681031 | 0.000000000000 | 0.000000000000 |
| ENSG00000119787 | 5.668481 | 14.48505 | -1.35353 | 0.000000000000 | 0.000000000000 |
| ENSG00000283149 | 0.0001 | 14.47851 | -17.1436 | 0.000000000000 | 0.000000000000 |
| ENSG00000181381 | 4.877506 | 10.69208 | -1.13233 | 0.000000000000 | 0.000000000000 |
| ENSG00000188215 | 3.124768 | 6.533326 | -1.06407 | 0.000000000000 | 0.000000000000 |
| ENSG00000162913 | 8.906378 | 4.090527 | 1.122552 | 0.000000000000 | 0.000000000000 |
| ENSG00000238103 | 0.79027 | 10.82774 | -3.77624 | 0.000000000000 | 0.000000000000 |
| ENSG00000134255 | 3.890983 | 9.059078 | -1.21923 | 0.000000000000 | 0.000000000000 |
| ENSG00000166479 | 1.537216 | 5.041584 | -1.71356 | 0.000000000000 | 0.000000000000 |
| ENSG00000198835 | 3.239061 | 8.719253 | -1.42863 | 0.000000000000 | 0.000000000000 |
| ENSG00000171658 | 2.529187 | 10.49249 | -2.05261 | 0.000000000000 | 0.000000000000 |
| ENSG00000072364 | 43.43691 | 16.09897 | 1.431953 | 0.000000000000 | 0.000000000000 |
| ENSG00000164144 | 2.285794 | 10.36365 | -2.18077 | 0.000000000000 | 0.000000000000 |
| ENSG00000165795 | 19.74725 | 9.08381 | 1.120282 | 0.000000000000 | 0.000000000000 |
| ENSG00000113460 | 10.29301 | 24.54118 | -1.25354 | 0.000000000000 | 0.000000000000 |
| ENSG00000164654 | 5.145555 | 10.60404 | -1.04322 | 0.000000000000 | 0.000000000000 |
| ENSG00000137040 | 3.593587 | 7.19355 | -1.00128 | 0.000000000000 | 0.000000000000 |
| ENSG00000127334 | 16.29204 | 3.93414 | 2.050047 | 0.000000000000 | 0.000000000000 |
| ENSG00000107938 | 6.363906 | 14.6009 | -1.19807 | 0.000000000000 | 0.000000000000 |
| ENSG00000183137 | 1.459605 | 10.52179 | -2.84973 | 0.000000000000 | 0.000000000000 |
| ENSG00000179168 | 5.8866 | 1.643556 | 1.840614 | 0.000000000000 | 0.000000000000 |
| ENSG00000170502 | 3.928336 | 9.352581 | -1.25145 | 0.000000000000 | 0.000000000000 |
| ENSG00000266711 | 2.907519 | 16.48018 | -2.50287 | 0.000000000000 | 0.000000000000 |
| ENSG00000142856 | 3.92174 | 13.69874 | -1.80448 | 0.000000000000 | 0.000000000000 |
| ENSG00000143155 | 10.50484 | 21.18298 | -1.01185 | 0.000000000000 | 0.000000000000 |
| ENSG00000259529 | 5.777324 | 2.704066 | 1.095271 | 0.000000000000 | 0.000000000000 |
| ENSG00000198301 | 3.691623 | 9.688518 | -1.39202 | 0.000000000000 | 0.000000000000 |
| ENSG00000117000 | 2.665231 | 5.344523 | -1.0038 | 0.000000000000 | 0.000000000000 |
| ENSG00000283378 | 7.460965 | 2.104565 | 1.82584 | 0.000000000000 | 0.000000000000 |
| ENSG00000214391 | 3.761701 | 10.75627 | -1.51572 | 0.000000000000 | 0.000000000000 |
| ENSG00000205238 | 10.59595 | 3.713392 | 1.512703 | 0.000000000000 | 0.000000000000 |
| ENSG00000067334 | 1.643636 | 9.101192 | -2.46916 | 0.000000000000 | 0.000000000000 |
| ENSG00000163584 | 1.811353 | 10.67868 | -2.55959 | 0.000000000000 | 0.000000000000 |
| ENSG00000273768 | 48.63367 | 7.345079 | 2.727105 | 0.000000000000 | 0.000000000000 |
| ENSG00000140044 | 5.333854 | 13.32521 | -1.32091 | 0.000000000000 | 0.000000000000 |
| ENSG00000280213 | 6.540475 | 2.856779 | 1.195006 | 0.000000000000 | 0.000000000000 |
| ENSG00000125538 | 1.521457 | 6.869235 | -2.1747 | 0.000000000000 | 0.000000000000 |
| ENSG00000163701 | 7.092174 | 2.225413 | 1.672155 | 0.000000000000 | 0.000000000000 |
| ENSG00000100867 | 2.43554 | 8.322936 | -1.77285 | 0.000000000000 | 0.000000000000 |
| ENSG00000227630 | 5.438967 | 2.221671 | 1.291687 | 0.000000000000 | 0.000000000000 |
| ENSG00000197153 | 5.010644 | 19.64592 | -1.97116 | 0.000000000000 | 0.000000000000 |
| ENSG00000218227 | 2.208588 | 13.77531 | -2.64089 | 0.000000000000 | 0.000000000000 |
| ENSG00000259642 | 14.12833 | 1.705501 | 3.050323 | 0.000000000000 | 0.000000000000 |
| ENSG00000174780 | 38.80032 | 86.16358 | -1.15101 | 0.000000000000 | 0.000000000000 |
| ENSG00000110429 | 4.707997 | 10.91206 | -1.21274 | 0.000000000000 | 0.000000000000 |
| ENSG00000277161 | 1.738946 | 5.803676 | -1.73875 | 0.000000000000 | 0.000000000000 |
| ENSG00000149308 | 4.737185 | 11.86531 | -1.32465 | 0.000000000000 | 0.000000000000 |
| ENSG00000223401 | 6.152895 | 0.788443 | 2.964187 | 0.000000000000 | 0.000000000000 |
| ENSG00000168917 | 2.54999 | 8.257212 | -1.69516 | 0.000000000000 | 0.000000000000 |
| ENSG00000112531 | 9.179996 | 24.19811 | -1.39833 | 0.000000000000 | 0.000000000000 |
| ENSG00000189423 | 34.04974 | 16.53068 | 1.042498 | 0.000000000000 | 0.000000000000 |
| ENSG00000005700 | 3.877077 | 7.91855 | -1.03027 | 0.000000000000 | 0.000000000000 |
| ENSG00000215256 | 5.17733 | 11.42825 | -1.14232 | 0.000000000000 | 0.000000000000 |
| ENSG00000138050 | 2.017573 | 11.37132 | -2.49471 | 0.000000000000 | 0.000000000000 |
| ENSG00000179965 | 10.27543 | 4.492075 | 1.193744 | 0.000000000000 | 0.000000000000 |
| ENSG00000278224 | 12.31181 | 3.746694 | 1.716353 | 0.000000000000 | 0.000000000000 |
| ENSG00000164142 | 21.62104 | 9.147001 | 1.241065 | 0.000000000000 | 0.000000000000 |
| ENSG00000138767 | 2.14731 | 7.929417 | -1.88468 | 0.000000000000 | 0.000000000000 |
| ENSG00000113638 | 6.789518 | 14.08427 | -1.0527 | 0.000000000000 | 0.000000000000 |
| ENSG00000134202 | 60.45445 | 122.6452 | -1.02057 | 0.000000000000 | 0.000000000000 |
| ENSG00000204257 | 26.01135 | 12.19137 | 1.093281 | 0.000000000000 | 0.000000000000 |
| ENSG00000120860 | 5.315433 | 18.38129 | -1.78998 | 0.000000000000 | 0.000000000000 |
| ENSG00000204072 | 4.574985 | 16.46522 | -1.84758 | 0.000000000000 | 0.000000000000 |
| ENSG00000259212 | 30.48429 | 10.16858 | 1.583948 | 0.000000000000 | 0.000000000000 |
| ENSG00000196296 | 8.033773 | 2.539426 | 1.661575 | 0.000000000000 | 0.000000000000 |
| ENSG00000010310 | 7.850221 | 3.187011 | 1.300529 | 0.000000000000 | 0.000000000000 |
| ENSG00000162972 | 14.12972 | 5.77296 | 1.29135 | 0.000000000000 | 0.000000000000 |
| ENSG00000109321 | 2.846392 | 9.976126 | -1.80935 | 0.000000000000 | 0.000000000000 |
| ENSG00000127328 | 2.9053 | 6.89444 | -1.24675 | 0.000000000000 | 0.000000000000 |
| ENSG00000213341 | 3.974698 | 13.49196 | -1.76318 | 0.000000000000 | 0.000000000000 |
| ENSG00000163539 | 5.869508 | 12.07264 | -1.04043 | 0.000000000000 | 0.000000000000 |
| ENSG00000059804 | 2.441288 | 7.904806 | -1.69509 | 0.000000000000 | 0.000000000000 |
| ENSG00000064703 | 3.315705 | 6.817692 | -1.03997 | 0.000000000000 | 0.000000000000 |
| ENSG00000144034 | 13.0725 | 30.7405 | -1.23361 | 0.000000000000 | 0.000000000000 |
| ENSG00000075131 | 5.131306 | 12.34071 | -1.26603 | 0.000000000000 | 0.000000000000 |
| ENSG00000278233 | 101.0632 | 36.0295 | 1.488007 | 0.000000000000 | 0.000000000000 |
| ENSG00000176273 | 3.43298 | 7.318731 | -1.09213 | 0.000000000000 | 0.000000000000 |
| ENSG00000167701 | 10.17758 | 3.677182 | 1.468722 | 0.000000000000 | 0.000000000000 |
| ENSG00000156453 | 7.310986 | 19.08578 | -1.38436 | 0.000000000000 | 0.000000000000 |
| ENSG00000149489 | 16.59826 | 7.747324 | 1.099262 | 0.000000000000 | 0.000000000000 |
| ENSG00000268460 | 5.309902 | 13.01766 | -1.29371 | 0.000000000000 | 0.000000000000 |
| ENSG00000240682 | 21.96449 | 49.97912 | -1.18615 | 0.000000000000 | 0.000000000000 |
| ENSG00000266933 | 15.79054 | 5.129048 | 1.622297 | 0.000000000000 | 0.000000000000 |
| ENSG00000251537 | 2.470716 | 5.05874 | -1.03385 | 0.000000000000 | 0.000000000000 |
| ENSG00000261840 | 18.45657 | 5.014102 | 1.880071 | 0.000000000000 | 0.000000000000 |
| ENSG00000105929 | 0.983825 | 5.485311 | -2.4791 | 0.000000000000 | 0.000000000000 |
| ENSG00000114098 | 15.04215 | 30.91826 | -1.03945 | 0.000000000000 | 0.000000000000 |
| ENSG00000087266 | 87.84174 | 38.49459 | 1.190251 | 0.000000000000 | 0.000000000000 |
| ENSG00000278921 | 9.220835 | 2.069931 | 2.155315 | 0.000000000000 | 0.000000000000 |
| ENSG00000145908 | 2.385957 | 5.665204 | -1.24756 | 0.000000000000 | 0.000000000000 |
| ENSG00000130962 | 1.780899 | 6.445395 | -1.85566 | 0.000000000000 | 0.000000000000 |
| ENSG00000170248 | 87.56469 | 35.07386 | 1.319953 | 0.000000000000 | 0.000000000000 |
| ENSG00000223361 | 0.782331 | 8.072351 | -3.36714 | 0.000000000000 | 0.000000000000 |
| ENSG00000164331 | 1.703695 | 6.257392 | -1.87689 | 0.000000000000 | 0.000000000000 |
| ENSG00000196793 | 3.312091 | 8.023599 | -1.27651 | 0.000000000000 | 0.000000000000 |
| ENSG00000173678 | 9.221017 | 2.835286 | 1.701432 | 0.000000000000 | 0.000000000000 |
| ENSG00000083544 | 2.702563 | 7.012933 | -1.37569 | 0.000000000000 | 0.000000000000 |
| ENSG00000160325 | 15.24444 | 7.151012 | 1.092064 | 0.000000000000 | 0.000000000000 |
| ENSG00000085433 | 2.449774 | 5.486465 | -1.16323 | 0.000000000000 | 0.000000000000 |
| ENSG00000134070 | 3.562474 | 7.420543 | -1.05865 | 0.000000000000 | 0.000000000000 |
| ENSG00000137825 | 8.229597 | 3.713616 | 1.147997 | 0.000000000000 | 0.000000000000 |
| ENSG00000267261 | 10.80118 | 2.916716 | 1.888771 | 0.000000000000 | 0.000000000000 |
| ENSG00000105514 | 6.163547 | 2.948264 | 1.063895 | 0.000000000000 | 0.000000000000 |
| ENSG00000129354 | 8.707485 | 18.57754 | -1.09323 | 0.000000000000 | 0.000000000000 |
| ENSG00000138600 | 3.613986 | 15.82031 | -2.13011 | 0.000000000000 | 0.000000000000 |
| ENSG00000113838 | 5.676341 | 12.85382 | -1.17916 | 0.000000000000 | 0.000000000000 |
| ENSG00000162694 | 1.775669 | 5.387743 | -1.60132 | 0.000000000000 | 0.000000000000 |
| ENSG00000271672 | 0.01226 | 7.628987 | -9.28139 | 0.000000000000 | 0.000000000000 |
| ENSG00000225972 | 0.592135 | 12.42344 | -4.391 | 0.000000000000 | 0.000000000000 |
| ENSG00000164414 | 2.332011 | 6.134789 | -1.39544 | 0.000000000000 | 0.000000000000 |
| ENSG00000170791 | 4.300628 | 11.74807 | -1.4498 | 0.000000000000 | 0.000000000000 |
| ENSG00000180376 | 2.62118 | 10.60671 | -2.01669 | 0.000000000000 | 0.000000000000 |
| ENSG00000181904 | 9.162646 | 4.578827 | 1.000786 | 0.000000000000 | 0.000000000000 |
| ENSG00000137070 | 12.25534 | 4.423114 | 1.470276 | 0.000000000000 | 0.000000000000 |
| ENSG00000151881 | 0.804982 | 5.287328 | -2.71551 | 0.000000000000 | 0.000000000000 |
| ENSG00000186301 | 9.883927 | 4.609383 | 1.100511 | 0.000000000000 | 0.000000000000 |
| ENSG00000125772 | 1.852608 | 6.776763 | -1.87104 | 0.000000000000 | 0.000000000000 |
| ENSG00000213213 | 8.780873 | 2.436442 | 1.849589 | 0.000000000000 | 0.000000000000 |
| ENSG00000113070 | 4.413646 | 9.560733 | -1.11515 | 0.000000000000 | 0.000000000000 |
| ENSG00000228172 | 5.113675 | 2.008988 | 1.347892 | 0.000000000000 | 0.000000000000 |
| ENSG00000138032 | 6.387675 | 13.88351 | -1.12001 | 0.000000000000 | 0.000000000000 |
| ENSG00000113272 | 6.285856 | 13.59989 | -1.11341 | 0.000000000000 | 0.000000000000 |
| ENSG00000197279 | 1.883223 | 5.871957 | -1.64064 | 0.000000000000 | 0.000000000000 |
| ENSG00000076053 | 4.910827 | 11.16451 | -1.18488 | 0.000000000000 | 0.000000000000 |
| ENSG00000144566 | 8.895329 | 18.74278 | -1.07521 | 0.000000000000 | 0.000000000000 |
| ENSG00000176912 | 31.79352 | 13.40282 | 1.246197 | 0.000000000000 | 0.000000000000 |
| ENSG00000102390 | 1.454383 | 6.767538 | -2.21822 | 0.000000000000 | 0.000000000000 |
| ENSG00000183929 | 19.84813 | 9.420673 | 1.075101 | 0.000000000000 | 0.000000000000 |
| ENSG00000197935 | 2.653442 | 6.4062 | -1.2716 | 0.000000000000 | 0.000000000000 |
| ENSG00000236824 | 100.2192 | 44.87355 | 1.159222 | 0.000000000000 | 0.000000000000 |
| ENSG00000175606 | 5.008151 | 10.64378 | -1.08766 | 0.000000000000 | 0.000000000000 |
| ENSG00000247240 | 6.920308 | 2.650153 | 1.384761 | 0.000000000000 | 0.000000000000 |
| ENSG00000139926 | 4.675444 | 9.535909 | -1.02827 | 0.000000000000 | 0.000000000000 |
| ENSG00000225345 | 6.899866 | 0.647332 | 3.413991 | 0.000000000000 | 0.000000000000 |
| ENSG00000165474 | 5.703932 | 11.44673 | -1.00491 | 0.000000000000 | 0.000000000000 |
| ENSG00000198237 | 26.22248 | 10.69101 | 1.294407 | 0.000000000000 | 0.000000000000 |
| ENSG00000112237 | 4.826408 | 10.30321 | -1.09407 | 0.000000000000 | 0.000000000000 |
| ENSG00000253669 | 6.266154 | 15.97652 | -1.3503 | 0.000000000000 | 0.000000000000 |
| ENSG00000167380 | 5.380199 | 12.31564 | -1.19476 | 0.000000000000 | 0.000000000000 |
| ENSG00000122644 | 8.494246 | 19.7951 | -1.22059 | 0.000000000000 | 0.000000000000 |
| ENSG00000131732 | 7.65689 | 16.10389 | -1.07258 | 0.000000000000 | 0.000000000000 |
| ENSG00000104164 | 12.06963 | 5.436001 | 1.150764 | 0.000000000000 | 0.000000000000 |
| ENSG00000188293 | 1.194223 | 7.930596 | -2.73136 | 0.000000000000 | 0.000000000000 |
| ENSG00000207389 | 35.58387 | 5.092112 | 2.804888 | 0.000000000000 | 0.000000000000 |
| ENSG00000186654 | 21.46833 | 10.04303 | 1.096015 | 0.000000000000 | 0.000000000000 |
| ENSG00000073584 | 25.81376 | 57.40762 | -1.1531 | 0.000000000000 | 0.000000000000 |
| ENSG00000145860 | 16.01082 | 34.28485 | -1.09852 | 0.000000000000 | 0.000000000000 |
| ENSG00000182600 | 8.382902 | 2.833187 | 1.565024 | 0.000000000000 | 0.000000000000 |
| ENSG00000272933 | 8.421267 | 2.755415 | 1.611768 | 0.000000000000 | 0.000000000000 |
| ENSG00000164291 | 2.535358 | 6.386237 | -1.33278 | 0.000000000000 | 0.000000000000 |
| ENSG00000065243 | 5.103168 | 14.73801 | -1.53008 | 0.000000000000 | 0.000000000000 |
| ENSG00000281991 | 1.082554 | 6.416695 | -2.56739 | 0.000000000000 | 0.000000000000 |
| ENSG00000136828 | 5.277688 | 1.203023 | 2.133242 | 0.000000000000 | 0.000000000000 |
| ENSG00000231991 | 9.035723 | 19.78311 | -1.13056 | 0.000000000000 | 0.000000000000 |
| ENSG00000138650 | 8.128849 | 2.59962 | 1.64475 | 0.000000000000 | 0.000000000000 |
| ENSG00000257698 | 6.733266 | 20.82068 | -1.62864 | 0.000000000000 | 0.000000000000 |
| ENSG00000165501 | 4.902782 | 13.17004 | -1.42559 | 0.000000000000 | 0.000000000000 |
| ENSG00000224631 | 0.270782 | 8.385513 | -4.9527 | 0.000000000000 | 0.000000000000 |
| ENSG00000160323 | 5.253492 | 1.371426 | 1.9376 | 0.000000000000 | 0.000000000000 |
| ENSG00000162526 | 6.62832 | 1.410583 | 2.232352 | 0.000000000000 | 0.000000000000 |
| ENSG00000206652 | 35.46704 | 5.42847 | 2.707861 | 0.000000000000 | 0.000000000000 |
| ENSG00000265096 | 6.115916 | 16.75563 | -1.45401 | 0.000000000000 | 0.000000000000 |
| ENSG00000237499 | 9.855569 | 22.26475 | -1.17575 | 0.000000000000 | 0.000000000000 |
| ENSG00000007944 | 5.948138 | 1.916735 | 1.633787 | 0.000000000000 | 0.000000000000 |
| ENSG00000186847 | 3.417845 | 8.898859 | -1.38053 | 0.000000000000 | 0.000000000000 |
| ENSG00000240370 | 18.07726 | 6.21278 | 1.540865 | 0.000000000000 | 0.000000000000 |
| ENSG00000163818 | 6.447609 | 15.00215 | -1.21833 | 0.000000000000 | 0.000000000000 |
| ENSG00000213621 | 5.598995 | 15.04917 | -1.42644 | 0.000000000000 | 0.000000000000 |
| ENSG00000228873 | 25.97111 | 3.091345 | 3.070601 | 0.000000000000 | 0.000000000000 |
| ENSG00000274615 | 5.832804 | 13.41947 | -1.20207 | 0.000000000000 | 0.000000000000 |
| ENSG00000189143 | 1.690582 | 6.120054 | -1.85602 | 0.000000000000 | 0.000000000000 |
| ENSG00000023287 | 3.470253 | 11.47335 | -1.72517 | 0.000000000000 | 0.000000000000 |
| ENSG00000205885 | 32.45983 | 14.96582 | 1.116984 | 0.000000000000 | 0.000000000000 |
| ENSG00000163681 | 27.5262 | 55.67153 | -1.01613 | 0.000000000000 | 0.000000000000 |
| ENSG00000160781 | 15.87603 | 7.929776 | 1.001498 | 0.000000000000 | 0.000000000000 |
| ENSG00000118849 | 13.94246 | 6.480159 | 1.105384 | 0.000000000000 | 0.000000000000 |
| ENSG00000206588 | 34.38454 | 5.356251 | 2.682465 | 0.000000000000 | 0.000000000000 |
| ENSG00000171202 | 8.27782 | 20.94439 | -1.33924 | 0.000000000000 | 0.000000000000 |
| ENSG00000167550 | 12.49072 | 26.23334 | -1.07054 | 0.000000000000 | 0.000000000000 |
| ENSG00000260025 | 0.859548 | 11.45244 | -3.73593 | 0.000000000000 | 0.000000000000 |
| ENSG00000137802 | 17.96567 | 8.484121 | 1.082405 | 0.000000000000 | 0.000000000000 |
| ENSG00000108423 | 3.653237 | 9.503835 | -1.37933 | 0.000000000000 | 0.000000000000 |
| ENSG00000213906 | 12.47036 | 4.807867 | 1.375035 | 0.000000000000 | 0.000000000000 |
| ENSG00000132825 | 5.902683 | 2.93838 | 1.00635 | 0.000000000000 | 0.000000000000 |
| ENSG00000140299 | 17.71356 | 8.692685 | 1.02698 | 0.000000000000 | 0.000000000000 |
| ENSG00000110203 | 2.125032 | 9.071077 | -2.09379 | 0.000000000000 | 0.000000000000 |
| ENSG00000106460 | 5.360312 | 11.64219 | -1.11897 | 0.000000000000 | 0.000000000000 |
| ENSG00000249936 | 4.610695 | 15.82926 | -1.77954 | 0.000000000000 | 0.000000000000 |
| ENSG00000067533 | 2.586544 | 5.192795 | -1.00549 | 0.000000000000 | 0.000000000000 |
| ENSG00000155324 | 10.11954 | 21.2971 | -1.07351 | 0.000000000000 | 0.000000000000 |
| ENSG00000233237 | 8.871744 | 2.618081 | 1.760708 | 0.000000000000 | 0.000000000000 |
| ENSG00000198586 | 3.075891 | 6.197594 | -1.0107 | 0.000000000000 | 0.000000000000 |
| ENSG00000094841 | 18.9429 | 4.924454 | 1.943621 | 0.000000000000 | 0.000000000000 |
| ENSG00000177946 | 3.429485 | 7.21451 | -1.07291 | 0.000000000000 | 0.000000000000 |
| ENSG00000250995 | 8.046472 | 2.285422 | 1.815896 | 0.000000000000 | 0.000000000000 |
| ENSG00000139726 | 39.54642 | 14.28523 | 1.469022 | 0.000000000000 | 0.000000000000 |
| ENSG00000043093 | 2.189152 | 5.216032 | -1.25258 | 0.000000000000 | 0.000000000000 |
| ENSG00000101076 | 6.705798 | 2.313282 | 1.535468 | 0.000000000000 | 0.000000000000 |
| ENSG00000245768 | 6.120675 | 0.0001 | 15.9014 | 0.000000000000 | 0.000000000000 |
| ENSG00000184163 | 28.11573 | 13.47589 | 1.060997 | 0.000000000000 | 0.000000000000 |
| ENSG00000009950 | 7.426675 | 2.275943 | 1.706252 | 0.000000000000 | 0.000000000000 |
| ENSG00000104894 | 6.137887 | 1.89159 | 1.698143 | 0.000000000000 | 0.000000000000 |
| ENSG00000151743 | 3.966227 | 9.85745 | -1.31345 | 0.000000000000 | 0.000000000000 |
| ENSG00000176842 | 6.701625 | 3.194645 | 1.068855 | 0.000000000000 | 0.000000000000 |
| ENSG00000108602 | 13.69969 | 6.836739 | 1.002763 | 0.000000000000 | 0.000000000000 |
| ENSG00000163536 | 4.423985 | 10.51224 | -1.24865 | 0.000000000000 | 0.000000000000 |
| ENSG00000104953 | 13.65581 | 28.55182 | -1.06407 | 0.000000000000 | 0.000000000000 |
| ENSG00000034693 | 8.037875 | 18.77939 | -1.22426 | 0.000000000000 | 0.000000000000 |
| ENSG00000200795 | 24.28644 | 0.0001 | 17.88979 | 0.000000000000 | 0.000000000000 |
| ENSG00000179776 | 8.229386 | 3.954115 | 1.05743 | 0.000000000000 | 0.000000000000 |
| ENSG00000131943 | 7.575336 | 16.58256 | -1.13028 | 0.000000000000 | 0.000000000000 |
| ENSG00000163738 | 7.988606 | 3.659946 | 1.126121 | 0.000000000000 | 0.000000000000 |
| ENSG00000120075 | 9.564085 | 4.730661 | 1.015585 | 0.000000000000 | 0.000000000000 |
| ENSG00000133997 | 3.676228 | 11.15076 | -1.60084 | 0.000000000000 | 0.000000000000 |
| ENSG00000177873 | 2.274233 | 5.397541 | -1.24692 | 0.000000000000 | 0.000000000000 |
| ENSG00000259823 | 1.702448 | 6.33596 | -1.89595 | 0.000000000000 | 0.000000000000 |
| ENSG00000113163 | 4.519925 | 12.00289 | -1.40901 | 0.000000000000 | 0.000000000000 |
| ENSG00000233954 | 3.987155 | 8.443203 | -1.08243 | 0.000000000000 | 0.000000000000 |
| ENSG00000172748 | 0.34297 | 5.967116 | -4.12088 | 0.000000000000 | 0.000000000000 |
| ENSG00000181315 | 2.235632 | 7.456051 | -1.73773 | 0.000000000000 | 0.000000000000 |
| ENSG00000104833 | 9.063278 | 2.84107 | 1.673599 | 0.000000000000 | 0.000000000000 |
| ENSG00000227258 | 1.346512 | 8.821733 | -2.71184 | 0.000000000000 | 0.000000000000 |
| ENSG00000147650 | 3.217832 | 11.47941 | -1.83489 | 0.000000000000 | 0.000000000000 |
| ENSG00000200087 | 42.40067 | 12.91948 | 1.714539 | 0.000000000000 | 0.000000000000 |
| ENSG00000008516 | 5.548322 | 0.941832 | 2.55851 | 0.000000000000 | 0.000000000000 |
| ENSG00000153339 | 1.767272 | 5.474723 | -1.63126 | 0.000000000000 | 0.000000000000 |
| ENSG00000188747 | 10.31688 | 4.883162 | 1.07912 | 0.000000000000 | 0.000000000000 |
| ENSG00000147118 | 2.790576 | 5.634478 | -1.01372 | 0.000000000000 | 0.000000000000 |
| ENSG00000237004 | 17.79557 | 5.010737 | 1.828423 | 0.000000000000 | 0.000000000000 |
| ENSG00000189050 | 14.20427 | 6.409154 | 1.148118 | 0.000000000000 | 0.000000000000 |
| ENSG00000154719 | 15.33162 | 31.60453 | -1.04362 | 0.000000000000 | 0.000000000000 |
| ENSG00000077157 | 16.19799 | 4.301095 | 1.913038 | 0.000000000000 | 0.000000000000 |
| ENSG00000212664 | 0.869518 | 7.657707 | -3.13862 | 0.000000000000 | 0.000000000000 |
| ENSG00000274267 | 38.70984 | 17.7346 | 1.126134 | 0.000000000000 | 0.000000000000 |
| ENSG00000278771 | 20.09349 | 4.314457 | 2.219477 | 0.000000000000 | 0.000000000000 |
| ENSG00000111358 | 5.381451 | 13.20418 | -1.29493 | 0.000000000000 | 0.000000000000 |
| ENSG00000206573 | 5.249478 | 17.9166 | -1.77105 | 0.000000000000 | 0.000000000000 |
| ENSG00000155792 | 5.627152 | 2.347173 | 1.261481 | 0.000000000000 | 0.000000000000 |
| ENSG00000274808 | 13.37833 | 5.308017 | 1.333653 | 0.000000000000 | 0.000000000000 |
| ENSG00000129534 | 2.659814 | 10.0183 | -1.91324 | 0.000000000000 | 0.000000000000 |
| ENSG00000178295 | 3.533287 | 7.772146 | -1.1373 | 0.000000000000 | 0.000000000000 |
| ENSG00000175322 | 8.129562 | 4.003283 | 1.021994 | 0.000000000000 | 0.000000000000 |
| ENSG00000144395 | 5.769016 | 13.95644 | -1.27453 | 0.000000000000 | 0.000000000000 |
| ENSG00000168522 | 45.67833 | 17.32785 | 1.398417 | 0.000000000000 | 0.000000000000 |
| ENSG00000187650 | 5.792694 | 2.440634 | 1.246978 | 0.000000000000 | 0.000000000000 |
| ENSG00000188223 | 1.673185 | 6.640342 | -1.98866 | 0.000000000000 | 0.000000000000 |
| ENSG00000103351 | 7.714275 | 16.07472 | -1.05919 | 0.000000000000 | 0.000000000000 |
| ENSG00000260367 | 5.732032 | 1.805991 | 1.666256 | 0.000000000000 | 0.000000000000 |
| ENSG00000163633 | 7.387513 | 15.71704 | -1.08917 | 0.000000000000 | 0.000000000000 |
| ENSG00000006606 | 12.40329 | 4.403841 | 1.493888 | 0.000000000000 | 0.000000000000 |
| ENSG00000226564 | 0.589731 | 6.682032 | -3.50216 | 0.000000000000 | 0.000000000000 |
| ENSG00000153485 | 6.282173 | 14.26167 | -1.18281 | 0.000000000000 | 0.000000000000 |
| ENSG00000243885 | 1.155832 | 5.641873 | -2.28724 | 0.000000000000 | 0.000000000000 |
| ENSG00000108785 | 11.67812 | 4.535041 | 1.364621 | 0.000000000000 | 0.000000000000 |
| ENSG00000267270 | 12.45694 | 4.582653 | 1.442695 | 0.000000000000 | 0.000000000000 |
| ENSG00000083812 | 3.403609 | 6.995942 | -1.03945 | 0.000000000000 | 0.000000000000 |
| ENSG00000105865 | 1.997903 | 5.652433 | -1.50039 | 0.000000000000 | 0.000000000000 |
| ENSG00000102738 | 4.340688 | 10.32792 | -1.25055 | 0.000000000000 | 0.000000000000 |
| ENSG00000229656 | 6.388167 | 14.24489 | -1.15697 | 0.000000000000 | 0.000000000000 |
| ENSG00000235316 | 6.87023 | 2.886902 | 1.250836 | 0.000000000000 | 0.000000000000 |
| ENSG00000174151 | 8.804449 | 3.89475 | 1.176702 | 0.000000000000 | 0.000000000000 |
| ENSG00000230383 | 1.28692 | 6.305604 | -2.29271 | 0.000000000000 | 0.000000000000 |
| ENSG00000156017 | 9.149302 | 4.034161 | 1.181393 | 0.000000000000 | 0.000000000000 |
| ENSG00000249962 | 17.22574 | 6.603388 | 1.383287 | 0.000000000000 | 0.000000000000 |
| ENSG00000133773 | 11.76467 | 26.00059 | -1.14408 | 0.000000000000 | 0.000000000000 |
| ENSG00000114520 | 8.964672 | 19.87314 | -1.1485 | 0.000000000000 | 0.000000000000 |
| ENSG00000143156 | 2.771289 | 6.686331 | -1.27066 | 0.000000000000 | 0.000000000000 |
| ENSG00000242498 | 28.13941 | 13.89525 | 1.018 | 0.000000000000 | 0.000000000000 |
| ENSG00000144357 | 3.159203 | 6.788649 | -1.10356 | 0.000000000000 | 0.000000000000 |
| ENSG00000235703 | 9.236654 | 4.189855 | 1.14047 | 0.000000000000 | 0.000000000000 |
| ENSG00000086189 | 6.053423 | 13.6363 | -1.17163 | 0.000000000000 | 0.000000000000 |
| ENSG00000047457 | 5.279018 | 1.898502 | 1.475408 | 0.000000000000 | 0.000000000000 |
| ENSG00000262664 | 14.78464 | 7.281734 | 1.021745 | 0.000000000000 | 0.000000000000 |
| ENSG00000228521 | 11.31501 | 1.624425 | 2.800237 | 0.000000000000 | 0.000000000000 |
| ENSG00000228314 | 5.089342 | 0.839259 | 2.600291 | 0.000000000000 | 0.000000000000 |
| ENSG00000166352 | 8.693187 | 18.26316 | -1.07098 | 0.000000000000 | 0.000000000000 |
| ENSG00000100558 | 4.783448 | 10.68423 | -1.15936 | 0.000000000000 | 0.000000000000 |
| ENSG00000169684 | 2.750717 | 7.143097 | -1.37674 | 0.000000000000 | 0.000000000000 |
| ENSG00000059728 | 22.24782 | 6.931637 | 1.682396 | 0.000000000000 | 0.000000000000 |
| ENSG00000206596 | 29.09734 | 5.314462 | 2.452892 | 0.000000000000 | 0.000000000000 |
| ENSG00000135002 | 2.591318 | 5.880957 | -1.18236 | 0.000000000000 | 0.000000000000 |
| ENSG00000118939 | 5.84225 | 14.1613 | -1.27736 | 0.000000000000 | 0.000000000000 |
| ENSG00000271810 | 0.668923 | 5.061922 | -2.91977 | 0.000000000000 | 0.000000000000 |
| ENSG00000244128 | 1.119979 | 7.536037 | -2.75033 | 0.000000000000 | 0.000000000000 |
| ENSG00000166407 | 9.806388 | 3.222923 | 1.605352 | 0.000000000000 | 0.000000000000 |
| ENSG00000267769 | 11.46362 | 2.767907 | 2.050195 | 0.000000000000 | 0.000000000000 |
| ENSG00000206344 | 8.223682 | 18.76976 | -1.19055 | 0.000000000000 | 0.000000000000 |
| ENSG00000090989 | 3.180321 | 13.40419 | -2.07544 | 0.000000000000 | 0.000000000000 |
| ENSG00000261423 | 5.163479 | 2.30274 | 1.164992 | 0.000000000000 | 0.000000000000 |
| ENSG00000125726 | 6.748214 | 1.148674 | 2.554536 | 0.000000000000 | 0.000000000000 |
| ENSG00000197757 | 4.742282 | 9.604606 | -1.01814 | 0.000000000000 | 0.000000000000 |
| ENSG00000133302 | 10.81076 | 24.31107 | -1.16915 | 0.000000000000 | 0.000000000000 |
| ENSG00000083123 | 14.31932 | 5.786042 | 1.307314 | 0.000000000000 | 0.000000000000 |
| ENSG00000122958 | 2.395464 | 5.28142 | -1.14062 | 0.000000000000 | 0.000000000000 |
| ENSG00000278662 | 8.407193 | 2.002964 | 2.069488 | 0.000000000000 | 0.000000000000 |
| ENSG00000056050 | 3.156985 | 6.604448 | -1.06489 | 0.000000000000 | 0.000000000000 |
| ENSG00000273812 | 23.41625 | 11.21883 | 1.061588 | 0.000000000000 | 0.000000000000 |
| ENSG00000162894 | 2.521666 | 8.205155 | -1.70215 | 0.000000000000 | 0.000000000000 |
| ENSG00000156239 | 2.318465 | 5.946331 | -1.35883 | 0.000000000000 | 0.000000000000 |
| ENSG00000128915 | 3.483319 | 10.48989 | -1.59046 | 0.000000000000 | 0.000000000000 |
| ENSG00000109323 | 7.527151 | 17.49695 | -1.21693 | 0.000000000000 | 0.000000000000 |
| ENSG00000230629 | 0.731425 | 9.31902 | -3.6714 | 0.000000000000 | 0.000000000000 |
| ENSG00000175048 | 8.787198 | 2.83857 | 1.630239 | 0.000000000000 | 0.000000000000 |
| ENSG00000164162 | 5.381934 | 12.93307 | -1.26487 | 0.000000000000 | 0.000000000000 |
| ENSG00000242960 | 1.406068 | 7.935834 | -2.49672 | 0.000000000000 | 0.000000000000 |
| ENSG00000210164 | 14.2748 | 70.47916 | -2.30373 | 0.000000000000 | 0.000000000000 |
| ENSG00000253861 | 4.745039 | 9.954647 | -1.06895 | 0.000000000000 | 0.000000000000 |
| ENSG00000172123 | 2.552365 | 5.981301 | -1.22862 | 0.000000000000 | 0.000000000000 |
| ENSG00000200488 | 18.2584 | 5.012724 | 1.864894 | 0.000000000000 | 0.000000000000 |
| ENSG00000244586 | 20.24989 | 8.212643 | 1.301995 | 0.000000000000 | 0.000000000000 |
| ENSG00000112739 | 17.79054 | 41.04059 | -1.20594 | 0.000000000000 | 0.000000000000 |
| ENSG00000115109 | 2.180846 | 8.054525 | -1.88491 | 0.000000000000 | 0.000000000000 |
| ENSG00000010270 | 6.77837 | 13.70178 | -1.01535 | 0.000000000000 | 0.000000000000 |
| ENSG00000279253 | 9.478588 | 4.566836 | 1.053477 | 0.000000000000 | 0.000000000000 |
| ENSG00000198353 | 6.693688 | 2.350395 | 1.509898 | 0.000000000000 | 0.000000000000 |
| ENSG00000276496 | 24.54125 | 0.337197 | 6.185473 | 0.000000000000 | 0.000000000000 |
| ENSG00000279283 | 6.142741 | 2.596406 | 1.242367 | 0.000000000000 | 0.000000000000 |
| ENSG00000171227 | 8.635346 | 4.007533 | 1.10754 | 0.000000000000 | 0.000000000000 |
| ENSG00000281348 | 6.350182 | 0.854877 | 2.893009 | 0.000000000000 | 0.000000000000 |
| ENSG00000163743 | 1.336476 | 7.817003 | -2.54818 | 0.000000000000 | 0.000000000000 |
| ENSG00000237522 | 6.09562 | 2.089149 | 1.544858 | 0.000000000000 | 0.000000000000 |
| ENSG00000166444 | 29.61765 | 14.28162 | 1.052297 | 0.000000000000 | 0.000000000000 |
| ENSG00000215630 | 11.27172 | 3.462613 | 1.702775 | 0.000000000000 | 0.000000000000 |
| ENSG00000197006 | 37.41988 | 12.86291 | 1.540588 | 0.000000000000 | 0.000000000000 |
| ENSG00000213178 | 1.492627 | 9.251516 | -2.63184 | 0.000000000000 | 0.000000000000 |
| ENSG00000118600 | 5.384845 | 10.84753 | -1.01039 | 0.000000000000 | 0.000000000000 |
| ENSG00000092330 | 11.15193 | 24.52818 | -1.13715 | 0.000000000000 | 0.000000000000 |
| ENSG00000267395 | 14.78171 | 5.992477 | 1.302589 | 0.000000000000 | 0.000000000000 |
| ENSG00000182327 | 7.023803 | 2.363617 | 1.571256 | 0.000000000000 | 0.000000000000 |
| ENSG00000205869 | 14.09245 | 6.813282 | 1.0485 | 0.000000000000 | 0.000000000000 |
| ENSG00000125618 | 9.847968 | 4.896807 | 1.007985 | 0.000000000000 | 0.000000000000 |
| ENSG00000121542 | 5.76615 | 14.31185 | -1.31153 | 0.000000000000 | 0.000000000000 |
| ENSG00000231305 | 9.868839 | 4.11621 | 1.261564 | 0.000000000000 | 0.000000000000 |
| ENSG00000147687 | 1.084649 | 5.191864 | -2.25902 | 0.000000000000 | 0.000000000000 |
| ENSG00000170325 | 13.23856 | 6.148675 | 1.106399 | 0.000000000000 | 0.000000000000 |
| ENSG00000168899 | 20.95776 | 10.4732 | 1.000782 | 0.000000000000 | 0.000000000000 |
| ENSG00000167904 | 2.514703 | 6.974092 | -1.47162 | 0.000000000000 | 0.000000000000 |
| ENSG00000277846 | 1.133827 | 33.24933 | -4.87405 | 0.000000000000 | 0.000000000000 |
| ENSG00000115514 | 2.210052 | 6.199316 | -1.48803 | 0.000000000000 | 0.000000000000 |
| ENSG00000132294 | 4.496668 | 12.93819 | -1.52471 | 0.000000000000 | 0.000000000000 |
| ENSG00000242529 | 10.63044 | 0.173477 | 5.937314 | 0.000000000000 | 0.000000000000 |
| ENSG00000121417 | 3.423019 | 6.876799 | -1.00647 | 0.000000000000 | 0.000000000000 |
| ENSG00000198520 | 6.203056 | 1.920349 | 1.691611 | 0.000000000000 | 0.000000000000 |
| ENSG00000256683 | 2.533797 | 6.232355 | -1.29848 | 0.000000000000 | 0.000000000000 |
| ENSG00000236871 | 33.94775 | 15.89871 | 1.094406 | 0.000000000000 | 0.000000000000 |
| ENSG00000204618 | 6.921189 | 3.308785 | 1.064718 | 0.000000000000 | 0.000000000000 |
| ENSG00000128710 | 5.752256 | 2.724718 | 1.078021 | 0.000000000000 | 0.000000000000 |
| ENSG00000266469 | 2.528515 | 7.727119 | -1.61164 | 0.000000000000 | 0.000000000000 |
| ENSG00000119632 | 25.43005 | 11.97608 | 1.086379 | 0.000000000000 | 0.000000000000 |
| ENSG00000281358 | 5.384982 | 1.181633 | 2.188159 | 0.000000000000 | 0.000000000000 |
| ENSG00000271576 | 7.62573 | 3.678878 | 1.05161 | 0.000000000000 | 0.000000000000 |
| ENSG00000162599 | 19.88861 | 8.485997 | 1.228786 | 0.000000000000 | 0.000000000000 |
| ENSG00000150433 | 7.985838 | 16.10572 | -1.01206 | 0.000000000000 | 0.000000000000 |
| ENSG00000238072 | 1.251462 | 11.08068 | -3.14636 | 0.000000000000 | 0.000000000000 |
| ENSG00000241015 | 2.926619 | 6.274798 | -1.10033 | 0.000000000000 | 0.000000000000 |
| ENSG00000242798 | 9.484399 | 2.760906 | 1.780415 | 0.000000000000 | 0.000000000000 |
| ENSG00000073849 | 10.95301 | 5.393002 | 1.022167 | 0.000000000000 | 0.000000000000 |
| ENSG00000111348 | 1.404723 | 5.592893 | -1.99331 | 0.000000000000 | 0.000000000000 |
| ENSG00000051825 | 4.207403 | 14.13181 | -1.74794 | 0.000000000000 | 0.000000000000 |
| ENSG00000177721 | 1.54299 | 5.075704 | -1.71788 | 0.000000000000 | 0.000000000000 |
| ENSG00000273478 | 12.71019 | 5.132246 | 1.308323 | 0.000000000000 | 0.000000000000 |
| ENSG00000265415 | 1.969021 | 11.16543 | -2.50349 | 0.000000000000 | 0.000000000000 |
| ENSG00000145819 | 3.61055 | 8.338859 | -1.20763 | 0.000000000000 | 0.000000000000 |
| ENSG00000117228 | 2.191343 | 5.045105 | -1.20307 | 0.000000000000 | 0.000000000000 |
| ENSG00000239523 | 7.617724 | 16.3062 | -1.09799 | 0.000000000000 | 0.000000000000 |
| ENSG00000230606 | 6.936944 | 3.238047 | 1.099176 | 0.000000000000 | 0.000000000000 |
| ENSG00000069943 | 7.976451 | 17.10506 | -1.1006 | 0.000000000000 | 0.000000000000 |
| ENSG00000250734 | 5.280922 | 0.602636 | 3.131431 | 0.000000000000 | 0.000000000000 |
| ENSG00000146592 | 1.824523 | 6.429279 | -1.81714 | 0.000000000000 | 0.000000000000 |
| ENSG00000230408 | 9.603249 | 3.935106 | 1.28712 | 0.000000000000 | 0.000000000000 |
| ENSG00000165416 | 33.24536 | 12.07049 | 1.461668 | 0.000000000000 | 0.000000000000 |
| ENSG00000169715 | 3.058753 | 8.648646 | -1.49953 | 0.000000000000 | 0.000000000000 |
| ENSG00000131171 | 6.659514 | 3.269634 | 1.026288 | 0.000000000000 | 0.000000000000 |
| ENSG00000180867 | 3.117531 | 6.855273 | -1.13681 | 0.000000000000 | 0.000000000000 |
| ENSG00000271826 | 3.196898 | 8.353145 | -1.38565 | 0.000000000000 | 0.000000000000 |
| ENSG00000275223 | 5.205864 | 1.047535 | 2.313139 | 0.000000000000 | 0.000000000000 |
| ENSG00000235272 | 12.0432 | 27.27293 | -1.17925 | 0.000000000000 | 0.000000000000 |
| ENSG00000226281 | 1.643984 | 7.232504 | -2.1373 | 0.000000000000 | 0.000000000000 |
| ENSG00000276788 | 0.035342 | 26.44595 | -9.54745 | 0.000000000000 | 0.000000000000 |
| ENSG00000111490 | 2.820059 | 6.879131 | -1.2865 | 0.000000000000 | 0.000000000000 |
| ENSG00000094975 | 3.016945 | 7.41678 | -1.2977 | 0.000000000000 | 0.000000000000 |
| ENSG00000186868 | 11.67927 | 4.235698 | 1.463278 | 0.000000000000 | 0.000000000000 |
| ENSG00000109466 | 4.153012 | 8.682355 | -1.06393 | 0.000000000000 | 0.000000000000 |
| ENSG00000188368 | 7.305759 | 3.441985 | 1.085793 | 0.000000000000 | 0.000000000000 |
| ENSG00000233930 | 7.247068 | 3.337484 | 1.118637 | 0.000000000000 | 0.000000000000 |
| ENSG00000059915 | 6.49334 | 2.699056 | 1.266506 | 0.000000000000 | 0.000000000000 |
| ENSG00000152292 | 12.25659 | 5.24998 | 1.223174 | 0.000000000000 | 0.000000000000 |
| ENSG00000115041 | 5.739202 | 1.923926 | 1.576797 | 0.000000000000 | 0.000000000000 |
| ENSG00000274565 | 5.349363 | 1.874485 | 1.512873 | 0.000000000000 | 0.000000000000 |
| ENSG00000233101 | 13.4012 | 5.753159 | 1.219936 | 0.000000000000 | 0.000000000000 |
| ENSG00000128692 | 2.437496 | 6.73293 | -1.46583 | 0.000000000000 | 0.000000000000 |
| ENSG00000158234 | 18.18594 | 8.934488 | 1.025367 | 0.000000000000 | 0.000000000000 |
| ENSG00000256879 | 7.187441 | 1.531287 | 2.230734 | 0.000000000000 | 0.000000000000 |
| ENSG00000265393 | 20.72536 | 7.894921 | 1.392401 | 0.000000000000 | 0.000000000000 |
| ENSG00000209082 | 28.96807 | 83.60369 | -1.5291 | 0.000000000000 | 0.000000000000 |
| ENSG00000251136 | 1.638444 | 5.237578 | -1.67657 | 0.000000000000 | 0.000000000000 |
| ENSG00000151665 | 4.129146 | 10.55697 | -1.35428 | 0.000000000000 | 0.000000000000 |
| ENSG00000236859 | 4.262183 | 8.817524 | -1.04878 | 0.000000000000 | 0.000000000000 |
| ENSG00000197385 | 3.47789 | 7.605809 | -1.12889 | 0.000000000000 | 0.000000000000 |
| ENSG00000166471 | 28.99871 | 10.19409 | 1.508256 | 0.000000000000 | 0.000000000000 |
| ENSG00000233818 | 1.108889 | 5.305207 | -2.25829 | 0.000000000000 | 0.000000000000 |
| ENSG00000167476 | 9.63265 | 4.129899 | 1.221826 | 0.000000000000 | 0.000000000000 |
| ENSG00000263823 | 6.212594 | 2.941444 | 1.078671 | 0.000000000000 | 0.000000000000 |
| ENSG00000249572 | 19.25029 | 9.278969 | 1.052844 | 0.000000000000 | 0.000000000000 |
| ENSG00000235558 | 14.96283 | 5.459654 | 1.454502 | 0.000000000000 | 0.000000000000 |
| ENSG00000266401 | 2.430515 | 5.69095 | -1.22741 | 0.000000000000 | 0.000000000000 |
| ENSG00000257553 | 0.454706 | 5.130368 | -3.49606 | 0.000000000000 | 0.000000000000 |
| ENSG00000262185 | 3.587886 | 7.492631 | -1.06234 | 0.000000000000 | 0.000000000000 |
| ENSG00000182903 | 3.347256 | 7.095875 | -1.084 | 0.000000000000 | 0.000000000000 |
| ENSG00000139323 | 2.417072 | 7.017643 | -1.53773 | 0.000000000000 | 0.000000000000 |
| ENSG00000272173 | 7.318939 | 3.197266 | 1.194796 | 0.000000000000 | 0.000000000000 |
| ENSG00000100802 | 19.26131 | 7.406551 | 1.378832 | 0.000000000000 | 0.000000000000 |
| ENSG00000229124 | 6.701065 | 1.922817 | 1.801169 | 0.000000000000 | 0.000000000000 |
| ENSG00000232818 | 10.44832 | 5.003711 | 1.062201 | 0.000000000000 | 0.000000000000 |
| ENSG00000134982 | 13.06243 | 3.471842 | 1.91165 | 0.000000000000 | 0.000000000000 |
| ENSG00000270412 | 9.305022 | 2.50552 | 1.8929 | 0.000000000000 | 0.000000000000 |
| ENSG00000246982 | 7.09451 | 2.728215 | 1.378746 | 0.000000000000 | 0.000000000000 |
| ENSG00000228589 | 6.182294 | 13.27802 | -1.10283 | 0.000000000000 | 0.000000000000 |
| ENSG00000168228 | 3.91076 | 8.181206 | -1.06486 | 0.000000000000 | 0.000000000000 |
| ENSG00000132429 | 3.764657 | 8.070739 | -1.10018 | 0.000000000000 | 0.000000000000 |
| ENSG00000196302 | 15.1548 | 5.904173 | 1.359968 | 0.000000000000 | 0.000000000000 |
| ENSG00000183527 | 30.69039 | 63.19995 | -1.04214 | 0.000000000000 | 0.000000000000 |
| ENSG00000184451 | 5.03487 | 2.47803 | 1.022761 | 0.000000000000 | 0.000000000000 |
| ENSG00000058063 | 5.19593 | 11.45877 | -1.141 | 0.000000000000 | 0.000000000000 |
| ENSG00000166562 | 21.36005 | 43.24418 | -1.01759 | 0.000000000000 | 0.000000000000 |
| ENSG00000273340 | 5.771571 | 2.351 | 1.29569 | 0.000000000000 | 0.000000000000 |
| ENSG00000145217 | 8.153382 | 1.865185 | 2.12808 | 0.000000000000 | 0.000000000000 |
| ENSG00000205309 | 6.812101 | 3.345147 | 1.02603 | 0.000000000000 | 0.000000000000 |
| ENSG00000225032 | 6.65081 | 2.066342 | 1.686451 | 0.000000000000 | 0.000000000000 |
| ENSG00000123427 | 21.56492 | 8.624612 | 1.322155 | 0.000000000000 | 0.000000000000 |
| ENSG00000254428 | 7.290455 | 1.914115 | 1.929331 | 0.000000000000 | 0.000000000000 |
| ENSG00000227615 | 1.650361 | 7.712019 | -2.22433 | 0.000000000000 | 0.000000000000 |
| ENSG00000163281 | 4.053837 | 8.384085 | -1.04837 | 0.000000000000 | 0.000000000000 |
| ENSG00000156531 | 6.022488 | 12.15684 | -1.01334 | 0.000000000000 | 0.000000000000 |
| ENSG00000135698 | 3.454927 | 7.7761 | -1.17039 | 0.000000000000 | 0.000000000000 |
| ENSG00000241288 | 2.112043 | 6.470905 | -1.61533 | 0.000000000000 | 0.000000000000 |
| ENSG00000135373 | 9.877218 | 4.324632 | 1.191527 | 0.000000000000 | 0.000000000000 |
| ENSG00000278615 | 14.07753 | 6.606804 | 1.09137 | 0.000000000000 | 0.000000000000 |
| ENSG00000132313 | 15.04654 | 39.77919 | -1.40258 | 0.000000000000 | 0.000000000000 |
| ENSG00000262888 | 2.614601 | 7.123475 | -1.44599 | 0.000000000000 | 0.000000000000 |
| ENSG00000239335 | 9.930324 | 4.064051 | 1.288922 | 0.000000000000 | 0.000000000000 |
| ENSG00000278635 | 13.87408 | 5.846819 | 1.246668 | 0.000000000000 | 0.000000000000 |
| ENSG00000155100 | 4.650057 | 11.31067 | -1.28236 | 0.000000000000 | 0.000000000000 |
| ENSG00000155622 | 4.791423 | 11.20168 | -1.22519 | 0.000000000000 | 0.000000000000 |
| ENSG00000152240 | 10.72762 | 22.45451 | -1.06568 | 0.000000000000 | 0.000000000000 |
| ENSG00000256591 | 4.490862 | 12.57109 | -1.48505 | 0.000000000000 | 0.000000000001 |
| ENSG00000121988 | 2.375831 | 7.894409 | -1.7324 | 0.000000000000 | 0.000000000001 |
| ENSG00000127324 | 2.530004 | 6.582814 | -1.37956 | 0.000000000000 | 0.000000000001 |
| ENSG00000230795 | 2.225442 | 5.614767 | -1.33513 | 0.000000000000 | 0.000000000001 |
| ENSG00000174365 | 5.424076 | 12.6703 | -1.224 | 0.000000000000 | 0.000000000001 |
| ENSG00000272768 | 5.468766 | 2.611029 | 1.066597 | 0.000000000000 | 0.000000000001 |
| ENSG00000100918 | 6.024058 | 2.639758 | 1.19033 | 0.000000000001 | 0.000000000002 |
| ENSG00000202363 | 9.060118 | 0.178019 | 5.669427 | 0.000000000001 | 0.000000000003 |
| ENSG00000069696 | 5.147191 | 2.539447 | 1.019271 | 0.000000000001 | 0.000000000003 |
| ENSG00000257489 | 9.227354 | 3.827822 | 1.269393 | 0.000000000001 | 0.000000000004 |
| ENSG00000274471 | 8.946861 | 4.306303 | 1.054932 | 0.000000000001 | 0.000000000004 |
| ENSG00000117906 | 6.103627 | 26.2359 | -2.1038 | 0.000000000001 | 0.000000000005 |
| ENSG00000124657 | 2.883842 | 6.721296 | -1.22075 | 0.000000000001 | 0.000000000005 |
| ENSG00000105650 | 5.832689 | 2.830092 | 1.043312 | 0.000000000002 | 0.000000000006 |
| ENSG00000004838 | 5.557916 | 1.627272 | 1.772089 | 0.000000000002 | 0.000000000006 |
| ENSG00000272473 | 2.951334 | 7.031042 | -1.25237 | 0.000000000002 | 0.000000000006 |
| ENSG00000108443 | 79.2682 | 19.11968 | 2.051684 | 0.000000000002 | 0.000000000006 |
| ENSG00000262413 | 2.352852 | 7.273865 | -1.62831 | 0.000000000002 | 0.000000000007 |
| ENSG00000223773 | 23.73203 | 9.970716 | 1.251066 | 0.000000000002 | 0.000000000008 |
| ENSG00000149089 | 3.16344 | 6.332017 | -1.00117 | 0.000000000002 | 0.000000000008 |
| ENSG00000051620 | 5.789354 | 1.927891 | 1.586379 | 0.000000000002 | 0.000000000009 |
| ENSG00000103111 | 45.8761 | 19.97278 | 1.199708 | 0.000000000004 | 0.000000000015 |
| ENSG00000254706 | 4.197418 | 10.11318 | -1.26866 | 0.000000000011 | 0.000000000037 |
| ENSG00000166225 | 3.263894 | 9.79388 | -1.58529 | 0.000000000012 | 0.000000000040 |
| ENSG00000261159 | 8.547888 | 4.089236 | 1.063737 | 0.000000000012 | 0.000000000042 |
| ENSG00000168803 | 3.461586 | 7.205994 | -1.05776 | 0.000000000013 | 0.000000000043 |
| ENSG00000237595 | 2.404416 | 6.187731 | -1.36372 | 0.000000000013 | 0.000000000044 |
| ENSG00000136021 | 2.990993 | 6.821777 | -1.18952 | 0.000000000013 | 0.000000000045 |
| ENSG00000203705 | 4.777562 | 9.934219 | -1.05613 | 0.000000000015 | 0.000000000052 |
| ENSG00000269743 | 10.94614 | 5.100233 | 1.101787 | 0.000000000016 | 0.000000000055 |
| ENSG00000232702 | 1.73721 | 6.462315 | -1.89528 | 0.000000000017 | 0.000000000057 |
| ENSG00000122691 | 6.205822 | 2.67739 | 1.212795 | 0.000000000025 | 0.000000000086 |
| ENSG00000276170 | 2.193226 | 6.85889 | -1.64492 | 0.000000000027 | 0.000000000089 |
| ENSG00000124140 | 8.565495 | 2.92399 | 1.550598 | 0.000000000030 | 0.000000000102 |
| ENSG00000212232 | 24.90842 | 11.28802 | 1.141841 | 0.000000000035 | 0.000000000116 |
| ENSG00000119685 | 34.49461 | 14.115 | 1.289142 | 0.000000000036 | 0.000000000122 |
| ENSG00000278922 | 5.211654 | 2.561282 | 1.024875 | 0.000000000037 | 0.000000000125 |
| ENSG00000164134 | 4.232402 | 8.941062 | -1.07897 | 0.000000000039 | 0.000000000131 |
| ENSG00000228705 | 1.905127 | 6.16747 | -1.69479 | 0.000000000046 | 0.000000000153 |
| ENSG00000160678 | 7.097718 | 3.124248 | 1.183846 | 0.000000000048 | 0.000000000161 |
| ENSG00000188338 | 5.799308 | 2.686302 | 1.110259 | 0.000000000060 | 0.000000000200 |
| ENSG00000156795 | 4.449121 | 9.950555 | -1.16126 | 0.000000000063 | 0.000000000207 |
| ENSG00000267697 | 0.020741 | 6.378196 | -8.26452 | 0.000000000069 | 0.000000000228 |
| ENSG00000201129 | 0.023334 | 8.343835 | -8.48213 | 0.000000000069 | 0.000000000228 |
| ENSG00000268129 | 2.786731 | 7.433354 | -1.41544 | 0.000000000107 | 0.000000000353 |
| ENSG00000014824 | 25.10329 | 10.79847 | 1.21705 | 0.000000000110 | 0.000000000360 |
| ENSG00000198723 | 6.869516 | 2.857269 | 1.265572 | 0.000000000110 | 0.000000000360 |
| ENSG00000162576 | 9.411744 | 3.649471 | 1.366775 | 0.000000000144 | 0.000000000471 |
| ENSG00000135346 | 2.337208 | 5.232869 | -1.16282 | 0.000000000151 | 0.000000000491 |
| ENSG00000240970 | 6.375066 | 1.825468 | 1.804174 | 0.000000000166 | 0.000000000538 |
| ENSG00000235241 | 9.300518 | 3.886057 | 1.259004 | 0.000000000168 | 0.000000000544 |
| ENSG00000012048 | 36.75821 | 17.60573 | 1.062021 | 0.000000000171 | 0.000000000553 |
| ENSG00000198417 | 4.735616 | 9.482778 | -1.00176 | 0.000000000189 | 0.000000000613 |
| ENSG00000234949 | 5.310329 | 2.654365 | 1.000434 | 0.000000000213 | 0.000000000689 |
| ENSG00000273143 | 2.563335 | 8.717273 | -1.76585 | 0.000000000254 | 0.000000000818 |
| ENSG00000263968 | 5.509166 | 0.771329 | 2.836416 | 0.000000000263 | 0.000000000848 |
| ENSG00000269688 | 11.89087 | 5.647666 | 1.074128 | 0.000000000276 | 0.000000000886 |
| ENSG00000225205 | 6.436897 | 2.646131 | 1.282481 | 0.000000000330 | 0.000000001057 |
| ENSG00000168538 | 4.091232 | 8.823683 | -1.10885 | 0.000000000357 | 0.000000001142 |
| ENSG00000105672 | 5.538624 | 1.923832 | 1.525545 | 0.000000000374 | 0.000000001195 |
| ENSG00000236603 | 2.398526 | 6.228969 | -1.37685 | 0.000000000390 | 0.000000001243 |
| ENSG00000269970 | 7.320408 | 0.951779 | 2.943226 | 0.000000000391 | 0.000000001248 |
| ENSG00000255072 | 6.332687 | 0.516307 | 3.616517 | 0.000000000401 | 0.000000001278 |
| ENSG00000224126 | 3.851938 | 9.274365 | -1.26766 | 0.000000000416 | 0.000000001326 |
| ENSG00000229980 | 5.564506 | 2.717882 | 1.033771 | 0.000000000429 | 0.000000001365 |
| ENSG00000124783 | 139.1714 | 24.5174 | 2.504986 | 0.000000000437 | 0.000000001391 |
| ENSG00000236269 | 6.248073 | 2.656255 | 1.234018 | 0.000000000457 | 0.000000001451 |
| ENSG00000223975 | 16.04845 | 6.674026 | 1.265805 | 0.000000000540 | 0.000000001710 |
| ENSG00000260233 | 11.9776 | 3.913547 | 1.61379 | 0.000000000546 | 0.000000001726 |
| ENSG00000249456 | 3.508772 | 8.332703 | -1.24782 | 0.000000000891 | 0.000000002792 |
| ENSG00000259031 | 6.759028 | 2.597243 | 1.379835 | 0.000000000970 | 0.000000003033 |
| ENSG00000267265 | 7.397519 | 2.61572 | 1.499833 | 0.000000001012 | 0.000000003161 |
| ENSG00000261064 | 9.467983 | 3.448414 | 1.457124 | 0.000000001012 | 0.000000003161 |
| ENSG00000071189 | 3.66221 | 8.104114 | -1.14594 | 0.000000001355 | 0.000000004204 |
| ENSG00000251611 | 13.73385 | 6.136949 | 1.162143 | 0.000000001928 | 0.000000005946 |
| ENSG00000108852 | 22.86429 | 11.03945 | 1.050428 | 0.000000002356 | 0.000000007240 |
| ENSG00000161958 | 5.501778 | 2.561746 | 1.10277 | 0.000000002488 | 0.000000007637 |
| ENSG00000239257 | 2.728893 | 7.335674 | -1.42661 | 0.000000003263 | 0.000000009961 |
| ENSG00000153922 | 3.205787 | 9.863905 | -1.62148 | 0.000000003889 | 0.000000011830 |
| ENSG00000277694 | 0.0001 | 6.49014 | -15.986 | 0.000000004074 | 0.000000012377 |
| ENSG00000201998 | 10.45086 | 2.237525 | 2.223646 | 0.000000004854 | 0.000000014678 |
| ENSG00000262766 | 6.93791 | 2.802942 | 1.307559 | 0.000000005518 | 0.000000016651 |
| ENSG00000131724 | 62.34191 | 27.63283 | 1.173819 | 0.000000005632 | 0.000000016989 |
| ENSG00000143756 | 16.70211 | 8.049061 | 1.053138 | 0.000000006045 | 0.000000018211 |
| ENSG00000269242 | 5.72386 | 2.636641 | 1.118287 | 0.000000006163 | 0.000000018560 |
| ENSG00000105855 | 8.751924 | 4.340748 | 1.011657 | 0.000000009310 | 0.000000027814 |
| ENSG00000092978 | 2.287977 | 5.098438 | -1.15598 | 0.000000009864 | 0.000000029445 |
| ENSG00000254614 | 10.82544 | 4.236138 | 1.353604 | 0.000000012740 | 0.000000037785 |
| ENSG00000237015 | 6.240534 | 1.875193 | 1.73463 | 0.000000012781 | 0.000000037904 |
| ENSG00000221164 | 5.023551 | 17.6242 | -1.81078 | 0.000000013173 | 0.000000039045 |
| ENSG00000270038 | 2.990084 | 6.497783 | -1.11976 | 0.000000013931 | 0.000000041243 |
| ENSG00000213904 | 19.1787 | 9.346387 | 1.037024 | 0.000000014322 | 0.000000042372 |
| ENSG00000267030 | 5.958127 | 2.242472 | 1.409769 | 0.000000017486 | 0.000000051486 |
| ENSG00000247092 | 4.613089 | 9.875565 | -1.09813 | 0.000000018984 | 0.000000055772 |
| ENSG00000271762 | 5.208163 | 1.835805 | 1.504362 | 0.000000026421 | 0.000000077064 |
| ENSG00000141338 | 6.452172 | 2.269588 | 1.507354 | 0.000000026840 | 0.000000078260 |
| ENSG00000115084 | 6.101183 | 13.16029 | -1.10903 | 0.000000030531 | 0.000000088750 |
| ENSG00000175595 | 10.01796 | 3.024197 | 1.727964 | 0.000000036999 | 0.000000106978 |
| ENSG00000264346 | 6.995629 | 0.119224 | 5.874707 | 0.000000037978 | 0.000000109795 |
| ENSG00000263776 | 0.004529 | 5.672505 | -10.2906 | 0.000000060455 | 0.000000173017 |
| ENSG00000277887 | 19.80793 | 7.005337 | 1.499552 | 0.000000063896 | 0.000000182559 |
| ENSG00000200624 | 13.06298 | 2.566618 | 2.347544 | 0.000000065985 | 0.000000188268 |
| ENSG00000269888 | 18.2322 | 8.70497 | 1.066578 | 0.000000066637 | 0.000000190106 |
| ENSG00000238917 | 25.15868 | 10.71193 | 1.231838 | 0.000000069690 | 0.000000198679 |
| ENSG00000164975 | 15.05513 | 7.096884 | 1.084997 | 0.000000073533 | 0.000000209467 |
| ENSG00000278527 | 13.67035 | 30.50779 | -1.15813 | 0.000000119649 | 0.000000337239 |
| ENSG00000253203 | 13.09689 | 6.390821 | 1.035151 | 0.000000125831 | 0.000000354021 |
| ENSG00000198464 | 9.705451 | 3.790463 | 1.356421 | 0.000000128972 | 0.000000362693 |
| ENSG00000207304 | 0.0001 | 5.285063 | -15.6896 | 0.000000148645 | 0.000000416723 |
| ENSG00000253616 | 2.434432 | 6.265612 | -1.36387 | 0.000000155424 | 0.000000435162 |
| ENSG00000100528 | 96.72398 | 45.46817 | 1.089017 | 0.000000244903 | 0.000000679186 |
| ENSG00000092098 | 56.03473 | 26.602 | 1.074786 | 0.000000280080 | 0.000000773894 |
| ENSG00000101624 | 3.639144 | 7.73885 | -1.08852 | 0.000000303535 | 0.000000836750 |
| ENSG00000100344 | 5.375512 | 2.62757 | 1.032673 | 0.000000320619 | 0.000000882767 |
| ENSG00000174500 | 1.721976 | 5.622062 | -1.70703 | 0.000000353551 | 0.000000970752 |
| ENSG00000210195 | 0.211706 | 10.59834 | -5.64563 | 0.000000447406 | 0.000001219094 |
| ENSG00000201823 | 31.4461 | 9.800355 | 1.681975 | 0.000000462556 | 0.000001259338 |
| ENSG00000266953 | 2.405388 | 5.29764 | -1.13908 | 0.000000541384 | 0.000001468482 |
| ENSG00000278708 | 6.987806 | 2.725551 | 1.358292 | 0.000000547423 | 0.000001484296 |
| ENSG00000277918 | 6.931133 | 1.024003 | 2.758871 | 0.000000550104 | 0.000001490754 |
| ENSG00000255248 | 1.898941 | 5.957694 | -1.64956 | 0.000000674313 | 0.000001816954 |
| ENSG00000165355 | 10.37343 | 4.873113 | 1.089977 | 0.000000791078 | 0.000002123868 |
| ENSG00000210077 | 14.63174 | 37.75095 | -1.36741 | 0.000000879737 | 0.000002356932 |
| ENSG00000080947 | 8.443388 | 3.915789 | 1.108519 | 0.000001073070 | 0.000002857343 |
| ENSG00000134152 | 5.558388 | 12.12765 | -1.12556 | 0.000001509503 | 0.000003984043 |
| ENSG00000166575 | 5.767218 | 2.129299 | 1.437497 | 0.000001562216 | 0.000004115740 |
| ENSG00000087299 | 10.87128 | 3.915025 | 1.473429 | 0.000001663888 | 0.000004376872 |
| ENSG00000257261 | 3.474084 | 7.389573 | -1.08886 | 0.000001743605 | 0.000004580025 |
| ENSG00000238795 | 8.838378 | 3.385566 | 1.384385 | 0.000001799390 | 0.000004721073 |
| ENSG00000260793 | 15.56197 | 7.030384 | 1.146349 | 0.000001821719 | 0.000004777639 |
| ENSG00000182628 | 8.003592 | 16.12203 | -1.01031 | 0.000002077361 | 0.000005430897 |
| ENSG00000265660 | 29.3151 | 10.01967 | 1.548809 | 0.000002098222 | 0.000005482551 |
| ENSG00000128000 | 5.052729 | 2.007614 | 1.331581 | 0.000002486792 | 0.000006445691 |
| ENSG00000164825 | 6.473327 | 2.994544 | 1.112171 | 0.000003185750 | 0.000008205180 |
| ENSG00000135931 | 9.332662 | 26.42094 | -1.50132 | 0.000003939396 | 0.000010080457 |
| ENSG00000223482 | 11.76728 | 24.93485 | -1.08338 | 0.000003940906 | 0.000010083283 |
| ENSG00000267458 | 2.167101 | 6.535428 | -1.59252 | 0.000004301689 | 0.000010986036 |
| ENSG00000166762 | 7.911315 | 3.87464 | 1.029855 | 0.000004374741 | 0.000011163427 |
| ENSG00000228106 | 3.974461 | 8.261517 | -1.05565 | 0.000004410824 | 0.000011254349 |
| ENSG00000206760 | 8.305932 | 18.7002 | -1.17084 | 0.000004478168 | 0.000011420316 |
| ENSG00000153936 | 4.931285 | 11.09848 | -1.17033 | 0.000005149284 | 0.000013078140 |
| ENSG00000230330 | 4.086412 | 9.646509 | -1.23917 | 0.000005160487 | 0.000013105255 |
| ENSG00000015153 | 5.457204 | 11.85952 | -1.11981 | 0.000005407564 | 0.000013713101 |
| ENSG00000198001 | 3.722098 | 7.820041 | -1.07106 | 0.000005533441 | 0.000014025160 |
| ENSG00000269481 | 5.004972 | 2.471682 | 1.017869 | 0.000005715372 | 0.000014474478 |
| ENSG00000137845 | 49.57319 | 16.04929 | 1.62705 | 0.000005971423 | 0.000015092188 |
| ENSG00000113851 | 44.74047 | 14.02758 | 1.673315 | 0.000007712770 | 0.000019343792 |
| ENSG00000231587 | 21.98951 | 7.795187 | 1.49616 | 0.000008932148 | 0.000022299643 |
| ENSG00000114670 | 3.332449 | 7.271115 | -1.12559 | 0.000013219353 | 0.000032546895 |
| ENSG00000279381 | 5.653378 | 1.337504 | 2.07957 | 0.000014625594 | 0.000035881206 |
| ENSG00000264201 | 13.45947 | 1.900375 | 2.824265 | 0.000015932502 | 0.000038966320 |
| ENSG00000168300 | 1.96202 | 5.787427 | -1.56058 | 0.000017419158 | 0.000042429007 |
| ENSG00000257921 | 2.064015 | 6.441549 | -1.64195 | 0.000022334932 | 0.000053958539 |
| ENSG00000203791 | 5.314453 | 19.32003 | -1.8621 | 0.000025996186 | 0.000062542315 |
| ENSG00000210100 | 0.182649 | 7.276021 | -5.316 | 0.000027212130 | 0.000065341181 |
| ENSG00000232653 | 5.616974 | 2.616169 | 1.102337 | 0.000027264149 | 0.000065447122 |
| ENSG00000277942 | 10.02136 | 24.78643 | -1.30647 | 0.000027905491 | 0.000066928483 |
| ENSG00000259256 | 3.063811 | 6.482448 | -1.08121 | 0.000030098347 | 0.000072024511 |
| ENSG00000230454 | 5.276635 | 2.506456 | 1.073969 | 0.000036074687 | 0.000085825852 |
| ENSG00000259248 | 3.242727 | 6.513769 | -1.00628 | 0.000038926422 | 0.000092398212 |
| ENSG00000264268 | 17.40359 | 5.206849 | 1.740902 | 0.000039085342 | 0.000092757718 |
| ENSG00000144161 | 3.845584 | 9.428356 | -1.2938 | 0.000045290273 | 0.000106779884 |
| ENSG00000210107 | 5.82573 | 18.80557 | -1.69065 | 0.000046415787 | 0.000109257313 |
| ENSG00000277775 | 6.559685 | 3.225204 | 1.024236 | 0.000049729291 | 0.000116824590 |
| ENSG00000207445 | 12.92927 | 5.316569 | 1.282074 | 0.000053359437 | 0.000124921641 |
| ENSG00000258788 | 5.924379 | 1.892041 | 1.646721 | 0.000053967517 | 0.000126256053 |
| ENSG00000151576 | 7.10865 | 15.14512 | -1.09121 | 0.000057211908 | 0.000133707823 |
| ENSG00000151553 | 13.58052 | 4.168333 | 1.703996 | 0.000061634941 | 0.000143760883 |
| ENSG00000284520 | 0.126094 | 5.362282 | -5.41028 | 0.000077854686 | 0.000179629083 |
| ENSG00000273874 | 34.03994 | 15.57576 | 1.127926 | 0.000095210427 | 0.000217702658 |
| ENSG00000276753 | 24.88526 | 10.82465 | 1.200972 | 0.000115408285 | 0.000261695288 |
| ENSG00000137710 | 53.90582 | 25.45235 | 1.082642 | 0.000137232339 | 0.000308063239 |
| ENSG00000236663 | 5.297026 | 2.581169 | 1.037158 | 0.000150519281 | 0.000336977193 |
| ENSG00000136111 | 8.645864 | 4.305758 | 1.005743 | 0.000159598778 | 0.000356533371 |
| ENSG00000266651 | 7.603651 | 3.664969 | 1.052891 | 0.000165184542 | 0.000368713377 |
| ENSG00000264553 | 12.727 | 3.88798 | 1.7108 | 0.000169582434 | 0.000378156504 |
| ENSG00000272482 | 6.227921 | 2.990933 | 1.058155 | 0.000171243776 | 0.000381792675 |
| ENSG00000163512 | 9.524545 | 30.06493 | -1.65836 | 0.000206325006 | 0.000456323576 |
| ENSG00000234287 | 2.287558 | 5.774414 | -1.33587 | 0.000256733041 | 0.000562702866 |
| ENSG00000213658 | 5.928062 | 2.947719 | 1.007962 | 0.000284057211 | 0.000620403312 |
| ENSG00000210151 | 0.673281 | 6.619739 | -3.29749 | 0.000305442912 | 0.000664920477 |
| ENSG00000103034 | 5.038087 | 1.427966 | 1.818914 | 0.000333319440 | 0.000721340877 |
| ENSG00000121486 | 4.630599 | 9.999795 | -1.1107 | 0.000336773491 | 0.000728435318 |
| ENSG00000128908 | 13.85528 | 35.71541 | -1.36611 | 0.000479336860 | 0.001021508602 |
| ENSG00000264293 | 6.231687 | 3.051948 | 1.029892 | 0.000633085200 | 0.001332875857 |
| ENSG00000202503 | 21.52675 | 9.826547 | 1.131374 | 0.000820764026 | 0.001702059100 |
| ENSG00000073670 | 5.419034 | 2.449313 | 1.145659 | 0.000887851213 | 0.001835286287 |
| ENSG00000163626 | 5.774783 | 1.634132 | 1.821242 | 0.000968614276 | 0.001995263534 |
| ENSG00000221539 | 2.356538 | 8.943069 | -1.9241 | 0.001072011491 | 0.002190283546 |
| ENSG00000274111 | 19.48217 | 8.077786 | 1.270123 | 0.001123473393 | 0.002291284718 |
| ENSG00000253540 | 5.212496 | 2.489973 | 1.065844 | 0.001170046378 | 0.002381286511 |
| ENSG00000227758 | 5.568956 | 2.407701 | 1.209751 | 0.001458087601 | 0.002934473604 |
| ENSG00000233382 | 5.038984 | 1.89615 | 1.41006 | 0.001675619480 | 0.003351913199 |
| ENSG00000281420 | 1.117307 | 5.441983 | -2.28411 | 0.001743518428 | 0.003480176468 |
| ENSG00000226744 | 1.668339 | 5.307785 | -1.6697 | 0.002009466948 | 0.003968010798 |
| ENSG00000102158 | 84.28086 | 29.49054 | 1.514953 | 0.002403889218 | 0.004699077878 |
| ENSG00000145246 | 10.74877 | 5.001792 | 1.103655 | 0.002431334645 | 0.004750859623 |
| ENSG00000202521 | 5.52638 | 1.331807 | 2.05295 | 0.002530101942 | 0.004934348906 |
| ENSG00000277678 | 10.63158 | 5.312039 | 1.001018 | 0.002583756360 | 0.005035433128 |
| ENSG00000203778 | 2.780535 | 7.553799 | -1.44184 | 0.003005159549 | 0.005815886866 |
| ENSG00000266392 | 5.559911 | 0.45672 | 3.60568 | 0.003070220771 | 0.005914172173 |
| ENSG00000164070 | 4.277267 | 13.85724 | -1.69588 | 0.003086114346 | 0.005942945794 |
| ENSG00000033867 | 1.83652 | 7.523144 | -2.03436 | 0.003186032901 | 0.006127289735 |
| ENSG00000182841 | 22.49838 | 50.12208 | -1.15563 | 0.003264091071 | 0.006270132283 |
| ENSG00000207650 | 5.404611 | 12.13466 | -1.16687 | 0.003274655426 | 0.006289696706 |
| ENSG00000086300 | 5.538368 | 2.435292 | 1.185366 | 0.003301147321 | 0.006338865892 |
| ENSG00000276161 | 8.452236 | 3.786294 | 1.158547 | 0.003506633483 | 0.006717354229 |
| ENSG00000275022 | 6.757442 | 2.987646 | 1.177468 | 0.003506633483 | 0.006717354229 |
| ENSG00000284258 | 20.41654 | 9.956633 | 1.036009 | 0.003933018510 | 0.007489985910 |
| ENSG00000252835 | 8.434031 | 3.904497 | 1.111086 | 0.004013195591 | 0.007637407864 |
| ENSG00000153896 | 10.19922 | 4.255197 | 1.261161 | 0.004560070196 | 0.008634849155 |
| ENSG00000282206 | 5.737616 | 1.895341 | 1.597994 | 0.004895707667 | 0.009214250433 |
| ENSG00000122435 | 10.99151 | 2.779017 | 1.983743 | 0.005549186541 | 0.010367240125 |
| ENSG00000252408 | 0.874462 | 5.291652 | -2.59725 | 0.005669670470 | 0.010577625914 |
| ENSG00000266079 | 6.516521 | 2.995049 | 1.121522 | 0.005814474166 | 0.010828679430 |
| ENSG00000276926 | 3.345704 | 9.220581 | -1.46255 | 0.006051420542 | 0.011233349048 |
| ENSG00000143995 | 5.273569 | 2.463897 | 1.097838 | 0.006553092174 | 0.012089224594 |
| ENSG00000115750 | 15.45805 | 5.764458 | 1.423101 | 0.008413572662 | 0.015257618418 |
| ENSG00000158411 | 11.02794 | 27.58002 | -1.32246 | 0.009052485522 | 0.016360159417 |
| ENSG00000169967 | 27.30915 | 10.75265 | 1.344693 | 0.011143746867 | 0.019922332844 |
| ENSG00000208772 | 7.339275 | 3.362806 | 1.125972 | 0.012087380913 | 0.021433587341 |
| ENSG00000202566 | 7.574546 | 2.389025 | 1.664737 | 0.012446673949 | 0.022011808498 |
| ENSG00000198791 | 51.06093 | 7.608206 | 2.746592 | 0.013404763314 | 0.023624659792 |
| ENSG00000283971 | 2.304367 | 7.063763 | -1.61607 | 0.014948233552 | 0.026196294515 |
| ENSG00000066427 | 1.572595 | 6.08107 | -1.95118 | 0.015607815633 | 0.027256110242 |
| ENSG00000166974 | 8.39322 | 18.29667 | -1.12428 | 0.016336202983 | 0.028428241218 |
| ENSG00000207757 | 5.371582 | 1.608166 | 1.739931 | 0.024470240090 | 0.041065133535 |
| ENSG00000116406 | 8.610431 | 3.819299 | 1.172778 | 0.026693805758 | 0.044478343594 |

**Sup.Tab.4** RNA-seq analysis of the gene expression profile affected by both sh-1 and sh-2 treatment for SNX9.

| Gene id |  | sh-NC | sh-1 | sh-2 |
| --- | --- | --- | --- | --- |
| ENSG00000124208 | | 7.731574 | 3.841499 | 2.639428 |
| ENSG00000113739 | | 94.89493 | 19.48043 | 42.78589 |
| ENSG00000171401 | | 52.83612 | 11.01056 | 18.40001 |
| ENSG00000280407 | | 2.744271 | 6.695593 | 6.567105 |
| ENSG00000198417 | | 9.482778 | 3.039379 | 4.181661 |
| ENSG00000103187 | | 346.7943 | 130.9991 | 130.4493 |
| ENSG00000163083 | | 6.149646 | 1.280648 | 2.980033 |
| ENSG00000059804 | | 7.904806 | 0.46031 | 2.780843 |
| ENSG00000092020 | | 2.168191 | 6.335446 | 8.52069 |
| ENSG00000183291 | | 20.74058 | 53.87877 | 99.37027 |
| ENSG00000205765 | | 4.150264 | 10.85925 | 18.97415 |
| ENSG00000147687 | | 5.191864 | 11.58603 | 22.61466 |
| ENSG00000272335 | | 2.170429 | 5.092083 | 7.920755 |
| ENSG00000132823 | | 25.43351 | 58.34975 | 70.08611 |
| ENSG00000200624 | | 2.566618 | 16.78407 | 14.23249 |
| ENSG00000241288 | | 6.470905 | 1.474141 | 2.218654 |
| ENSG00000129422 | | 14.14674 | 28.99848 | 29.24689 |
| ENSG00000263740 | | 160.4339 | 72.37756 | 457.2966 |
| ENSG00000204758 | | 15.16679 | 2.612242 | 2.937448 |
| ENSG00000202521 | | 1.331807 | 7.555683 | 5.963887 |
| ENSG00000173391 | | 62.59703 | 10.69802 | 24.26185 |
| ENSG00000141338 | | 2.269588 | 6.424245 | 7.808337 |
| ENSG00000268621 | | 524.5468 | 115.659 | 115.0518 |
| ENSG00000114857 | | 12.89443 | 29.46929 | 33.81695 |
| ENSG00000213859 | | 97.06745 | 48.46461 | 42.6432 |
| ENSG00000114854 | | 90.19296 | 29.93264 | 44.76176 |
| ENSG00000129514 | | 3.289006 | 14.02201 | 11.71758 |
| ENSG00000258017 | | 1019.874 | 472.8357 | 509.7137 |
| ENSG00000198894 | | 3.373883 | 8.119675 | 6.8046 |
| ENSG00000198898 | | 8.312141 | 21.92592 | 27.22906 |
| ENSG00000113851 | | 14.02758 | 38.41774 | 47.99521 |
| ENSG00000108064 | | 1.186444 | 5.698326 | 11.62098 |
| ENSG00000101596 | | 6.042057 | 13.25571 | 22.80573 |
| ENSG00000183607 | | 10.92316 | 1.88114 | 0.991346 |
| ENSG00000185432 | | 0.93921 | 7.664958 | 6.734219 |
| ENSG00000155622 | | 11.20168 | 4.21902 | 2.914697 |
| ENSG00000101974 | | 1.642737 | 6.405258 | 5.377182 |
| ENSG00000125898 | | 16.10064 | 5.791785 | 5.157794 |
| ENSG00000206596 | | 5.314462 | 16.82542 | 93.16036 |
| ENSG00000270136 | | 0.696356 | 46.53352 | 52.06733 |
| ENSG00000238266 | | 15.40596 | 6.769897 | 6.910795 |
| ENSG00000128342 | | 47.33786 | 18.04887 | 22.90603 |
| ENSG00000170298 | | 40.41373 | 5.633287 | 19.4488 |
| ENSG00000275560 | | 2.676549 | 6.136312 | 8.81102 |
| ENSG00000197006 | | 12.86291 | 36.54185 | 32.74445 |
| ENSG00000210154 | | 4.608305 | 15.09233 | 16.89028 |
| ENSG00000198796 | | 21.46776 | 6.104886 | 10.67855 |
| ENSG00000265660 | | 10.01967 | 26.14595 | 25.88719 |
| ENSG00000097021 | | 127.3666 | 59.31126 | 58.21545 |
| ENSG00000198668 | | 21.7092 | 52.7314 | 50.0112 |
| ENSG00000111348 | | 5.592893 | 1.878029 | 1.459059 |
| ENSG00000168685 | | 7.602631 | 1.183603 | 0.471971 |
| ENSG00000276496 | | 0.337197 | 17.47441 | 19.13431 |
| ENSG00000128165 | | 14.29593 | 2.108003 | 3.862691 |
| ENSG00000198406 | | 34.56565 | 103.7862 | 102.7352 |
| ENSG00000197172 | | 0.198212 | 31.35389 | 28.90693 |
| ENSG00000082497 | | 16.71874 | 3.182686 | 3.415405 |
| ENSG00000237864 | | 5.096415 | 1.648954 | 2.033206 |
| ENSG00000135919 | | 90.4394 | 23.30874 | 21.23372 |
| ENSG00000118523 | | 7.105779 | 0.50747 | 0.591446 |
| ENSG00000237595 | | 6.187731 | 2.121899 | 3.091139 |
| ENSG00000156162 | | 3.831795 | 9.254557 | 11.60821 |
| ENSG00000214922 | | 19.59513 | 6.044002 | 7.264624 |
| ENSG00000279117 | | 2.141529 | 5.094016 | 5.466434 |
| ENSG00000244021 | | 14.51081 | 32.01985 | 33.19692 |
| ENSG00000210135 | | 944.7426 | 2968.349 | 2274.613 |
| ENSG00000105281 | | 397.7608 | 132.6085 | 170.4887 |
| ENSG00000177606 | | 56.57069 | 22.96075 | 21.21251 |
| ENSG00000136244 | | 205.0733 | 14.81992 | 6.473201 |
| ENSG00000115009 | | 15.88397 | 1.45277 | 2.497792 |
| ENSG00000149798 | | 55.73309 | 17.03354 | 19.03885 |
| ENSG00000165685 | | 10.35095 | 1.097797 | 1.614868 |
| ENSG00000266711 | | 16.48018 | 3.210518 | 1.232674 |
| ENSG00000249641 | | 5.748573 | 2.602528 | 2.25208 |
| ENSG00000115464 | | 11.22018 | 33.15097 | 23.96642 |
| ENSG00000116161 | | 33.25993 | 67.98969 | 111.5394 |
| ENSG00000168994 | | 81.28843 | 21.3035 | 31.71992 |
| ENSG00000166669 | | 2.16557 | 5.709823 | 7.502345 |
| ENSG00000125954 | | 8.950398 | 0 | 0.106419 |
| ENSG00000267530 | | 39.68639 | 15.32524 | 19.6747 |
| ENSG00000100342 | | 37.09788 | 5.880852 | 12.57594 |
| ENSG00000100345 | | 496.3726 | 207.3561 | 216.7324 |
| ENSG00000159176 | | 439.1967 | 86.09644 | 137.2004 |
| ENSG00000228223 | | 5.165976 | 12.12265 | 17.32176 |
| ENSG00000278334 | | 44.19896 | 0.030694 | 0.033336 |
| ENSG00000117983 | | 1.84788 | 8.766535 | 5.795876 |
| ENSG00000277957 | | 12.79078 | 37.20763 | 49.33246 |
| ENSG00000239470 | | 1.79949 | 12.50266 | 13.07885 |
| ENSG00000106714 | | 3.171883 | 7.067959 | 12.82833 |
| ENSG00000246273 | | 16.84321 | 5.04835 | 4.583205 |
| ENSG00000171462 | | 14.30094 | 5.590249 | 6.953362 |
| ENSG00000102100 | | 90.2485 | 36.69828 | 42.91241 |
| ENSG00000220842 | | 41.38263 | 144.6617 | 135.2935 |
| ENSG00000105854 | | 83.8883 | 173.7657 | 193.0194 |
| ENSG00000131263 | | 9.901253 | 20.58422 | 33.00008 |
| ENSG00000283515 | | 9.956838 | 4.311295 | 1.493673 |
| ENSG00000154734 | | 19.09107 | 8.00856 | 5.780115 |
| ENSG00000149311 | | 8.092015 | 21.54603 | 38.13423 |
| ENSG00000105929 | | 5.485311 | 1.321568 | 0.822925 |
| ENSG00000226278 | | 1.609653 | 5.94444 | 11.75673 |
| ENSG00000027869 | | 49.5309 | 22.07924 | 19.25669 |
| ENSG00000267265 | | 2.61572 | 6.222302 | 7.111884 |
| ENSG00000204778 | | 1.853659 | 6.390436 | 7.395686 |
| ENSG00000210127 | | 406.3652 | 1991.51 | 1218.971 |
| ENSG00000148841 | | 94.09417 | 32.61293 | 31.97259 |
| ENSG00000047457 | | 1.898502 | 11.45414 | 5.562007 |
| ENSG00000187017 | | 3.391159 | 9.379729 | 9.858681 |
| ENSG00000172667 | | 7.112667 | 14.76769 | 15.2082 |
| ENSG00000206737 | | 7.031068 | 24.62433 | 158.1762 |
| ENSG00000224389 | | 13.85739 | 39.85053 | 42.81624 |
| ENSG00000179862 | | 226.4734 | 91.52481 | 98.43102 |
| ENSG00000128710 | | 2.724718 | 6.048402 | 6.830439 |
| ENSG00000166473 | | 6.027668 | 0.798092 | 1.801549 |
| ENSG00000139926 | | 9.535909 | 3.077285 | 3.09083 |
| ENSG00000152056 | | 1.943735 | 6.657026 | 7.55918 |
| ENSG00000227329 | | 0.275277 | 8.111075 | 12.45175 |
| ENSG00000148426 | | 59.82249 | 25.00202 | 14.10653 |
| ENSG00000121774 | | 221.4473 | 93.1381 | 102.5331 |
| ENSG00000110619 | | 69.9801 | 27.12905 | 26.51827 |
| ENSG00000122958 | | 5.28142 | 11.70154 | 25.68518 |
| ENSG00000137414 | | 6.190029 | 13.1132 | 18.84884 |
| ENSG00000164236 | | 5.770862 | 1.792662 | 2.498476 |
| ENSG00000185561 | | 2.132923 | 5.530388 | 6.384367 |
| ENSG00000185567 | | 6.78913 | 13.59762 | 19.49419 |
| ENSG00000270757 | | 1.947285 | 12.05216 | 6.17555 |
| ENSG00000155287 | | 24.07488 | 54.04807 | 59.34583 |
| ENSG00000234160 | | 22.21716 | 10.46021 | 9.295856 |
| ENSG00000171658 | | 10.49249 | 3.322799 | 3.81763 |
| ENSG00000152256 | | 17.14849 | 57.76468 | 68.79279 |
| ENSG00000082258 | | 2.833947 | 7.449181 | 6.893419 |
| ENSG00000005469 | | 2.249084 | 5.56431 | 5.032659 |
| ENSG00000183801 | | 20.74291 | 5.045249 | 7.205454 |
| ENSG00000103066 | | 27.22361 | 12.16975 | 11.66694 |
| ENSG00000276965 | | 10.35382 | 4.198428 | 0.972061 |
| ENSG00000100867 | | 8.322936 | 2.931396 | 1.618536 |
| ENSG00000166004 | | 4.970973 | 10.5609 | 19.98192 |
| ENSG00000234498 | | 25.91884 | 7.608696 | 4.753818 |
| ENSG00000169359 | | 4.443292 | 18.52364 | 12.54178 |
| ENSG00000139269 | | 13.89998 | 2.320173 | 5.469463 |
| ENSG00000280071 | | 24.44698 | 78.76458 | 65.58073 |
| ENSG00000135931 | | 26.42094 | 8.402138 | 6.122962 |
| ENSG00000207389 | | 5.092112 | 18.2761 | 118.4086 |
| ENSG00000138801 | | 29.19992 | 75.5626 | 80.24069 |
| ENSG00000214944 | | 7.129524 | 2.24694 | 2.913418 |
| ENSG00000128965 | | 12.50558 | 2.00728 | 1.658815 |
| ENSG00000071189 | | 8.104114 | 21.39494 | 31.31561 |
| ENSG00000276170 | | 6.85889 | 1.769629 | 2.085195 |
| ENSG00000281490 | | 6.252997 | 13.86541 | 12.86047 |
| ENSG00000066279 | | 3.994333 | 9.942974 | 30.23282 |
| ENSG00000213860 | | 2.375226 | 12.3534 | 15.91497 |
| ENSG00000188112 | | 11.79637 | 3.415668 | 4.809777 |
| ENSG00000163545 | | 15.50222 | 4.298685 | 5.495624 |
| ENSG00000142910 | | 220.9791 | 35.23274 | 65.24152 |
| ENSG00000186847 | | 8.898859 | 3.130322 | 2.842629 |
| ENSG00000179387 | | 3.274977 | 6.708862 | 14.80268 |
| ENSG00000172650 | | 2.0352 | 11.20333 | 5.94311 |
| ENSG00000164048 | | 13.90266 | 4.002345 | 2.67526 |
| ENSG00000133328 | | 76.54984 | 37.19742 | 37.3883 |
| ENSG00000166689 | | 23.17032 | 7.859536 | 9.127255 |
| ENSG00000128708 | | 22.43377 | 48.49409 | 77.077 |
| ENSG00000182287 | | 1.539209 | 5.601254 | 9.387104 |
| ENSG00000165887 | | 23.67719 | 1.684479 | 3.240889 |
| ENSG00000172869 | | 6.527703 | 16.20083 | 13.19645 |
| ENSG00000122435 | | 2.779017 | 9.413114 | 9.028728 |
| ENSG00000173744 | | 76.87247 | 30.27902 | 20.77268 |
| ENSG00000267769 | | 2.767907 | 7.453463 | 6.301497 |
| ENSG00000130513 | | 217.8267 | 74.84061 | 91.37969 |
| ENSG00000101928 | | 6.289828 | 13.52042 | 15.46523 |
| ENSG00000113558 | | 104.6955 | 252.2648 | 230.2714 |
| ENSG00000088451 | | 2.498567 | 6.075656 | 9.930507 |
| ENSG00000165338 | | 1.608446 | 6.776588 | 6.406146 |
| ENSG00000100092 | | 51.76777 | 6.60346 | 6.445217 |
| ENSG00000197969 | | 2.627396 | 5.705082 | 8.626451 |
| ENSG00000204941 | | 11.20667 | 0.366014 | 1.179461 |
| ENSG00000261353 | | 0.483521 | 5.127094 | 7.070802 |
| ENSG00000117525 | | 142.2828 | 24.49643 | 42.22972 |
| ENSG00000175105 | | 2.453033 | 5.84533 | 12.72362 |
| ENSG00000212304 | | 0.122066 | 12.36983 | 8.156145 |
| ENSG00000078804 | | 0.504089 | 9.184402 | 6.414757 |
| ENSG00000123607 | | 7.40406 | 16.60119 | 18.79772 |
| ENSG00000220793 | | 21.3555 | 46.68312 | 58.84316 |
| ENSG00000198162 | | 3.604065 | 11.74001 | 8.630394 |
| ENSG00000198160 | | 6.313188 | 14.21801 | 14.87695 |
| ENSG00000168765 | | 45.43968 | 22.15633 | 20.1486 |
| ENSG00000006327 | | 342.2341 | 69.90039 | 94.09769 |
| ENSG00000108846 | | 182.5772 | 85.23998 | 88.70998 |
| ENSG00000041988 | | 44.37545 | 20.15984 | 21.36562 |
| ENSG00000269834 | | 5.153096 | 2.395724 | 1.738846 |
| ENSG00000240682 | | 49.97912 | 21.62542 | 13.21436 |
| ENSG00000115252 | | 7.610585 | 25.96231 | 18.92934 |
| ENSG00000115255 | | 14.39663 | 32.43572 | 31.75621 |
| ENSG00000149591 | | 33.9072 | 8.706709 | 10.37552 |
| ENSG00000118193 | | 2.448234 | 7.85074 | 11.99897 |
| ENSG00000133138 | | 2.748084 | 6.685957 | 10.61826 |
| ENSG00000188293 | | 7.930596 | 0.882219 | 1.640928 |
| ENSG00000146592 | | 6.429279 | 2.131747 | 1.178053 |
| ENSG00000278233 | | 36.0295 | 111.1666 | 330.6343 |
| ENSG00000138018 | | 1.42874 | 5.05156 | 8.748875 |
| ENSG00000052802 | | 2.588246 | 9.020772 | 17.67766 |
| ENSG00000137944 | | 4.243639 | 11.58225 | 13.13271 |
| ENSG00000136147 | | 5.126069 | 11.32931 | 10.41851 |
| ENSG00000254706 | | 10.11318 | 4.845965 | 3.006318 |
| ENSG00000268357 | | 2.79252 | 6.005813 | 9.232606 |
| ENSG00000104164 | | 5.436001 | 11.68952 | 17.11603 |
| ENSG00000116285 | | 229.5957 | 73.78388 | 49.83871 |
| ENSG00000203706 | | 36.39032 | 8.164653 | 6.326053 |
| ENSG00000104611 | | 47.81968 | 22.93129 | 20.73646 |
| ENSG00000140044 | | 13.32521 | 2.421064 | 2.237667 |
| ENSG00000145819 | | 8.338859 | 3.226203 | 3.905713 |
| ENSG00000253270 | | 27.07085 | 1.884361 | 0.471304 |
| ENSG00000072310 | | 121.9969 | 290.8403 | 329.0716 |
| ENSG00000103260 | | 20.88909 | 45.99155 | 42.66592 |
| ENSG00000183826 | | 3.422076 | 7.122818 | 6.889145 |
| ENSG00000124102 | | 30.39834 | 4.780984 | 8.364233 |
| ENSG00000136122 | | 2.501409 | 7.309694 | 10.20451 |
| ENSG00000087266 | | 38.49459 | 81.92847 | 143.9558 |
| ENSG00000134531 | | 73.00797 | 11.70248 | 11.27047 |
| ENSG00000214049 | | 59.95556 | 6.58123 | 16.62905 |
| ENSG00000189060 | | 394.5346 | 176.161 | 197.065 |
| ENSG00000230606 | | 3.238047 | 8.039764 | 11.40047 |
| ENSG00000109079 | | 135.3004 | 42.10978 | 61.68175 |
| ENSG00000135317 | | 5.039798 | 17.41166 | 21.47117 |
| ENSG00000135318 | | 17.59933 | 3.031011 | 5.199089 |
| ENSG00000162783 | | 36.70538 | 16.54786 | 16.22988 |
| ENSG00000166200 | | 2.271461 | 19.94892 | 21.19344 |
| ENSG00000057663 | | 4.349873 | 9.324044 | 10.58238 |
| ENSG00000226332 | | 23.95408 | 11.68959 | 11.04931 |
| ENSG00000210049 | | 52.64635 | 129.6104 | 161.5173 |
| ENSG00000188910 | | 22.19155 | 7.341332 | 7.697366 |
| ENSG00000105372 | | 1404.996 | 588.2961 | 459.6364 |
| ENSG00000015153 | | 11.85952 | 5.566939 | 5.92284 |
| ENSG00000180035 | | 16.54764 | 4.45434 | 8.269557 |
| ENSG00000115758 | | 107.1528 | 48.78614 | 42.94712 |
| ENSG00000101255 | | 142.0244 | 55.05401 | 58.08668 |
| ENSG00000137936 | | 79.52197 | 23.89459 | 26.74327 |
| ENSG00000148411 | | 4.267676 | 13.37043 | 13.30018 |
| ENSG00000232472 | | 2.493145 | 16.00592 | 11.85383 |
| ENSG00000072163 | | 30.2634 | 8.757208 | 11.09846 |
| ENSG00000099994 | | 11.04396 | 3.558857 | 3.492049 |
| ENSG00000120708 | | 137.9248 | 56.63454 | 66.28983 |
| ENSG00000159556 | | 11.34273 | 5.345253 | 4.027924 |
| ENSG00000101076 | | 2.313282 | 6.966496 | 8.292271 |
| ENSG00000161638 | | 237.4228 | 116.8113 | 100.9426 |
| ENSG00000104892 | | 13.78461 | 5.829432 | 6.242574 |
| ENSG00000257390 | | 15.12403 | 1.316835 | 0.576608 |
| ENSG00000175832 | | 45.8654 | 8.858068 | 21.51641 |
| ENSG00000183696 | | 65.20166 | 30.17527 | 32.27648 |
| ENSG00000087074 | | 132.249 | 31.89844 | 31.43079 |
| ENSG00000113597 | | 4.100664 | 9.612766 | 11.47978 |
| ENSG00000178075 | | 2.288896 | 5.484102 | 5.438064 |
| ENSG00000220804 | | 2.174572 | 6.064767 | 6.561383 |
| ENSG00000198146 | | 7.193478 | 14.52334 | 29.8213 |
| ENSG00000142583 | | 13.64367 | 5.578845 | 4.745521 |
| ENSG00000176697 | | 11.5529 | 5.406145 | 4.509173 |
| ENSG00000224126 | | 9.274365 | 4.17573 | 3.817697 |
| ENSG00000151229 | | 2.93948 | 10.19556 | 8.551999 |
| ENSG00000167106 | | 14.1629 | 34.65952 | 37.86168 |
| ENSG00000115365 | | 39.27602 | 79.52888 | 83.49139 |
| ENSG00000166986 | | 165.3721 | 73.54325 | 74.05254 |
| ENSG00000243207 | | 27.82485 | 4.659064 | 7.628769 |
| ENSG00000187908 | | 23.17676 | 8.037423 | 4.929212 |
| ENSG00000269814 | | 2.312912 | 5.117262 | 5.583334 |
| ENSG00000251259 | | 0 | 9.065315 | 5.943394 |
| ENSG00000162636 | | 2.910681 | 7.303399 | 7.41078 |
| ENSG00000250474 | | 6.391468 | 1.71604 | 2.948807 |
| ENSG00000162734 | | 151.1154 | 38.94631 | 60.70734 |
| ENSG00000063322 | | 44.09955 | 9.543899 | 14.5211 |
| ENSG00000214510 | | 63.07437 | 187.2939 | 135.8062 |
| ENSG00000162688 | | 3.303396 | 7.256157 | 9.57164 |
| ENSG00000103811 | | 3.078784 | 6.327306 | 7.818247 |
| ENSG00000162994 | | 3.249904 | 8.16992 | 13.44274 |
| ENSG00000172379 | | 6.222623 | 21.69067 | 14.84317 |
| ENSG00000067955 | | 13.57401 | 41.89469 | 34.24183 |
| ENSG00000116711 | | 9.008233 | 21.21326 | 25.94633 |
| ENSG00000116717 | | 59.26122 | 12.19781 | 10.66991 |
| ENSG00000113369 | | 15.02256 | 31.67639 | 41.36236 |
| ENSG00000205084 | | 18.10611 | 4.816947 | 8.267832 |
| ENSG00000165169 | | 9.511756 | 29.78462 | 30.31416 |
| ENSG00000196757 | | 1.846463 | 5.535344 | 8.418881 |
| ENSG00000159228 | | 241.036 | 117.6141 | 107.0047 |
| ENSG00000025800 | | 12.7371 | 30.44066 | 30.31365 |
| ENSG00000145901 | | 258.675 | 91.96287 | 117.4845 |
| ENSG00000198182 | | 1.827426 | 5.363544 | 6.018344 |
| ENSG00000182400 | | 1.820256 | 5.100638 | 9.905677 |
| ENSG00000176046 | | 73.80618 | 28.18925 | 27.66844 |
| ENSG00000181350 | | 10.88092 | 2.888177 | 3.825914 |
| ENSG00000207005 | | 10.73495 | 38.46573 | 235.9254 |
| ENSG00000111859 | | 59.88854 | 8.912534 | 16.24154 |
| ENSG00000151239 | | 13.22277 | 33.34925 | 57.10629 |
| ENSG00000128272 | | 627.3217 | 283.9095 | 289.7817 |
| ENSG00000167447 | | 5.694531 | 17.75515 | 13.5201 |
| ENSG00000210191 | | 96.94064 | 311.8642 | 218.4449 |
| ENSG00000145348 | | 2.967116 | 6.252496 | 8.948168 |
| ENSG00000210194 | | 90.62433 | 352.3842 | 306.7841 |
| ENSG00000146072 | | 17.59484 | 5.69143 | 4.094102 |
| ENSG00000118496 | | 2.387528 | 6.146449 | 5.143548 |
| ENSG00000115514 | | 6.199316 | 15.10033 | 16.58355 |
| ENSG00000074047 | | 5.827523 | 1.474928 | 2.309662 |
| ENSG00000267519 | | 45.52696 | 21.28296 | 20.06751 |
| ENSG00000277739 | | 36.36565 | 116.2276 | 388.1252 |
| ENSG00000163347 | | 64.4744 | 10.22227 | 20.83157 |
| ENSG00000278774 | | 4.189622 | 22.51253 | 140.697 |
| ENSG00000226281 | | 7.232504 | 2.904361 | 2.550572 |
| ENSG00000112773 | | 21.16555 | 44.92656 | 61.30106 |
| ENSG00000272414 | | 0.680415 | 6.204491 | 11.16022 |
| ENSG00000137269 | | 15.97227 | 5.744483 | 5.177703 |
| ENSG00000182795 | | 16.52634 | 6.147115 | 5.534313 |
| ENSG00000172780 | | 7.626609 | 19.37968 | 22.06038 |
| ENSG00000000971 | | 4.070459 | 17.56569 | 13.50883 |
| ENSG00000138592 | | 12.58499 | 26.04163 | 33.13529 |
| ENSG00000164975 | | 7.096884 | 17.82846 | 26.24042 |
| ENSG00000128510 | | 37.07925 | 3.110029 | 14.44668 |
| ENSG00000258289 | | 7.053098 | 20.38861 | 16.77663 |
| ENSG00000174327 | | 14.60335 | 3.459972 | 7.225224 |
| ENSG00000270038 | | 6.497783 | 3.006403 | 2.601994 |
| ENSG00000201998 | | 2.237525 | 6.10494 | 12.9981 |
| ENSG00000128463 | | 32.45008 | 80.65986 | 81.97265 |
| ENSG00000173846 | | 11.99293 | 5.229921 | 4.918054 |
| ENSG00000133816 | | 11.83 | 4.361292 | 4.561579 |
| ENSG00000085788 | | 11.02146 | 26.04264 | 25.76714 |
| ENSG00000156510 | | 44.79305 | 14.63301 | 21.38611 |
| ENSG00000218227 | | 13.77531 | 5.401725 | 3.164994 |
| ENSG00000160678 | | 3.124248 | 6.468075 | 7.364751 |
| ENSG00000041357 | | 48.90033 | 105.6023 | 163.3747 |
| ENSG00000117054 | | 14.06499 | 53.76198 | 53.06384 |
| ENSG00000198492 | | 12.32914 | 63.5898 | 39.41535 |
| ENSG00000179583 | | 1.005348 | 7.175155 | 6.172274 |
| ENSG00000176907 | | 18.08891 | 3.961195 | 1.94995 |
| ENSG00000273541 | | 0.116997 | 14.97232 | 37.90668 |
| ENSG00000155324 | | 21.2971 | 10.16409 | 8.615066 |
| ENSG00000171621 | | 12.52979 | 5.990388 | 4.697293 |
| ENSG00000205871 | | 1.896505 | 6.756793 | 7.030409 |
| ENSG00000171552 | | 85.23518 | 32.63746 | 25.8116 |
| ENSG00000202538 | | 0 | 42.25275 | 99.28567 |
| ENSG00000241975 | | 3.181439 | 7.69304 | 7.206728 |
| ENSG00000120875 | | 23.06345 | 2.525687 | 3.487817 |
| ENSG00000263934 | | 15.27275 | 33.61927 | 137.3119 |
| ENSG00000110203 | | 9.071077 | 1.749204 | 1.437542 |
| ENSG00000163006 | | 3.627654 | 8.061496 | 8.939875 |
| ENSG00000075651 | | 9.866661 | 23.96515 | 23.82915 |
| ENSG00000078401 | | 16.50404 | 4.702872 | 3.949281 |
| ENSG00000228705 | | 6.16747 | 2.359114 | 2.303344 |
| ENSG00000069424 | | 58.10721 | 27.48286 | 28.77154 |
| ENSG00000266401 | | 5.69095 | 1.515603 | 1.999195 |
| ENSG00000114107 | | 5.990731 | 12.9624 | 28.00453 |
| ENSG00000118804 | | 9.910404 | 4.064575 | 2.500111 |
| ENSG00000071794 | | 6.829925 | 19.75002 | 38.16653 |
| ENSG00000226564 | | 6.682032 | 3.067953 | 1.892355 |
| ENSG00000205403 | | 1.149027 | 11.69289 | 14.56756 |
| ENSG00000114744 | | 10.19179 | 22.48841 | 24.55928 |
| ENSG00000255860 | | 24.16694 | 1.649386 | 1.267934 |
| ENSG00000281991 | | 6.416695 | 2.155802 | 1.275097 |
| ENSG00000272601 | | 0.587227 | 8.429969 | 14.32151 |
| ENSG00000270820 | | 9.106625 | 3.970258 | 4.021873 |
| ENSG00000154265 | | 3.224083 | 6.67147 | 17.94971 |
| ENSG00000137693 | | 1288.154 | 54.06374 | 147.0798 |
| ENSG00000173221 | | 150.186 | 65.03477 | 54.59107 |
| ENSG00000173559 | | 22.29068 | 54.74773 | 51.32414 |
| ENSG00000102034 | | 9.087803 | 28.64705 | 25.90519 |
| ENSG00000125257 | | 16.39387 | 35.37663 | 53.35571 |
| ENSG00000272473 | | 7.031042 | 2.892751 | 2.697884 |
| ENSG00000145725 | | 2.114451 | 6.45032 | 11.91086 |
| ENSG00000198839 | | 3.587011 | 10.56569 | 12.24561 |
| ENSG00000198835 | | 8.719253 | 3.800741 | 3.48827 |
| ENSG00000154646 | | 2.360194 | 5.070404 | 6.560859 |
| ENSG00000081019 | | 3.243674 | 9.276406 | 8.485299 |
| ENSG00000168952 | | 8.114644 | 2.86823 | 2.609761 |
| ENSG00000214401 | | 6.832972 | 2.103165 | 2.887775 |
| ENSG00000048707 | | 6.22477 | 15.06659 | 14.25233 |
| ENSG00000090539 | | 11.52904 | 29.96802 | 29.09692 |
| ENSG00000145439 | | 2.090754 | 5.546796 | 6.141119 |
| ENSG00000106789 | | 15.30681 | 6.922274 | 6.019304 |
| ENSG00000228253 | | 385.0959 | 1015.98 | 2393.966 |
| ENSG00000238795 | | 3.385566 | 8.888731 | 10.17449 |
| ENSG00000253738 | | 2.365417 | 5.695763 | 11.57159 |
| ENSG00000144895 | | 45.34486 | 96.53768 | 93.64167 |
| ENSG00000180573 | | 15.81472 | 39.94202 | 36.93903 |
| ENSG00000213178 | | 9.251516 | 27.34181 | 30.87907 |
| ENSG00000169604 | | 151.2794 | 63.17478 | 68.10459 |
| ENSG00000143398 | | 126.7732 | 55.70304 | 63.37077 |
| ENSG00000114019 | | 186.7007 | 33.14326 | 53.55532 |
| ENSG00000198223 | | 20.14739 | 7.528271 | 8.928312 |
| ENSG00000142627 | | 340.3448 | 71.29536 | 93.06469 |
| ENSG00000113070 | | 9.560733 | 3.332738 | 2.847055 |
| ENSG00000094841 | | 4.924454 | 11.05879 | 13.32188 |
| ENSG00000074527 | | 35.01678 | 9.608791 | 14.69685 |
| ENSG00000153162 | | 73.72942 | 35.38839 | 18.08717 |
| ENSG00000230795 | | 5.614767 | 1.776237 | 1.667818 |
| ENSG00000210082 | | 2417.161 | 6094.444 | 12846.61 |
| ENSG00000221381 | | 17.27634 | 0.268058 | 0.206193 |
| ENSG00000241360 | | 15.41179 | 76.70679 | 112.6809 |
| ENSG00000205413 | | 1.934027 | 5.244119 | 21.82819 |
| ENSG00000210156 | | 9.454102 | 38.87694 | 61.32501 |
| ENSG00000225972 | | 12.42344 | 44.02619 | 26.5879 |
| ENSG00000200418 | | 1.51783 | 13.22041 | 9.936187 |
| ENSG00000257732 | | 16.02687 | 2.391289 | 3.225895 |
| ENSG00000251580 | | 2.484292 | 7.701867 | 6.2822 |
| ENSG00000122035 | | 17.13928 | 65.29326 | 56.04046 |
| ENSG00000137801 | | 29.25703 | 3.473638 | 3.840967 |
| ENSG00000070961 | | 21.39021 | 42.93575 | 43.54814 |
| ENSG00000164951 | | 19.38303 | 6.682982 | 7.788835 |
| ENSG00000119471 | | 27.58761 | 62.39102 | 62.90181 |
| ENSG00000006611 | | 5.946612 | 36.07842 | 15.76556 |
| ENSG00000273709 | | 3.125391 | 7.545579 | 62.6456 |
| ENSG00000108219 | | 28.7604 | 7.543307 | 5.752456 |
| ENSG00000212719 | | 11.48042 | 4.379372 | 5.518009 |
| ENSG00000100033 | | 23.39253 | 7.644488 | 6.949929 |
| ENSG00000173432 | | 15.16456 | 2.663166 | 3.017314 |
| ENSG00000125246 | | 1.71028 | 5.388276 | 7.097642 |
| ENSG00000234797 | | 17.25008 | 56.5445 | 38.4385 |
| ENSG00000156804 | | 13.48114 | 4.712052 | 4.845191 |
| ENSG00000140961 | | 25.03589 | 6.677604 | 10.30565 |
| ENSG00000171570 | | 8.550097 | 0.319772 | 0.458505 |
| ENSG00000181026 | | 58.80644 | 29.25505 | 26.173 |
| ENSG00000255513 | | 6.430089 | 14.36506 | 17.64134 |
| ENSG00000129315 | | 7.439802 | 16.82823 | 22.50156 |
| ENSG00000171155 | | 10.00044 | 24.4075 | 44.94605 |
| ENSG00000168078 | | 11.41051 | 25.90718 | 38.90052 |
| ENSG00000243137 | | 20.30757 | 1.968911 | 1.971032 |
| ENSG00000165389 | | 40.41221 | 183.9585 | 127.6985 |
| ENSG00000243885 | | 5.641873 | 1.982617 | 1.071669 |
| ENSG00000250920 | | 7.014958 | 3.408477 | 2.435303 |
| ENSG00000188010 | | 4.353026 | 12.71031 | 23.21903 |
| ENSG00000273088 | | 0 | 25.56524 | 29.23759 |
| ENSG00000162645 | | 1.955655 | 9.023298 | 8.51989 |
| ENSG00000047617 | | 26.37013 | 7.538409 | 3.520103 |
| ENSG00000226145 | | 18.43274 | 4.10813 | 5.336129 |
| ENSG00000133710 | | 3.487135 | 10.00046 | 7.552343 |
| ENSG00000263776 | | 5.672505 | 25.38893 | 22.18032 |
| ENSG00000115963 | | 85.41354 | 16.5572 | 28.95417 |
| ENSG00000109572 | | 11.97586 | 34.58646 | 28.49457 |
| ENSG00000034152 | | 126.3015 | 35.72795 | 27.95514 |
| ENSG00000206652 | | 5.42847 | 19.6412 | 110.3718 |
| ENSG00000227039 | | 6.795229 | 2.454772 | 2.570159 |
| ENSG00000101335 | | 101.2765 | 39.46577 | 44.47707 |
| ENSG00000132952 | | 3.523852 | 7.493647 | 9.995008 |
| ENSG00000283154 | | 36.72746 | 17.71559 | 11.09174 |
| ENSG00000200795 | | 0 | 25.02162 | 77.2717 |
| ENSG00000267922 | | 25.4126 | 4.801218 | 12.10887 |
| ENSG00000267697 | | 6.378196 | 0.151407 | 1.454776 |
| ENSG00000258056 | | 11.10449 | 5.475363 | 4.130512 |
| ENSG00000006606 | | 4.403841 | 10.27692 | 12.58313 |
| ENSG00000208772 | | 3.362806 | 8.528011 | 8.653254 |
| ENSG00000060749 | | 3.780136 | 10.21609 | 15.20413 |
| ENSG00000251537 | | 5.05874 | 0.119331 | 0.1325 |
| ENSG00000125347 | | 34.93863 | 16.7981 | 14.63556 |
| ENSG00000253368 | | 8.647322 | 3.753495 | 2.155086 |
| ENSG00000155111 | | 2.999806 | 7.156885 | 10.60319 |
| ENSG00000143322 | | 40.16933 | 17.52598 | 17.94267 |
| ENSG00000029153 | | 7.158309 | 2.415703 | 2.164209 |
| ENSG00000083123 | | 5.786042 | 11.58708 | 12.91782 |
| ENSG00000156475 | | 8.521544 | 3.441122 | 2.978706 |
| ENSG00000134287 | | 220.5344 | 78.09964 | 70.96488 |
| ENSG00000175197 | | 98.48229 | 38.03598 | 39.21118 |
| ENSG00000168398 | | 4.901495 | 18.94923 | 17.04003 |
| ENSG00000079257 | | 30.20948 | 129.315 | 83.47775 |
| ENSG00000248607 | | 12.48523 | 5.837608 | 6.129799 |
| ENSG00000163633 | | 15.71704 | 4.254445 | 5.443665 |
| ENSG00000262160 | | 0.23642 | 9.80634 | 10.77488 |
| ENSG00000139289 | | 19.51024 | 1.813883 | 3.554008 |
| ENSG00000247240 | | 2.650153 | 5.775959 | 5.802794 |
| ENSG00000166575 | | 2.129299 | 8.415404 | 6.705052 |
| ENSG00000095739 | | 26.84948 | 12.71399 | 13.36956 |
| ENSG00000213977 | | 66.82596 | 32.22167 | 19.59272 |
| ENSG00000055955 | | 2.062194 | 14.26144 | 9.707876 |
| ENSG00000092295 | | 24.61626 | 4.819242 | 6.438569 |
| ENSG00000179304 | | 38.43723 | 87.30881 | 89.58371 |
| ENSG00000231721 | | 11.77128 | 4.40085 | 4.414909 |
| ENSG00000162894 | | 8.205155 | 0.547955 | 2.125749 |
| ENSG00000047579 | | 59.90912 | 27.54139 | 23.8101 |
| ENSG00000132819 | | 33.54177 | 8.387429 | 10.56402 |
| ENSG00000173320 | | 1.487811 | 5.320085 | 5.642027 |
| ENSG00000210174 | | 6.451745 | 16.25554 | 151.4286 |
| ENSG00000138623 | | 29.16613 | 7.754066 | 11.48629 |
| ENSG00000268879 | | 101.0577 | 21.31878 | 42.74341 |
| ENSG00000185480 | | 4.045255 | 8.291643 | 11.07626 |
| ENSG00000139974 | | 2.135543 | 5.462656 | 5.843195 |
| ENSG00000137710 | | 25.45235 | 64.25623 | 55.43489 |
| ENSG00000127993 | | 2.521735 | 11.61225 | 13.14954 |
| ENSG00000201129 | | 8.343835 | 0.056684 | 0.060792 |
| ENSG00000162073 | | 21.26003 | 74.1468 | 97.43367 |
| ENSG00000177000 | | 9.562443 | 21.5137 | 20.19671 |
| ENSG00000272030 | | 6.126413 | 2.896429 | 1.018249 |
| ENSG00000077713 | | 12.69908 | 5.446009 | 5.373462 |
| ENSG00000179195 | | 19.34788 | 42.16477 | 57.20952 |
| ENSG00000173852 | | 8.630136 | 27.18322 | 30.18137 |
| ENSG00000148019 | | 5.787356 | 12.07522 | 13.65555 |
| ENSG00000275342 | | 10.18741 | 3.289503 | 2.898796 |
| ENSG00000101544 | | 6.5267 | 18.17913 | 18.11027 |
| ENSG00000257261 | | 7.389573 | 1.306551 | 1.51005 |
| ENSG00000170153 | | 2.603935 | 7.78548 | 6.229801 |
| ENSG00000186001 | | 30.83314 | 13.3457 | 14.53751 |
| ENSG00000161011 | | 753.3383 | 216.6722 | 348.844 |
| ENSG00000139112 | | 178.1454 | 45.25466 | 42.42945 |
| ENSG00000156467 | | 37.37625 | 92.2686 | 104.4214 |
| ENSG00000107338 | | 34.03703 | 9.474549 | 15.16379 |
| ENSG00000156463 | | 10.67283 | 4.711085 | 2.862316 |
| ENSG00000267261 | | 2.916716 | 8.548162 | 9.992003 |
| ENSG00000171421 | | 203.9693 | 85.13149 | 68.23988 |
| ENSG00000064270 | | 10.42383 | 4.683844 | 4.902624 |
| ENSG00000131171 | | 3.269634 | 9.801673 | 10.16038 |
| ENSG00000156136 | | 1.013593 | 5.84138 | 7.841392 |
| ENSG00000206878 | | 1.902349 | 8.182586 | 7.19428 |
| ENSG00000225345 | | 0.647332 | 5.866694 | 5.869371 |
| ENSG00000276612 | | 20.71474 | 0 | 0 |
| ENSG00000207513 | | 10.76639 | 38.70692 | 240.2064 |
| ENSG00000163395 | | 9.658869 | 1.01826 | 1.306962 |
| ENSG00000187634 | | 27.01196 | 104.9201 | 55.96544 |
| ENSG00000148773 | | 15.69159 | 51.25858 | 64.17832 |
| ENSG00000142871 | | 1064.849 | 56.62669 | 58.20179 |
| ENSG00000268460 | | 13.01766 | 1.365369 | 3.358186 |
| ENSG00000084731 | | 6.003907 | 2.222118 | 1.807974 |
| ENSG00000187185 | | 7.670444 | 3.164171 | 3.086006 |
| ENSG00000277459 | | 18.19439 | 8.471076 | 8.544538 |
| ENSG00000070404 | | 1121.674 | 345.0061 | 465.0621 |
| ENSG00000127329 | | 1.815496 | 7.455741 | 7.263943 |
| ENSG00000165929 | | 1.185057 | 5.929895 | 8.570413 |
| ENSG00000238597 | | 0.172254 | 10.64172 | 11.45138 |
| ENSG00000243988 | | 3.800638 | 8.549305 | 10.89001 |
| ENSG00000138166 | | 103.0572 | 14.39932 | 21.91795 |
| ENSG00000133466 | | 74.9159 | 34.32223 | 36.16414 |
| ENSG00000141750 | | 3.689662 | 14.41151 | 11.60668 |
| ENSG00000223975 | | 6.674026 | 15.80767 | 14.7615 |
| ENSG00000257671 | | 29.14229 | 12.41437 | 11.65764 |
| ENSG00000130766 | | 31.88035 | 8.644769 | 10.03956 |
| ENSG00000231887 | | 8.989506 | 4.474112 | 1.955506 |
| ENSG00000273143 | | 8.717273 | 2.090835 | 2.40489 |
| ENSG00000164674 | | 8.574103 | 2.954086 | 3.957109 |
| ENSG00000138182 | | 4.669056 | 11.93328 | 13.89666 |
| ENSG00000196196 | | 37.57282 | 13.73221 | 13.77002 |
| ENSG00000278189 | | 43.87384 | 367.9951 | 681.4319 |
| ENSG00000276850 | | 28.84908 | 12.65417 | 10.1843 |
| ENSG00000174282 | | 13.68937 | 48.69279 | 49.50184 |
| ENSG00000279821 | | 6.564902 | 2.548664 | 2.843364 |
| ENSG00000232995 | | 4.199763 | 11.56702 | 11.17805 |
| ENSG00000156453 | | 19.08578 | 7.251198 | 8.689102 |
| ENSG00000171161 | | 10.58061 | 42.58164 | 34.7164 |
| ENSG00000125148 | | 941.0363 | 243.3604 | 293.4985 |
| ENSG00000170345 | | 31.09114 | 143.682 | 97.40515 |
| ENSG00000239523 | | 16.3062 | 5.602302 | 5.189463 |
| ENSG00000269888 | | 8.70497 | 23.35609 | 18.97851 |
| ENSG00000167779 | | 54.6252 | 26.98938 | 25.59842 |
| ENSG00000167774 | | 60.14008 | 4.206734 | 0 |
| ENSG00000138399 | | 5.609192 | 11.66756 | 19.76714 |
| ENSG00000243566 | | 20.93468 | 5.24191 | 5.167593 |
| ENSG00000255339 | | 19.51516 | 58.6913 | 49.0627 |
| ENSG00000269955 | | 0 | 15.31743 | 5.505066 |
| ENSG00000106366 | | 89.70608 | 21.28414 | 17.70682 |
| ENSG00000254485 | | 7.733324 | 3.532033 | 2.347752 |
| ENSG00000167191 | | 5.625278 | 1.018464 | 2.078523 |
| ENSG00000148926 | | 220.381 | 108.7323 | 95.94464 |
| ENSG00000176170 | | 44.2212 | 21.77656 | 12.39645 |
| ENSG00000147168 | | 20.32346 | 5.840919 | 7.50805 |
| ENSG00000188647 | | 7.2114 | 14.57584 | 23.05429 |
| ENSG00000214900 | | 12.96652 | 3.401938 | 3.466837 |
| ENSG00000187193 | | 299.5215 | 69.19692 | 55.259 |
| ENSG00000251141 | | 2.680649 | 12.22977 | 7.846422 |
| ENSG00000004766 | | 10.46178 | 30.3349 | 24.68176 |
| ENSG00000249992 | | 11.49298 | 2.509922 | 1.52045 |
| ENSG00000186480 | | 43.29433 | 92.94971 | 124.1986 |
| ENSG00000258315 | | 4.643372 | 16.71951 | 17.54849 |
| ENSG00000138376 | | 3.806322 | 7.690441 | 9.998135 |
| ENSG00000275215 | | 38.84138 | 130.9144 | 498.5607 |
| ENSG00000132906 | | 82.68601 | 171.8865 | 38.65733 |
| ENSG00000204291 | | 21.4538 | 53.88449 | 51.37111 |
| ENSG00000233369 | | 24.35354 | 50.24997 | 51.8748 |
| ENSG00000270504 | | 19.53524 | 8.636332 | 9.190175 |
| ENSG00000138772 | | 155.0399 | 41.58538 | 59.93761 |
| ENSG00000223749 | | 15.0557 | 4.216588 | 5.407223 |
| ENSG00000273768 | | 7.345079 | 25.32631 | 154.4572 |
| ENSG00000198862 | | 4.071027 | 9.334195 | 15.05746 |
| ENSG00000183508 | | 3.370196 | 7.352125 | 8.82549 |
| ENSG00000244731 | | 12.86394 | 29.57844 | 41.18277 |
| ENSG00000116815 | | 3.752207 | 8.027303 | 19.81916 |
| ENSG00000137285 | | 15.9355 | 6.380656 | 5.857972 |
| ENSG00000074590 | | 10.90112 | 2.595947 | 4.29952 |
| ENSG00000077150 | | 283.4767 | 121.6136 | 140.3843 |
| ENSG00000005102 | | 5.861109 | 13.41238 | 20.76583 |
| ENSG00000283378 | | 2.104565 | 5.233351 | 7.939488 |
| ENSG00000148677 | | 28.95184 | 0.112719 | 0.317052 |
| ENSG00000206588 | | 5.356251 | 16.78253 | 101.4896 |
| ENSG00000206585 | | 6.664264 | 25.33263 | 162.7809 |
| ENSG00000257337 | | 4.694623 | 13.92993 | 19.96002 |
| ENSG00000120129 | | 669.8516 | 277.4541 | 195.6736 |
| ENSG00000117569 | | 2.736989 | 7.534044 | 7.415063 |
| ENSG00000284520 | | 5.362282 | 0.303376 | 0.304099 |
| ENSG00000284258 | | 9.956633 | 0.375786 | 0.381825 |
| ENSG00000277998 | | 0.457121 | 5.978623 | 10.37958 |
| ENSG00000155545 | | 2.248186 | 5.995603 | 16.53586 |
| ENSG00000033327 | | 17.65492 | 6.661526 | 8.696613 |
| ENSG00000265096 | | 16.75563 | 7.725533 | 5.356411 |
| ENSG00000235750 | | 10.92835 | 5.184176 | 4.944765 |
| ENSG00000277209 | | 5.633257 | 32.54404 | 276.0276 |
| ENSG00000182871 | | 10.48701 | 3.84564 | 4.897478 |
| ENSG00000256206 | | 7.654489 | 1.102352 | 1.290984 |

**Sup.Tab.5** KEGG analysis of RNA-seq data from SNX9-depleted RCTEC and control cells using genes affected by the two shRNAs (sh-1 and sh-2) and showed similar expression patterns.

| id | Description | Significant | Annotated | Pvalue | Qvalue |
| --- | --- | --- | --- | --- | --- |
| ko04390 | Hippo signaling pathway | 12/178 | 147/7279 | 0.000239 | 0.020028 |
| ko04668 | TNF signaling pathway | 11/178 | 122/7279 | 0.000183 | 0.020028 |
| ko04510 | Focal adhesion | 13/178 | 195/7279 | 0.00094 | 0.05245 |
| ko04380 | Osteoclast differentiation | 11/178 | 181/7279 | 0.004709 | 0.197017 |
| ko04350 | TGF-beta signaling pathway | 7/178 | 90/7279 | 0.006304 | 0.211034 |
| ko04722 | Neurotrophin signaling pathway | 8/178 | 123/7279 | 0.010335 | 0.241369 |
| ko04520 | Adherens junction | 6/178 | 76/7279 | 0.010444 | 0.241369 |
| ko04210 | Apoptosis | 9/178 | 151/7279 | 0.011537 | 0.241369 |
| ko00830 | Retinol metabolism | 5/178 | 58/7279 | 0.01331 | 0.24751 |
| ko04010 | MAPK signaling pathway | 13/178 | 281/7279 | 0.02 | 0.29907 |
| ko04670 | Leukocyte transendothelial migration | 7/178 | 114/7279 | 0.02137 | 0.29907 |
| ko04912 | GnRH signaling pathway | 6/178 | 89/7279 | 0.021443 | 0.29907 |
| ko04261 | Adrenergic signaling in cardiomyocytes | 8/178 | 146/7279 | 0.026434 | 0.310878 |
| ko04530 | Tight junction | 8/178 | 146/7279 | 0.026434 | 0.310878 |
| ko04391 | Hippo signaling pathway -fly | 5/178 | 70/7279 | 0.027862 | 0.310878 |
| ko04978 | Mineral absorption | 4/178 | 49/7279 | 0.031179 | 0.321291 |
| ko04212 | Longevity regulating pathway - worm | 5/178 | 73/7279 | 0.032634 | 0.321291 |
| ko00970 | Aminoacyl-tRNA biosynthesis | 4/178 | 55/7279 | 0.044893 | 0.416968 |
| ko04060 | Cytokine-cytokine receptor interaction | 12/178 | 286/7279 | 0.047335 | 0.416968 |
| ko04064 | NF-kappa B signaling pathway | 6/178 | 118/7279 | 0.068783 | 0.498598 |
| ko04624 | Toll and Imd signaling pathway | 3/178 | 39/7279 | 0.069197 | 0.498598 |
| ko00260 | Glycine, serine and threonine metabolism | 3/178 | 40/7279 | 0.073518 | 0.498598 |
| ko04810 | Regulation of actin cytoskeleton | 9/178 | 211/7279 | 0.07354 | 0.498598 |
| ko04921 | Oxytocin signaling pathway | 7/178 | 150/7279 | 0.074441 | 0.498598 |
| ko04115 | p53 signaling pathway | 4/178 | 66/7279 | 0.077455 | 0.498598 |
| ko04621 | NOD-like receptor signaling pathway | 4/178 | 66/7279 | 0.077455 | 0.498598 |
| ko04630 | Jak-STAT signaling pathway | 7/178 | 155/7279 | 0.085227 | 0.523219 |
| ko04623 | Cytosolic DNA-sensing pathway | 4/178 | 69/7279 | 0.087942 | 0.523219 |
| ko04071 | Sphingolipid signaling pathway | 6/178 | 128/7279 | 0.093229 | 0.523219 |
| ko00592 | alpha-Linolenic acid metabolism | 2/178 | 22/7279 | 0.099867 | 0.523219 |
| ko04913 | Ovarian Steroidogenesis | 3/178 | 47/7279 | 0.106893 | 0.523219 |
| ko04728 | Dopaminergic synapse | 6/178 | 133/7279 | 0.107003 | 0.523219 |
| ko04664 | Fc epsilon RI signaling pathway | 4/178 | 75/7279 | 0.110837 | 0.523219 |
| ko04260 | Cardiac muscle contraction | 4/178 | 76/7279 | 0.11489 | 0.523219 |
| ko00591 | Linoleic acid metabolism | 2/178 | 24/7279 | 0.115661 | 0.523219 |
| ko00524 | Neomycin, kanamycin and gentamicin biosynthesis | 1/178 | 5/7279 | 0.116464 | 0.523219 |
| ko04151 | PI3K-Akt signaling pathway | 12/178 | 337/7279 | 0.122209 | 0.523219 |
| ko00480 | Glutathione metabolism | 3/178 | 50/7279 | 0.122717 | 0.523219 |
| ko00980 | Metabolism of xenobiotics by cytochrome P450 | 4/178 | 78/7279 | 0.123188 | 0.523219 |
| ko00600 | Sphingolipid metabolism | 3/178 | 51/7279 | 0.128172 | 0.523219 |
| ko04214 | Apoptosis - fly | 3/178 | 51/7279 | 0.128172 | 0.523219 |
| ko04725 | Cholinergic synapse | 5/178 | 110/7279 | 0.131668 | 0.524692 |
| ko00750 | Vitamin B6 metabolism | 1/178 | 6/7279 | 0.138085 | 0.530009 |
| ko04923 | Regulation of lipolysis in adipocyte | 3/178 | 53/7279 | 0.139336 | 0.530009 |
| ko00630 | Glyoxylate and dicarboxylate metabolism | 2/178 | 28/7279 | 0.148898 | 0.545178 |
| ko04392 | Hippo signaling pathway - multiple species | 2/178 | 29/7279 | 0.157485 | 0.545178 |
| ko04745 | Phototransduction - fly | 2/178 | 29/7279 | 0.157485 | 0.545178 |
| ko00590 | Arachidonic acid metabolism | 3/178 | 57/7279 | 0.162585 | 0.545178 |
| ko04370 | VEGF signaling pathway | 3/178 | 57/7279 | 0.162585 | 0.545178 |
| ko04012 | ErbB signaling pathway | 4/178 | 87/7279 | 0.163455 | 0.545178 |
| ko04620 | Toll-like receptor signaling pathway | 5/178 | 119/7279 | 0.166125 | 0.545178 |
| ko04215 | Apoptosis - multiple species | 2/178 | 31/7279 | 0.174923 | 0.559176 |
| ko04122 | Sulfur relay system | 1/178 | 8/7279 | 0.179761 | 0.559176 |
| ko00062 | Fatty acid elongation | 2/178 | 32/7279 | 0.183754 | 0.559176 |
| ko04139 | Regulation of mitophagy - yeast | 2/178 | 32/7279 | 0.183754 | 0.559176 |
| ko00051 | Fructose and mannose metabolism | 2/178 | 33/7279 | 0.19265 | 0.559482 |
| ko04068 | FoxO signaling pathway | 5/178 | 126/7279 | 0.19514 | 0.559482 |
| ko00250 | Alanine, aspartate and glutamate metabolism | 2/178 | 34/7279 | 0.2016 | 0.559482 |
| ko04066 | HIF-1 signaling pathway | 4/178 | 95/7279 | 0.202676 | 0.559482 |
| ko04014 | Ras signaling pathway | 8/178 | 231/7279 | 0.2042 | 0.559482 |
| ko00561 | Glycerolipid metabolism | 3/178 | 64/7279 | 0.20572 | 0.559482 |
| ko00514 | Other types of O-glycan biosynthesis | 2/178 | 35/7279 | 0.210597 | 0.559482 |
| ko04966 | Collecting duct acid secretion | 2/178 | 35/7279 | 0.210597 | 0.559482 |
| ko04360 | Axon guidance | 6/178 | 165/7279 | 0.216627 | 0.565016 |
| ko00521 | Streptomycin biosynthesis | 1/178 | 10/7279 | 0.219432 | 0.565016 |
| ko00450 | Selenocompound metabolism | 1/178 | 12/7279 | 0.257195 | 0.611856 |
| ko00982 | Drug metabolism - cytochrome P450 | 3/178 | 72/7279 | 0.257709 | 0.611856 |
| ko03320 | PPAR signaling pathway | 3/178 | 73/7279 | 0.264342 | 0.611856 |
| ko04918 | Thyroid hormone synthesis | 3/178 | 73/7279 | 0.264342 | 0.611856 |
| ko00564 | Glycerophospholipid metabolism | 4/178 | 107/7279 | 0.265881 | 0.611856 |
| ko00565 | Ether lipid metabolism | 2/178 | 42/7279 | 0.274304 | 0.611856 |
| ko00603 | Glycosphingolipid biosynthesis - globo series | 1/178 | 13/7279 | 0.27539 | 0.611856 |
| ko00790 | Folate biosynthesis | 1/178 | 13/7279 | 0.27539 | 0.611856 |
| ko04072 | Phospholipase D signaling pathway | 5/178 | 146/7279 | 0.285958 | 0.611856 |
| ko00533 | Glycosaminoglycan biosynthesis - keratan sulfate | 1/178 | 14/7279 | 0.293141 | 0.611856 |
| ko03010 | Ribosome | 5/178 | 148/7279 | 0.295496 | 0.611856 |
| ko04622 | RIG-I-like receptor signaling pathway | 3/178 | 78/7279 | 0.297778 | 0.611856 |
| ko04726 | Serotonergic synapse | 4/178 | 113/7279 | 0.298776 | 0.611856 |
| ko04919 | Thyroid hormone signaling pathway | 4/178 | 113/7279 | 0.298776 | 0.611856 |
| ko04141 | Protein processing in endoplasmic reticulum | 6/178 | 185/7279 | 0.299013 | 0.611856 |
| ko04310 | Wnt signaling pathway | 5/178 | 149/7279 | 0.300285 | 0.611856 |
| ko04962 | Vasopressin-regulated water reabsorption | 2/178 | 45/7279 | 0.301654 | 0.611856 |
| ko04660 | T cell receptor signaling pathway | 4/178 | 114/7279 | 0.304311 | 0.611856 |
| ko00270 | Cysteine and methionine metabolism | 2/178 | 46/7279 | 0.310738 | 0.611856 |
| ko04973 | Carbohydrate digestion and absorption | 2/178 | 46/7279 | 0.310738 | 0.611856 |
| ko00280 | Valine, leucine and isoleucine degradation | 2/178 | 47/7279 | 0.319798 | 0.618584 |
| ko04330 | Notch signaling pathway | 2/178 | 48/7279 | 0.328829 | 0.618584 |
| ko04915 | Estrogen signaling pathway | 4/178 | 119/7279 | 0.332135 | 0.618584 |
| ko04512 | ECM-receptor interaction | 3/178 | 84/7279 | 0.338184 | 0.618584 |
| ko04640 | Hematopoietic cell lineage | 3/178 | 84/7279 | 0.338184 | 0.618584 |
| ko04152 | AMPK signaling pathway | 4/178 | 121/7279 | 0.34331 | 0.618584 |
| ko00604 | Glycosphingolipid biosynthesis - ganglio series | 1/178 | 17/7279 | 0.343841 | 0.618584 |
| ko04666 | Fc gamma R-mediated phagocytosis | 3/178 | 85/7279 | 0.344918 | 0.618584 |
| ko04270 | Vascular smooth muscle contraction | 4/178 | 122/7279 | 0.348902 | 0.618584 |
| ko04024 | cAMP signaling pathway | 6/178 | 197/7279 | 0.351115 | 0.618584 |
| ko04611 | Platelet activation | 4/178 | 124/7279 | 0.36009 | 0.627788 |
| ko04727 | GABAergic synapse | 3/178 | 91/7279 | 0.385142 | 0.662373 |
| ko00532 | Glycosaminoglycan biosynthesis - chondroitin sulfate / dermatan sulfate | 1/178 | 20/7279 | 0.390924 | 0.662373 |
| ko04750 | Inflammatory mediator regulation of TRP channels | 3/178 | 92/7279 | 0.3918 | 0.662373 |
| ko04977 | Vitamin digestion and absorption | 1/178 | 21/7279 | 0.40586 | 0.679281 |
| ko04974 | Protein digestion and absorption | 3/178 | 95/7279 | 0.411659 | 0.682166 |
| ko04211 | Longevity regulating pathway - mammal | 3/178 | 98/7279 | 0.431318 | 0.699482 |
| ko04730 | Long-term depression | 2/178 | 60/7279 | 0.433731 | 0.699482 |
| ko04614 | Renin-angiotensin system | 1/178 | 23/7279 | 0.434647 | 0.699482 |
| ko04723 | Retrograde endocannabinoid signaling | 3/178 | 100/7279 | 0.444294 | 0.708182 |
| ko01040 | Biosynthesis of unsaturated fatty acids | 1/178 | 24/7279 | 0.448515 | 0.708182 |
| ko04720 | Long-term potentiation | 2/178 | 65/7279 | 0.474871 | 0.729736 |
| ko04144 | Endocytosis | 8/178 | 306/7279 | 0.475186 | 0.729736 |
| ko00680 | Methane metabolism | 1/178 | 26/7279 | 0.475246 | 0.729736 |
| ko04011 | MAPK signaling pathway - yeast | 1/178 | 28/7279 | 0.500689 | 0.748306 |
| ko04724 | Glutamatergic synapse | 3/178 | 110/7279 | 0.507265 | 0.748306 |
| ko04062 | Chemokine signaling pathway | 5/178 | 192/7279 | 0.507768 | 0.748306 |
| ko00512 | Mucin type O-glycan biosynthesis | 1/178 | 29/7279 | 0.512946 | 0.748306 |
| ko00760 | Nicotinate and nicotinamide metabolism | 1/178 | 29/7279 | 0.512946 | 0.748306 |
| ko04111 | Cell cycle - yeast | 2/178 | 70/7279 | 0.514166 | 0.748306 |
| ko04662 | B cell receptor signaling pathway | 2/178 | 72/7279 | 0.529337 | 0.748696 |
| ko04976 | Bile secretion | 2/178 | 72/7279 | 0.529337 | 0.748696 |
| ko00030 | Pentose phosphate pathway | 1/178 | 31/7279 | 0.53657 | 0.748696 |
| ko04710 | Circadian rhythm | 1/178 | 31/7279 | 0.53657 | 0.748696 |
| ko04721 | Synaptic vesicle cycle | 2/178 | 73/7279 | 0.536801 | 0.748696 |
| ko04917 | Prolactin signaling pathway | 2/178 | 74/7279 | 0.544185 | 0.752722 |
| ko01230 | Biosynthesis of amino acids | 2/178 | 76/7279 | 0.558706 | 0.754581 |
| ko00052 | Galactose metabolism | 1/178 | 33/7279 | 0.559054 | 0.754581 |
| ko04130 | SNARE interactions in vesicular transport | 1/178 | 33/7279 | 0.559054 | 0.754581 |
| ko04015 | Rap1 signaling pathway | 5/178 | 208/7279 | 0.579547 | 0.771517 |
| ko00350 | Tyrosine metabolism | 1/178 | 36/7279 | 0.590763 | 0.771517 |
| ko04013 | MAPK signaling pathway - fly | 2/178 | 81/7279 | 0.593562 | 0.771517 |
| ko04911 | Insulin secretion | 2/178 | 81/7279 | 0.593562 | 0.771517 |
| ko04145 | Phagosome | 7/178 | 297/7279 | 0.59465 | 0.771517 |
| ko04140 | Regulation of autophagy | 1/178 | 37/7279 | 0.60082 | 0.773525 |
| ko04925 | Aldosterone synthesis and secretion | 2/178 | 85/7279 | 0.619947 | 0.776509 |
| ko00380 | Tryptophan metabolism | 1/178 | 39/7279 | 0.620203 | 0.776509 |
| ko04975 | Fat digestion and absorption | 1/178 | 39/7279 | 0.620203 | 0.776509 |
| ko03008 | Ribosome biogenesis in eukaryotes | 2/178 | 86/7279 | 0.626335 | 0.776509 |
| ko04970 | Salivary secretion | 2/178 | 86/7279 | 0.626335 | 0.776509 |
| ko03013 | RNA transport | 4/178 | 180/7279 | 0.646913 | 0.796124 |
| ko00071 | Fatty acid degradation | 1/178 | 43/7279 | 0.656205 | 0.801664 |
| ko00983 | Drug metabolism - other enzymes | 1/178 | 44/7279 | 0.664662 | 0.804677 |
| ko04540 | Gap junction | 2/178 | 95/7279 | 0.680127 | 0.804677 |
| ko04713 | Circadian entrainment | 2/178 | 95/7279 | 0.680127 | 0.804677 |
| ko00330 | Arginine and proline metabolism | 1/178 | 46/7279 | 0.680961 | 0.804677 |
| ko03015 | mRNA surveillance pathway | 2/178 | 96/7279 | 0.685699 | 0.804677 |
| ko00520 | Amino sugar and nucleotide sugar metabolism | 1/178 | 48/7279 | 0.696471 | 0.804677 |
| ko04340 | Hedgehog signaling pathway | 1/178 | 48/7279 | 0.696471 | 0.804677 |
| ko04550 | Signaling pathways regulating pluripotency of stem cells | 3/178 | 146/7279 | 0.698275 | 0.804677 |
| ko04922 | Glucagon signaling pathway | 2/178 | 99/7279 | 0.701941 | 0.804677 |
| ko00240 | Pyrimidine metabolism | 2/178 | 102/7279 | 0.717485 | 0.811381 |
| ko04916 | Melanogenesis | 2/178 | 102/7279 | 0.717485 | 0.811381 |
| ko03420 | Nucleotide excision repair | 1/178 | 54/7279 | 0.738646 | 0.821927 |
| ko00190 | Oxidative phosphorylation | 3/178 | 157/7279 | 0.744146 | 0.821927 |
| ko04514 | Cell adhesion molecules (CAMs) | 5/178 | 253/7279 | 0.747403 | 0.821927 |
| ko01212 | Fatty acid metabolism | 1/178 | 56/7279 | 0.751366 | 0.821927 |
| ko03022 | Basal transcription factors | 1/178 | 56/7279 | 0.751366 | 0.821927 |
| ko04113 | Meiosis - yeast | 1/178 | 57/7279 | 0.757493 | 0.823249 |
| ko04022 | cGMP - PKG signaling pathway | 3/178 | 162/7279 | 0.763097 | 0.823989 |
| ko02010 | ABC transporters | 1/178 | 59/7279 | 0.7693 | 0.825363 |
| ko00500 | Starch and sucrose metabolism | 1/178 | 60/7279 | 0.774988 | 0.825766 |
| ko01200 | Carbon metabolism | 2/178 | 116/7279 | 0.781287 | 0.825766 |
| ko04672 | Intestinal immune network for IgA production | 2/178 | 118/7279 | 0.789298 | 0.825766 |
| ko03050 | Proteasome | 1/178 | 63/7279 | 0.791228 | 0.825766 |
| ko04020 | Calcium signaling pathway | 3/178 | 171/7279 | 0.794346 | 0.825766 |
| ko04110 | Cell cycle | 2/178 | 125/7279 | 0.815344 | 0.837606 |
| ko00010 | Glycolysis / Gluconeogenesis | 1/178 | 68/7279 | 0.815744 | 0.837606 |
| ko04142 | Lysosome | 2/178 | 127/7279 | 0.822242 | 0.83913 |
| ko00562 | Inositol phosphate metabolism | 1/178 | 73/7279 | 0.837396 | 0.848349 |
| ko04971 | Gastric acid secretion | 1/178 | 74/7279 | 0.841412 | 0.848349 |
| ko04146 | Peroxisome | 1/178 | 82/7279 | 0.870188 | 0.870115 |
| ko04920 | Adipocytokine signaling pathway | 1/178 | 83/7279 | 0.873399 | 0.870115 |
| ko04910 | Insulin signaling pathway | 2/178 | 149/7279 | 0.884189 | 0.875653 |
| ko04610 | Complement and coagulation cascades | 1/178 | 93/7279 | 0.901465 | 0.88751 |
| ko04972 | Pancreatic secretion | 1/178 | 96/7279 | 0.908608 | 0.889312 |
| ko04070 | Phosphatidylinositol signaling system | 1/178 | 100/7279 | 0.917338 | 0.892563 |
| ko04150 | mTOR signaling pathway | 2/178 | 169/7279 | 0.922596 | 0.892563 |
| ko04114 | Oocyte meiosis | 1/178 | 114/7279 | 0.941855 | 0.905958 |
| ko03040 | Spliceosome | 1/178 | 171/7279 | 0.986219 | 0.939702 |
| ko00230 | Purine metabolism | 1/178 | 177/7279 | 0.988165 | 0.939702 |
| ko04080 | Neuroactive ligand-receptor interaction | 2/178 | 309/7279 | 0.996342 | 0.93988 |
| ko04650 | Natural killer cell mediated cytotoxicity | 2/178 | 402/7279 | 0.999583 | 0.93988 |
